# MRF: a tool to overcome the barrier of inconsistent genome annotations and perform comparative genomics studies for the largest animal DNA virus

**DOI:** 10.1186/s12985-023-02035-w

**Published:** 2023-04-18

**Authors:** Karthic Krishnan, Vinaya Kumar Katneni, Sudheesh K. Prabhudas, Nimisha Kaikkolante, Ashok Kumar Jangam, Upendra Kumar Katneni, Chris Hauton, Luca Peruzza, Shashi Shekhar Mudagandur, Vijayan K. Koyadan, Jithendran Karingalakkandy Poochirian, Joykrushna Jena

**Affiliations:** 1 Centre for Bioinformatics, Nutrition Genetics and Biotechnology Division, ICAR – Central Institute of Brackishwater Aquaculture, 75, Santhome High Road, MRC Nagar, RA Puram, Chennai, Tamil Nadu 600028 India; 2 The Center for Blood Oxygen Transport and Hemostasis, Department of Pediatrics, University of Maryland School of Medicine, Maryland, USA; 3 Ocean and Earth Science, National Oceanography Centre Southampton, University of Southampton Waterfront Campus, Southampton, UK; 4 Department of Comparative Biomedicine and Food Science, University of Padova, Legnaro, Padua, Italy; 5 Aquatic Animal Health and Environment Division, ICAR – Central Institute of Brackishwater Aquaculture, 75, Santhome High Road, MRC Nagar, RA Puram, Chennai, Tamil Nadu 600028 India; 6 Indian Council of Agricultural Research, New Delhi, India

**Keywords:** Virology, Comparative genomics, MRF, BLAST, Deleted CDS, Genome analysis

## Abstract

**Background:**

The genome of the largest known animal virus, the white spot syndrome virus (WSSV) responsible for huge economic losses and loss of employment in aquaculture, suffers from inconsistent annotation nomenclature. Novel genome sequence, circular genome and variable genome length led to nomenclature inconsistencies. Since vast knowledge has already accumulated in the past two decades with inconsistent nomenclature, the insights gained on a genome could not be easily extendable to other genomes. Therefore, the present study aims to perform comparative genomics studies in WSSV on uniform nomenclature.

**Methods:**

We have combined the standard *mummer* tool with custom scripts to develop missing regions finder (MRF) that documents the missing genome regions and coding sequences in virus genomes in comparison to a reference genome and in its annotation nomenclature. The procedure was implemented as web tool and in command-line interface. Using MRF, we have documented the missing coding sequences in WSSV and explored their role in virulence through application of phylogenomics, machine learning models and homologous genes.

**Results:**

We have tabulated and depicted the missing genome regions, missing coding sequences and deletion hotspots in WSSV on a common annotation nomenclature and attempted to link them to virus virulence. It was observed that the ubiquitination, transcription regulation and nucleotide metabolism might be essentially required for WSSV pathogenesis; and the structural proteins, VP19, VP26 and VP28 are essential for virus assembly. Few minor structural proteins in WSSV would act as envelope glycoproteins. We have also demonstrated the advantage of MRF in providing detailed graphic/tabular output in less time and also in handling of low-complexity, repeat-rich and highly similar regions of the genomes using other virus cases.

**Conclusions:**

Pathogenic virus research benefits from tools that could directly indicate the missing genomic regions and coding sequences between isolates/strains. In virus research, the analyses performed in this study provides an advancement to find the differences between genomes and to quickly identify the important coding sequences/genomes that require early attention from researchers. To conclude, the approach implemented in MRF complements similarity-based tools in comparative genomics involving large, highly-similar, length-varying and/or inconsistently annotated viral genomes.

**Supplementary Information:**

The online version contains supplementary material available at 10.1186/s12985-023-02035-w.

## Introduction

The white spot syndrome virus (WSSV) is the largest known animal virus with genome size ranging from 280 to 314 Kb and contains > 500 coding sequences. The broad host range for the virus is reviewed by Pradeep et al. [1] which includes a commercially significant entity, the penaeid shrimp whose fishery and culture contributes to global nutritional security and employment. As per the latest FAO yearbook of fishery and aquaculture statistics [2], the global shrimp production for the year 2019 was 6.55 million tonnes with an estimated value of USD 40.67 billion. Since its first report in early nineties the WSSV has caused huge economic losses to shrimp farming to the tune of billions of USD [3–8] in addition to the loss of employment as WSSV can induce 100% cumulative mortality in as little as 3 days’ time [9, 10].

The genomics studies with WSSV suffers from a peculiar problem of inconsistency in genome annotation nomenclature owing to its novel genome sequence, circular DNA as genetic material, variation in genome length between isolates and genome-wide deletions. Looking deeper, when the genome of WSSV was first deciphered in 2001 [11], its sequence did not show similarity to any other pathogen. Taxonomically, the issue was addressed by creating a new Genus, *Whispovirus* (*Whi* and *spo* taken from initial letters of White and Spot respectively) for WSSV under the Family, *Nimaviridae*, a new animal virus. The genome annotation was performed by annotating the coding sequences (CDS) serially with Arabic numerals and a common prefix like wsv001, wsv002, …, wsv526 in the order in which they were arranged in the genome. In the genomes published subsequently, other prefixes like WSSV (AF440570), ORF (MF768985) etc. were also used which led to the existence of different nomenclature systems for genomes of various WSSV isolates.

**Fig. 1:**
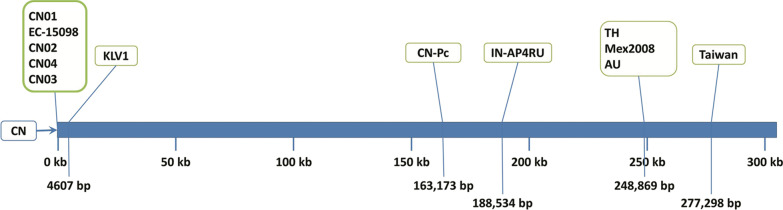
The start position of different WSSV genomes in comparison to WSSV-CN isolate. The horizontal blue bar is the representation for the sequence of WSSV-CN isolate. The start positions of all other isolates are marked relative to the sequence of WSSV-CN isolate

The circular nature of WSSV genome added further complexity to the annotation nomenclature. First, when the circular genomes were published as linear sequence, they did not have uniform start position (Fig. 1). Secondly, even if the sequence of isolates starts with same CDS and use the same prefix, the Arabic numeral for CDS changed from the very first instance of a deletion or insertion in one of the genomes. As the annotation nomenclature varied across the genomes, the results inferred on one WSSV genome could not be easily comparable to other genomes. This has remained a challenging area in comparative genomics studies of WSSV isolates. As mentioned in Wang et al. [12], for CDS in WSSV genome, a uniform annotated name exists only for 48 structural proteins and 41 non-structural proteins. Even for some of these proteins there are aliases like, VP13B for VP16, VP160A for VP190, VP36B for VP33, VP60A for VP56 etc. For example, a single CDS is annotated with different names like wsv162 in AF332093, WSSV218 in AF440570, hypothetical protein in KT995472, KT995470 and KU216744, wsv161 in KY827813, ORF90 in AF369029 and ORF215 in MF768985. Such a complex annotation situation warrants comparison of genomes on the basis of only genome sequences rather than on CDS nomenclature. We strongly believe that inconsistent annotation nomenclature between isolates acted as a hindrance to knowledge dissemination and better understanding of the virus. Therefore, the present study aims to overcome the complexities existing in current nomenclature while comparing different genomes of WSSV on uniform basis.

Another issue of concern is very high similarity (> 98%) between genome sequences of any two WSSV isolates (Additional file 1). Despite high similarity, WSSV genomes exhibit considerable variation in genome length, from 280,591 bp (MG702567; [13]) to 309,286 bp (KT995472; [14]). Therefore, in WSSV case, the tools capable of identifying the missing genomic regions and missing CDS between genomes are of high value than similarity search tools as the CDS missing in some isolates might be crucial for a better understanding of the pathogen. However, we do not have tools that could quickly tabulate and depict missing CDS in one genome when compared to another. This has motivated us to develop a general utility tool that helps in comprehensive analysis of WSSV genomes in one annotation nomenclature and also tabulates/depicts missing CDS profile, while comparing highly-similar, length-varying and inconsistently annotated genomes.

In this context, we have developed a tool that relies upon sequence similarity but differs from the existing ones by accounting for missing genomic regions located between identical regions and uses the annotation table to tabulate CDS present in those missing genomic regions. The tool, missing regions finder (MRF) principally developed to overcome the inconsistent annotation nomenclature of WSSV genomes has also been tested with other DNA/RNA virus genomes and the results are presented. The results implied the potential utility of MRF in virus research involving comparative genomics studies.

## Methods

### Workflow of MRF tool

MRF is developed as a virus comparative genomics tool that compares genomes and generates completely and partially missing coding sequences (CDS) in one genome (query) with respect to the other (reference) and presents them in a MirCos plot in addition to tabular output.

The MRF requires three input files for analysis: a query genome sequence, a reference genome sequence and its annotation table in gff3 format. First, the genome regions (GR) that have exact base matches between reference and query genomes are obtained with MUMmer (MUMmer, RRID:SCR_018171) run with *mum* option [15]. Using the three column’s output from MUMmer, for each of the matching GR, the end base positions of reference and query genomes are calculated as below:

$${\left({r}_{e}\right)}_{i}=\left[{\left({r}_{s}\right)}_{i}+{M}_{i}\right] -1$$

$${\left({q}_{e}\right)}_{i}=\left[{\left({q}_{s}\right)}_{i}+{M}_{i}\right] -1$$

where *i* = 1, 2,3,…, n (exact matching GR), *r*_*s*_ and *r*_*e*_ are the nucleotide start and end positions of reference genome, *q*_*s*_ and *q*_*e*_ are the nucleotide start and end positions of query genome, *M* is the length of matching GR between reference and query.

Based on the above coordinates, the missing GR or otherwise simply called as missing regions (MR) between two consecutive matching GR are calculated as follows,

$${G}_{i}=\left[{\left({r}_{s}\right)}_{i}-{\left({r}_{e}\right)}_{i-1}\right] -1$$

$${\left({R}_{s}\right)}_{i}={\left({r}_{e}\right)}_{i-1}+ 1$$

$${\left({R}_{e}\right)}_{i}=\left[{\left({r}_{s}\right)}_{i}+ {G}_{i}\right]-1$$

where *G* is the length of the sequence missing between two matching GR in the iterations *i* and *i* − 1, *R*_*s*_ and *R*_*e*_ are the nucleotide start and end positions of MR of reference.

The MR can be a negative or positive integer or single base pair mismatch. The negative values which result from overlapping matches are ignored along with single base pair mismatches, leaving only the positive values which are the true MR. Then MRF compares the base positions of the CDS in gff3 table with the MR and prints the missing CDS. Based on the positions they fall in, the output is arranged in two parts as completely and partially missing CDS. The partially missing CDS can be of three types depending on the deletions at the beginning, end or within the sequence (Additional file 2).

### Programmatic implementation of MRF

The core program which processes user inputs and generates the output is written in perl v5.20.2 and few minor parts in Bash v4.3.30(1)-release. The MRF tool is available as a web tool and also as command-line utility.

#### Graphical user interface (GUI)

The online version of MRF is developed using Bootstrap 4 and PHP 5.6.33 making the site fast, responsive and multi-device and multi-browser compatible. The website is hosted on a Debian GNU/Linux 3.2.96-2 operating system and intel x86_64 architecture. The home page takes all inputs from the user, upon submission, the inputs are processed by the core program and then the results page is displayed. Here, all the outputs can be downloaded and are available until the session expires.

#### Command line utility

A command line version of MRF is available for linux (Unix like systems). It is provided in two forms, single mode and batch mode, accessed by calling their respective bash wrapper scripts. In single mode, user can perform all the actions that are available in the GUI. The bash wrapper script calls the internal perl modules and also generates the configuration file for MirCos image, which user can use as such or modify it to suit their appeal. The batch mode of MRF offers the versatility of analyzing multiple query genomes against a reference and sort the results based on user defined criteria. In batch mode, MRF aggregates the missing regions and missing coding sequences of multiple queries and parses them using perl scripts. In addition, batch mode generates graphical outputs such as heatmap and stacked barplot using R scripts (see user manual, p25-48). Currently, batch mode is available only for command line version of MRF.

### Advanced features of MRF

#### Exact match length or mum length (-m)

It represents the minimum length of an exact match that mummer will find between query and reference genomes being compared. All the resulting matches are greater than or equal to the length being set [Default: 20]. The terms ‘exact match length’ and ‘mum length’ will be used interchangeably throughout this document. The default *exact match length* of 20 works best for most genomes. However, for genomes with high mutation rates such as RNA viruses, reducing the *exact match length* will result in proper matches which in turn prevent false reporting of missing coding sequences.

#### False match length cut off value (-l)

False exact matches tend to occur at random locations with shorter *mum length* leading to masking of true missing regions. To safe guard against finding false matches, MRF is equipped with two parameters, false match length and offset parameters. False match length is the length cut off, where matches less than or equal to the set value [default: 15] are screened further using the offset window options to determine if they are true matches or not.

#### Negative offset and positive offset (-n & -p)

These two parameters in advanced options determine whether the matches screened using ‘false match length cut off value’ parameter are truly a false match. It helps user to rule out false matches especially while running MRF with shorter *mum length*. When these options are defined, then for each exact match, MRF checks the specified number of exact matches upstream (negative offset) and downstream (positive offset) to the current exact match, for the base coordinates representing sequence contiguity of the query genome. If base coordinates are not contiguous then the current exact match would be treated as a false match. At the default setting [1, 1], a match in query genome is examined whether it is contiguous by looking at one upstream and one downstream match. Any match that doesn’t fall in between the upstream and downstream matches is deemed to be a false match.

### Output of MRF

#### Missing coding sequences

The MRF prints two tables listing the completely missing coding sequences and partially missing coding sequences (along with base position coordinates) in the query in comparison to reference genome. These tables can be downloaded using *Download* feature in *Dashboard***.**

#### Missing genomic regions

The MRF also prints the details of exact matches; missing genomic regions and point mutations located between two exact matches, if any. This table can be downloaded using *Download* feature in *Dashboard*.

#### Completely present coding sequences

This output can only be downloaded to a file but cannot be visualized in results page at web interface. This file consists of the list of completely present coding sequences in the query with respect to the reference.

#### MirCos plot

The MirCos plot is generated by utilizing Circos (Circos, RRID:SCR_011798) [16] libraries gives an overview of completely and partially missing CDS in the query genome. It is a graphical plot representing the reference genome (outer ring); the genes present on sense strand of reference genome (second ring from outside); the genes present on anti-sense strand of reference genome (third ring from outside); completely missing coding sequences (red), partially missing coding sequences (orange), and completely present coding sequences (yellow) of query genome (inner ring). The MirCos plot is available for download with and without the legend.

### Comparative analysis of WSSV genomes using MRF

White Spot Syndrome Virus exhibits a highly variable genome size between isolates. There is a length difference of about 28.6 Kb between the shortest and the longest genomes. To draw insights on the variable genome lengths, we have collected 13 complete WSSV genome sequences (Table 1) and analyzed them using on-line version of MRF tool with default parameters to extract the missing proteins between isolates. The China isolate, AF332093 was kept as the reference and the 12 other genomes were considered as queries. Nucleotide fasta sequences of the reference and a query along with the gff3 table of the reference were given as input for MRF tool.

**Table 1:** List of complete WSSV genomes used for comparative genomics analyses in this study using MRF tool

Accession	Country of origin	Isolate	Host	Genome length, bp	Sample collection year	References
MF768985	Australia	AU	*Penaeus monodon*	285,973	2016	[61]
KX686117	China	CN-Pc	*Procambarus clarkii*	300,223	2015	[60]
AF332093	China	CN	*Marsupenaeus japonicus*	305,119	1996	[11]
KT995470	China	CN02	*Procambarus clarkii*	294,261	2010	[14]
KT995471	China	CN03	*Litopenaeus vannamei*	284,148	2010	[14]
KT995472	China	CN01	*Marsupenaeus japonicus*	309,286	1994	[14]
KY827813	China	CN04	*Marsupenaeus japonicus*	281,054	2012	[37]
KU216744	Mexico	MEX2008	*Litopenaeus vannamei*	293,183	2008	[62]
AF440570	Taiwan	Taiwan	*Penaeus monodon*	307,287	1994	[63]
AF369029	Thailand	TH	*Procambarus clarkii*	292,967	1996	[33]
MG702567	India	IN_AP4RU	*Litopenaeus vannamei*	280,591	2013	[64]
JX515788	Korea	K-LV1	*Litopenaeus vannamei*	295,884	2011	[65]
MH090824	Ecuador	EC-15098	*Litopenaeus vannamei*	288,997	2015	[66]

### Phylogenomic analyses

The pathogenicity status of only three of 13 WSSV isolates is known through experimental challenge in host species [17]. Therefore, phylogenomic analyses have been conducted among the WSSV isolates to establish possible correlation, if any existing, between pathogenicity and phylogenetic relations. As WSSV genome is circular, initially the sequence of all 13 genomes was oriented to start with VP28 gene. Then multiple sequence alignment (MSA) was performed using MAFFT v7.305b [18] following FFT-NS-2 algorithm which is a fast and progressive method. The consensus alignment was analyzed in RAxML version 8.2.9 [19] with GTRGAMMAI model and random seed value of 12,345, to build maximum likelihood (ML) tree where the confidence of tree nodes was obtained after running 1000 bootstrap replications. With the same model, the consensus MSA was also analyzed in MrBayes 3.2.6 [20] to generate Bayes tree. For Bayesian inference, two simultaneous but completely independent runs that start from different random trees were executed with 4 chains for 10 million generations. The trees were sampled every 100 generations and convergence statistics were calculated every 1000 generations. First 25% of samples were discarded and hence not included for calculating summary statistics. The maximum standard deviation of split frequencies and potential scale reduction factor, PSRF were used to check the convergence of runs.

### Ranking important CDS with random forest machine learning model

A random forest machine learning model was deployed to rank the missing coding sequences that are important for classification of WSSV isolates to different clusters obtained in the phylogenetic tree. Each of the CDS that is completely deleted in one or the other query isolates as compared to CN isolate were coded as a binary categorical variable (0 = CDS deleted; 1 = CDS present). These categorical variables made out of deletion pattern of CDS were selected as independent variables in this analysis. The independent variables displaying similar deletion patterns across all WSSV isolates were removed from analyses except for one variable, to avoid redundancy in the feature set. A categorical dependent variable with three levels is made based on the clustering patterns of WSSV isolates in phylogenetic tree (for example, 1 for isolates in cluster 1, 2 for isolates in cluster 2 etc.). Then random forest model was run in Python’s scikit learn library v0.21.1 to rank the important independent variables.

### Homologous genes with host

The genome of WSSV was compared with the genomes of two susceptible hosts, *Penaeus vannamei* (assembly ASM378908v1) and *Eriocheir sinensis* (assembly ASM333651v1) and one completely resistant species, *Homo sapiens* (assembly GRCh38.p13) with the objective of finding homologous genes, if any, existing between genomes. A blast database was created separately for each of the three species with the gene datasets of *Penaeus vannamei* and *Homo sapiens* and genome of *Eriocheir sinensis* from NCBI using *make blast database* option in Blast2GO v4.1.9 [21]. Then a homology search was conducted between WSSV genes and each of the three blast databases using *blastn* option of Blast2GO v4.1.9 with an e value of 1e-03. The genes showing an alignment length of > 200 bp were selected as homologous genes.

### Validating MRF as a general utility tool

The primary intention of developing MRF tool is for comprehensive understanding of inconsistently annotated WSSV genomes where contrasting features of high genome similarity and variation in genome length exists across different isolates. However, its general utility has also been tested with other virus cases like, a DNA-virus with multigene families (African Swine Fever Virus, ASFV), a clinical RNA-virus (Human Immunodeficiency Virus-1, HIV-1) and a poultry virus (Marek’s disease virus, MDV) and the results are presented.

#### African swine fever virus

While analyzing ASFV genomes, major emphasis was to test MRF’s ability to find deletion profile of genes in MGF360 and MGF530/505 gene families which are crucial for virulence of the pathogen. For example, the OURT88/3 isolate has deletion of six copies of MGF360 and two copies of MGF505/530 compared to the virulent Benin 97/1 isolate [22]. Therefore, we used MRF to tabulate the deletion profiles of genes in 17 complete genomes of AFSV in comparison to highly virulent, Benin 97/1 isolate (Additional file 17: Table 4 at p. 44 and Fig. A3 at p. 80 in user manual).

#### Human immunodeficiency virus-1

Generally, the mutation rates in RNA genomes are higher than DNA genomes of viruses [23]. The mutations are likely to hinder the formation of alignments and exact matches in BLAST search and MRF run respectively. Therefore, we chose Human Immunodeficiency Virus (HIV) as an example case to test the suitability of MRF tool for handling RNA virus genomes. The Negative factor (Nef) gene of HIV genome plays a crucial role in progression of HIV infected individuals towards Acquired Immune Deficiency Syndrome, AIDS [24, 25]. Attenuation of HIV was observed in mutated strains carrying deletions in the Nef gene, where the onset of AIDS was also delayed [26, 27]. Here, we tracked the Nef gene in 9 HIV-1 type C strains (Additional file 17: Table A21 at p. 89 in user manual) keeping the refseq strain (Accession no. NC_001802) as reference, using MRF tool (refer Additional file 17: Fig. A6 at p. 91 in user manual).

#### Marek’s disease virus

In the case of MDV, an 18 bp deletion in UL49 gene differentiates a vaccine strain from a virulent strain [28]. Here, the utility of MRF was verified to detect deletions as shorter as 18 bp by comparing a vaccine strain (DQ530348) and a virulent strain (EF523390).

## Results

In this study, we present a new tool, missing regions finder (MRF) and demonstrate its utility to gain insights about WSSV genomes that have unclear annotation nomenclature. MRF is a unique tool where it directly outputs the deletions in CDS unlike most of the existing tools, which print similarities. This output is highly valuable while studying pathogenic viruses. In this study, the results of MRF were compared with the very popular and ubiquitously used similarity-search tool, the BLAST [29], for benchmarking purpose. The results with virus cases showed that MRF is effortless to use when comparing viruses.

### Comparative genomics of WSSV using MRF

The length of thirteen complete WSSV genomes analysed in this study varied from 280,591 to 309,286 base pairs. The GC content is mostly uniform ranging from 40.85 to 41.08%. The 13 isolates belong to 8 different countries. The CN isolate which is taken as reference in this study is one of the earliest sequenced genomes and longer than most other complete genomes (except CN01 and Taiwan) thus gave an opportunity to understand how the newly sequenced genomes have changed during the course of time. We have analyzed all the isolates against CN isolate using on-line version of MRF and tabulated the missing genomic regions and missing CDS which gave few interesting observations as detailed below.

Compared to CN isolate, the bases deleted in most other isolates are quite close to the absolute genome length difference between them (Additional file 3). However, there are certain isolates which differ, for example, the MEX2008 isolate is only 11,936 bases shorter but lost about 13,183 bases compared to CN isolate. Similarly, the IN_AP4RU and CN-Pc isolates which are shorter by 24,528 and 4896 bases but show deletions of 26,713 and 6429 bases respectively when compared to CN isolate. Therefore, it may be inferred that the longer genome does not necessarily have all the bases of the shorter genomes. This is very much evident in the case of CN01 and Taiwan isolates which are 4167 and 2168 bases longer than the reference but still lack 970 and 772 bases that are present in the CN isolate.

The MRF tool helped us to document the completely (Additional file 4) and partially (Additional file 5) missing CDS in all the 12 WSSV isolates in comparison to CN isolate. Importantly, the missing CDS are reported and commented in this manuscript in the annotation nomenclature of only CN isolate. The CN isolate has 524 CDS, out of which 82 CDS are either completely or partially lost in at least one of the other WSSV isolates. There are 46 CDS that show complete deletion in at least one isolate compared to CN isolate. Some of them are deleted in only one isolate while others in as many as 10 isolates (Table 2).

**Table 2:** Completely deleted coding sequences and the number of isolates showing deletion in comparison to CN isolate

Completely deleted CDS of WSSV	No. of isolates showing deletion
wsv060, wsv234, wsv235, wsv236, wsv242, wsv244, wsv319, wsv320, wsv337	1
wsv053, wsv074, wsv196, wsv245, wsv480, wsv500, wsv501, wsv503	2
wsv178, wsv179, wsv180, wsv338	3
wsv237	4
wsv238, wsv241	5
wsv239, wsv240, wsv462, wsv463, wsv485, wsv486, wsv487, wsv488	6
wsv481, wsv482, wsv483, wsv484	7
wsv489, wsv497	9
wsv490, wsv492, wsv493, wsv494, wsv495, wsv496, wsv498, wsv499	10

### Deletion hotspots in WSSV genome

**Fig. 2:**
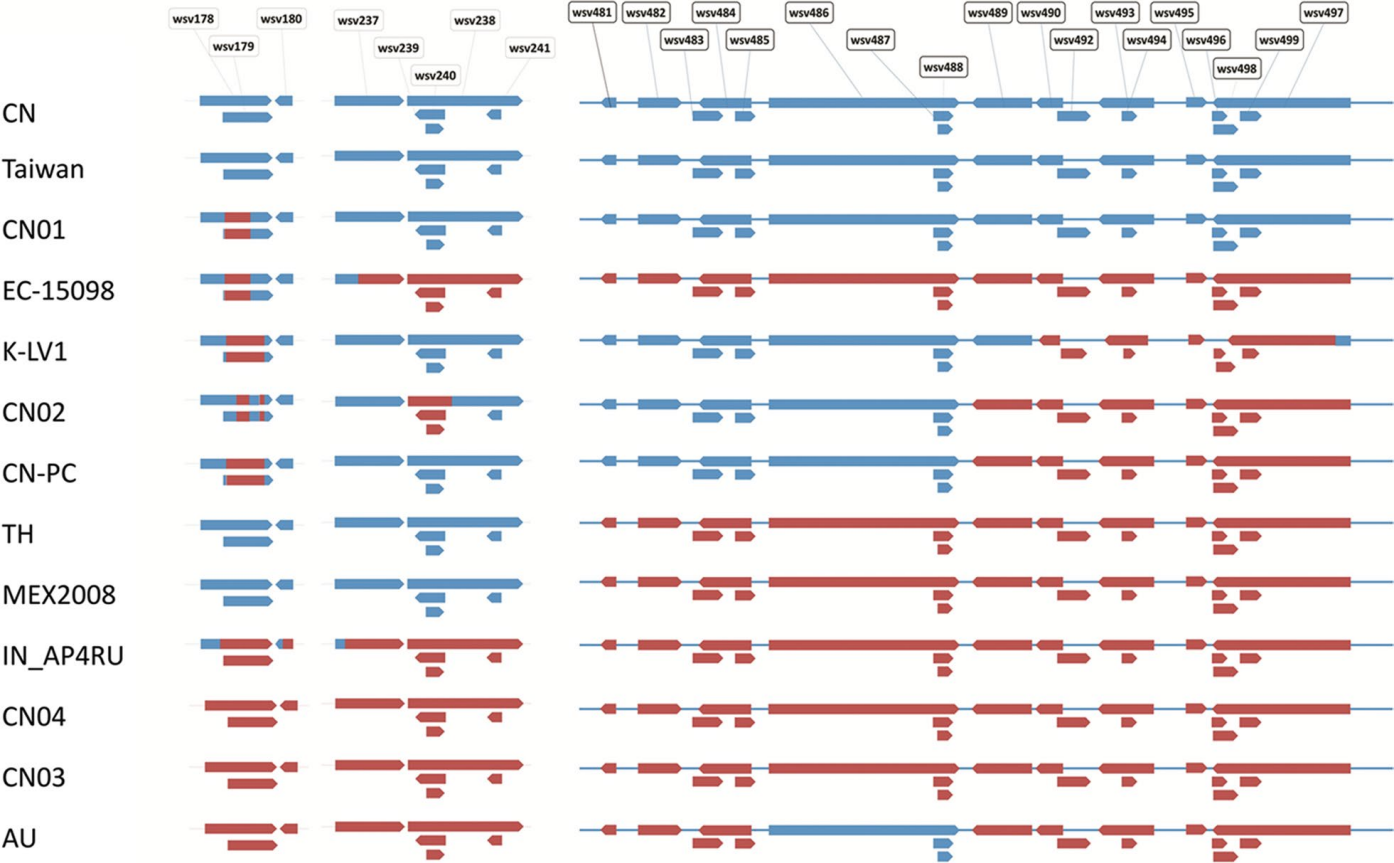
Depiction of the deleted base positions within the coding sequences of WSSV genomes. Red color indicates the stretch of the coding sequence that is deleted

In this study, the sequence regions where a stretch of 3 CDS are completely lost in three or more isolates are assumed as deletion hotspots. We report three deletion hotspots in WSSV genome which are wsv481/wsv499 (read as wsv481 through wsv499), wsv237/wsv241 and wsv178/wsv180 (Additional file 6). As MRF also prints the missing genomic regions coordinates it is possible to understand the position of deleted bases (5′ end or 3′ end or within) in the CDS present in deletion hotspots (Fig. 2).

#### wsv481/wsv499

The sequence region that encodes wsv481/wsv499 is lost in EC-15098, IN_AP4RU, MEX2008, TH and CN03 & CN04 isolates of China. These 6 isolates along with AU showed partial deletion (> 40%) of wsv479. The TH and MEX2008 isolates in particular share a common deletion of wsv480/wsv503 which includes additional CDS on either side of the deletion hotspot. The genomes of CN02, CN-Pc and AU isolates share a common deletion pattern ranging from wsv489 to wsv499. The K-LV1 isolate also shares similar deletion pattern except that wsv489 is present and wsv497 is partially (89%) lost. The CN01 and Taiwan isolates retained entire genomic region that included this deletion hotspot.

#### wsv237/wsv241

The Taiwan, CN01, K-LV1, CN-Pc, TH, and MEX2008 isolates did not show any deletions in this deletion hotspot which consists of two important envelope proteins VP41A (wsv237) and VP52A (wsv238). The genomes of AU, IN_AP4RU, EC-15098, CN02, CN03 and CN04 isolates have lost this segment in variable proportions. The wsv237 is only partially lost in IN_AP4RU and EC-15098 isolates. Another envelope protein, VP41B (wsv242) which is adjacent to this deletion hotspot is partially lost in EC-15098, CN03, CN04 and AU isolates whereas the same CDS is completely lost in IN_AP4RU isolate. Other CDS nearer to this deletion hotspot, the wsv234/wsv236 are completely lost in only CN02 isolate and wsv244 is lost only in IN_AP4RU isolate. The CN02 retained wsv241 and lost partial sequence of wsv238.

#### wsv178/wsv180

All three proteins in this deletion hotspot are completely lost in CN03, CN04 and AU isolates but all 3 proteins showed only partial deletions in IN_AP4RU isolate. The CN01, EC-15098, K-LV1, CN02 and CN-Pc isolates showed partial deletions in wsv178 and wsv179 only while retaining full coding sequence for wsv180. The Taiwan, TH and MEX2008 are not affected by this deletion hotspot. The wsv178 displaying partial to complete deletion in various isolates is an immediate early protein.

#### Other deletions

The annotated coding sequences and the genome-wide deleted regions in WSSV isolates have been depicted in Additional file 7. The CDS for wsv53 is deleted in CN01 and AU isolates whereas the CDS for wsv60 is deleted in EC-15098 isolate. Only CN03 and AU isolates showed deletion of CDS for wsv74 and wsv196 proteins. The coding sequence of two proteins, wsv319 and wsv320 are deleted in only IN_AP4RU isolate. The CDS of an envelope protein, VP62 (wsv338) is completely deleted in CN03, CN04 and AU isolates whereas the same isolates lost more than 95% of the CDS of another envelope protein, VP39 (wsv339). The VP39 was identified as integument protein [30] and studies show targeting this protein increases the survival rate in shrimps [31]. The coding sequence of protein, wsv337 was observed to be lost in only AU isolate. The genomes of K-LV1, CN-Pc, IN_AP4RU, CN03, CN04 and AU isolates completely lost the CDS for wsv462 and wsv463 proteins.

### Phylogenomic analyses

The ML and Bayes trees depicting evolutionary relations among WSSV isolates have been shown in Additional file 8. Three distinct clusters could be observed in ML tree where each cluster has at least an isolate from China. The clustering did not exhibit a geographical or time trend but it did indicate a trend about virulence of WSSV. The Bayes tree is in agreement with ML tree except for the position of Taiwan isolate in cluster 1.

### Important CDS identified with random forest model

The random forest model was used to obtain feature importance for all independent variables and rank them based on importance (Additional file 9). The top three CDS that are crucial to clustering of isolates as observed in phylogenetic tree were wsv237, wsv485 and wsv481. The coding sequences wsv486, wsv487 and wsv488 displayed similar deletion pattern as that of wsv485 and the CDS wsv482, wsv483 and wsv484 displayed similar deletion pattern as that of wsv481. Therefore, these 9 CDS are significant to clustering of isolates in a pattern that might be correlated to virulence of WSSV isolates.

### Homologous genes

**Fig. 3:**
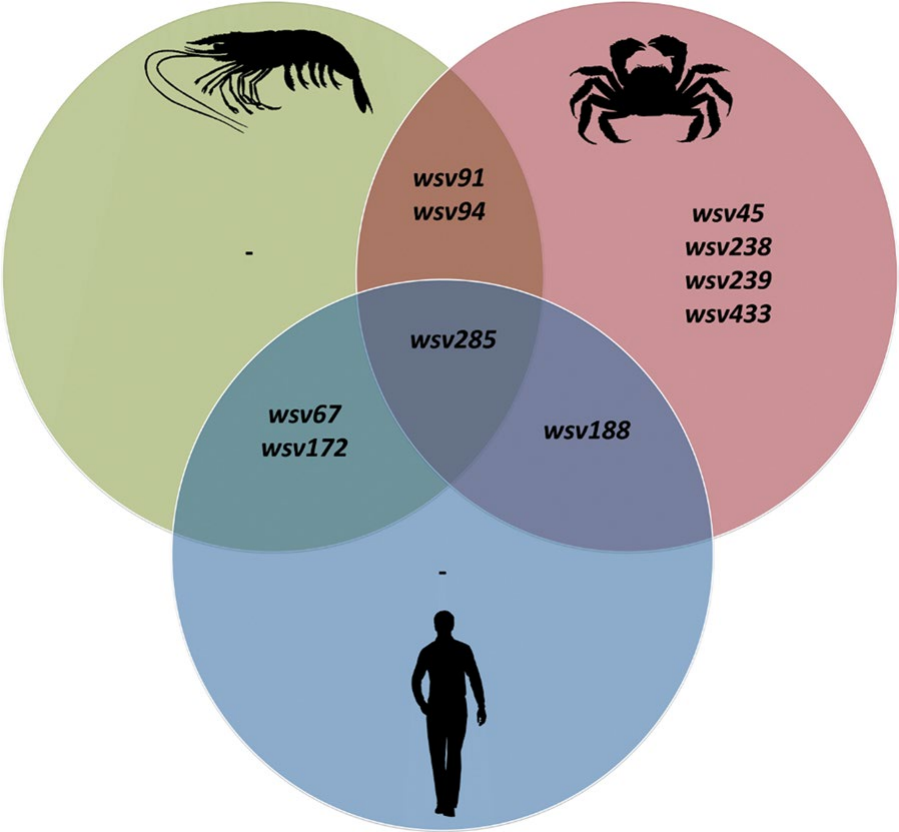
Homologous genes between WSSV and *Penaeus vannamei*, *Eriocheir sinensis* and *Homo sapiens*

It is of immense interest to know the homologous genes between the genomes of WSSV and its host, the shrimp. A blast search conducted for WSSV genes against the blast database of shrimp/human genes and Chinese mitten crab genome revealed the homologous genes (Fig. 3, Additional file 10). The sequence of a WSSV gene, wsv285, an unannotated protein showed homology in all the three species tested in the study. The human gene sequence showing homology to wsv285 is annotated as proline, glutamate and leucine rich protein 1 (PELP1).

Out of 524 coding sequences of CN isolate, only 4 of them showed significant (1e−05) similarity to shrimp genes. These four are wsv67 (thymidylate synthetase, 553 bp), wsv91 (immediate early protein, 240 bp), wsv172 (ribonucleotide reductase large subunit, 545 bp) and wsv285 (202 bp). All these genes except wsv91 also showed similarity to genes of the human genome. In addition, the wsv188, ribonucleotide reductase small subunit of WSSV also matches with similar gene of humans. The fragments of Chinese mitten crab genome showed similarity to wsv91, wsv188, wsv238 (VP52A) and few other unannotated genes of WSSV.

### Testing MRF with African swine fever virus

A single run of MRF in batch mode tabulated and depicted all the completely and partially deleted genes in query genomes compared to the reference genome (refer Additional file 17: Fig. A4 at p. 81 in user manual). Therefore, we could notice that apart from the MGF360 and MGF530/505 genes, many isolates also showed complete deletion of certain MGF110 family of genes. The low virulent isolates, OURT88/3 and NHV shared an identical deletion profile for both complete and partially deleted genes. Both of them did not have genes, MGF360-10L to MGF360-14L and MGF530/505-1R to MGF530/505-2R. The MGF530/505-3R gene is only partially lost in these isolates. Another avirulent isolate, Ba71V also shared similar deletion profile as that of OURT88/3 and NHV isolates, except that it still retained MGF530/505-2R and MGF530/505-3R genes and the MGF360-14L gene is only partially lost. The MGF110-12L and MGF110-13L genes have been found to be completely deleted in OURT88/3, NHV, Ba71V and tengani isolates. However it has been reported that the presence or absence of MGF110 family of genes does not alter the state of virulence of the virus [32]. The results highlight the importance of MRF to quickly obtain information related to the genetic variability among African swine fever virus isolates that differ in pathogenicity.

### Testing MRF with human immunodeficiency virus-1, HIV-1

In case of HIV-1 genomes, it was observed that all the query genomes suffered deletions of about 400 bp in Nef gene with the least deletion of 358 bp in KP109484 genome. Here, the utility of MRF was showcased to track the deletions in specific genes like Nef which is important information for researchers working with HIV virus.

### Testing MRF with Marek’s disease virus

Comparable results were obtained between MRF and BLAST for reporting minor but significant deletions as was demonstrated with the case of MDV. In the case of MDV, where an 18 bp deletion in UL49 gene differentiates a vaccine strain from a virulent strain [28], MRF had sufficient skill to find this deletion in partially missing coding sequences table (Additional file 11).

### Batch mode utility via command-line for MRF

The on-line tool of MRF has a limitation of being able to compare only two genomes in one go. Therefore, a command-line interface is also developed for MRF where multiple query genomes can be compared to a single reference genome in batch mode. The batch mode is of particular interest in virus cases where several genome sequences are available. The batch mode offers two additional output of significant value, the important *n* (any integer) number of query genomes and CDS that display maximum differences to a particular reference genome along with a heatmap of deletions. The batch mode of MRF was tested with WSSV (> 500 CDS and 13 genomes) that represent a good case for multiple CDS and genomes. Here, the MRF printed figures summarizing the most important 10 (or user specified) CDS/genomes that made query genomes different to the reference (Additional file 12). The output in batch mode of MRF helps researchers to find significant CDS/genomes to focus further rather than proceeding with huge datasets.

## Discussion

Unique sequence of WSSV genome and its circular nature led to different annotation nomenclature systems for various complete genome submissions. The comparative genome studies published for WSSV genomes are not easily comprehensible as different annotation nomenclature was followed in each case and this warrants performing genome-level analyses in only one nomenclature. As ICTV study group on *Whispovirus* considers WSSV-CN (AF332093) as exemplar isolate [12], we conducted comparative genomics analysis with WSSV genomes keeping WSSV-CN isolate as the reference and in its nomenclature system.

The morphology and sequence of different WSSV isolates are similar but huge variation exists in genome length. The average nucleotide identity (ANI) that indicates genome-wide similarity (Additional file 1) is very high (> 98%) between any two isolates. Several reports indicated variable virulence of different WSSV isolates [17, 33, 34]. Therefore, the features present in additional genome regions in longer genomes or missing genome regions in shorter genomes are valuable and should be focused in comparative genomics studies. In this regard, the MRF fills a void in the field of comparative genomics, where there is a dearth of suitable tools to analyze and interpret the genomic data. MRF stands out from other tools in this domain as it does not focus on what is present in a genome with respect to a reference but instead focuses on what is absent. Such a tool has also the potential to address the nomenclature issue concerning WSSV genomes, as users can compare several genomes against a single reference genome. In such case, results interpreted in the annotation nomenclature of a single reference genome would be easily comprehensible.

### Deletions in WSSV genomes

Beyond doubt, for WSSV case, it is inferred from this study that the longer genome need not necessarily have all the CDS that are present in shorter genome. Despite a length difference of about 25 Kb, the CN01 isolate does not have CDS of wsv486 (CN03 nomenclature) that is present in CN03 isolate. The study also documented 3 deletion hotspots in WSSV where a stretch of 3 CDS are completely lost in at least 3 isolates. Some of the previous reports have also documented similar major deletions in WSSV isolates [35, 36]. Few envelope proteins, VP41A, VP52A, VP35 and an immediate early protein are present in these hotspots. The deletion hotspot, wsv481/wsv499 which is a widely studied variable region is retained completely by CN, CN01 and Taiwan isolates when rest of other genomes has suffered major deletions. Sequence overlaps among certain CDS resulted in finding of partial deletion in longer coding sequence. For example, the partial deletion of wsv479 in several isolates could be due to the overlapping of wsv479 (279,829–281,358) with wsv480 (280,337–280,669) and wsv481 (281,196–281,390) which are completely deleted. So far there is no evidence associating the presence or absence of this segment with the virulence of WSSV. As majority of recently sequenced genomes lack many proteins in this stretch, probably the virus might not require them for infection and propagation.

### Significance of CDS retained by all WSSV isolates

One of the standard outputs tabulated by MRF tool is the CDS that did not exhibit any deletions. An interesting finding, we have observed with WSSV is the retention of certain CDS in all isolates that have possible or confirmed roles in virus pathogenesis. The structural proteins in WSSV are essential for maintaining the structure of virions. Of the major structural proteins, VP19, VP26 and VP28 have been retained in all isolates of WSSV without displaying even minor deletions whereas the envelope protein, VP24 is partially lost (47%) in CN04 isolate. Absence of VP24 in CN04 virions was hypothesized to be responsible for decreased peroral infectivity in *Litopenaeus vannamei* [37]. It is also postulated that VP24 may not be necessary for virus assembly or maintenance of viral structure as WSSV virions with normal morphology could be assembled without VP24 [37].

The *per os* infectivity factors (PIFs) are found to be essential for infectivity of insect midgut cells in case of baculoviruses. Four genes that are homologues to PIFs were previously identified in WSSV [38]. These are wsv35 (VP110), wsv115, wsv209 (VP187) and wsv306 (VP39A). All these four PIFs did not exhibit any deletion in all the WSSV isolates used in this study. Further, four of the WSSV proteins, wsv199, wsv222, wsv249 and wsv403 possess RING-H2 domain which is capable of functioning as E3 ubiquitin protein ligases [39] and help in virus pathogenesis. The coding sequences of these RING proteins whose ubiquitination activity has been proven in shrimp [40–43] did not exhibit deletion in any of the isolates. The VP9 (wsv230), a metal ion binding, non-structural protein capable of regulating transcription in WSSV [44] is retained by all isolates. Five WSSV proteins linked to nucleotide metabolism, wsv67, wsv112, wsv172, wsv188 and wsv395 [11] are again retained in all the isolates. Therefore, we hypothesize that the ubiquitination, transcription regulation and nucleotide metabolism might be few functions that might be essentially required for WSSV pathogenesis.

Recently, a few other nimaviral genome sequences have been deciphered from host genomic fragments which shared about 28 core genes when analyzed along with WSSV genome [45]. Of these 28 genes, 15 genes which are associated with virus gene expression, DNA replication and DNA packaging are retained in all WSSV isolates used in this study. Only exception is with wsv313 which showed partial deletion in only IN_AP4RU isolate. Other 13 genes are hypothetical proteins and not yet annotated. Several immediate-early (IE) genes that are expressed during early stages of WSSV infection play an important role in viral replication [46, 47]. These IE genes have been retained in all WSSV isolates considered in this study. Only exception is with wsv178 which is completely deleted in CN03, CN04 and AU isolates while showing partial deletions in many other isolates. Analyses of these structural proteins, PIFs, RING proteins, IE genes and other genes related to DNA replication and DNA packaging indicates that these are the major genes essential for virus and have been retained in all WSSV isolates used in the study though wide difference is noticed in genome length between isolates. The MRF tool helped in comprehensive analyses of WSSV isolates by quickly tabulating the missing CDS in all isolates in one annotation nomenclature.

### Marker sequences indicative of WSSV virulence

The phylogenomic analysis indicated the existence of three major clusters for WSSV isolates. The clusters did not have common geographical pattern or time of isolate collection. Interestingly, the clusters did indicate a correlation to the virulence of isolate. For three Chinese isolates, the virulence pattern is known [17], CN01 is high-virulent, CN02 is medium-virulent and CN03 is low-virulent. These 3 isolates with varying virulence are grouped into 3 different clusters. The virulence of other isolates in each cluster might be similar to that of virulence-known Chinese isolates. Only the challenge trials with biological specimens and isolates, could confirm this correlated observation. Earlier studies found that VP41B (wsv242) which functions as a transcriptional activator for *PjCaspase* [48], is an important effector caspase involved in apoptosis process against viral infection in shrimp [49]. This envelope protein is completely lost in IN_AP4RU and partially lost in EC-15098, CN03, CN04 and AU isolates. Incidentally CN03 is a low virulent isolate.

Assuming that the three clusters in phylogenetic tree indicate virulence of WSSV, a survey of the missing CDS has been conducted using the output of MRF tool. The CDS that exhibited complete deletion in all isolates belonging to each of the hypothesized high-virulent (cluster 1), medium-virulent (cluster 2) and low-virulent (cluster 3) clusters have been identified through MRF tool in the annotation nomenclature of CN isolate (Additional file 13). These 14 CDS and the top 3 ranked CDS identified as important features by random forest model, have been examined for possible clues to virulence. However, majority of these CDS are hypothetical proteins except wsv237 which codes for a structural protein, VP41A. Therefore, we believe that valuable functional information linked to degree of virulence is hidden in these hypothetical proteins which need to be explored.

Generally, animal viruses depend on envelope glycoproteins for cell attachment. But the major structural proteins of WSSV are not glycosylated [50]. Non-glycosylation of major structural proteins was also observed in case of ASFV where few minor structural proteins were found to be glycosylated [51]. Therefore, a search was performed for glycosylation sites in the 14 proteins that exhibited complete deletion in medium-virulent and low-virulent clusters compared to high-virulent cluster at NetOGlyc3.1 Server [52] and NetNGlyc1.0Server [53]. Four and nine of the WSSV proteins were predicted to contain N-glycosylation and O-glycosylation sites respectively (Additional file 14). Of these, as given in Additional file 15, six proteins were annotated with GO terms like viral nucleocapsid (wsv489, wsv493 and wsv494), envelope glycoprotein (wsv490) or integral component of membrane (wsv482 and wsv484). Therefore, these minor structural proteins comprising glycosylation sites might help in cell attachment in WSSV in the absence of major glycoproteins in WSSV envelope. Deletions of these minor glycoproteins in medium and low virulent isolates add more evidence on their role in WSSV pathogenesis.

### Significance of homologous genes

Acquisition of unique genes and gene duplication events were shown to be responsible for virulence and broad host range for WSSV [45]. Three of the major structural proteins of WSSV, the VP24, VP26 and VP28 were proposed to have evolved by gene duplication which later acquired different functions [54]. The analyses on homologous genes between WSSV and host species revealed other interesting facts in this direction. One class of genes that WSSV genome showed homology to other host and non-host species is wsv172 and wsv188, the ribonucleotide reductases (RNRs). In living organisms, the RNR gene helps in DNA synthesis by catalyzing the formation of deoxyribonucleotide triphosphates (dNTPs) from ribonucleotide triphosphates [55]. Of the four deoxyribonucleotides (dCMP, dGMP, dAMP and dTMP), only the formation of dTMP from dUMP requires an additional catalytic transfer of methyl group mediated by an enzyme, thymidylate synthetase [56]. The WSSV genome also has thymidylate synthetase, wsv67 which has showed similarity to shrimp and human thymidylate synthetase that catalyzes the synthesis of thymidylate, a precursor for DNA synthesis [57]. Many viruses lack thymidylate synthetase protein and depend on host machinery for viral replication. In humans, cell death was observed due to deficiency in intracellular concentration of thymidylate and subsequent stoppage of DNA synthesis, when thymidylate synthetase was suppressed [58]. Overall, the WSSV is capable of synthesizing all the four dNTPs required for the synthesis of DNA. The presence of these DNA synthesis related enzymes in WSSV might have benefitted the virus in viral genome replication, persistence of infection and capacity to infect a wide range of host [59].

Overall, for WSSV, where diverse nomenclature exists for different isolates, the MRF tool gave new insights on deleted CDS in various isolates in a single annotation nomenclature. As tables of deleted CDS can be obtained ready-made in MRF tool, the comprehension of any new WSSV genomes in terms of complete or partial deletions in important structural and functional genes would become easier. In brief, the findings of the study have proven the utility of MRF in case of a double-stranded DNA virus like WSSV that has problems in gene annotation.

### Merits displayed by MRF for WSSV case

**Fig. 4:**
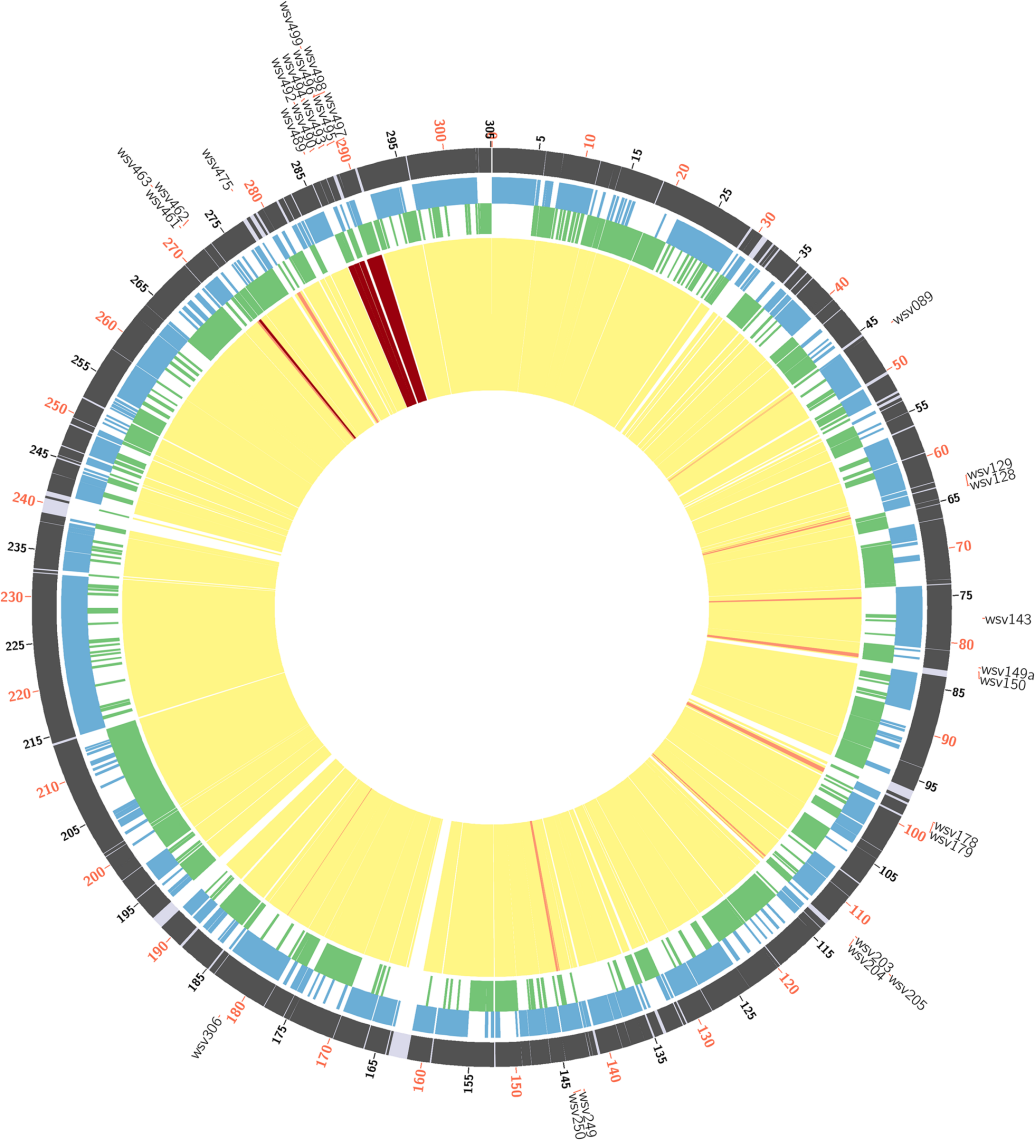
MirCos (Missing regions and Coding sequences) plot generated by comparing the WSSV genomes, AF332093 (reference) and KX686117 (query). The tracks represent coding sequences in reference genome (outer), coding sequences on sense strand (second), coding sequences on anti-sense strand (third) and missing coding sequences (inner track). The partially (orange lines) and completely missing (red lines) coding sequences in KX686117 genome in comparison to AF332093 genome can be observed in the inner track

While benchmarking MRF with BLAST using WSSV case, we noted that certain disadvantages displayed by BLAST like handling of low-complexity regions/repeat-regions, reporting false alignments and ignoring genome length differences can be overcome with the inbuilt features of MRF. For example, we have compared the WSSV accessions, AF332093 [11] and KX686117 [60] using both the MRF and the BLAST. The BLAST search reported a high similarity between genomes (99.76%) supported by 1242 alignments (p51 in user manual). So, with BLAST, the quest to map the base coordinates contributing to the 5 kb length difference between these two genomes would be a cumbersome exercise as one has to manually go through all the alignments considering the percentage identity and query coverage for each alignment. Added to this, the inconsistent start positions in linear sequence accession of these circular genomes pose further complications. The MRF, dependent on exact match algorithm directly tabulated the missing genomic regions and missing CDS and also depicted them in a MirCos plot (Fig. 4).

**Fig. 5:**
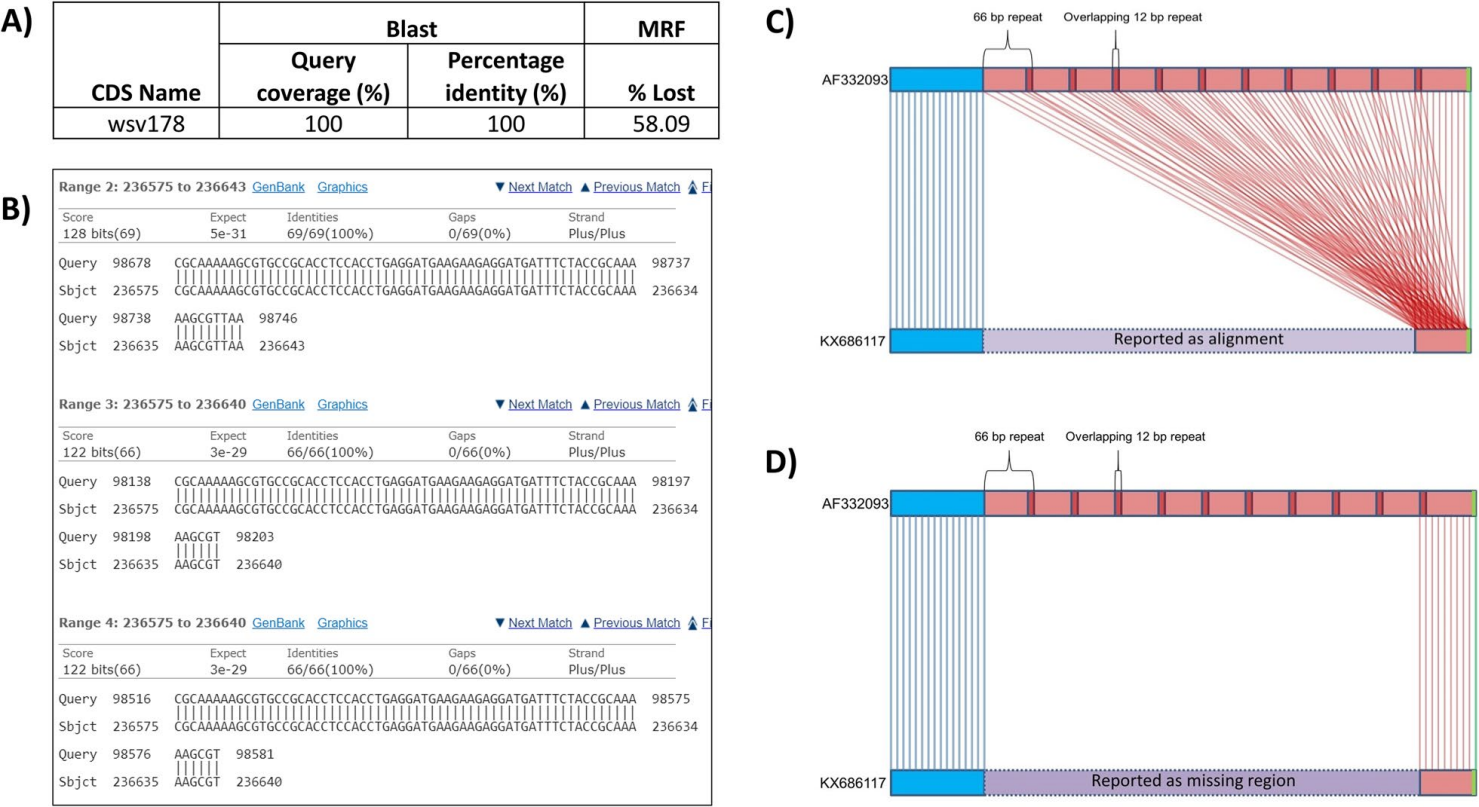
Illustration of MRF’s merit in handling repeat-rich regions, with WSSV case. For BLAST search, query is coding sequences of AF332093 genome and the subject is KX686117 genome. For MRF run, the query is KX686117 genome [300223 bp] and the reference is AF332093 genome [305119 bp]. **A** Summary of results from BLAST search and MRF run for the CDS, wsv178. In wsv178, there are overlapping repeat regions of 66 bp in AF332093 accession. Every repeat region shares a 12 bp sequence with previous repeat region. The BLAST reported a hit in subject genome with 100% similarity and 100% query coverage however MRF reported deletion of 58.09% of CDS length. **B** The alignments reported for wsv178 by BLAST. Here, several repeat regions in query genome made multiple alignments across single repeat region of subject genome leading to a positive hit with 100% query coverage. This lead to BLAST failing to report partial deletions in wsv178. As MRF relies on exact matches, it could report partial deletions in repeat regions of wsv178. **C** Depiction of repeat regions of wsv178 and the alignments made by BLAST between query and subject. **D** Depiction of repeat regions of wsv178 and the exact matches made by MRF between query and reference genomes

Here, for certain CDS such as wsv095 and wsv164, BLAST could retrieve hits only in runs with reduced word length [11] as alignments of these CDS have low-complexity regions and are ignored in default run (Additional file 16). In case of wsv178, a CDS with repeat-rich regions, BLAST failed to report partial deletions because several repeat regions in query genome made multiple alignments across a single repeat region of subject genome leading to a positive hit with 100% query coverage (Fig. 5). As MRF relies on exact matches, it could report hits to wsv095 and wsv164 and could identify partial deletions in repeat regions of wsv178 in standard run with default parameters.

### Insights with African swine fever virus case

**Fig. 6:**
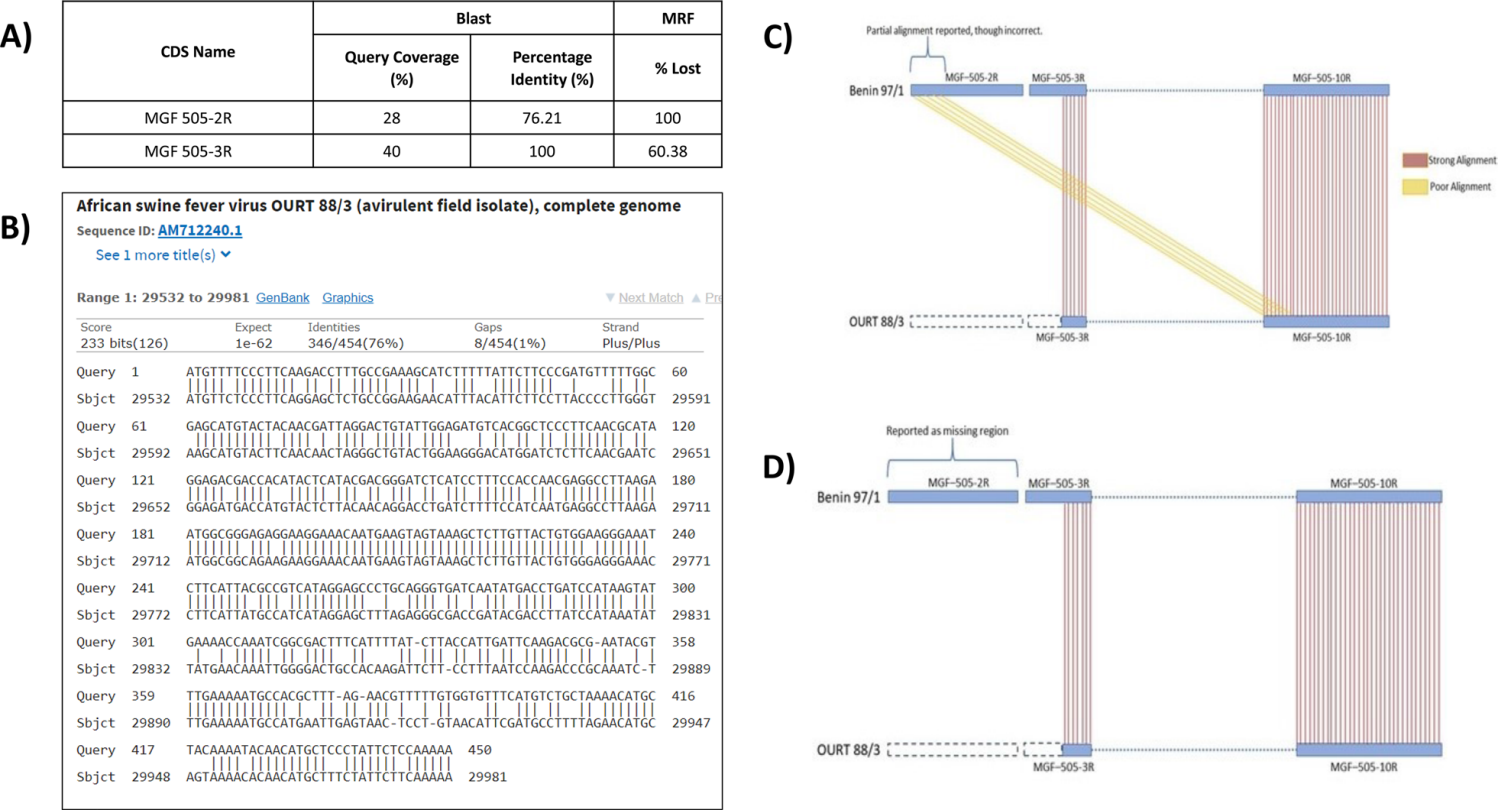
Illustration of MRF’s merit in handling highly-similar regions, with ASFV case. For MRF run, the query is AM712240 genome [171719 bp, Strain: OURT 88/3] and the reference is AM712239 genome [182284 bp, Strain: Benin 97/1]. For BLAST search, query is coding sequences of AM712239 genome and the subject is AM712240 genome. **A** Summary of results from BLAST search and MRF run for the CDS, MGF 505-2R. For MGF 505-2R, it was reported as lost by MRF whereas BLAST reported a poor alignment. **B** The alignment printed by BLAST for MGF 505-2R. When the coordinates of the hit region were further examined, it was found to be the sequence of another CDS, MGF 505-10R. This is actually a false alignment between MGF 505-2R of the query and MGF 505-10R of the subject. **C** Depiction of the alignment (yellow lines) made by BLAST between MGF 505-2R of the query and MGF 505-10R of the subject. **D** Depiction of the exact matches made by MRF between query and reference genomes

While comparing the non-pathogenic (OURT88/3, AM712240) and highly pathogenic (Benin 97/1, AM712239) ASFV isolates varying in genome length, we noticed that MRF handles highly similar genes better than BLAST search. It is observed that the BLAST reported false alignments for certain CDS (for example, MGF 505-2R gene in OURT 88/3 isolate). For ‘MGF 505-2R’, MRF printed a completely missing report whereas BLAST reported a weak alignment. Examination of CDS annotation of OURT88/3 genome revealed that it does not have ‘MGF 505-2R’. Further, when the coordinates of the hit region were examined, it was found to be the sequence of ‘MGF 505-10R’. This is an alignment between MGF 505-2R of the query and MGF 505-10R of the subject. Here BLAST search erred by falsely reporting an alignment with MGF-505-10R as a hit to MGF 505-2R. As the sequence of MGF 505-2R shares similarity with the sequence of MGF 505-10R, the BLAST with its heuristic nature of the algorithm is able to find a weak match. As the false matches were controlled by offset parameters of MRF, it could accurately print ‘completely missing’ report for MGF 505-2R (Fig. 6). Detection of false alignments in BLAST output requires tedious task of inspecting all the individual alignments but the advanced features of MRF automatically takes care of false exact matches without need of any further steps.

### Insights with other virus cases

In case of HIV-1 virus genomes with high mutations, we have observed that both MRF and BLAST struggled to avoid false matches/alignments respectively. Here, setting a lower word length in BLAST and lower mum length in MRF lead to false alignments and false matches respectively. However, the offset parameters in MRF helped to control most of the false matches as demonstrated with results of several re-runs (pp. 14–22 in user manual). With MDV use case, we have demonstrated that the MRF had sufficient skill to find a short deletion of 18 bp and display it in partially missing coding sequences table (Additional file 11).

### Limitations

A limitation we noticed with MRF is the reporting of inflated ‘missing genome lengths’ as it cannot detect matching genomic regions shorter than the exact match length. But the shorter *mum* lengths and offset parameters help MRF to overcome this drawback to a great extent. It might be argued that the missing genomic regions are not really missing instead not covered in assembly due to low sequence coverage. That is true to an extent when we had mostly contig-level genomes in public repositories. In recent years, developments in sequencing technologies have contributed to data explosion that led to publication of scaffold/chromosome-level genomes especially for viruses which have smaller genome lengths compared to others. The argument becomes void when more and more full-length genomes accumulate in public repositories.

## Conclusions

Pathogenic virus research benefits from tools that could directly indicate the missing genomic regions and CDS between isolates/strains. Existing comparative genomic tools primarily focus on genomic similarities. Often, the loss or gains of genomic regions are attributed to the degree of virulence. In viruses with more CDS, knowledge of those major CDS differing between genomes helps researchers to quickly focus on them first. Same is the case with viruses having a high number of genomes, where advances would be rapid, if focus can be brought down quickly to few genomes. The MRF complements similarity-based alignment tools in comparative genomics involving large, highly-similar, length-varying and/or inconsistently annotated viral genomes. To our knowledge, there is no other tool that instantly prints and depicts missing coding sequences in a query compared to a reference genome. In this study, with certain important virus use cases, we have demonstrated the advantage of MRF in providing detailed graphic/tabular output in less time and also in handling of highly similar, low-complexity and repeat-rich regions of the genomes. Further, MRF offers a choice for subject-matter specialists to quickly compare virus genomes of their interest and derive functionally meaningful inferences.

More research needs to be invested in to development of tools that focus on missing genomic regions and point mutations. In virus research, MRF provides advancement to find the differences between genomes and to quickly identify the important CDS/genomes that require early attention from researchers. There is scope to integrate options for detecting point mutations and the associated aminoacid changes in to the MRF tool. To conclude, MRF complements similarity-based tools like BLAST in virus research.

## Supplementary Information

**Additional file 1.** Average Nucleotide Identity estimates among various WSSV isolates used in the study. The ANI estimates were calculated using ANI calculator maintained at Kostas lab (http://enve-omics.ce.gatech.edu/ani/).

**Additional file 2.** Depicting of scenarios for complete and partially missing coding sequences as implemented in MRF tool. Each arrow in the figure indicates a coding sequence (CDS). When a CDS coordinates fall within a gap’s position, it is identified as completely missing feature. Whereas when a CDS position doesn’t fall perfectly within a gap region, it means only a part of it ends up deleted (partially missing feature, scenario 1 to 3). This can happen in three different ways. In the first scenario, the gap region starts downstream to the coding sequence, extends into it and ends within the coding sequence. The second scenario is opposite to the first, where the gap region starts from within the coding sequence and extends beyond it. In the last scenario, the gap region starts and ends within the coding sequence.

**Additional file 3.** Comparison of genome length difference (blue line) and base length deleted (red line) in WSSV isolates with respect to CN isolate as identified by MRF tool.

**Additional file 4.** The list of completely missing coding sequences as identified by using MRF tool in WSSV isolates in comparison to the reference, the CN isolate.

**Additional file 5.** The list of partially missing coding sequences as identified by using MRF tool in WSSV isolates in comparison to the reference, the CN isolate.

**Additional file 6.** Heat map of the deleted CDS and deletion hot spots in WSSV genomes. Each row represents one WSSV isolate and each column represents one coding sequence.

**Additional file 7.** Briggs image depicting the deletion patterns of important coding sequences in various WSSV isolates.

**Additional file 8.** Maximum likelihood (A) and Bayes (B) trees built using complete WSSV genomes. The branch lengths are shown above and bootstrap/clade credibility values are shown below the branches.

**Additional file 9.** **Sheet 1.** The data used for running random forest machine learning model. Only the coding sequences (CDS) that were deleted in all isolates of a phylogenetic cluster are used for this analysis. The CDS gets a code of 1 or 0 based on the presence or deletion respectively in an isolate. **Sheet 2.** The deletion pattern of certain CDS is same as that of others. Hence the redundant CDS are removed from analysis. This sheet contains the non-redundant data actually used in running random forest model. **Sheet 3**. List of genes displaying similar deletion trends as that of other genes. **Sheet 4.** The feature importance extracted in random forest machine learning model.

**Additional file 10.** Results of the blast search conducted for WSSV genes against the blast database of shrimp/human genes and Chinese mitten crab genome to find the homologous genes.

**Additional file 11.** Utility of MRF in finding minor deletions of significance explained with a case of Marek’s disease virus. For MRF run, the query is DQ530348 genome [178311 bp; GHV-2 CVI988/Rispens vaccine strain] and the reference is EF523390 genome [178246 bp, GHV-2 RB-1B virulent strain]. For BLAST search, query is coding sequences of EF523390 genome and the subject is DQ530348 genome. The MRF is able to find a partial deletion of 18 bp (table given above) which is significant to this virus. The same deletion was observed in the alignment generated by BLAST as well (alignment given below). Here, MRF fared on par with the BLAST.

**Additional file 12.** Output of MRF run in batch mode, WSSV, case with high number of coding sequences. Here 11 query genomes (AF369029, AF440570, JX515788, KT995470, KT995471, KT995472, KU216744, KX686117, KY827813, MF768985, MG702567) are compared to a reference genome (AF332093) in one go. **A** Bar plot of complete and partially missing CDS length (bases) in query genomes. **B** Heatmap showing the ten genomes that exhibited high missing genome lengths and the CDS contributing to missing genome lengths. The heatmap is accompanied by a clustering of genomes based on missing genome regions.

**Additional file 13.** The coding sequences that are completely deleted in all WSSV isolates of each of the three clusters obtained in phylogenetic tree. In this Venn diagram, left-top circle represents cluster 1 (high-virulent), right-top circle represents cluster 2 (medium-virulent) and bottom circle represents cluster 3 (low-virulent).

**Additional file 14.** List of coding sequences displaying complete deletion in certain isolates and which are harboring glycosylation sites.

**Additional file 15.** Functional annotations of missing coding sequences in few WSSV isolates.

**Additional file 16.** Illustration of MRF’s merit in reporting results for coding sequences having low complexity regions with WSSV case (highly-similar but length-varying genomes). For BLAST search, query is coding sequences of AF332093 genome and the subject is KX686117 genome. For MRF run, the query is KX686117 genome [300223 bp] and the reference is AF332093 genome [305119 bp]. **A** Summary of results from BLAST search and MRF run for 2 CDS, wsv094 and wsv164. Blast failed to report hits for wsv095 and wsv164 at default parameters (word length = 28). Whereas MRF prints nil deletion in query genome for these 2 coding sequences. **B** BLAST prints alignment for wsv094 when run with a reduced word length of 11. **C** BLAST prints alignment for wsv164 when run with a reduced word length of 11. As indicated in alignments, even though there were perfect matches that were greater than the length 28 (as shown in **B** and **C**), they were not considered as High Scoring Portions (HSPs) in default run as the alignments have low-complexity regions.

**Additional file 17.** User manual developed for MRF tool.

## Data Availability

Download MRF at https://github.com/vinayciba/MRF or access online at http://14.139.181.163/mrf.

## Abbreviations

CDS: Coding sequences
MRF: Missing regions finder
WSSV: White spot syndrome virus
FAO: Food and Agricultural Organisation
USD: United States dollar
ORF: Open reading frame
GR: Genome regions
MR: Missing regions
GUI: Graphical user interface
MSA: Multiple sequence alignment
ML: Maximum likelihood
PSRF: Potential scale reduction factor
ASFV: African swine fever virus
HIV: Human immunodeficiency virus
AIDS: Acquired immune deficiency syndrome
MDV: Marek’s disease virus
Nef: Negative factor
BLAST: Basic local alignment search tool
PELP1: Proline, glutamate and leucine rich protein 1
ICTV: International Committee on Taxonomy of Viruses
ANI: Average nucleotide identity
PIFs: Per os infectivity factors
IE: Immediate-early
RNRs: Ribonucleotide reductases
dNTPs: Deoxyribonucleotide triphosphates
dCMP: Deoxycytidylic acid
dGMP: Deoxyguanosine monophosphate
dAMP: Deoxyadenosine monophosphate
dTMP: Deoxythymidine monophosphate
dUMP: Deoxyuridine monophosphate
