# Supplementary figures and images for: MRF: a tool to overcome the barrier of inconsistent genome annotations and perform comparative genomics studies for the largest animal DNA virus
Source: Virol J. 2023 Apr 18;20:72. doi: 10.1186/s12985-023-02035-w (PMC10111743; doi:10.1186/s12985-023-02035-w)

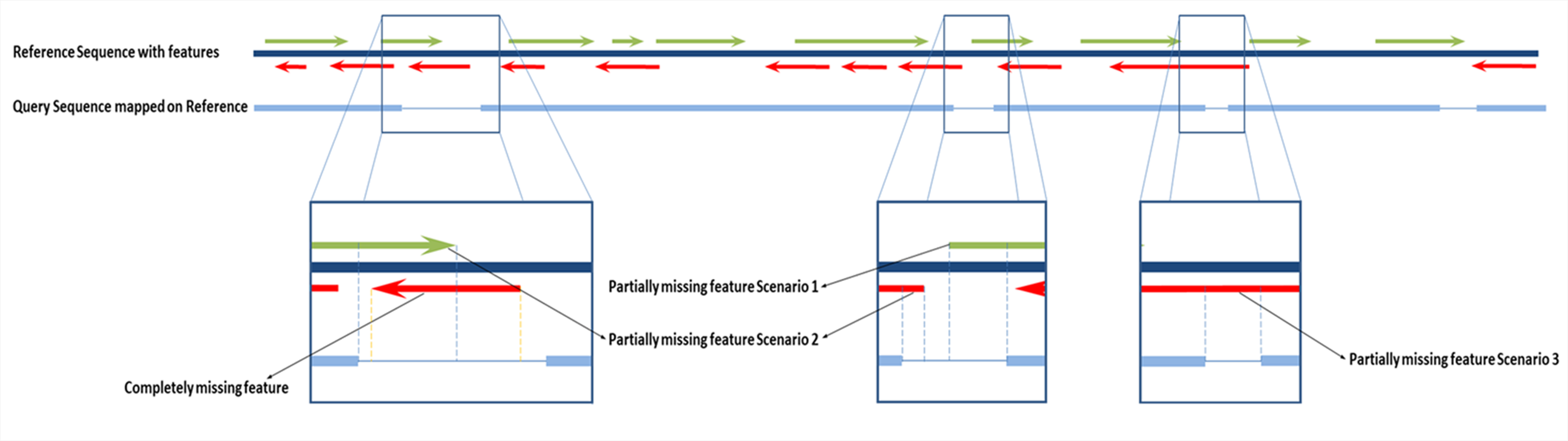

Supplement: Supplementary file 2 — Additional file 2. Depicting of scenarios for complete and partially missing coding sequences as implemented in MRF tool. Each arrow in the figure indicates a coding sequence (CDS). When a CDS coordinates fall within a gap’s position, it is identified as completely missing feature. Whereas when a CDS position doesn’t fall perfectly within a gap region, it means only a part of it ends up deleted (partially missing feature, scenario 1 to 3). This can happen in three different ways. In the first scenario, the gap region starts downstream to the coding sequence, extends into it and ends within the coding sequence. The second scenario is opposite to the first, where the gap region starts from within the coding sequence and extends beyond it. In the last scenario, the gap region starts and ends within the coding sequence. [file 12985_2023_2035_MOESM2_ESM.tif]

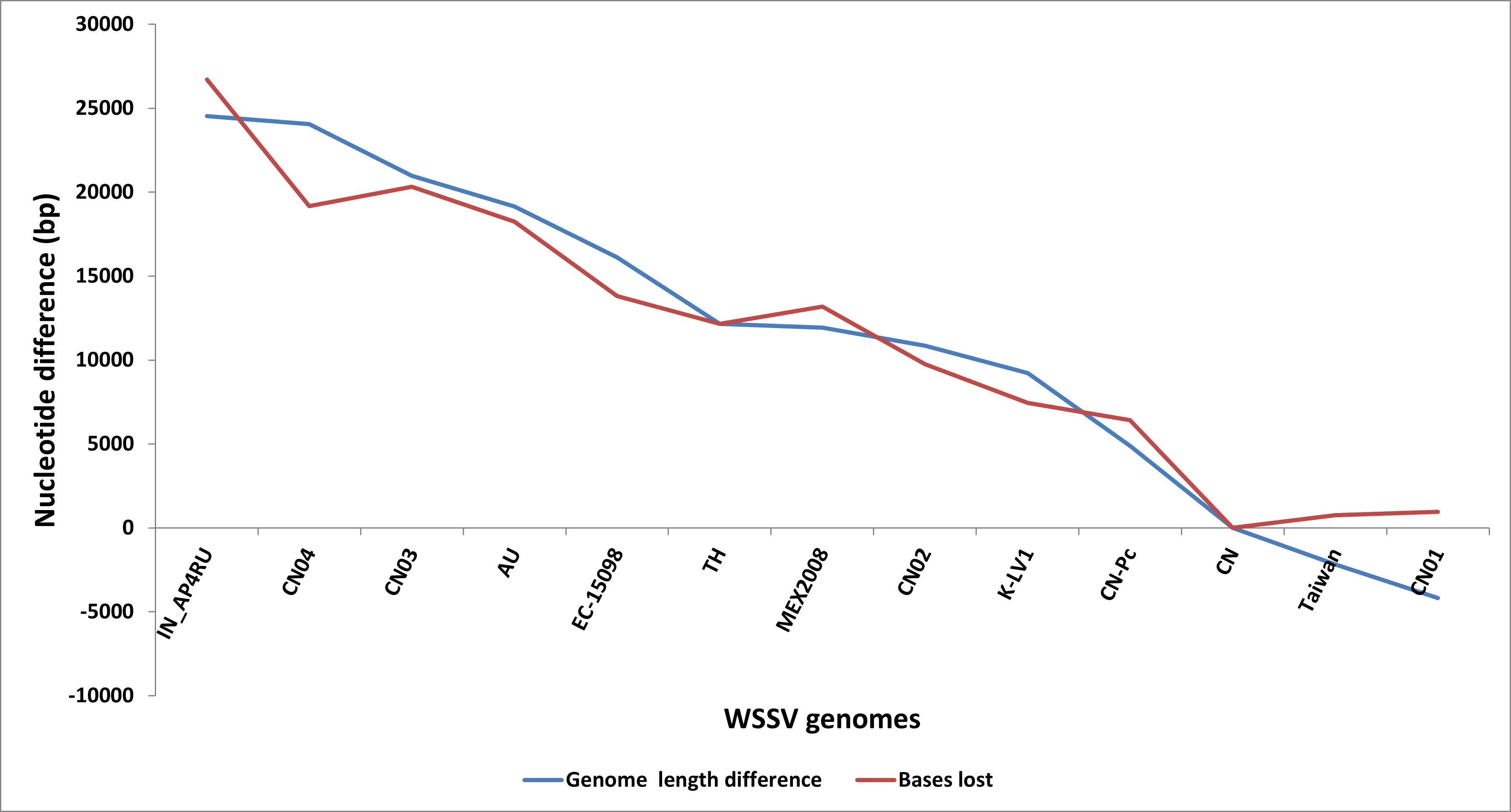

Supplement: Supplementary file 3 — Additional file 3. Comparison of genome length difference (blue line) and base length deleted (red line) in WSSV isolates with respect to CN isolate as identified by MRF tool. [file 12985_2023_2035_MOESM3_ESM.tif]

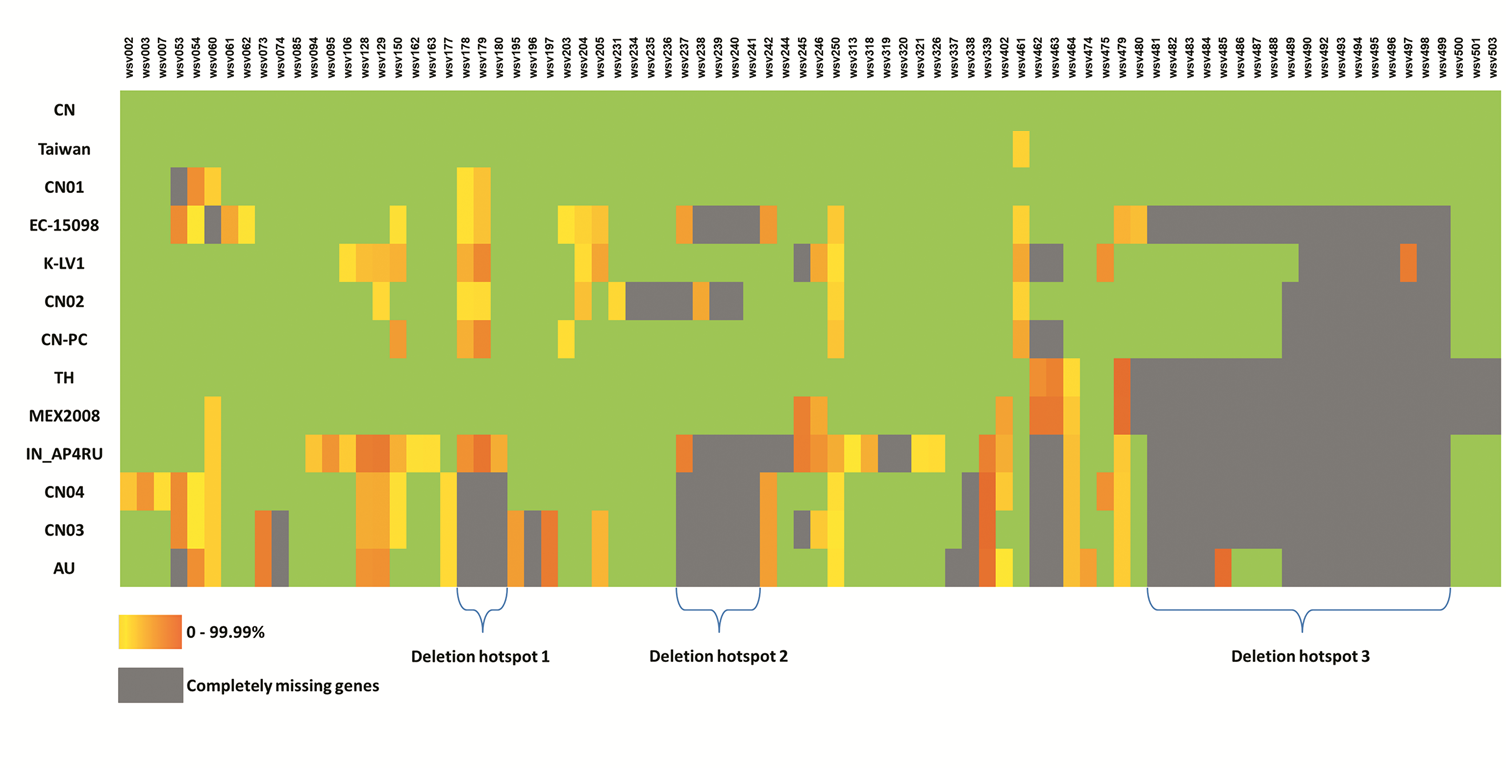

Supplement: Supplementary file 6 — Additional file 6. Heat map of the deleted CDS and deletion hot spots in WSSV genomes. Each row represents one WSSV isolate and each column represents one coding sequence. [file 12985_2023_2035_MOESM6_ESM.tif]

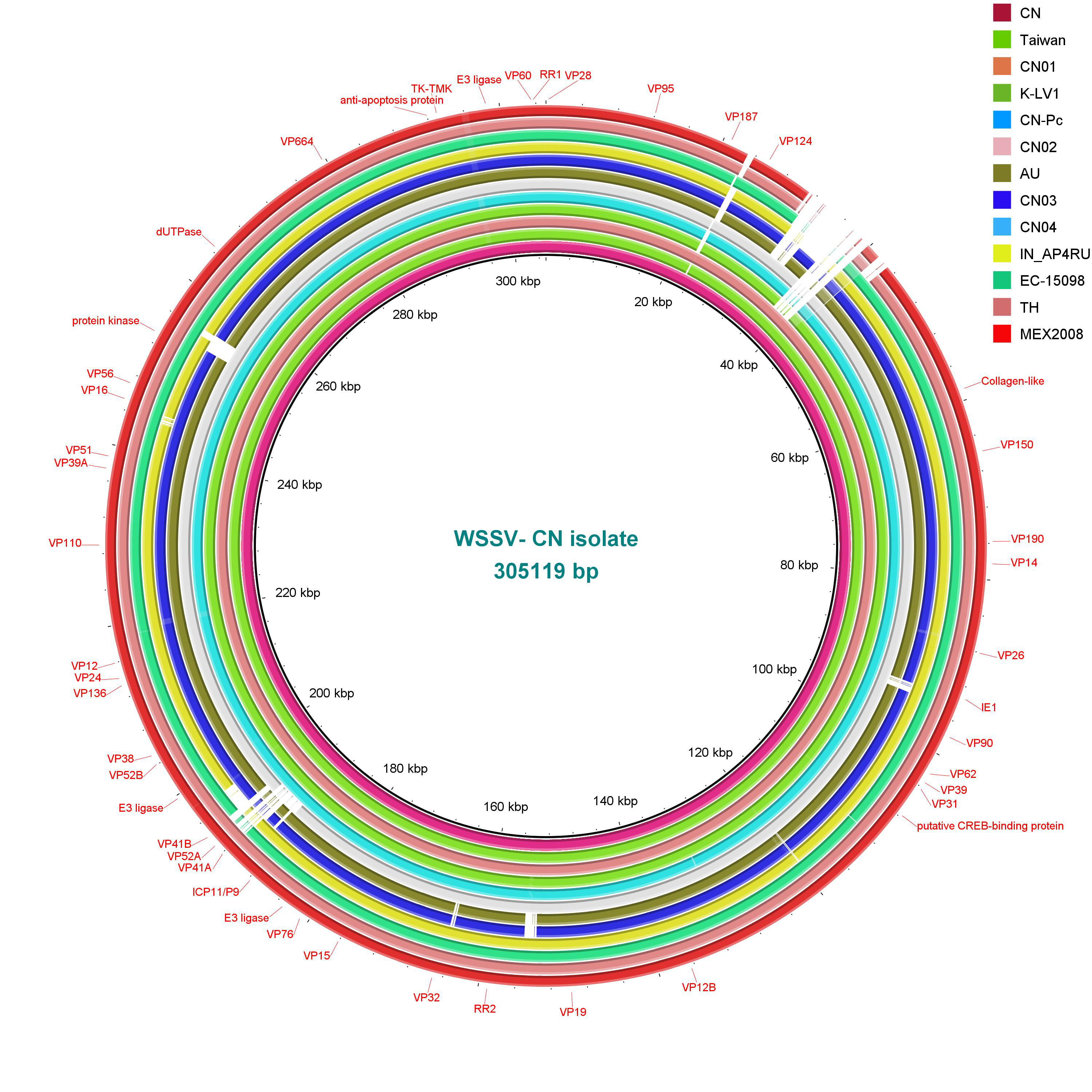

Supplement: Supplementary file 7 — Additional file 7. Briggs image depicting the deletion patterns of important coding sequences in various WSSV isolates. [file 12985_2023_2035_MOESM7_ESM.tif]

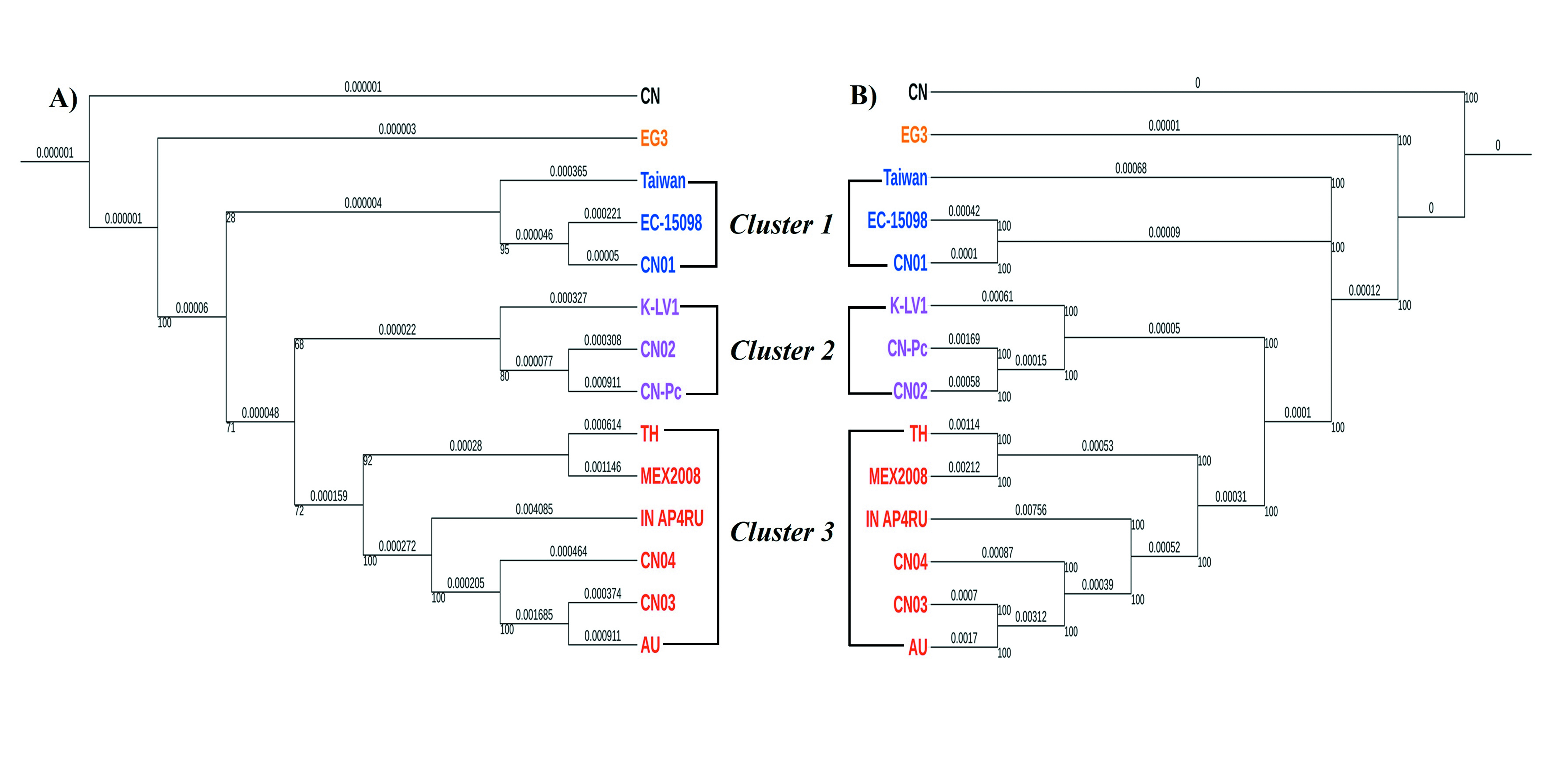

Supplement: Supplementary file 8 — Additional file 8. Maximum likelihood (A) and Bayes (B) trees built using complete WSSV genomes. The branch lengths are shown above and bootstrap/clade credibility values are shown below the branches. [file 12985_2023_2035_MOESM8_ESM.tif]

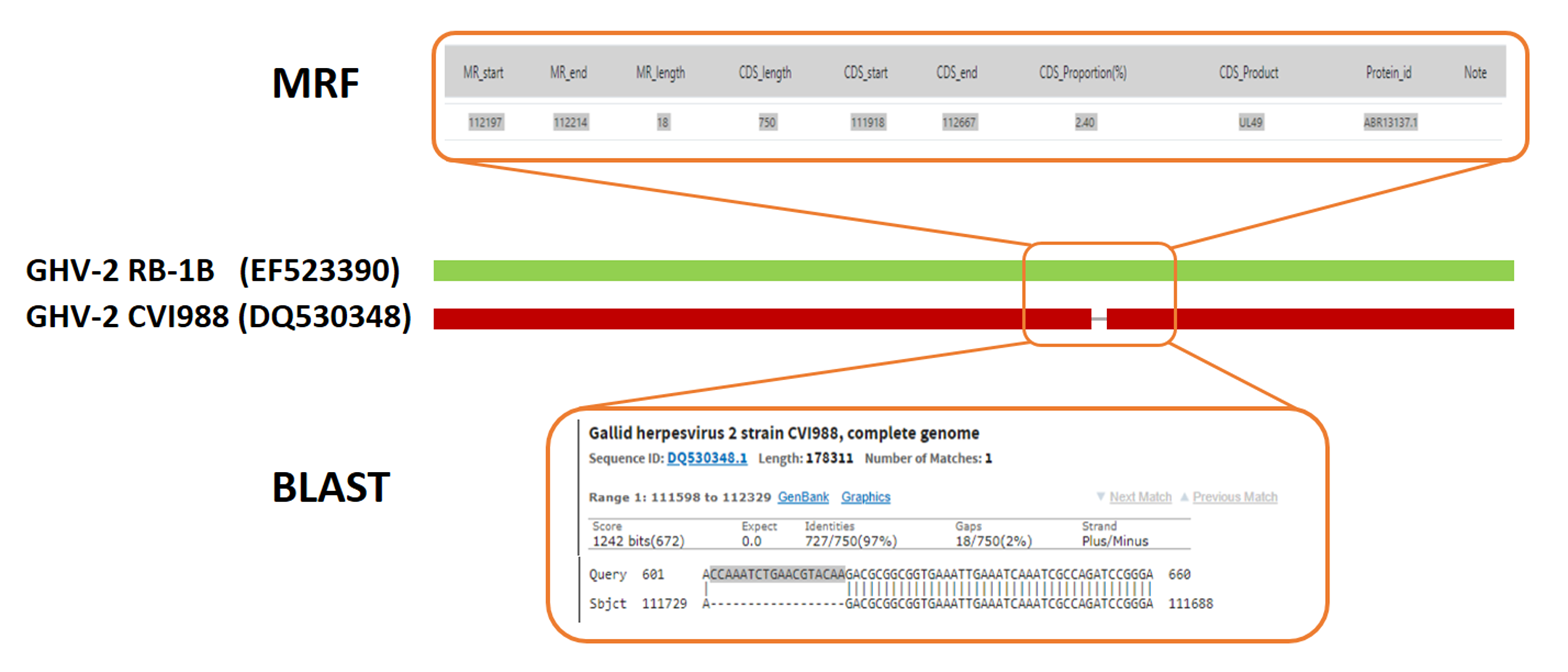

Supplement: Supplementary file 11 — Additional file 11. Utility of MRF in finding minor deletions of significance explained with a case of Marek’s disease virus. For MRF run, the query is DQ530348 genome [178311 bp; GHV-2 CVI988/Rispens vaccine strain] and the reference is EF523390 genome [178246 bp, GHV-2 RB-1B virulent strain]. For BLAST search, query is coding sequences of EF523390 genome and the subject is DQ530348 genome. The MRF is able to find a partial deletion of 18 bp (table given above) which is significant to this virus. The same deletion was observed in the alignment generated by BLAST as well (alignment given below). Here, MRF fared on par with the BLAST. [file 12985_2023_2035_MOESM11_ESM.tif]

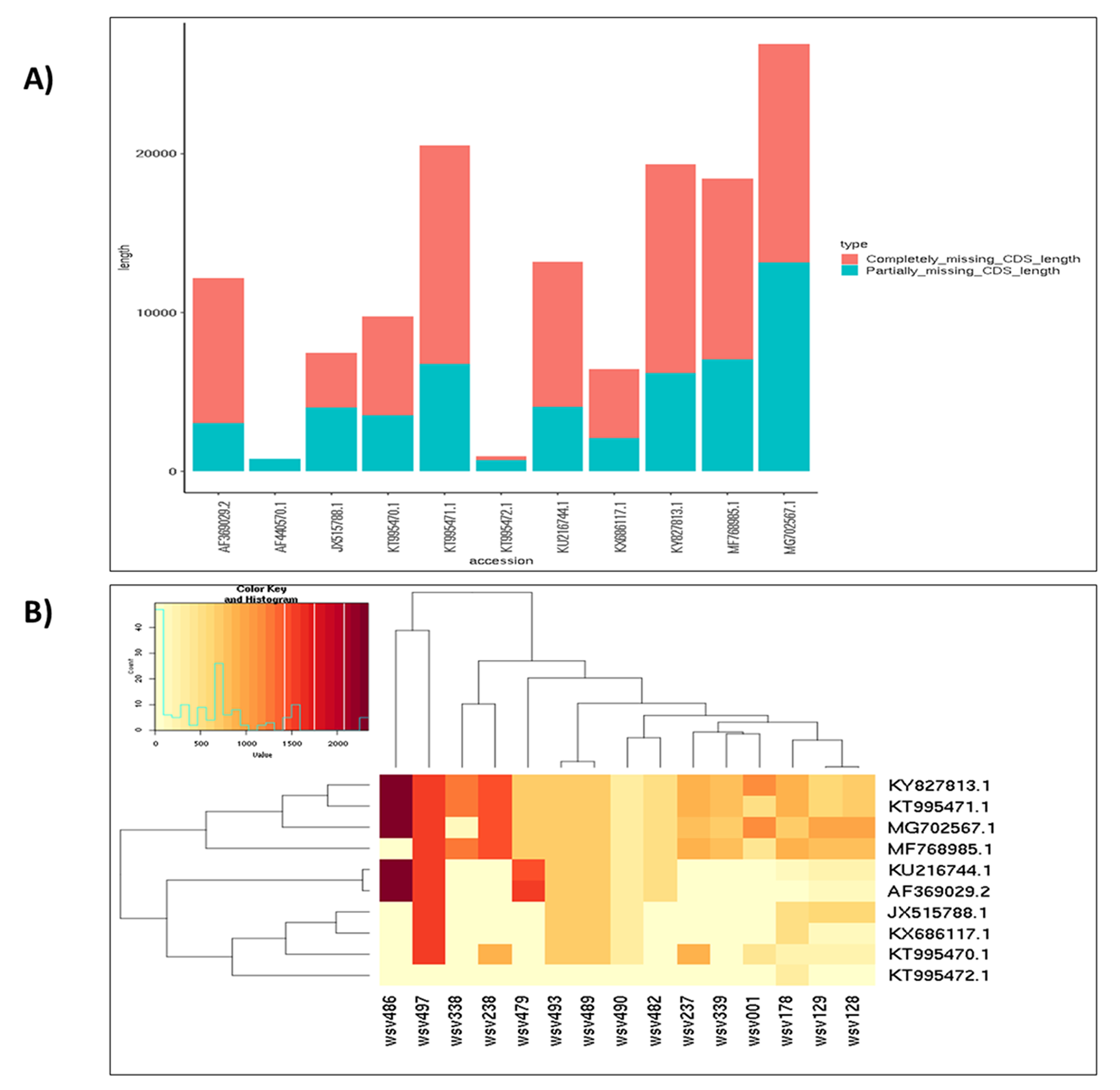

Supplement: Supplementary file 12 — Additional file 12. Output of MRF run in batch mode, WSSV, case with high number of coding sequences. Here 11 query genomes (AF369029, AF440570, JX515788, KT995470, KT995471, KT995472, KU216744, KX686117, KY827813, MF768985, MG702567) are compared to a reference genome (AF332093) in one go. A Bar plot of complete and partially missing CDS length (bases) in query genomes. B Heatmap showing the ten genomes that exhibited high missing genome lengths and the CDS contributing to missing genome lengths. The heatmap is accompanied by a clustering of genomes based on missing genome regions. [file 12985_2023_2035_MOESM12_ESM.tif]

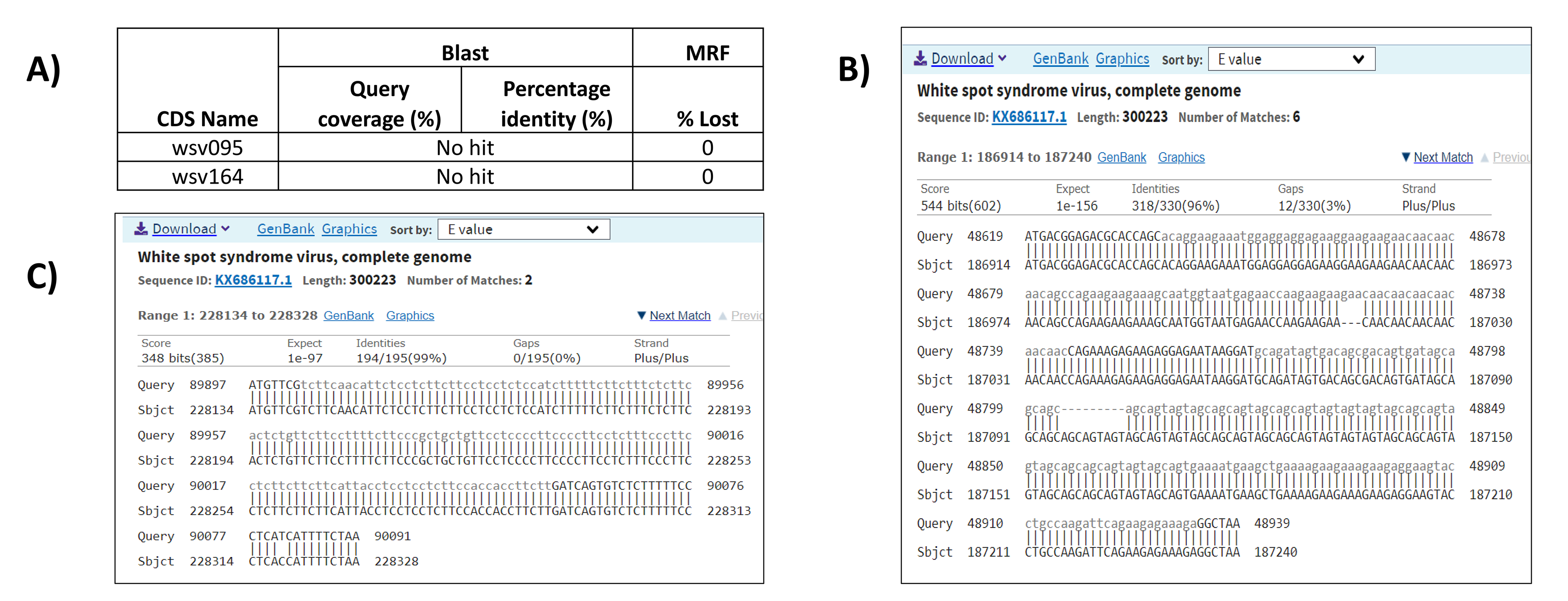

Supplement: Supplementary file 16 — Additional file 16. Illustration of MRF’s merit in reporting results for coding sequences having low complexity regions with WSSV case (highly-similar but length-varying genomes). For BLAST search, query is coding sequences of AF332093 genome and the subject is KX686117 genome. For MRF run, the query is KX686117 genome [300223 bp] and the reference is AF332093 genome [305119 bp]. A Summary of results from BLAST search and MRF run for 2 CDS, wsv094 and wsv164. Blast failed to report hits for wsv095 and wsv164 at default parameters (word length = 28). Whereas MRF prints nil deletion in query genome for these 2 coding sequences. B BLAST prints alignment for wsv094 when run with a reduced word length of 11. C BLAST prints alignment for wsv164 when run with a reduced word length of 11. As indicated in alignments, even though there were perfect matches that were greater than the length 28 (as shown in B and C), they were not considered as High Scoring Portions (HSPs) in default run as the alignments have low-complexity regions. [file 12985_2023_2035_MOESM16_ESM.tif]
