# Supplementary material for: MRF: a tool to overcome the barrier of inconsistent genome annotations and perform comparative genomics studies for the largest animal DNA virus
Source: Virol J. 2023 Apr 18;20:72. doi: 10.1186/s12985-023-02035-w (PMC10111743; doi:10.1186/s12985-023-02035-w)
Supplement: Supplementary file 14 — Additional file 14. List of coding sequences displaying complete deletion in certain isolates and which are harboring glycosylation sites. [file 12985_2023_2035_MOESM14_ESM.docx]

**Additional File 14**. This supplementary file contains the results of the search performed for glycosylation sites in the 14 proteins that exhibited complete deletion in medium-virulent and/or low-virulent clusters compared to high-virulent cluster. The prediction for glycosylation sites was performed at NetOGlyc3.1 Server (Julenius et al., 2005) and NetNGlyc1.0Server (Gupta et al., 2004) and the results were given in table below.

| **Cluster** | **Deleted**  **CDS** | **N-Glycosylation sites** | **O-Glycosylation sites** | **GO Terms** | **E Value** |
| --- | --- | --- | --- | --- | --- |
| coding sequences deleted in both cluster 2 (medium-virulent) and cluster 3 (low-virulent) | wsv489 | Yes | Yes | C:viral nucleocapsid | 8.17E-131 |
|  | wsv490 | Yes | Yes | Envelope glycoprotein gp2 | 41% |
|  | wsv492 | Yes | No | - |  |
|  | wsv493 | No | Yes | C:viral nucleocapsid | 2.59E-91 |
|  | wsv494 | No | Yes | C:viral nucleocapsid (similarity to vp35, partial) | 5.16E-10 |
|  | wsv495 | No | No | - |  |
|  | wsv496 | No | No | - |  |
|  | wsv498 | No | Yes | - |  |
|  | wsv499 | No | Yes | - |  |
| coding sequences deleted in only cluster 3 (low-virulent) | wsv481 | No | Yes | - |  |
|  | wsv482 | No | Yes | C:integral component of membrane | 4.32E-57 |
|  | wsv483 | No | No | - |  |
|  | wsv484 | Yes | Yes | C:integral component of membrane | 2.33E-119 |
|  | wsv487 | No | No | - |  |

**References:**

Gupta R, Jung E, Brunak S. Prediction of N-glycosylation Sites in Human Proteins. 2004. Available online: <http://www.cbs.dtu.dk/services/NetNGlyc/> (accessed on 19 November 2019).

Julenius K, Molgaard A, Gupta R, Brunak S. Preiction, conservation analysis and structural characterization of mammalian mucin-type O-glycosylation sites. Glycobiology, 2005;15: 153-164.

**Actual results on predicted N-Glycosylation sites:**

1. **wsv489**

**
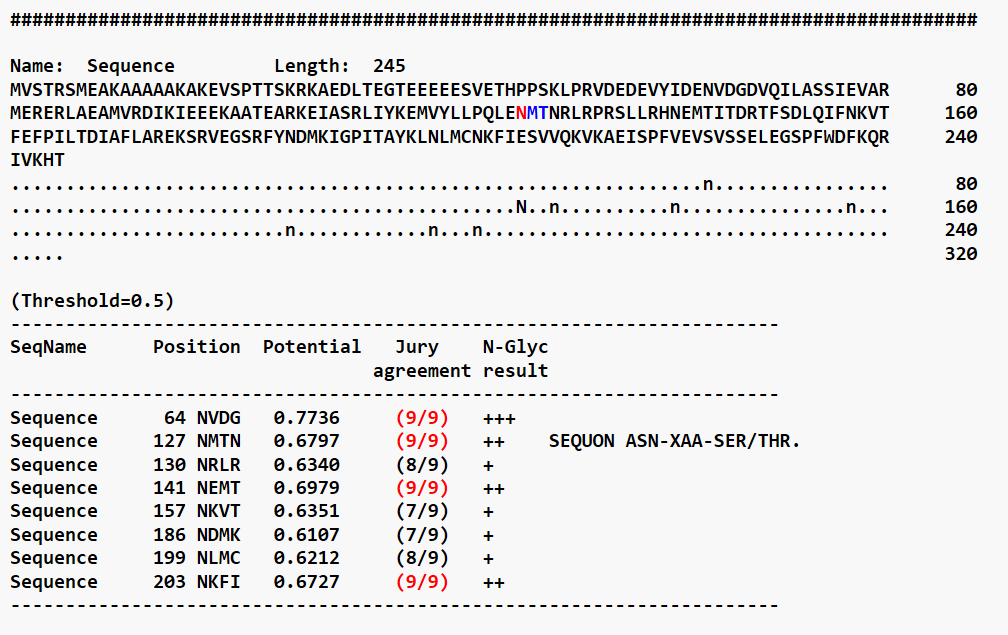
**

1. **wsv490**

**
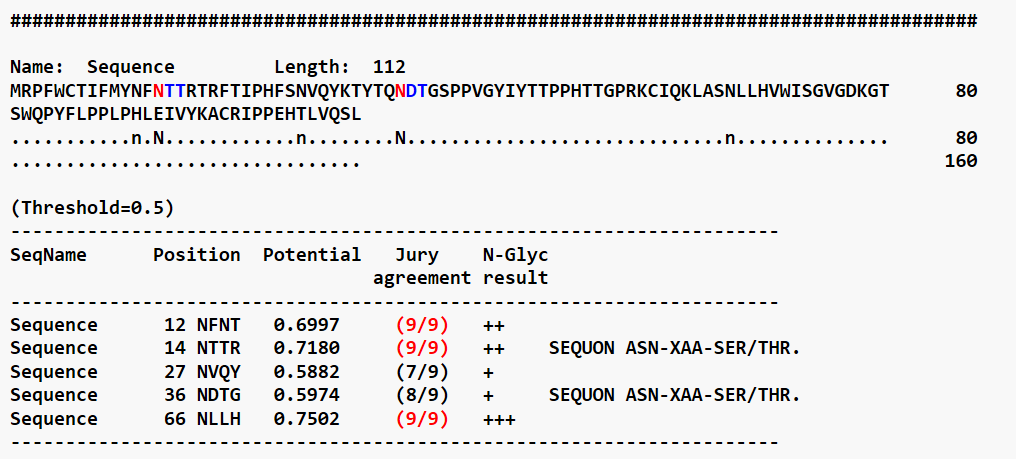
**

1. **wsv492**

**
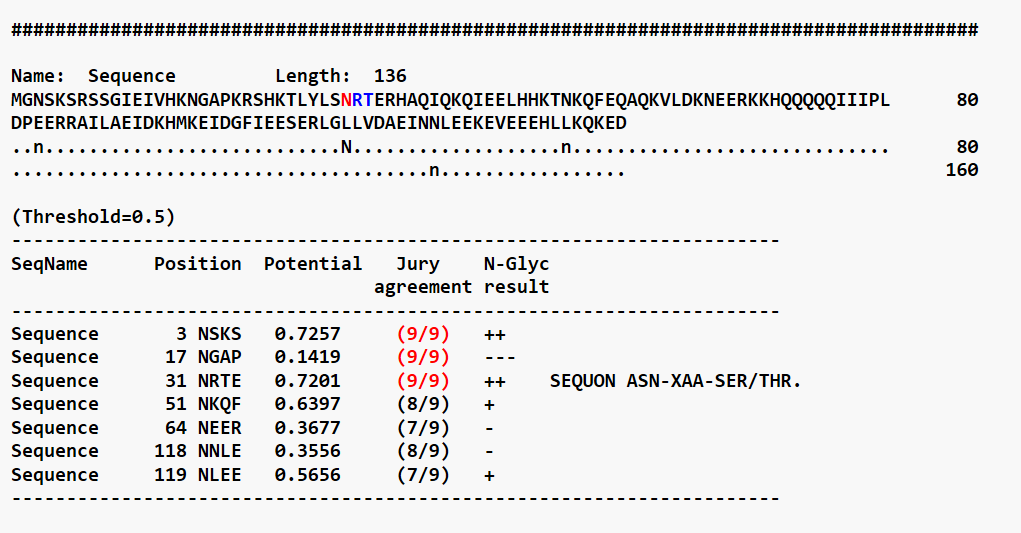
**

1. **wsv484**


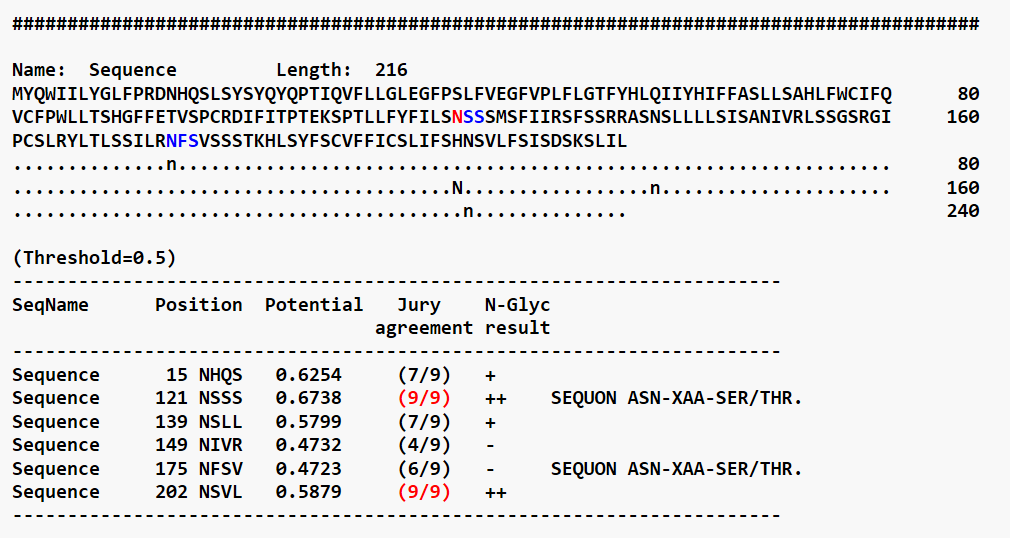


**Actual results on predicted O-Glycosylation sites:**

1. **wsv481**

**
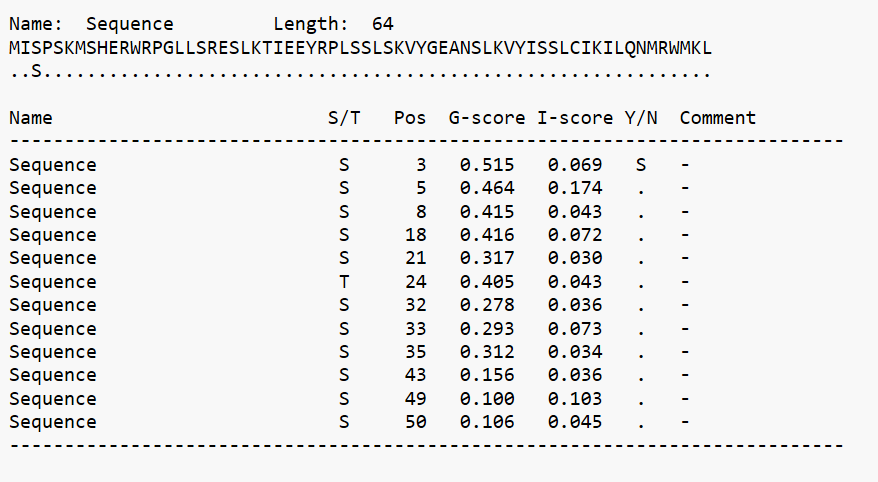
**

1. **wsv482**

**
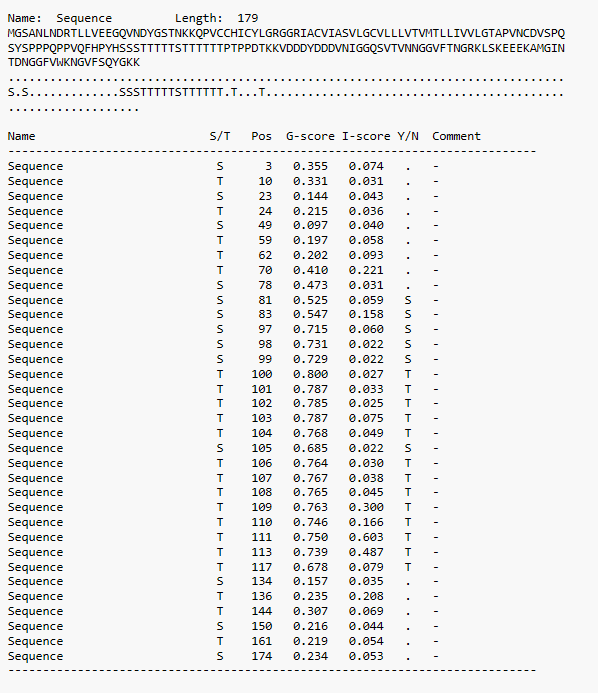
**

1. **wsv484**

**
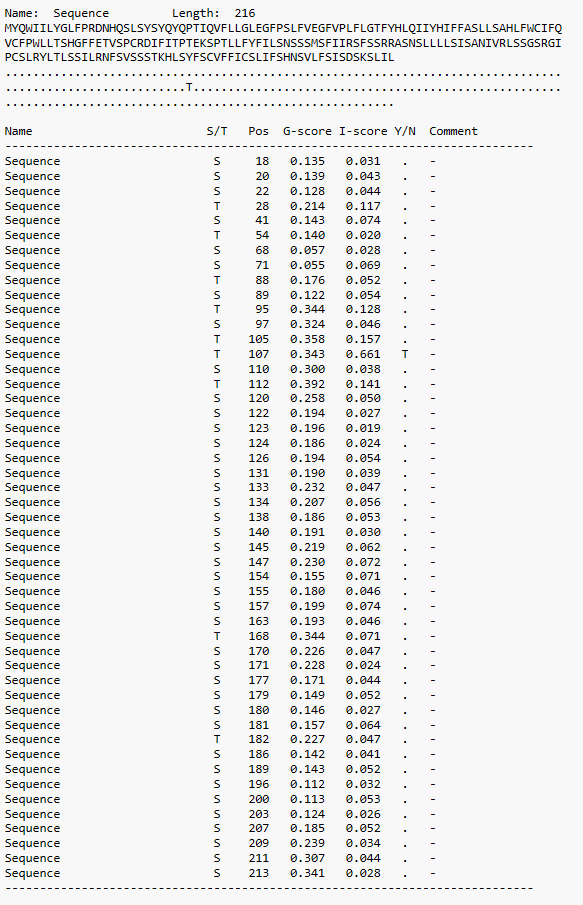
**

1. **wsv489**

**
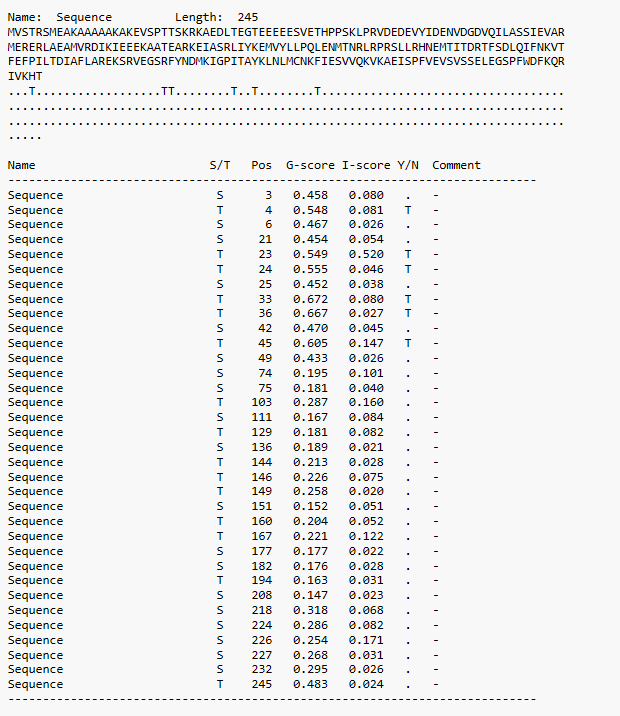
**

1. **wsv490**

**
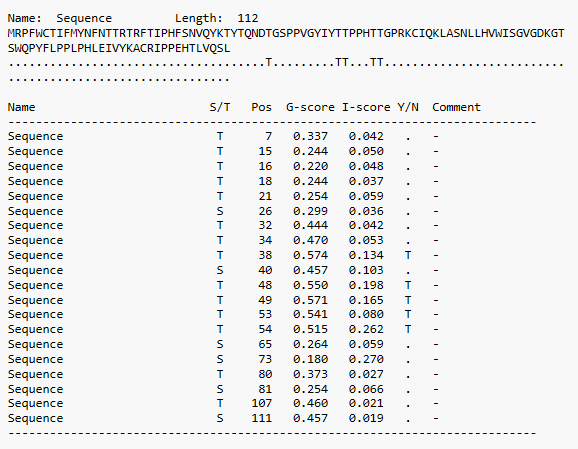
**

1. **wsv493**

**
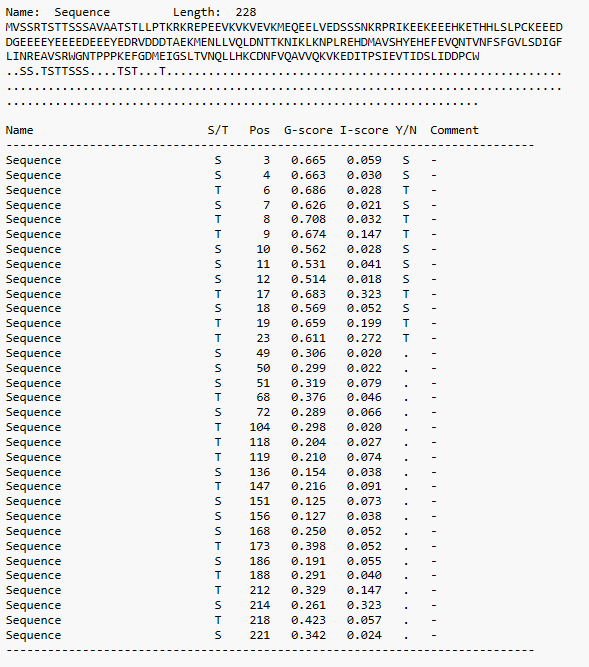
**

1. **wsv494**

**
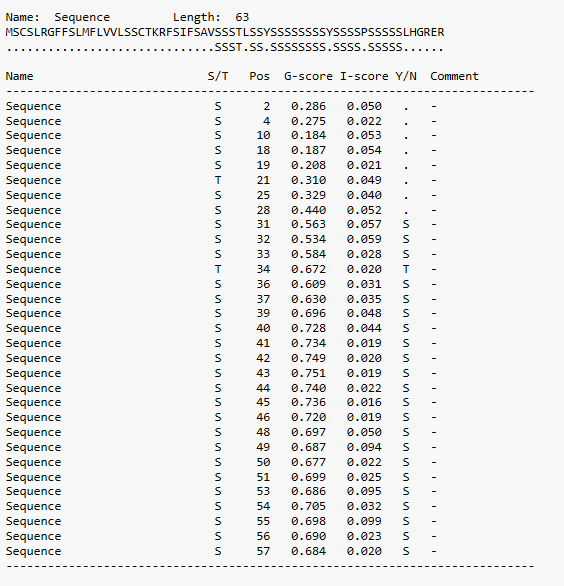
**

1. **wsv498**

**
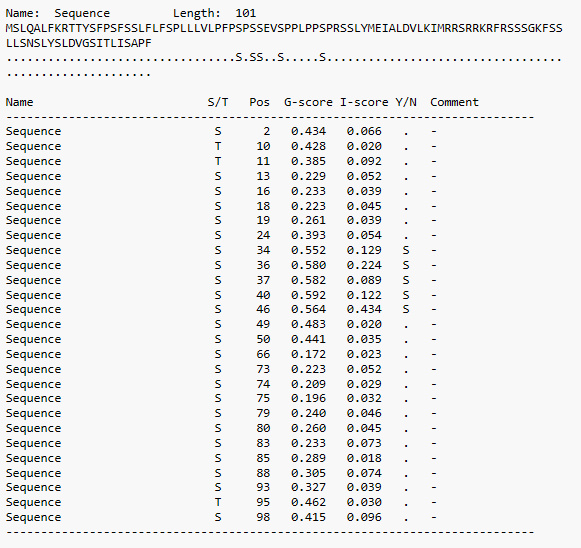
**

1. **wsv499**

**
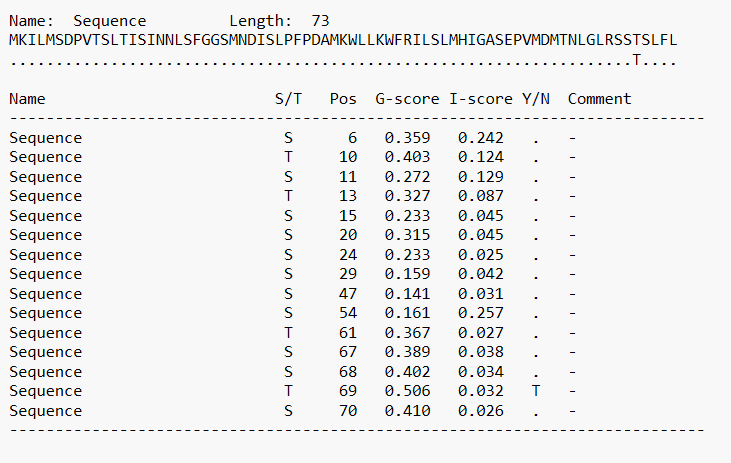
**
