# Supplementary material for: MRF: a tool to overcome the barrier of inconsistent genome annotations and perform comparative genomics studies for the largest animal DNA virus
Source: Virol J. 2023 Apr 18;20:72. doi: 10.1186/s12985-023-02035-w (PMC10111743; doi:10.1186/s12985-023-02035-w)
Supplement: Supplementary file 17 — Additional file 17. User manual developed for MRF tool. [file 12985_2023_2035_MOESM17_ESM.pdf]

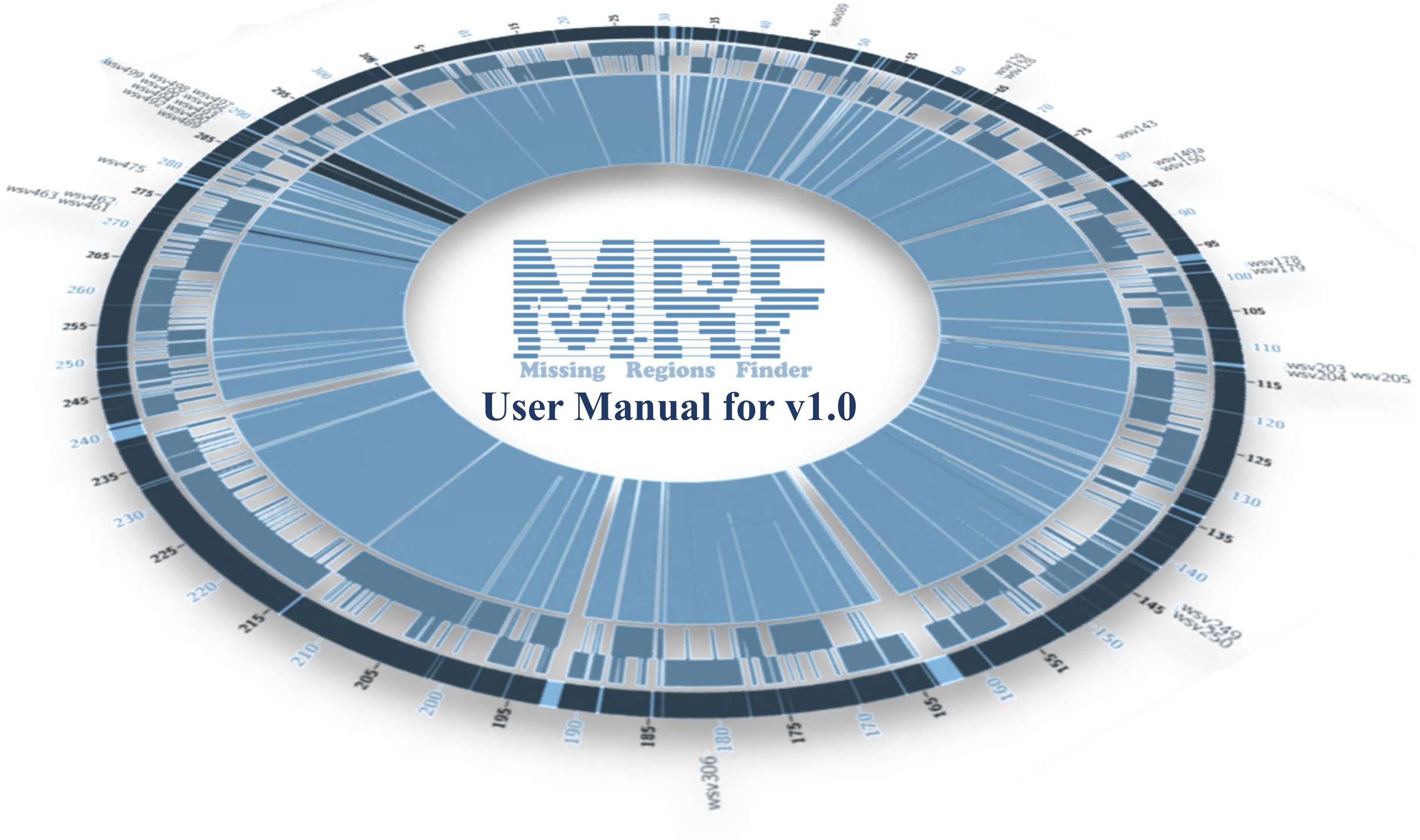

User Manual for v1.0

http://14.139.181.163/mrf

Table of Contents

1. What is MRF? ---------- **4**

2. Quick Introduction ---------- **5**

3. Input and Output description ---------- **9**

3.1 Inputs ---------- *9*

3.2 Outputs ---------- *10*

4. How MRF works ---------- **10**

5. Working with advanced options ---------- **12**

6. RNA viruses – Tweaking with False match length and offset window ---------- **14**

6.1 Exact match length – 20, False match length – 15, Offset window – 1,1 [Default] ---------- *15*

6.2 Exact match length – 10, False match length – 0, Offset window – 0,0 ---------- *16*

6.3 Mum length – 10, False match length – 15, Offset window – 1,1 ---------- *17*

6.4 Mum length – 5, False match length – 0, Offset window – 0,0 ---------- *19*

6.5 Mum length – 5, False match length – 15, Offset window – 1,1 ---------- *20*

6.6 Mum length – 5, False match length – 15, Offset window – 2,2 ---------- *20*

6.7 Mum length – 5, False match length – 15, Offset window – 3,3 ---------- *21*

6.8 Mum length – 5, False match length – 15, Offset window – 4,4 ---------- *22*

7. Filtering partially missing coding sequences ---------- **23**

8. MRF for comparing multiple genomes – Batch mode ---------- **25**

8.2 Running MRF-batch program ---------- *26*

8.3.1 Default run ---------- 27
8.3.2 Search by protein id (-I) ---------- 32
8.3.3 Search by protein names (-N) ---------- 33
8.3.4 Filter by missing CDS length (-Y) ---------- 34
8.3.5 List first n proteins (-F) ---------- 35
8.3.6 List last n proteins (-Z) ---------- 36
8.3.7 List proteins in range (-R) ---------- 37
8.3.8 List only top n affected genomes (-U) ---------- 38

8.4 Plot options: ---------- *39*

8.4.1 Use case: SARS-COV2 ---------- 39
8.4.2 Use case: ASFV ---------- 44

9. Conclusion ---------- **49**

APPENDIX – I (*Motivation for building MRF*) ---------- **50**

APPENDIX – II (*Benchmarking studies*) ---------- **55**

A2.1 USE CASE 1: White Spot Syndrome Virus (highly-similar but length-varying genomes) ---------- *55*

A2.1.1 Benchmarking MRF with Blast ---------- 55
A2.1.1.1 NCBI Blast ---------- 55

A2.1.1.2 MRF ---------- 56
A2.1.1.3 Comparison between Blast and MRF results ---------- 59
A2.1.2 Case study:Identifying deletion hotspots in isolates of WSSV with MRF ---------- 66
A2.1.2.1 Deletions in WSSV genomes ---------- 67
A2.1.2.2 Deletion hotspots ---------- 68

A2.2 USE CASE 2: African swine fever virus (ASFV) ---------- *71*
A2.2.1 Benchmarking MRF with Blast ---------- 71
A2.2.1.1 NCBI BLAST ---------- 71
A2.2.1.2 MRF ---------- 72
A2.2.1.3 Consolidated results from Blast and MRF ---------- 74
A2.2.2 Case study: Analyze multiple strains of AFSV with MRF and identify deletion hotspots ---------- 79

A2.3 USE CASE 3: HIV-1 genomes ---------- *82*
A2.3.1 Benchmarking Blast with MRF ---------- 82
A2.3.1.1 MRF ---------- 82
A2.3.1.2 NCBI Blast ---------- 85
A2.3.1.3 Comparison between blast and MRF output ---------- 85
A2.3.1.4 MRF run with reduced exact match length (10): ---------- 86
A2.3.1.5 NCBI blast search with reduced word length (11) ---------- 88
A2.3.1.6 Comparison between Blast and MRF output ---------- 88
A2.3.1.7 Inference ---------- 89
A2.3.2 Case Study: Tracking the *nef* gene in HIV-1 strains ---------- 89

A2.4 USE CASE 4: SARS-COV2 genomes ---------- *91*
A2.4.1 MRF ---------- 91
A2.4.2 NCBI blast: batch ---------- 92
A2.4.3 Case study: Identification of Single Nucleotide Polymorphisms (SNP's) ---------- 93

A2.5 USE CASE 5: Marek's disease virus (Gallid herpesvirus 2) ---------- *95*
A2.5.1 MRF ---------- 95
A2.5.2 Blast ---------- 96

A2.6 SUMMARY ---------- *97*

1. What is MRF?

MRF is a virus comparative genomics tool that compares two genomes and generates completely and partially missing coding sequences (CDS) in one genome (query) with respect to the other (reference) and presents them in a MirCos plot in addition to tabular output.

Genome-wide similarity-search tools like *blast* often end up reporting similar genomic regions between genomes. These tools cannot indicate genome-wide missing regions in a genome in comparison to another. The genome-wide similarity becomes irrelevant while comparing virus genomes that are highly similar but vary in genome length. Here, documentation of missing CDS in an isolate against a reference would be helpful to understand better about a virus isolate. Moreover, obtaining this information in one annotation nomenclature becomes mandatory when inconsistencies exist in annotations between genomes. In addition, comparative genomics studies involving pathogenic viruses need to interpret on missing CDS in one isolate/strain when compared to another. Therefore, a tool capable of documenting missing CDS has great potential to complement existing alignment-based similarity-search tools in virus research.

Software and availability

The MRF is available for download at https://github.com/vinayciba/MRF. Alternatively, users can access MRF as an online tool in any web browser at http://14.139.181.163/mrf.

Uses

- Main use of MRF is to know the complete and partial deletions in coding sequences of a query virus genome against a reference.
- MRF is best suited for comparing closely related virus species, strains of a species or isolates of a strain. However, it can still be useful in distantly related species to counter intuitively check for the presence of a particular coding sequence.
- With MRF, one can track complete or partial deletion of one or more genes in a virus genome in comparison to another.
- MRF is very useful in comparing genomes having inconsistent annotation nomenclature as it gives output in the annotation nomenclature of reference.
- One can quickly explore a newly sequenced virus genome for deletion of genomic regions harboring binding sites for diagnostic primers.
- One can quickly compare virus genomes and interpret for deletions in virulence-related or attenuation-related gene or gene families.

Key Features

- MRF can handle virus genomes of any size. The tool has been tested with the largest known virus, *Pandoravirus salinus* (2,473,870 bp; NC_022098).

- MRF takes the input files and instantly produces multiple outputs in real time.
- MRF prints genome coordinates for missing genomic regions as per the base positions of both reference and query genomes.
- The missing coding sequences output is given in both tabular and graphic form.
- Users can interactively filter out partially deleted coding sequences based on proportion of deleted sequence length.

2. Quick Introduction

1. The Quickest way to know how MRF works is by running the demo example facilitated in the MRF website through the Run demo button as shown in the following screenshots.

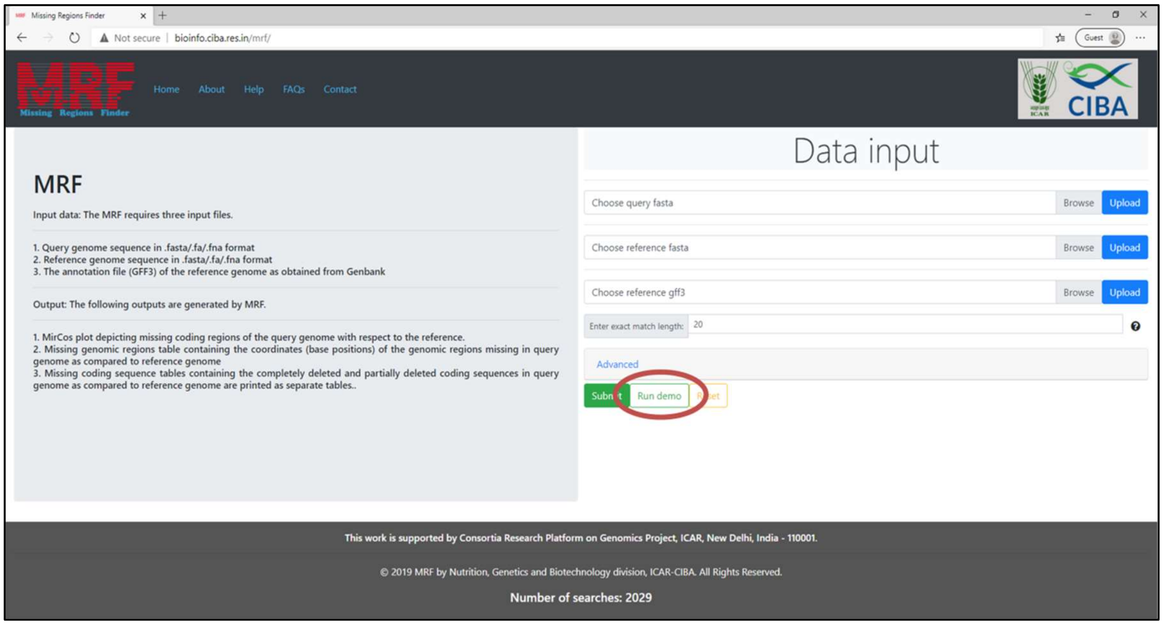

2. One click of Run demo automatically loads example datasets. Successful loading of example datasets is indicated by a message at the bottom of each of the three files. Otherwise, you may upload any other files of your interest. After files are uploaded, user would be prompted to click the Submit button.

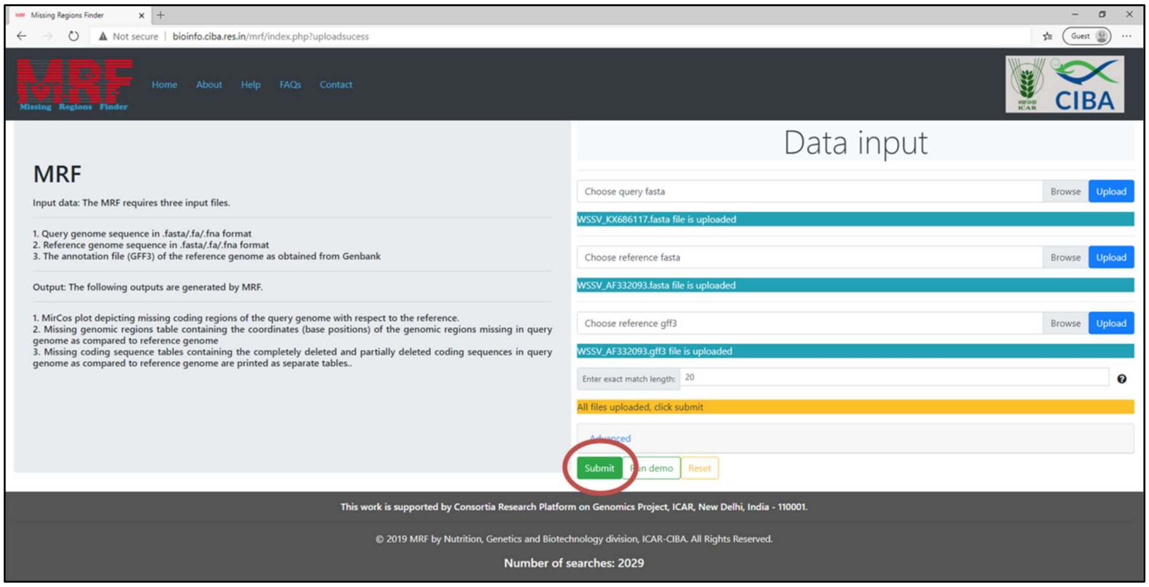

The files used for the demo run belong to the largest animal virus, White Spot Syndrome Virus (WSSV). The query fasta file (Accession no: KX686117) has a genome size of 300,223 bp, whereas the reference fasta file (Accession no: AF332093) has a genome size of 305,119 bp. The third file is annotation feature file for the same reference genome, in GFF3 format.

3. Once the Submit button is clicked, MRF processes the input files and generates output. The user is instantly taken to the results page. The results page is split into two sections, the right-hand section contains the list of completely and partially missing coding sequences in query genome displayed in two separate tables. The left-hand section is a *Dashboard* with few controls and summary of the results at the bottom.

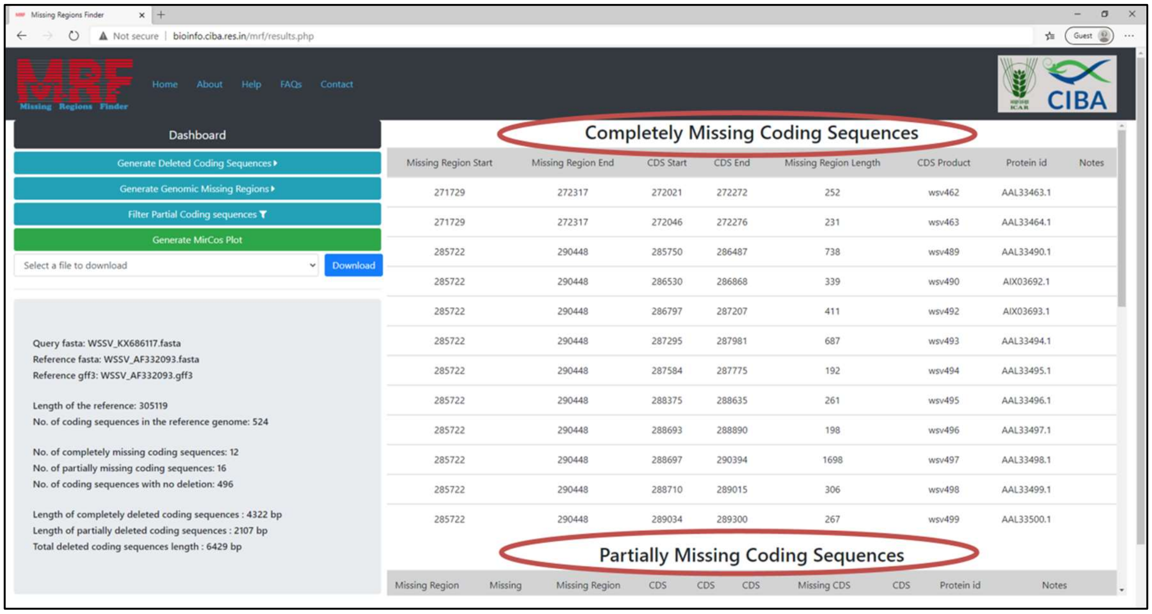

4. The missing coding sequences can be viewed as a circular visualization by clicking on ‘Generate MirCos Plot’ button. Clicking the button will prompt you to enter the title for the plot, clicking ok will reveal a textbox to enter the user preferred title for the plot. The MirCos plot has controls to zoom-in and zoom-out a particular area.

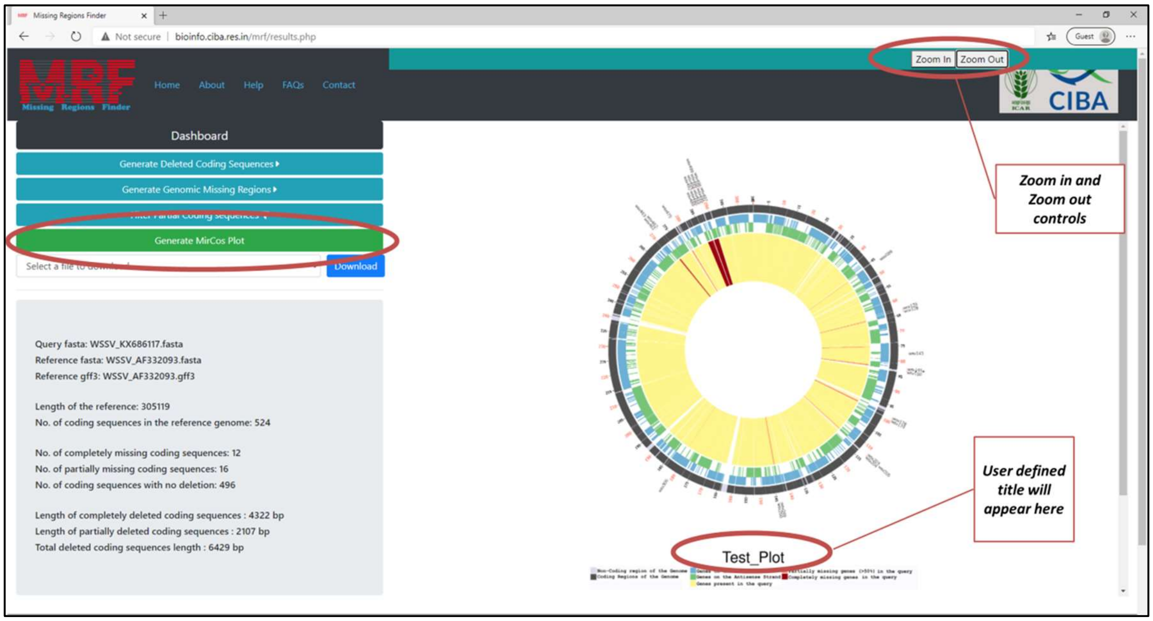

5. To further understand how the missing coding sequences are obtained, click on ‘Generate Genomic Missing Regions’ button.

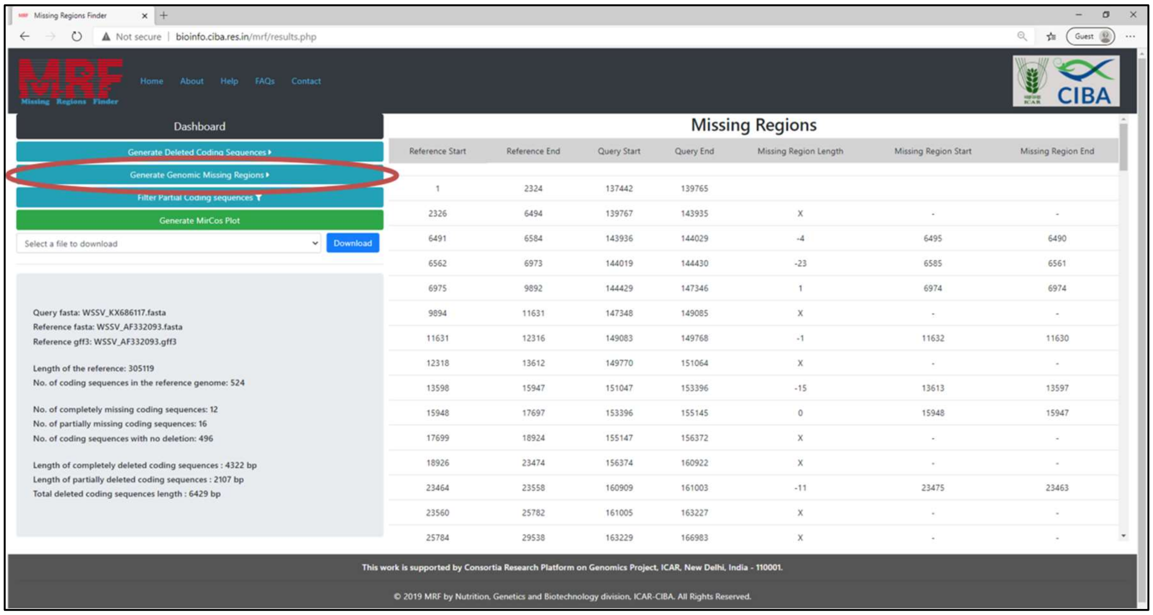

The first four columns give us the co-ordinates of perfect matches between reference genome and query genome. The 5th column gives us the length of non-hit region. The 6th

and 7th columns give us the co-ordinates of the non-hit region with respect to the reference genome.

The 5th column (non-hit region) gives three categories of values as explained in the following Figure 1.

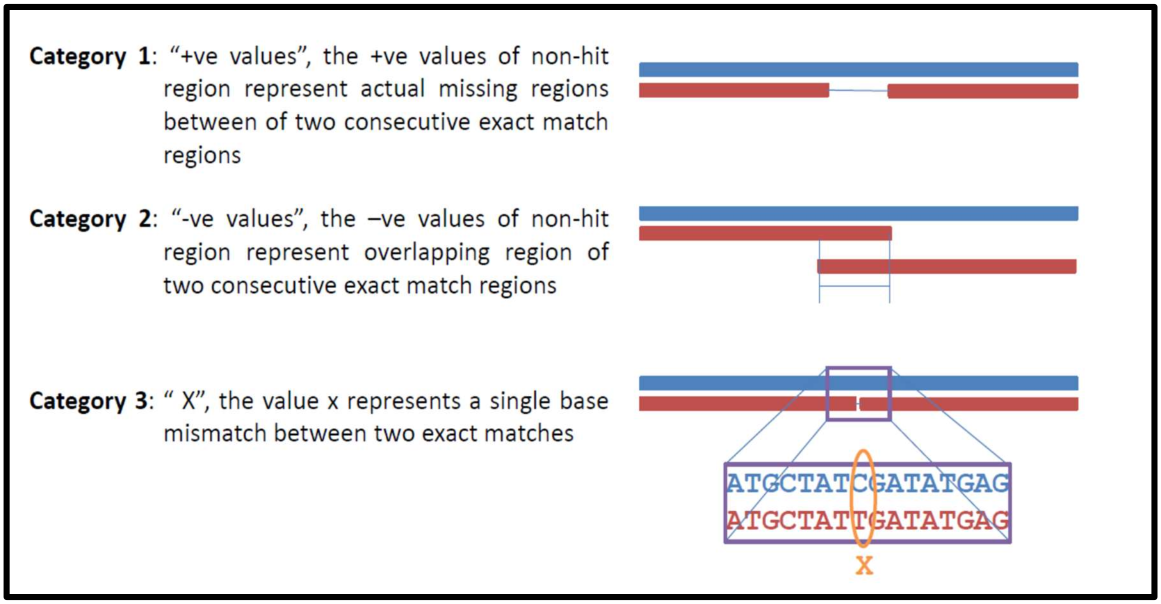

Figure 1: A pictorial representation of the three categories of missing region values offered by the tool.

3. Input and Output description

3.1 Inputs

1. **query fasta:** Nucleotide fasta[#] file of the query genome in .fa, .fna or .fasta format. Files with more than one chromosome or scaffold or contig are currently not handled.
2. **reference fasta:** Nucleotide fasta file of the reference genome in .fa, .fna or .fasta format. Files with more than one chromosome or scaffold or contig are currently not handled.

3. **reference gff3:** Annotation file of the reference genome as obtained from genbank in .gff3 format.

4. **exact match length (or mum length / -m):** The minimum length of an exact match that mummer will find between query and reference genomes being compared. All the resulting matches are greater than or equal to the length being set. [Default: 20]. The terms 'exact match length' and 'mum length' will be used interchangeably though out this document.

   The default *exact match length* of 20 works best for most genomes. However for genomes with high mutational rates such as RNA viruses, reducing the *exact match length* will result in more matches which in turn prevent false reporting of missing coding sequences.

5. **False match length cut off value (-l):** False exact matches tend to occur at random locations with shorter *mum length* leading to masking of true missing regions. To safe guard against finding false matches, MRF is equipped with two parameters, false match length and offset parameters. False match length is the length cut off, where matches less than or equal to the set value [default: 15] are screened further using the offset window options to determine if they are true matches or not.

6. **Negative offset and positive offset (-n & -p):** These two parameters in advanced options determine whether the matches screened using 'false match length cut off value' parameter are truly a false match. It helps user to rule out false matches especially while running MRF with shorter *mum length*. When these options are defined, then for each exact match, MRF checks the specified number of exact matches upstream (negative offset) and downstream (positive offset) to the current exact match for the base coordinates representing sequence contiguity of the query genome. If base coordinates are not contiguous then the current exact match would be treated as false match.

At the default setting [1,1], a match in query genome is examined whether it is contiguous by looking at one upstream and one downstream match. Any match that doesn't fall in between the upstream and downstream matches is deemed to be a false match.

Query fasta file need not necessarily contain full genome sequence. It can contain a single sequence representing partial genome to check for the presence or deletion of that sequence in the reference genome.

3.2 Outputs

1. **Missing coding sequences:** The MRF prints two tables listing the completely missing coding sequences and partially missing coding sequences (along with base position coordinates) in the query in comparison to reference genome. These tables can be downloaded using *Download* feature in *Dashboard*.

2. **Missing genomic regions:** The MRF also prints the details of exact matches; missing genomic regions and point mutations located between two exact matches, if any. This table can be downloaded using *Download* feature in *Dashboard*.

3. **Completely present coding sequences:** This output can only be downloaded to a file but cannot be visualized in results page. This file consists of the list of completely present coding sequences in the query with respect to the reference.

4. **MirCos plot:** It is a graphical plot representing the reference genome (outer ring); the genes present on sense strand of reference genome (second ring from outside); the genes present on anti-sense strand of reference genome (third ring from outside); completely missing coding sequences (red), partially missing coding sequences (orange), and completely present coding sequences (yellow) of query genome (inner ring). The MirCos plot is available for download with and without the legend.

4. How MRF works

MRF relies on the exact matches generated by MUMmer (mummer -mum -l 20 Reference.fasta Query.fasta) to extract the successive gaps between those matches. MUMmer generates a three column output which is read as reference_match_start_position, query_match_start_position and exact_match_length or as described in the MUMmer 3 manual "*For each match, the three columns list the position in the reference sequence, the position in the query sequence, and the length of the match respectively*". To know more about how mummer works, please visit the MUMmer home page. The head of the example ouput is shown below.

```
> KX686117.1
  1         137442      2324
  2326      139767      4169
  6491      143936      94
  6562      144019      412
  6975      144429      2918
  9894      147348      1738
  11631     149083      686
  12318     149770      1295
  13598     151047      2350
```

The above output is utilized to generate the end positions of the query and the reference and there after the coordinates of the missing regions. A screenshot of which is shown below.

Missing Regions

Reference Start	Reference End	Query Start	Query End	Missing Region Length	Missing Region Start	Missing Region End
1	2324	137442	139765			
2326	6494	139767	143935	X	-	-
6491	6584	143936	144029	-4	6495	6490
6562	6973	144019	144430	-23	6585	6561
6975	9892	144429	147346	1	6974	6974
9894	11631	147348	149085	X	-	-
11631	12316	149083	149768	-1	11632	11630
12318	13612	149770	151064	X	-	-
13598	15947	151047	153396	-15	13613	13597
15948	17697	153396	155145	0	15948	15947
17699	18924	155147	156372	X	-	-
18926	23474	156374	160922	X	-	-
23464	23558	160909	161003	-11	23475	23463
23560	25782	161005	163227	X	-	-
25784	29538	163229	166983	X	-	-

When we scroll down through the 'genomic missing regions' table, large deletions in query genome were observed (589 bp, 421 bp, 4727 bp).

272318	275501	109366	112549	589	271729	272317
275503	276428	112551	113476	X	-	-
276430	278004	113478	115052	X	-	-
277950	278156	115324	115530	-55	278005	277949
278578	285721	115627	122770	421	278157	278577
290449	293456	122768	125775	4727	285722	290448

All the ‘missing genomic regions’ of length greater than 1 bp are further compared with the annotation file (.gff3) to fetch the coding sequences present in these missing regions of query genome.

5. Working with advanced options

The default exact match length set in MRF is 20, therefore each match is 20 bp or longer when run with this setting. At this point, the default value of false match length of 15 and its corresponding offset window does not affect the output as the exact match length is greater than the false match length. For the below example, the default setting yields sufficiently large matches with no false matches.

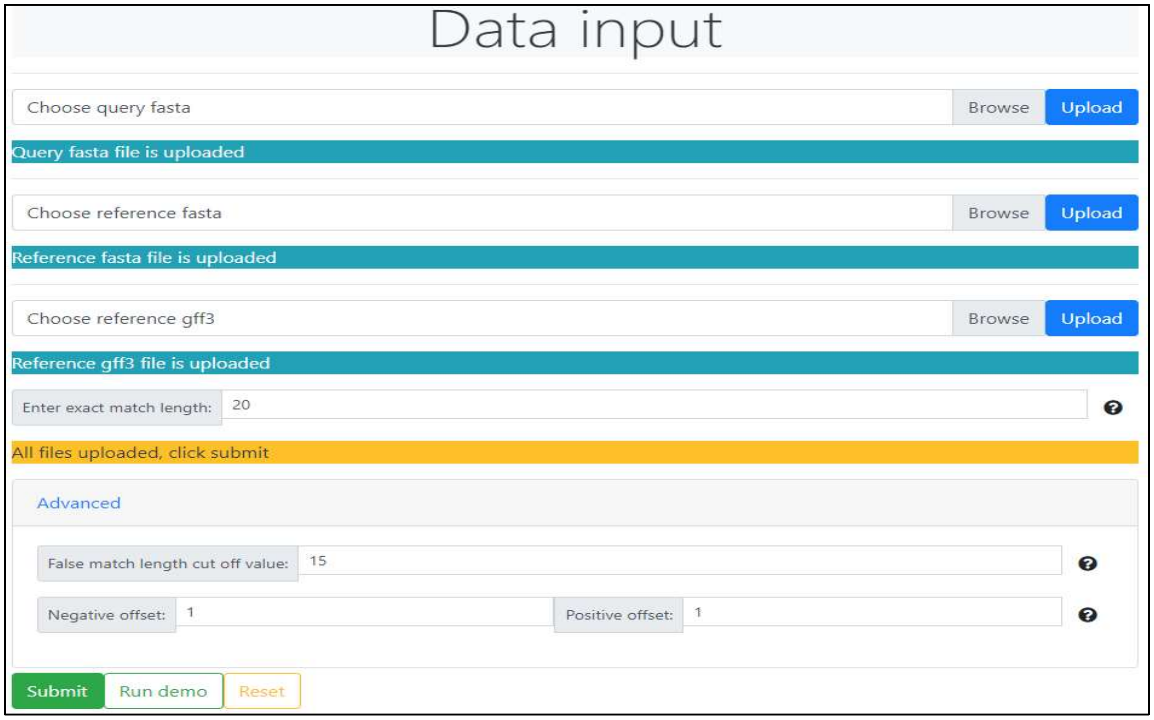

Missing Regions

Reference Start	Reference End	Query Start	Query End	Missing Region Length	Missing Region Start	Missing Region End
1	2324	137442	139765			
2326	6494	139767	143935	X	-	-
6491	6584	143936	144029	-4	6495	6490
6562	6973	144019	144430	-23	6585	6561
6975	9892	144429	147346	1	6974	6974
9894	11631	147348	149085	X	-	-
11631	12316	149083	149768	-1	11632	11630
12318	13612	149770	151064	X	-	-
13598	15947	151047	153396	-15	13613	13597
15948	17697	153396	155145	0	15948	15947
17699	18924	155147	156372	X	-	-

However when the exact match length is reduced to 5 and offset window is set to 0, many false matches appear which will affect the output.

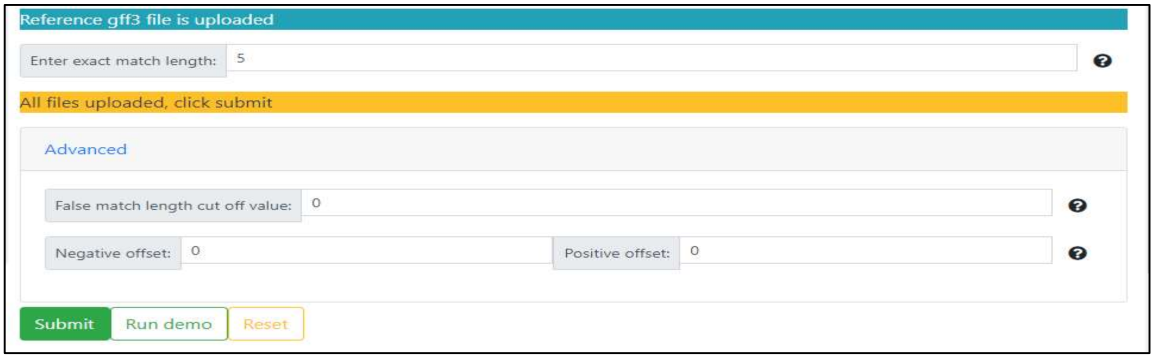

13598	15947	151047	153396	-15	13613	13597
15948	17697	153396	155145	0	15948	15947
17699	18924	155147	156372	X	-	-
18926	23474	156374	160922	X	-	-
23464	23558	160909	161003	-11	23475	23463
23560	25782	161005	163227	X	-	-
25784	29538	163229	166983	X	-	-
29540	30577	167286	168323	1	29539	29539
30568	30578	128561	128571	-10	30578	30567
30569	30580	239924	239935	-10	30579	30568
30573	30584	168316	168327	-8	30581	30572
30582	31048	168324	168790	-3	30585	30581
30754	32449	168725	170420	-295	31049	30753
32385	32726	170669	171010	-65	32450	32384
32728	37498	171012	175782	X	-	-
37500	37593	175784	175877	X	-	-

It can be seen that there are two false matches resulted from low mum length. However, when False match length and offset window parameters are set appropriately, they are eliminated.

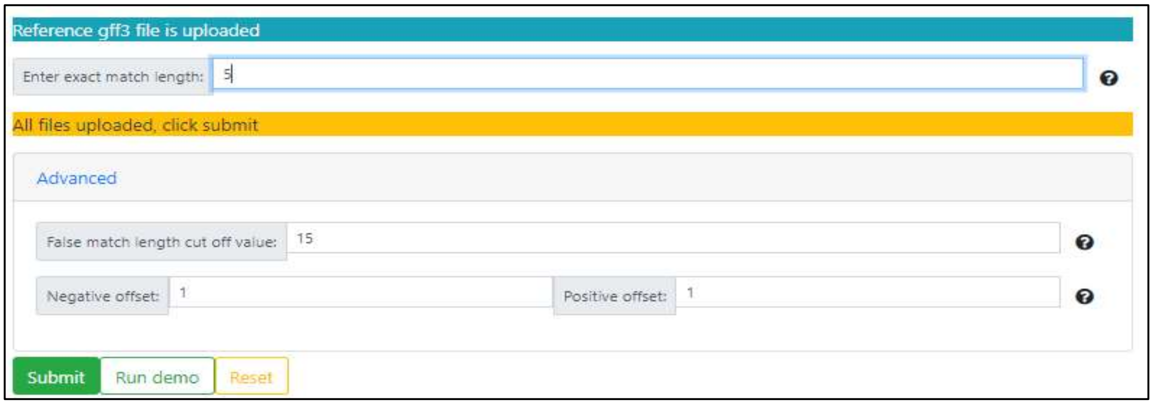

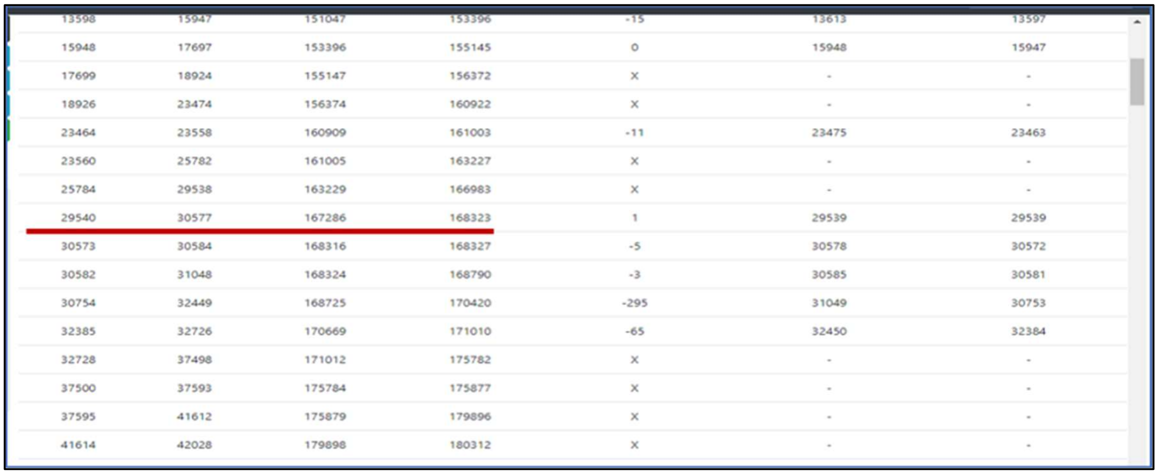

13598	15947	151047	153396	-15	13613	13597
15948	17697	153396	155145	0	15948	15947
17699	18924	155147	156372	X	-	-
18926	23474	156374	160922	X	-	-
23464	23558	160909	161003	-11	23475	23463
23560	25782	161005	163227	X	-	-
25784	29538	163229	166983	X	-	-
29540	30577	167286	168323	1	29539	29539
30573	30584	168316	168327	-5	30578	30572
30582	31048	168324	168790	-3	30585	30581
30754	32449	168725	170420	-295	31049	30753
32385	32726	170669	171010	-65	32450	32384
32728	37498	171012	175782	X	-	-
37500	37593	175784	175877	X	-	-
37595	41612	175879	179896	X	-	-
41614	42028	179898	180312	X	-	-

6. RNA viruses – Tweaking with False match length and offset window

RNA viruses pose challenge because it is difficult to obtain exact matches of sufficient length between query and reference due to high mutations in their genomes. To suit such cases, few parameters have been included as advanced options in MRF page. How the advanced features of MRF can be exploited to get appropriate results has been demonstrated here with the example of HIV-1 virus genomes. HIV-1 viruses are RNA viruses with genome size of about 9 – 10 kb.

MRF will be demonstrated with different settings to show how these parameters affect the results. The files used for this demo are the ICTV strain as reference (Accession no: AF033819) and an Indian strain as query (Accession no: KF766537).

6.1 Exact match length – 20, False match length – 15, Offset window – 1,1 [Default]

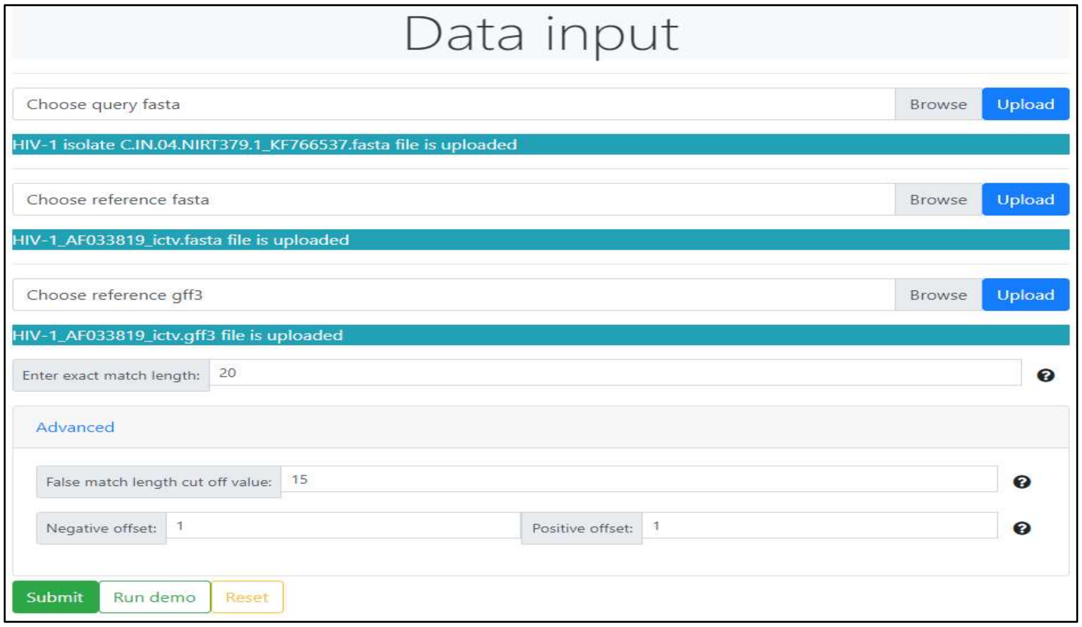

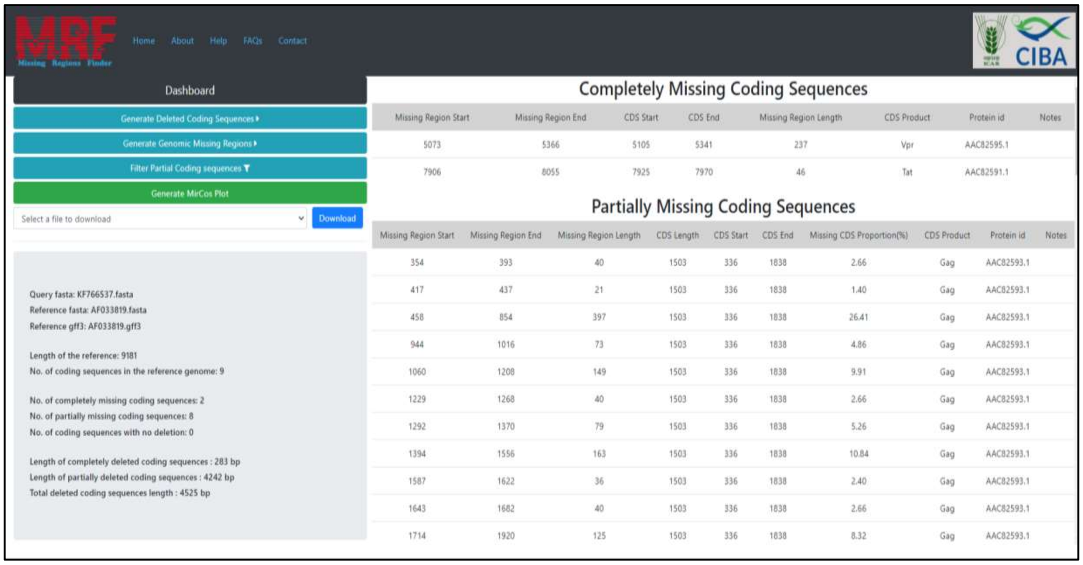

Observe the output summary given below the 'Dashboard' window. Two and eight CDS were completely missing and partially missing# respectively in the query genome and the total deleted coding sequence length was 6924 bp. These large numbers are owed to the inability of MRF to obtain exact matches between query and reference with a mum length of 20. The regions with mismatches prevent the formation of exact matches of 20 bp and these regions would be counted in missing regions list. This issue can be mitigated by reducing the mum length.

Though the reference has only 9 proteins, *tat* protein has two isoforms and one isoform is completely lost while the other is partially lost, so the discrepancy in number

6.2 Exact match length – 10, False match length – 0, Offset window – 0,0

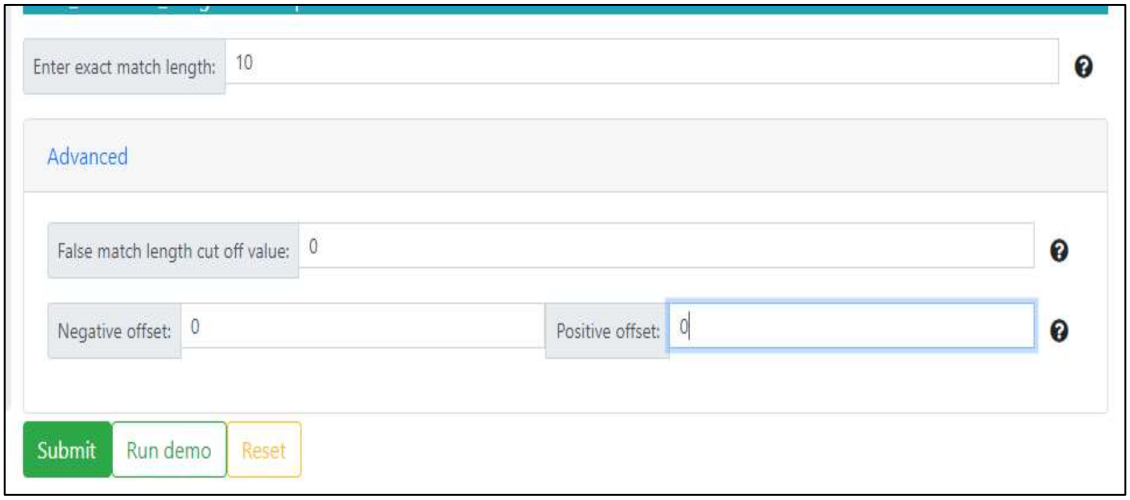

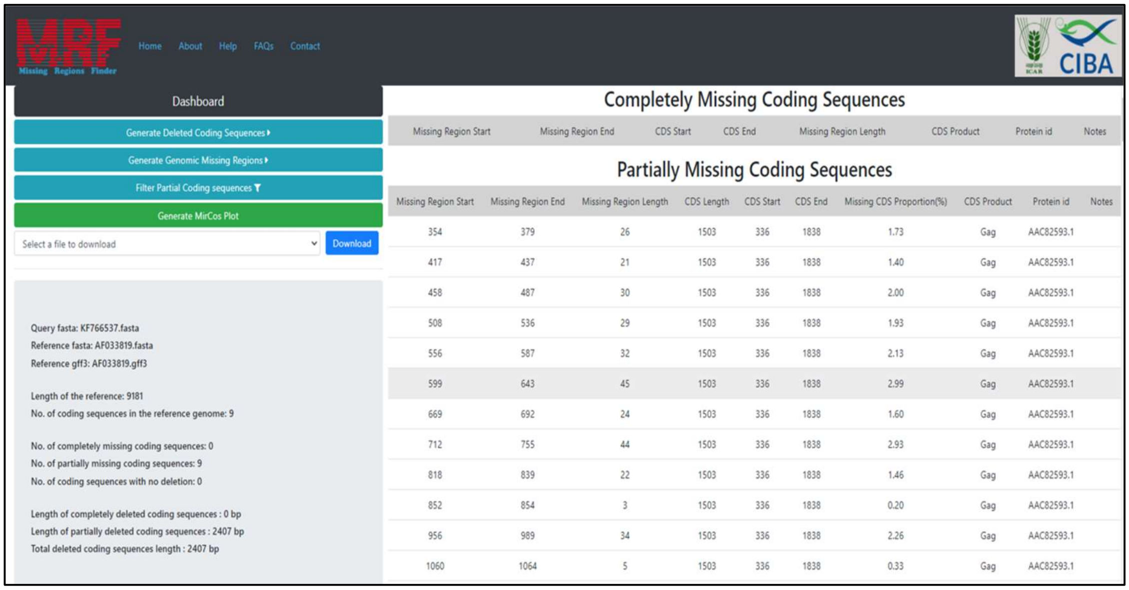

Reduced mum length resulted in a decrease in ‘deleted coding sequences length’. With shorter mum length, more exact matches could be made between query and reference which were not possible with a mum length of 20. Now, observe the missing genomic regions table. We observe a new problem here. Some of the exact matches were false. This might be due to formation of exact matches at random locations in genome due to short mum length. Notice the highlighted false matches in the screenshot given below.

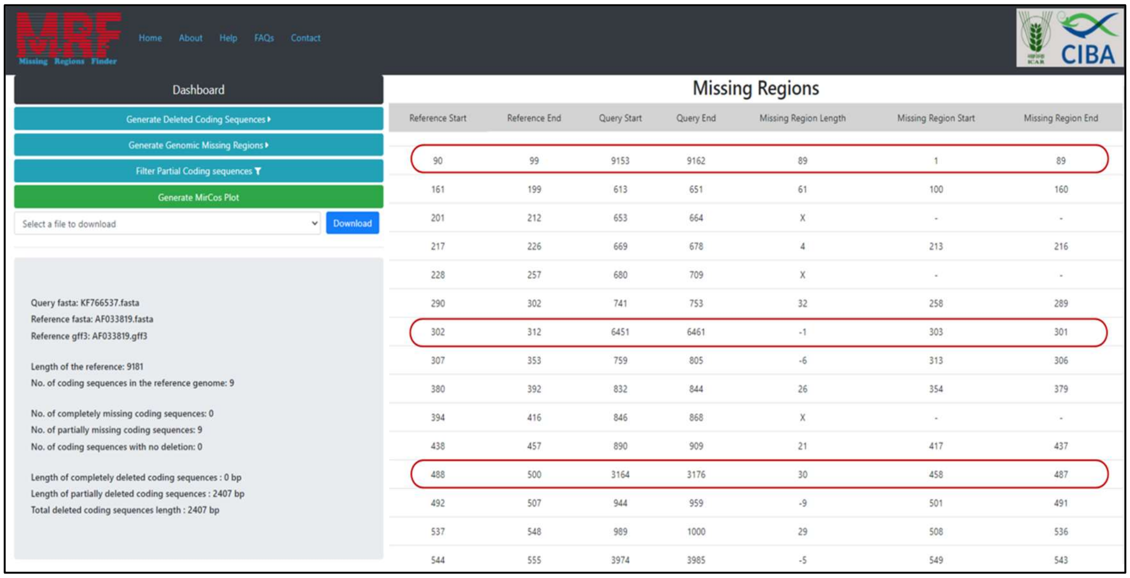

From the above image, it can be observed that there are few false matches, which in turn mask the true missing regions. For example in the above case, the actual missing region is from 1-160 bp of the reference, which should give a non-hit region of 160bp. Since there is a false hit at base 90-99, the non-hit region is split in to two regions of 89 bp and 61 bp separated by a false match region (90-99 bp). These false matches can be eliminated by utilizing the false match and offset window parameters.

6.3 Mum length – 10, False match length – 15, Offset window – 1,1

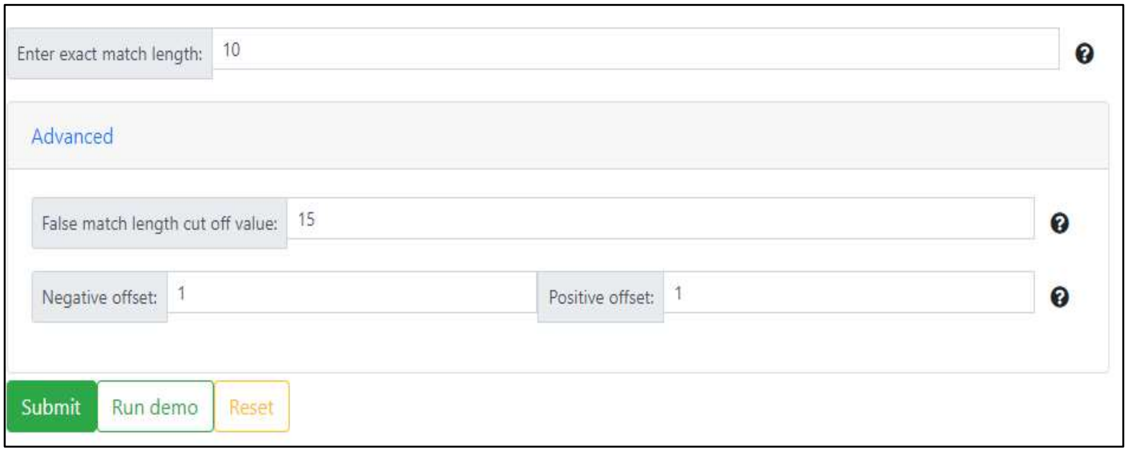

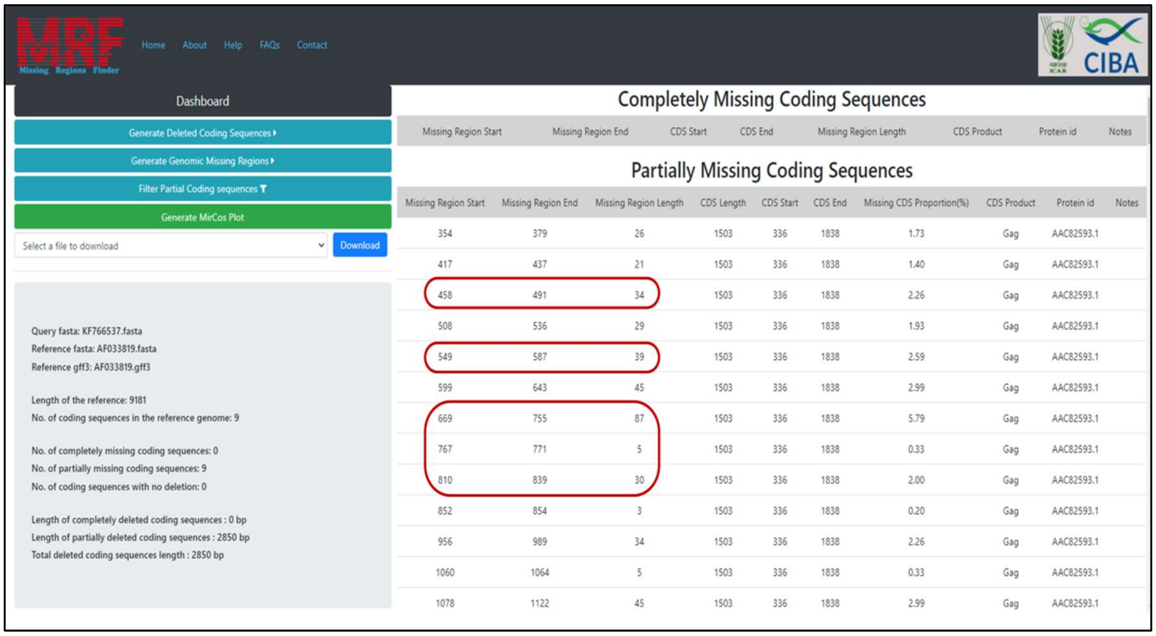

Missing Region Start	Missing Region End	Missing Region Length	CDS Length	CDS Start	CDS End	Missing CDS Proportion(%)	CDS Product	Protein id	Notes
354	379	26	1503	336	1838	1.73	Gag	AAC82593.1	
417	437	21	1503	336	1838	1.40	Gag	AAC82593.1	
458	491	34	1503	336	1838	2.26	Gag	AAC82593.1	
508	536	29	1503	336	1838	1.93	Gag	AAC82593.1	
549	587	39	1503	336	1838	2.59	Gag	AAC82593.1	
599	643	45	1503	336	1838	2.99	Gag	AAC82593.1	
669	755	87	1503	336	1838	5.79	Gag	AAC82593.1	
767	771	5	1503	336	1838	0.33	Gag	AAC82593.1	
810	839	30	1503	336	1838	2.00	Gag	AAC82593.1	
852	854	3	1503	336	1838	0.20	Gag	AAC82593.1	
956	989	34	1503	336	1838	2.26	Gag	AAC82593.1	
1060	1064	5	1503	336	1838	0.33	Gag	AAC82593.1	
1078	1122	45	1503	336	1838	2.99	Gag	AAC82593.1	

With the new parameters, many false matches were eliminated and many missing regions have appeared (some were extended and some were merged due to the elimination of false matches). This can be clearly observed in missing regions table in the following image where the elimination of false matches was depicted by a red line.

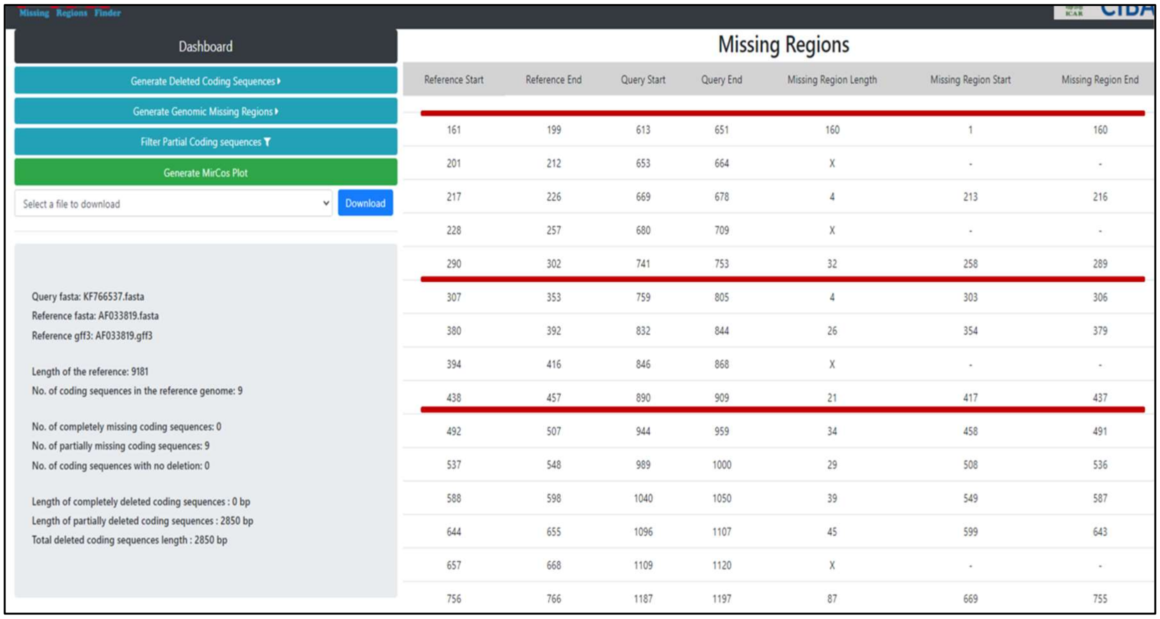

Reference Start	Reference End	Query Start	Query End	Missing Region Length	Missing Region Start	Missing Region End
161	199	613	651	160	1	160
201	212	653	664	X	-	-
217	226	669	678	4	213	216
228	257	680	709	X	-	-
290	302	741	753	32	258	289
307	353	759	805	4	303	306
380	392	832	844	26	354	379
394	416	846	868	X	-	-
438	457	890	909	21	417	437
492	507	944	959	34	458	491
537	548	989	1000	29	508	536
588	598	1040	1050	39	549	587
644	655	1096	1107	45	599	643
657	668	1109	1120	X	-	-
756	766	1187	1197	87	669	755

The false matches reported in the previous run (run 6.2) were eliminated by setting the false match cut-off to 15.

To further circumvent the errors in reporting the continuous stretch of exact matches, the mum length can be further reduced to 5, however it will drastically result in many

more false matches. How the results would vary and how they can be mitigated is demonstrated below

6.4 Mum length – 5, False match length – 0, Offset window – 0,0

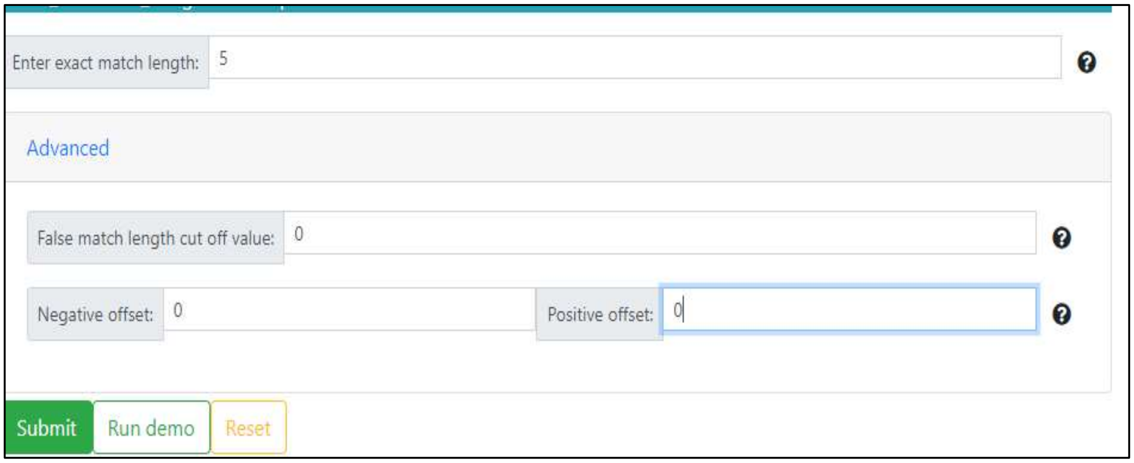

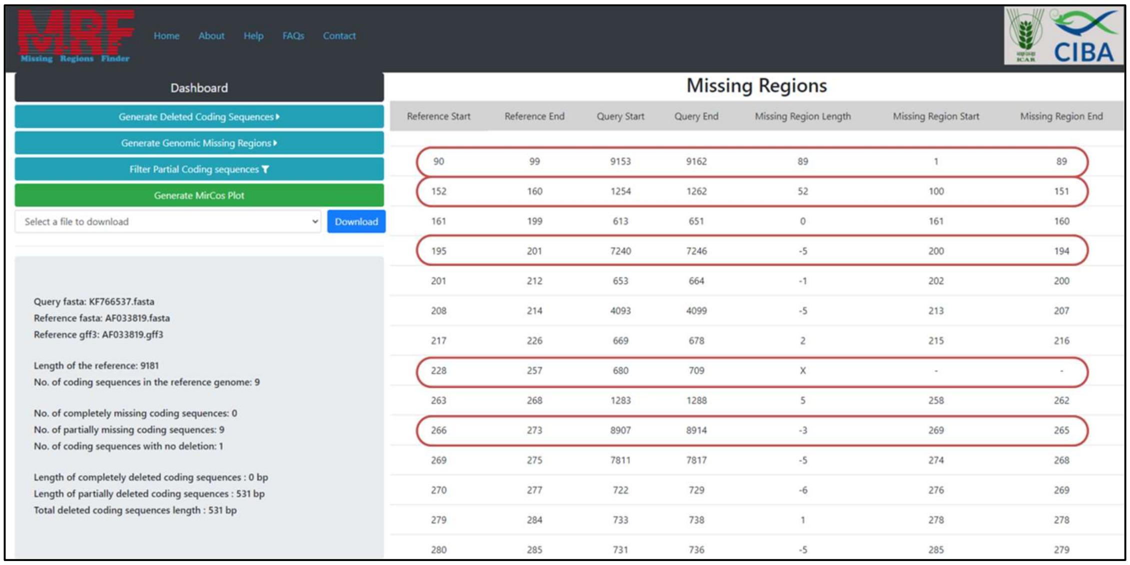

Reference Start	Reference End	Query Start	Query End	Missing Region Length	Missing Region Start	Missing Region End
90	99	9153	9162	89	1	89
152	160	1254	1262	52	100	151
161	199	613	651	0	161	160
195	201	7240	7246	-5	200	194
201	212	653	664	-1	202	200
208	214	4093	4099	-5	213	207
217	226	669	678	2	215	216
228	257	680	709	X	-	-
263	268	1283	1288	5	258	262
266	273	8907	8914	-3	269	265
269	275	7811	7817	-5	274	268
270	277	722	729	-6	276	269
279	284	733	738	1	278	278
280	285	731	736	-5	285	279

Due to lowering of exact match length to 5, several false matches have resulted, some of which can be eliminated when MRF is run with the below settings.

6.5 Mum length – 5, False match length – 15, Offset window – 1,1

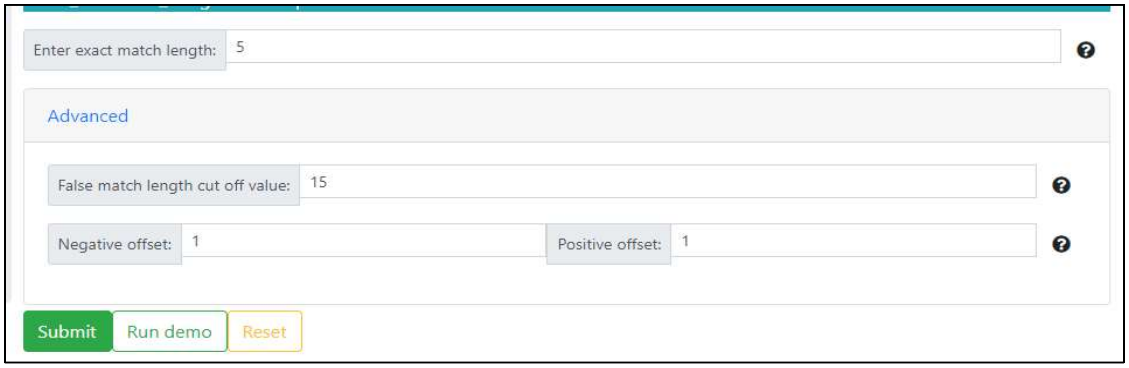

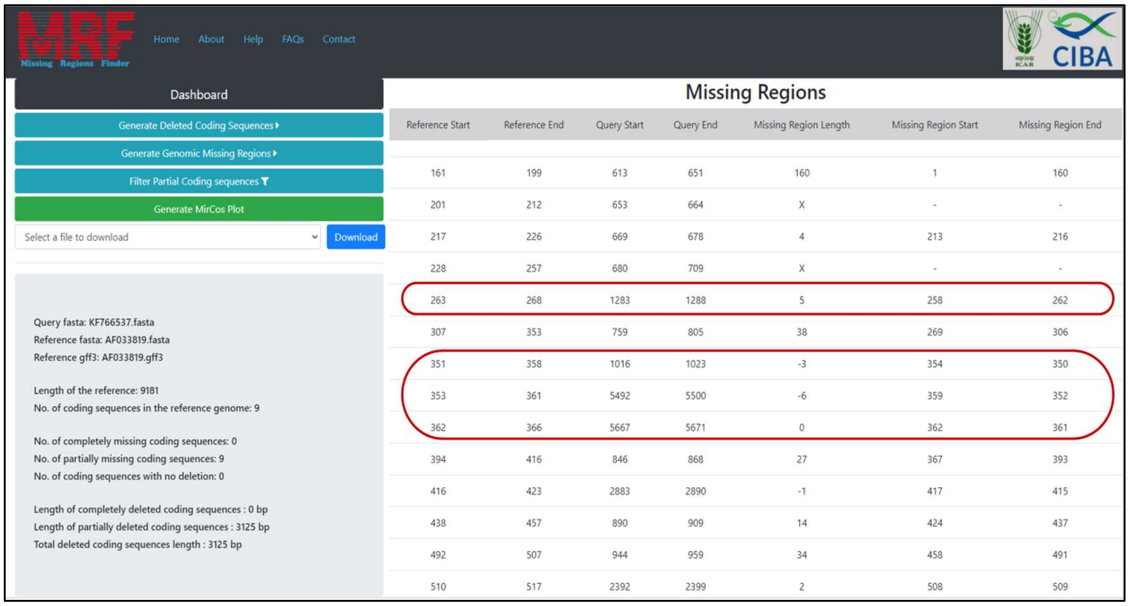

Reference Start	Reference End	Query Start	Query End	Missing Region Length	Missing Region Start	Missing Region End
161	199	613	651	160	1	160
201	212	653	664	X	-	-
217	226	669	678	4	213	216
228	257	680	709	X	-	-
263	268	1283	1288	5	258	262
307	353	759	805	38	269	306
351	358	1016	1023	-3	354	350
353	361	5492	5500	-6	359	352
362	366	5667	5671	0	362	361
394	416	846	868	27	367	393
416	423	2883	2890	-1	417	415
438	457	890	909	14	424	437
492	507	944	959	34	458	491
510	517	2392	2399	2	508	509

Though several false matches have been removed, still some persist. This can be addressed by adjusting offset window parameter.

6.6 Mum length – 5, False match length – 15, Offset window – 2,2

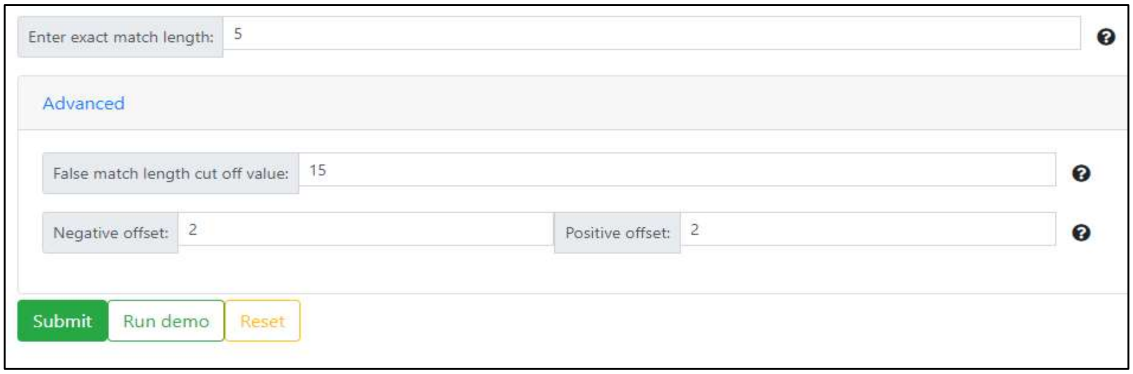

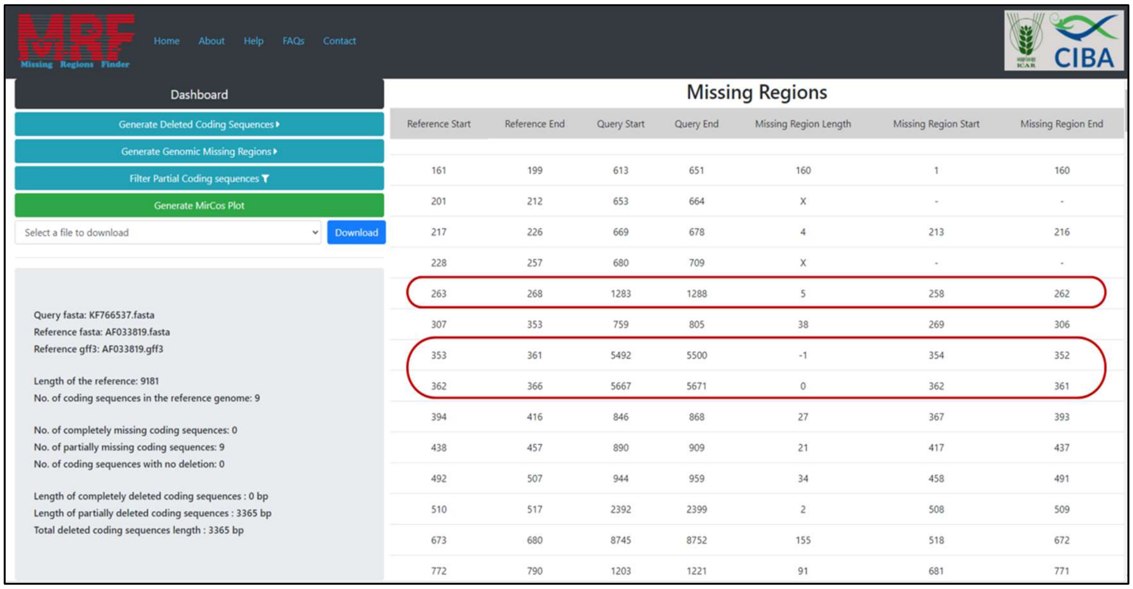

Reference Start	Reference End	Query Start	Query End	Missing Region Length	Missing Region Start	Missing Region End
161	199	613	651	160	1	160
201	212	653	664	X	-	-
217	226	669	678	4	213	216
228	257	680	709	X	-	-
263	268	1283	1288	5	258	262
307	353	759	805	38	269	306
353	361	5492	5500	-1	354	352
362	366	5667	5671	0	362	361
394	416	846	868	27	367	393
438	457	890	909	21	417	437
492	507	944	959	34	458	491
510	517	2392	2399	2	508	509
673	680	8745	8752	155	518	672
772	790	1203	1221	91	681	771

6.7 Mum length – 5, False match length – 15, Offset window – 3,3

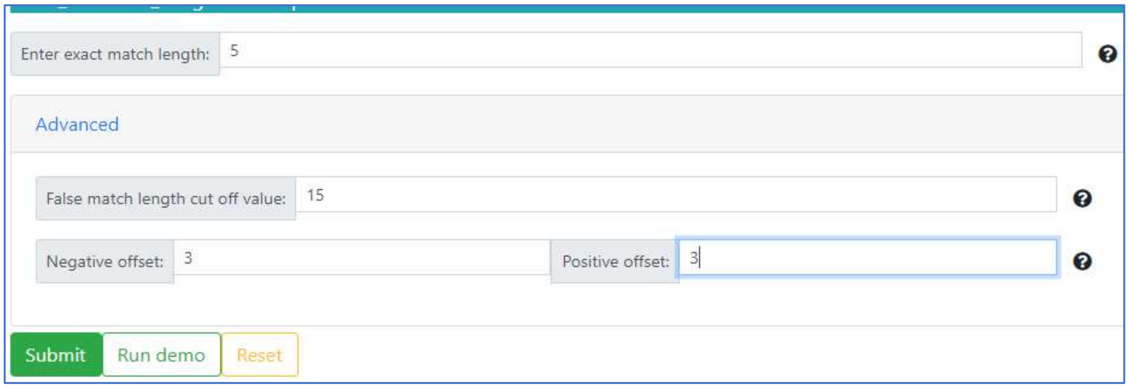

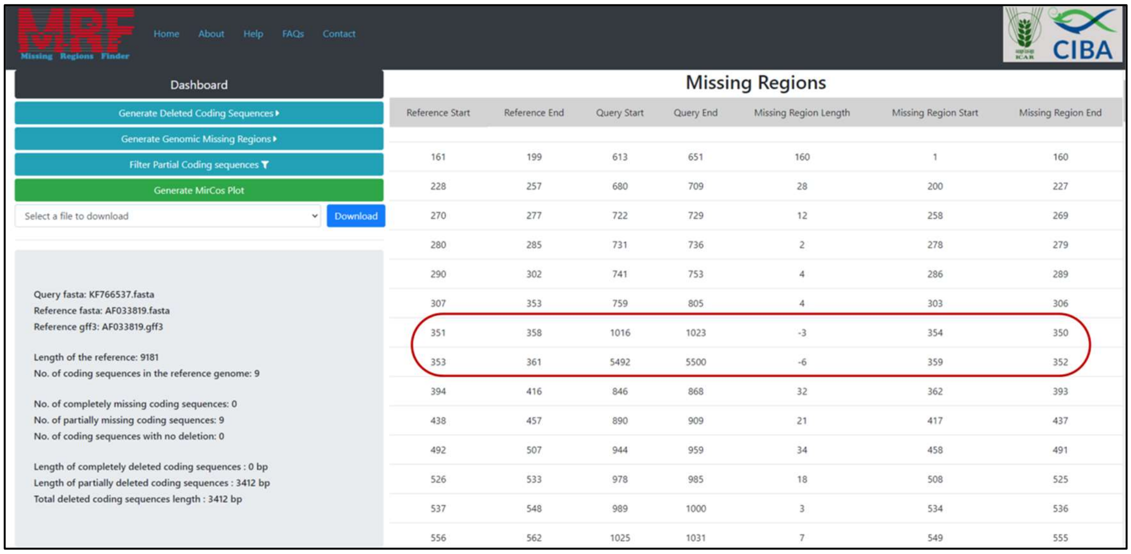

Reference Start	Reference End	Query Start	Query End	Missing Region Length	Missing Region Start	Missing Region End
161	199	613	651	160	1	160
228	257	680	709	28	200	227
270	277	722	729	12	258	269
280	285	731	736	2	278	279
290	302	741	753	4	286	289
307	353	759	805	4	303	306
351	358	1016	1023	-3	354	350
353	361	5492	5500	-6	359	352
394	416	846	868	32	362	393
438	457	890	909	21	417	437
492	507	944	959	34	458	491
526	533	978	985	18	508	525
537	548	989	1000	3	534	536
556	562	1025	1031	7	549	555

6.8 Mum length – 5, False match length – 15, Offset window – 4,4

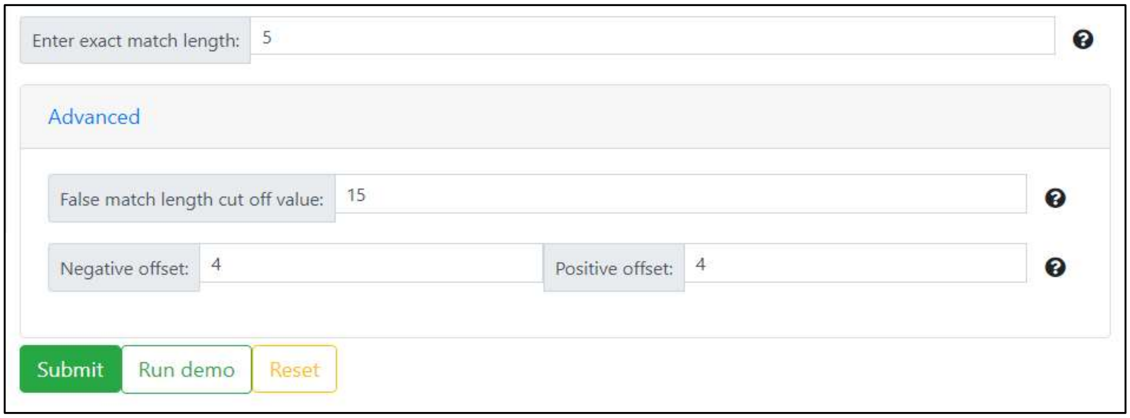

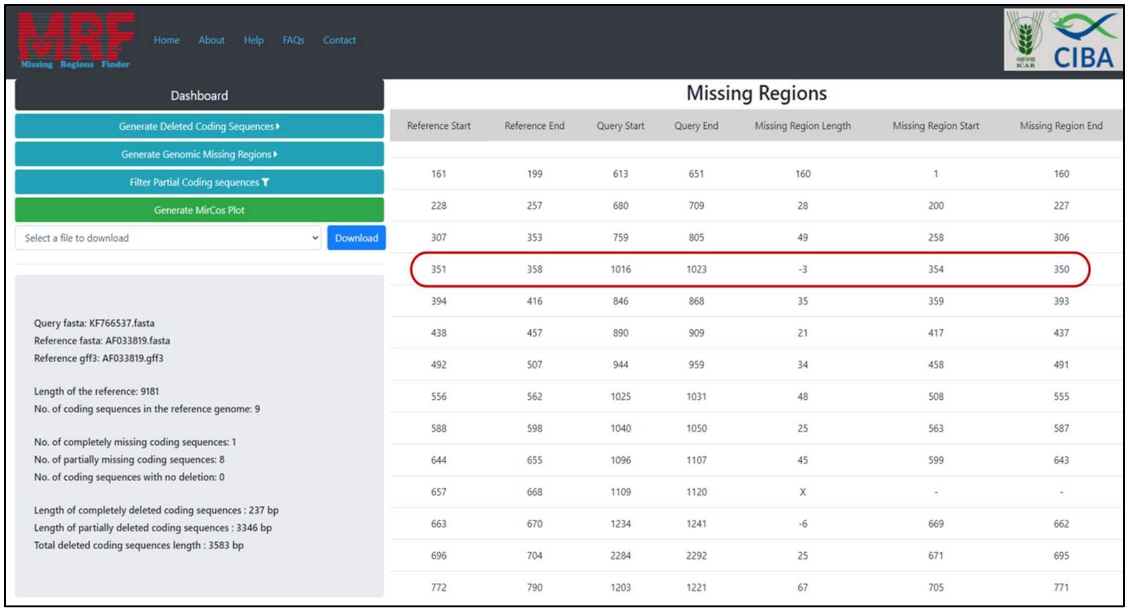

Through runs from 4 to 8, notice that several false matches were being eliminated by adjusting advanced features like, false match length and offset parameters even with a very short mum length of 5. However, few false matches still remain as you cannot completely avoid exact matches at random locations with very short mum lengths in genomes with high mutations and repeat regions. Even similarity search tools like blast err in reporting false alignments with hits to random locations (explained in Appendix II with use cases). There is never a perfect parameter setting that works universally for all the genomes. Therefore, users are advised to balance between the mum length and false match window parameters especially while handling highly mutable genomes. However, for most of DNA virus genomes, output of MRF is straightforward with basic parameters.

7. Filtering partially missing coding sequences

By default, MRF prints all the partially missing coding sequences irrespective of their length or percentage of deletion. However, this output can be controlled by setting a threshold under the ‘Filter Partially Missing Coding sequences’ tab.

The below example show all the partially missing coding sequences by using exact match length of 10.

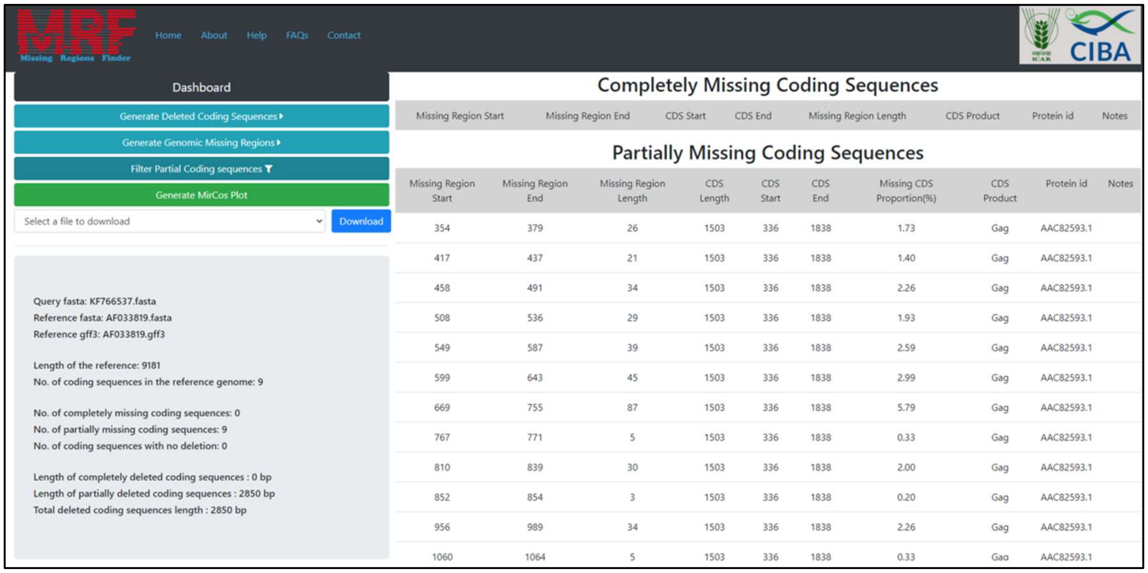

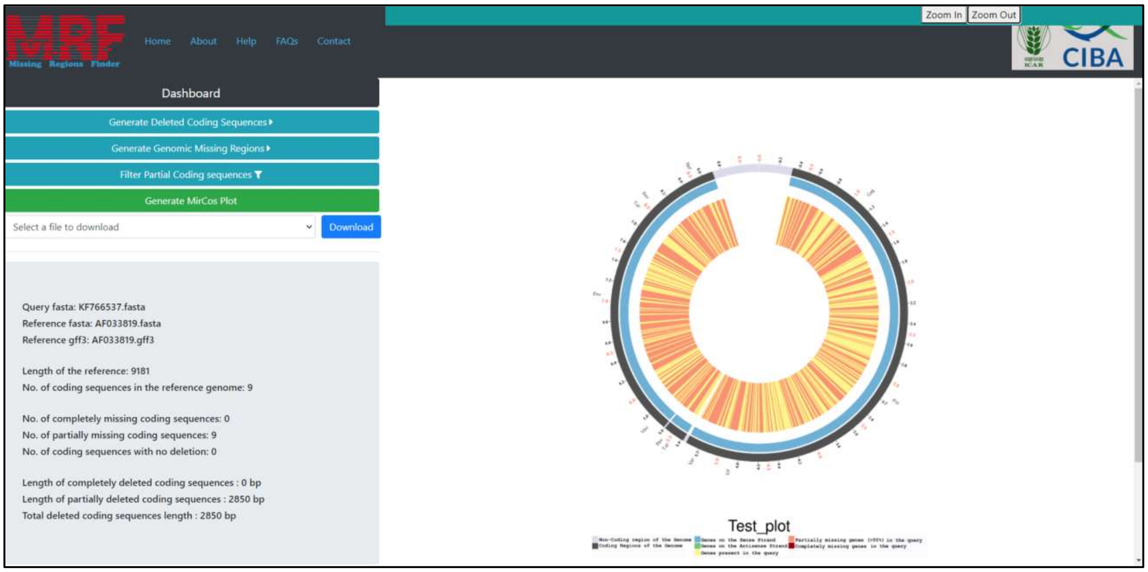

It is evident that many are miniscule missing coding sequences and most likely insignificant. By setting the threshold as 10 percent, most of them are filtered out from the results. This is shown in the below screenshots.

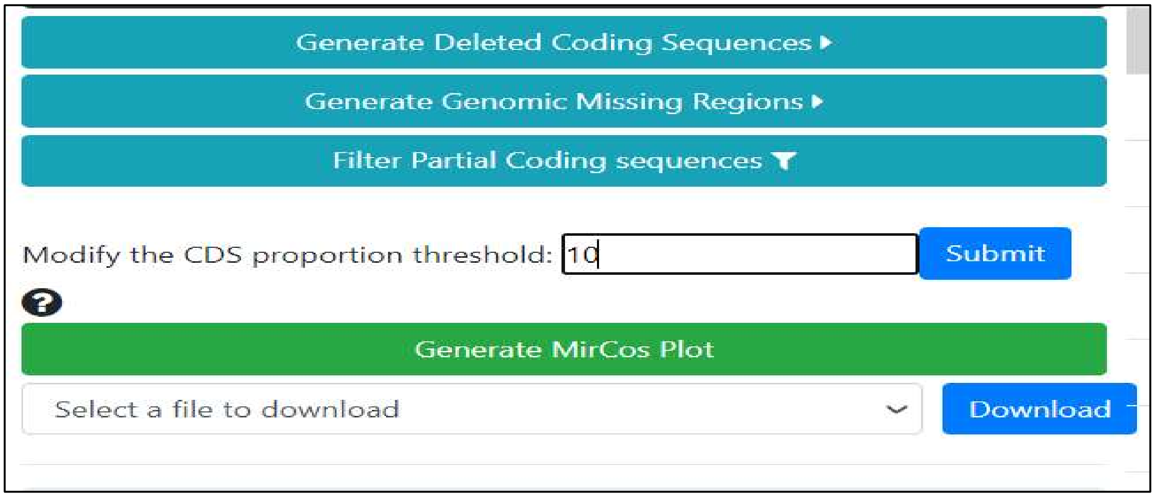

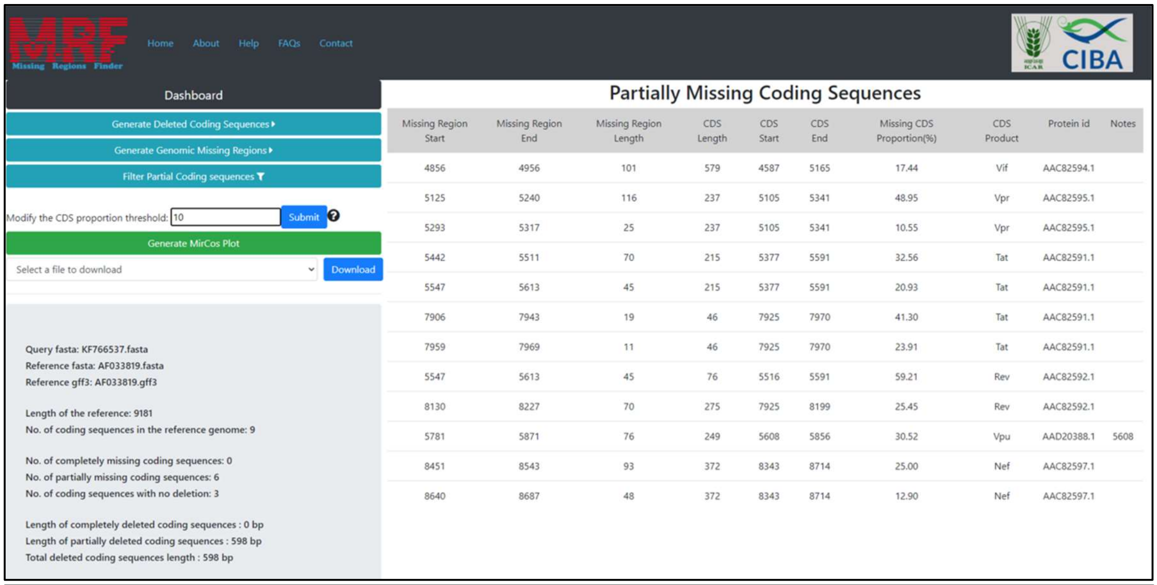

Partially Missing Coding Sequences

Missing Region Start	Missing Region End	Missing Region Length	CDS Length	CDS Start	CDS End	Missing CDS Proportion(%)	CDS Product	Protein id	Notes
4856	4956	101	579	4587	5165	17.44	Vif	AAC82594.1	
5125	5240	116	237	5105	5341	48.95	Vpr	AAC82595.1	
5293	5317	25	237	5105	5341	10.55	Vpr	AAC82595.1	
5442	5511	70	215	5377	5591	32.56	Tat	AAC82591.1	
5547	5613	45	215	5377	5591	20.93	Tat	AAC82591.1	
7906	7943	19	46	7925	7970	41.30	Tat	AAC82591.1	
7959	7969	11	46	7925	7970	23.91	Tat	AAC82591.1	
5547	5613	45	76	5516	5591	59.21	Rev	AAC82592.1	
8130	8227	70	275	7925	8199	25.45	Rev	AAC82592.1	
5781	5871	76	249	5608	5856	30.52	Vpu	AAD20388.1	5608
8451	8543	93	372	8343	8714	25.00	Nef	AAC82597.1	
8640	8687	48	372	8343	8714	12.90	Nef	AAC82597.1	

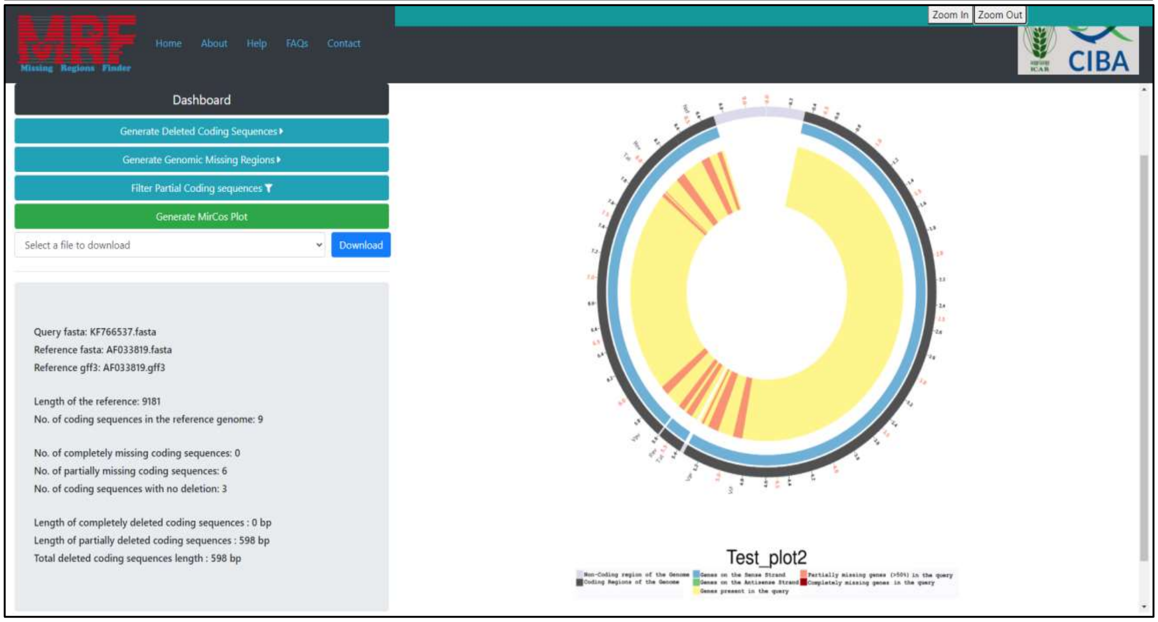

8. MRF for comparing multiple genomes – Batch mode

The standard usage of MRF is to compare a query genome with a reference genome and get the deletions in coding sequences (CDS) of query genome in the nomenclature of the reference genome. This gives a complete picture of the CDS in terms of complete, partial and no deletions in a MirCos plot along with the detailed coordinates in tables. While this usage is adequate for passively sequenced virus genomes such as those involved in causing diseases in poultry, aquaculture and other organisms of interests. However in circumstances such as viruses affecting human populations causing pandemic or endemic, there will be a concerted effort to sequence genomes in a large scale resulting in thousands of complete genomes available online eg. SARS-COV2, HIV, ZIKA etc. It is worthwhile noticing that even passively sequenced virus genomes are in large numbers.

In these scenarios, the standard usage of MRF may not be well suited, as one would want to see all the genomes at once and infer a pattern or narrow down the ones that are affected most. This can be achieved by running the MRF-batch program. This program is currently command line only.

8.1 Installation

Dependencies

There are very minimal dependencies for running MRF-batch program. Verify if you have the following and install accordingly. Alternatively one can create a conda environment with requisite packages (scroll below)

1. Perl v5
2. Bash
3. R > v3.0 and the following packages.
   i) RColorBrewer_1.1-2
   ii) tidyr_1.1.2
   iii) plyr_1.8.4
   iv) gplots_3.0.1.1
   v) ggplot2_3.1.1
   vi) dplyr_1.0.2

If you meet the above dependencies, clone the git repository (link), cd into the directory and start running the program.

Through conda

Clone the github repository (https://github.com/vinayciba/MRF)

```
cd mrf
conda env create -f MRF.conda_env.yml
source activate MRF.env
```

8.2 Running MRF-batch program

MRF-batch program is demonstrated with WSSV and SARS-COV2 test cases. The main program which takes care of everything is *batch-run-mrf-wrapper.sh,* it first calls the *MRF-batch.pl* program to generate missing regions and missing coding sequences for all the queried genomes. Next these outputs are parsed with the help of a program called *parse-mrf-output.pl* to generated three files, summaryAll.txt, cdsALL.txt and cdsHeatmap.txt. Finally, a R script, *genPlots.R* will utilize those three files and generates the plots.

Users can choose to run the programs *parse-mrf-output.pl* and *genPlots.R* independently without calling the main program *batch-run-mrf-wrapper.sh*. For example if an user is satisfied with the results of or options given to the *MRF-batch.pl* program, but want to parse the outputs and generate plots in a different manner, he /she can directly run the corresponding programs.

To see the available options of *batch-run-mrf-wrapper.sh* program, run the command given below.

```
bash batch-run-mrf-wrapper.sh -h
```

```
Usage:    batch-run-mrf-wrapper.sh -d query_fasta_directory -r reference_fasta -f
reference_gff3


options: -m      mummer exact match length [default: 20]
         -l      False match length threshold.Values below this will be screened and confirmed
                  if they are true matches [default: 15]
         -n      Negative offset. This option requires False match length as mandatory argument
                  [default: 1]
         -p      Positive offset. This option requires False match length as mandatory argument
                  [default: 1]
         -c      Partial coding sequences below this set threshold are not shown [default: 0]
         -o      output prefix [default: mrfOUT]

Output parsing options:

         -I      Search by protein ids [provide comma separated]
         -N      Search by protein names [provide comma separated]
         -Y      Filter by missing coding sequences length [default: 0]
         -F      List first n proteins [default:10]
         -Z      List last n proteins [default:10]
         -R      List by range[m,n]
         -U      List only top n affected genomes [default: 10]
         -A      List all proteins

Plot options:

        -L      Number of missing coding sequences to show in heatmap [default: 15]
        -B      Number of genomes to show in barplot [default: 50]

        -h      prints usage
```

The main options **(-m,-l,-n and -p**) work in a similar way as described in the section 3.1 and the option **-c** works as described in section 7., except for the **–o** option, which specifies the output file prefix format. As for the Output parsing and Plot options, a simple description has been given in the usage. Nevertheless, how each option works and the expected output of each option is described further with use cases.

Program equivalent options

batch-run-mrf-wrapper.sh	*parse-mrf-output.pl*
-I	--prot_ID
-N	--prot_name
-Y	--filter
-F	--first
-Z	--last
-R	--range
-U	--top
-A	--all
--L	--number
	genPlot.R
-B	*<value>*

8.3 Test case: WSSV

8.3.1 Default run

```
bash batch-run-mrf-wrapper.sh -d query -r Reference.fasta -f Reference.gff3
```

The above command takes three arguments, the –d option takes a directory called 'query' as input and options –r and -f take fasta and gff3 file of the reference respectively. The query directory contains all the genomes to be compared against the reference in the fasta format. The fasta file should be in nucleotide format and allowed extensions are fasta, fa and fna.

Contents of the query directory is listed below

```
AF369029.fasta      AF440570.fasta      JX515788.fasta      KT995470.fasta
KT995471.fasta      KT995472.fasta      KU216744.fasta      KX686117.fasta
KY827813.fasta      MF768985.fasta      MG702567.fasta
```

Once the command is executed, it runs MRF for each genome in the query directory against the reference genome. The output of all the runs are combined and parsed to produce three files called summaryAll.txt, cdsHeatmap.txt and cdsAll.txt . Apart from these three files, four easy to interpret figures, a Scatterplot, a Barplot and two Heatmaps (clustered and unclustered) are produced. These files are explained below.

Output File 1: summaryAll.txt

Table 1: The contents of summaryAll.txt file produced by the batch mode of MRF tool.

Accession	Num_CDS_in_Ref.	Num_completely_missing_CDS	Num_Partially_missing_CDS	Completely_missing_CDS_length	Partially_missing_CDS_length	Total_missing_CDS_length
**MG702567.1**	524	28	250	13728	13174	26902
**KT995471.1**	524	32	31	13774	6746	20520
**KY827813.1**	524	29	32	13165	6186	19351
**MF768985.1**	524	28	34	11399	7039	18438
**KU216744.1**	524	22	34	9116	4067	13183
**AF369029.2**	524	22	10	9116	3045	12161
**KT995470.1**	524	16	28	6233	3526	9759
**JX515788.1**	524	12	16	3441	4006	7447
**KX686117.1**	524	12	16	4322	2107	6429
**KT995472.1**	524	1	7	246	724	970
**AF440570.1**	524	0	21	0	772	772

The Table 1 shows the results of MRF run for each query genome in the query directory against the provided reference genome (AF332093). As described earlier, all the values are reported with respect to the reference genome. The above details are the same as reported in the MRF website for any one vs one run.

For the sake of better understanding, two images are generated out of the summary table to clearly see how the genomes are affected.

Output Image 1: cdsLostvsFreq.png

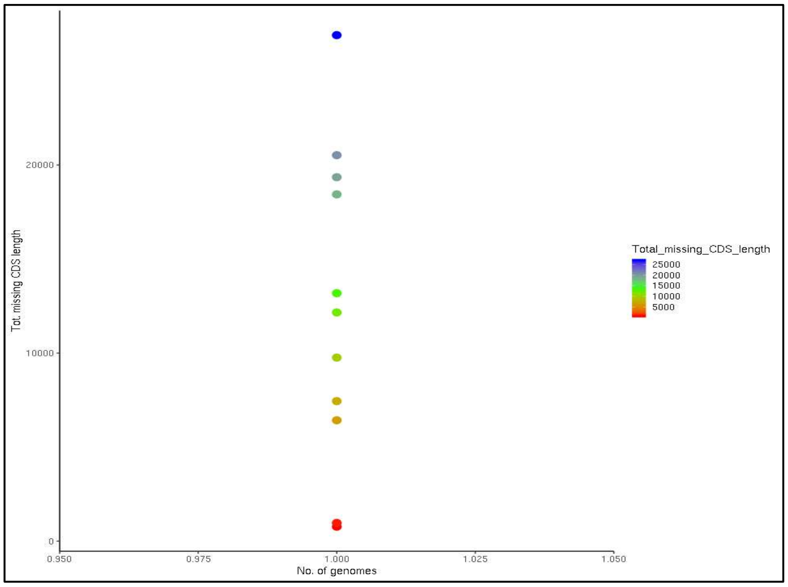

Figure 2: Graph showing each query genome as a point, with varying color. The intensity of color indicates the amount of base pairs lost in the query genome with respect to the reference genome.

Output Image 2: StackedBar.png

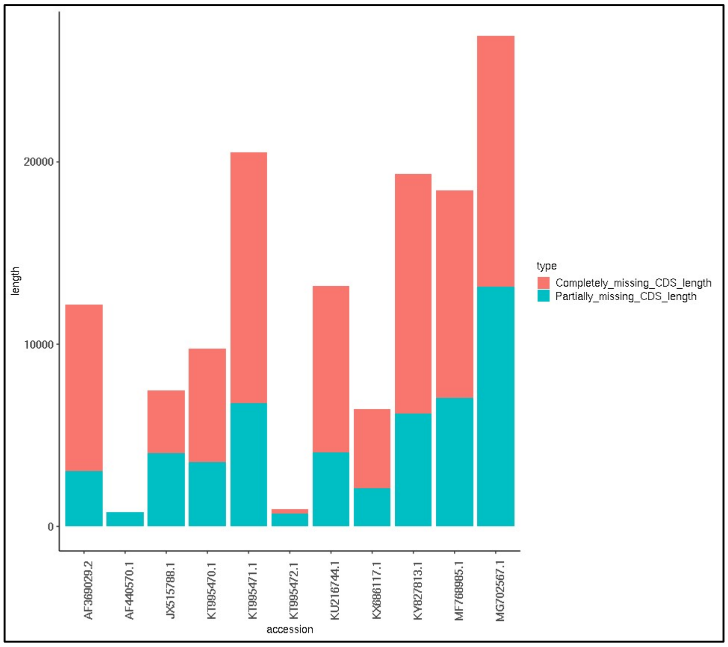

Figure 3: The above plot shows complete and partial missing CDS as a stacked bar, with their combined length indicating the total base lost.

Output File 2: cdsAll.txt

Table 2: The contents of cdsAll.txt file produced by the batch mode of MRF tool.

**#protein_name**	**wsv001**	**wsv002**	**wsv003**	**wsv004**	**wsv005**	**wsv006**	**…**	**wsv526**
**#protein_id**	**AIX03663.1**	**AAL33006.1**	**AAL33007.1**	**AAL33008.1**	**AAL33009.1**	**AAL33010.1**	**…**	**AAL33527.1**
**AF369029.2**	0	0	0	0	0	0	…	0
**AF440570.1**	4	0	0	0	0	0	…	0
**JX515788.1**	60	0	0	0	0	0	…	0
**KT995470.1**	411	0	0	0	0	0	…	0
**KT995471.1**	469	0	0	0	0	0	…	0
**KT995472.1**	0	0	0	0	0	0	…	0
**KU216744.1**	0	0	0	0	0	0	…	0
**KX686117.1**	0	0	0	0	0	0	…	0
**KY827813.1**	1172	298	239	0	0	84	…	0
**MF768985.1**	431	0	0	0	0	0	…	0
**MG702567.1**	1155	0	0	0	0	20	…	42

The Table 2. contains the length of bases lost for all the proteins present in the reference genome. The first two rows indicate CDS identifiers, protein_name and protein_id as reported in the supplied gff3 table of the reference genome. The rest of rows indicate the bases lost for the respective genome (accession in the first column).

In the above example run, the reference genome contains proteins wsv001 through wsv526. Going through these many proteins and inferring the result again becomes very tedious. So a concise table of top fifteen impacted proteins in the similar format as shown in the below table.

Output File 3: cdsHeatmap.txt

Table 3: The contents of cdsHeatmap.txt file produced by the batch mode of MRF tool.

**protein_name**	**wsv001**	**wsv128**	**wsv129**	**wsv178**	**wsv237**	**wsv238**	**wsv338**	**wsv339**	**wsv479**	**wsv482**	**wsv486**	**wsv489**	**wsv492**	**wsv493**	**wsv497**
**MG702567.1**	1155	972	964	672	757	1461	118	724	701	540	2340	738	339	687	1504
**KT995471.1**	469	663	655	909	879	1461	1302	817	688	540	2340	738	339	687	1504
**KY827813.1**	1172	663	655	909	879	1461	1302	817	688	540	2340	738	339	687	1504

**MF768985.1**	431	809	801	909	879	1461	1302	817	688	540	0	738	339	687	1504
**KU216744.1**	0	194	194	109	0	19	0	0	1491	540	2340	738	339	687	1504
**AF369029.2**	0	173	173	0	0	0	0	0	1501	540	2340	738	339	687	1504
**KT995470.1**	411	269	269	253	879	907	0	0	0	0	0	738	339	687	1504
**JX515788.1**	60	565	565	528	0	0	0	0	0	0	0	738	339	687	1511
**KX686117.1**	0	173	173	528	0	0	0	0	0	0	0	738	339	687	1504
**KT995472.1**	0	0	0	325	0	0	0	0	0	0	0	0	0	0	0

The Table 3. is extracted from the cdsAll.txt table by taking the mean of each column (CDS) and retaining the columns with top fifteen mean values. To further see a clear pattern, the above table is shown as a Heatmap.

Output images 3 & 4: HeatmapClusterOn.png and HeatmapNoClustering.png

A) B)

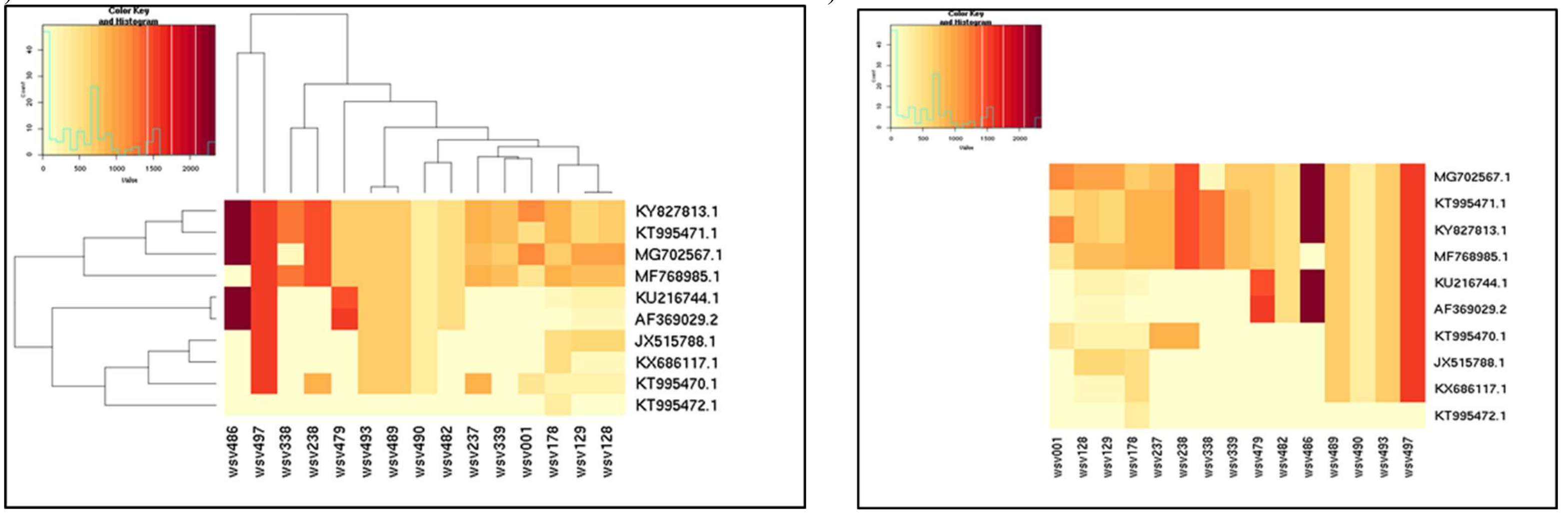

Figure 4 : The heatmaps generated in batch mode run of MRF tool. A) Heat map produced by clutering the missing proteins and genomes. B) Heat map produced without clustering.

Till now, the tables and images described a run with default parameters, however one can parse the cdsAll.txt file in various ways by calling the respective options. Each option is described below with the help of a heatmap (clustered only).

8.3.2 Search by protein id (-I)

Will fetch the CDS that are supplied through the **–I** argument. Only protein ids should be provided as comma separated values.

```
batch-run-mrf-wrapper.sh -d query -r AF332093.fasta -f AF332093.gff3 -o WSSV \
-I AAL33242.1,AAL33340.1,AAL33341.1,AAL33480.1,AIX03693.1,AAL33494.1
```

Output file : cdsHeatmap.txt

protein_name	wsv238	wsv338	wsv339	wsv479	wsv492	wsv493
**MG702567.1**	1461	118	724	701	339	687
**KT995471.1**	1461	1302	817	688	339	687
**KY827813.1**	1461	1302	817	688	339	687
**MF768985.1**	1461	1302	817	688	339	687
**KU216744.1**	19	0	0	1491	339	687
**AF369029.2**	0	0	0	1501	339	687
**KT995470.1**	907	0	0	0	339	687
**JX515788.1**	0	0	0	0	339	687
**KX686117.1**	0	0	0	0	339	687
**KT995472.1**	0	0	0	0	0	0

Output Image :HeatmapClusterOn.png

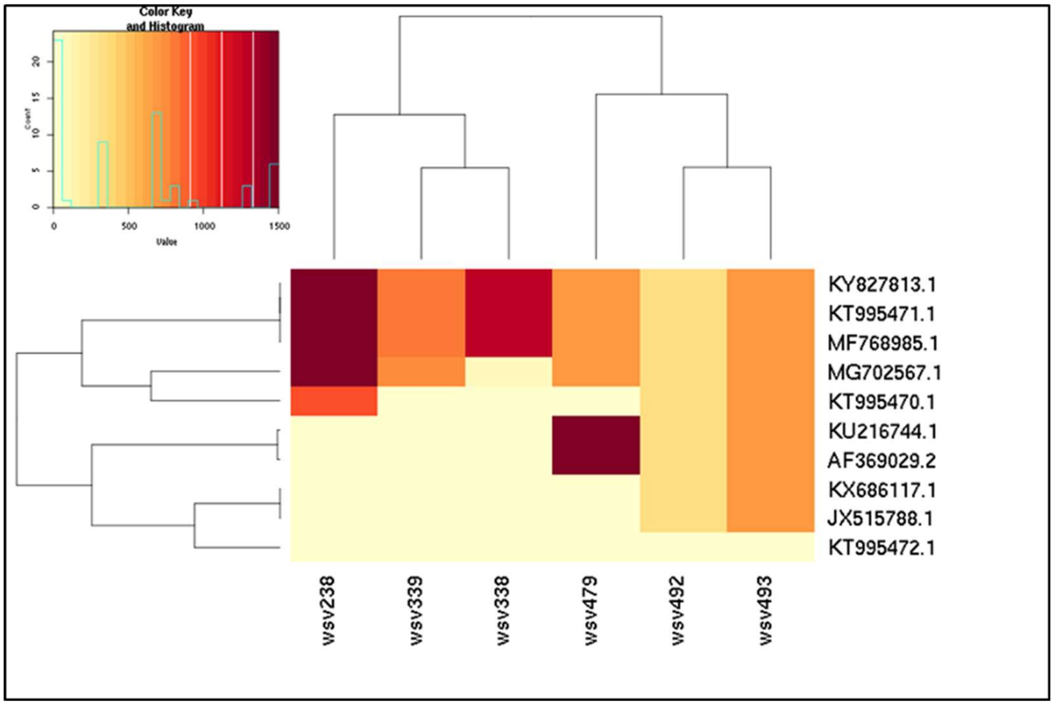

8.3.3 Search by protein names (-N)

Will fetch the CDS that are supplied through the **–I** argument. Only protein names should be provided as comma separated values.

```
bash batch-run-mrf-wrapper.sh -d query -r AF332093.fasta -f AF332093.gff3 \
-o WSSV -N wsv005,wsv050,wsv500,wsv501,wsv502
```

Output File: cdsHeatmap.txt

protein_name	wsv005	wsv050	wsv500	wsv501	wsv502
MG702567.1	0	0	0	0	37
MG702567.1	0	0	0	0	37
KT995471.1	0	0	0	0	0
KT995471.1	0	0	0	0	0
KY827813.1	0	0	0	0	0
KY827813.1	0	0	0	0	0
MF768985.1	0	0	0	0	0
MF768985.1	0	0	0	0	0
KU216744.1	0	0	630	0	688
KU216744.1	0	0	630	0	688

Output Image: HeatmapClusterOn.png

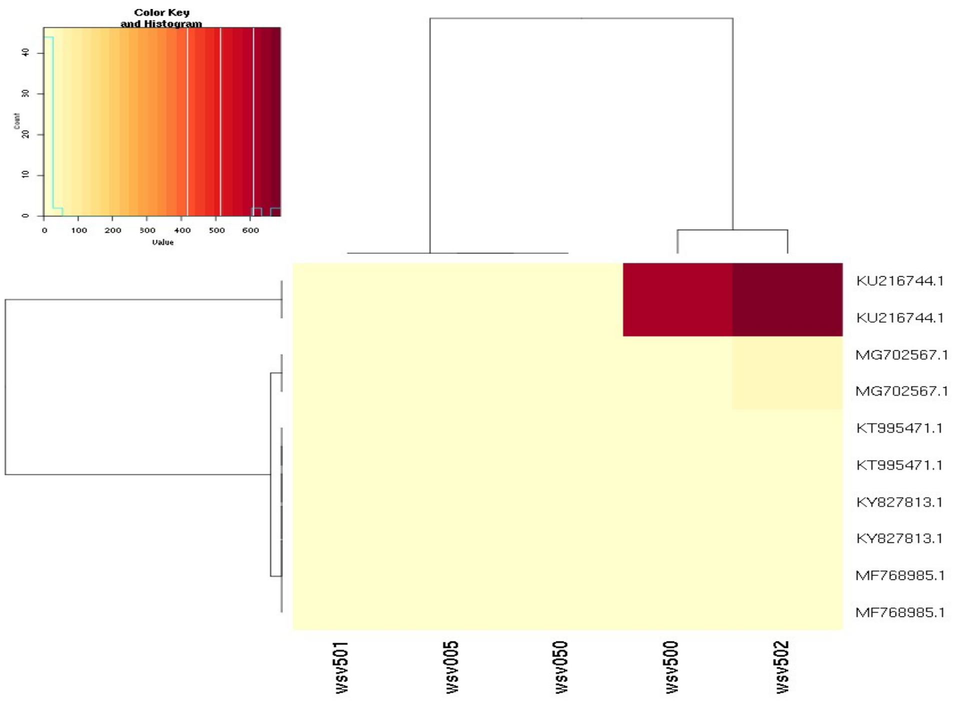

8.3.4 Filter by missing CDS length (-Y)

One can set a threshold value, where coding sequences that meet the threshold are only listed. But one has to exercise caution while setting a very low threshold as this will produce a very busy heatmap, when the number of CDS in the genome is very high, as is the case in WSSV.

```
bash batch-run-mrf-wrapper.sh -d query -r AF332093.fasta -f AF332093.gff3 \
-o WSSV -Y 1000
```

Output File: cdsHeatmap.txt

Protein_name	wsv001	wsv073	wsv238	wsv244	wsv313	wsv338	wsv479	wsv486	wsv497
**MG702567.1**	1155	4	1461	2376	1185	118	701	2340	1504
**KT995471.1**	469	1084	1461	0	0	1302	688	2340	1504
**KY827813.1**	1172	0	1461	0	0	1302	688	2340	1504
**MF768985.1**	431	1084	1461	0	0	1302	688	0	1504
**KU216744.1**	0	0	19	0	0	0	1491	2340	1504
**AF369029.2**	0	0	0	0	0	0	1501	2340	1504
**KT995470.1**	411	0	907	0	0	0	0	0	1504
**JX515788.1**	60	0	0	0	0	0	0	0	1511
**KX686117.1**	0	0	0	0	0	0	0	0	1504
**KT995472.1**	0	0	0	0	0	0	0	0	0

Output Image: HeatmapClusterOn.png

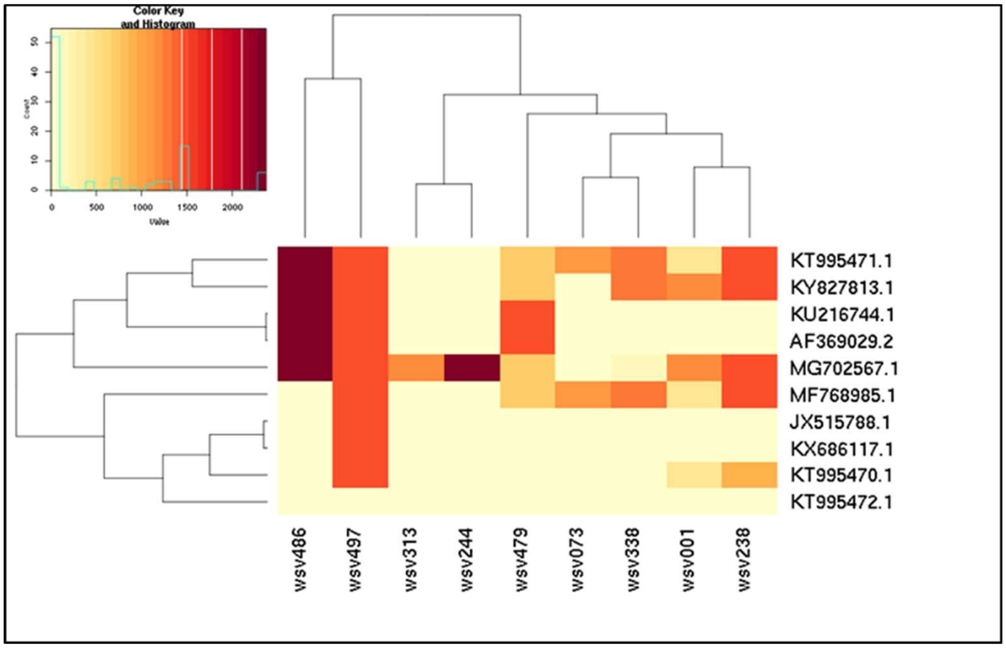

8.3.5 List first n proteins (-F)

To list only first n proteins, call the program with **–F** option. In this example, the first 10 coding sequences are fetched.

```
bash batch-run-mrf-wrapper.sh -d query -r AF332093.fasta -f AF332093.gff3 \
-o WSSV -F 10
```

Output File: cdsHeatmap.txt

Protein_name	wsv001	wsv002	wsv003	wsv004	wsv005	wsv006	wsv007	wsv008	wsv009	wsv010
MG702567.1	1155	0	0	0	0	20	0	20	0	0
KT995471.1	469	0	0	0	0	0	0	0	0	0
KY827813.1	1172	298	239	0	0	84	84	84	0	0
MF768985.1	431	0	0	0	0	0	0	0	0	0
KU216744.1	0	0	0	0	0	0	0	0	0	0
AF369029.2	0	0	0	0	0	0	0	0	0	0
KT995470.1	411	0	0	0	0	0	0	0	0	0
JX515788.1	60	0	0	0	0	0	0	0	0	0
KX686117.1	0	0	0	0	0	0	0	0	0	0
KT995472.1	0	0	0	0	0	0	0	0	0	0

Output Image: HeatmapClusterOn.png

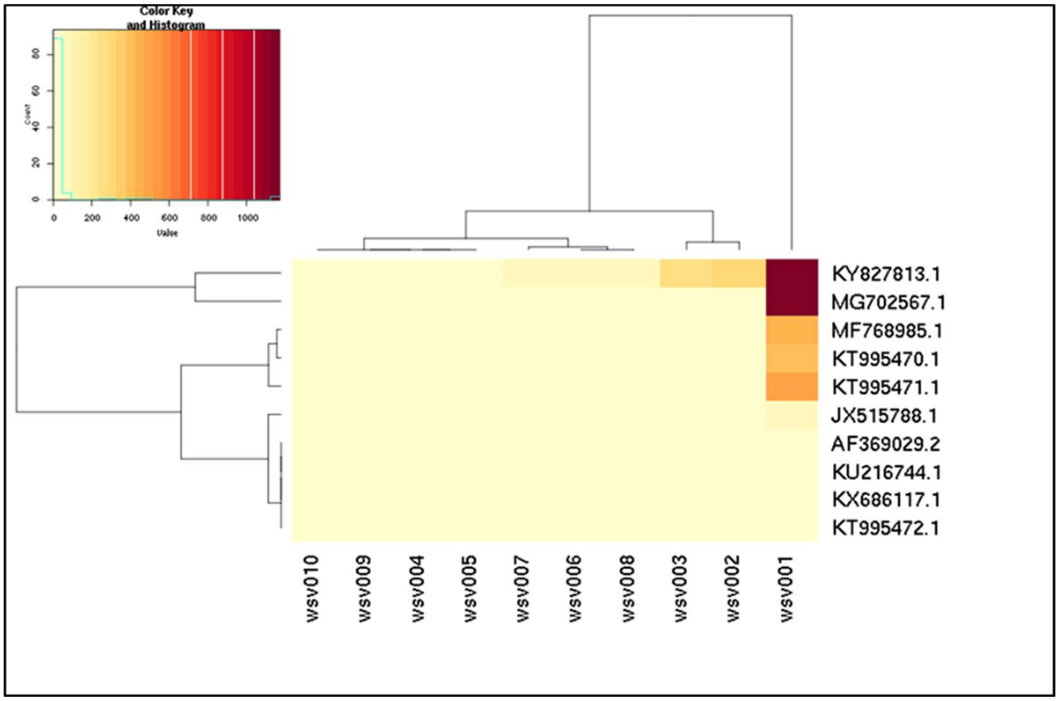

8.3.6 List last n proteins (-Z)

To list only last n proteins, call the program with **–Z** option. In this example, the last 10 coding sequences are fetched.

```
bash batch-run-mrf-wrapper.sh -d query -r AF332093.fasta -f AF332093.gff3 \
-o WSSV -Z 10
```

Output File: cdsHeatmap.txt

Protein_name	wsv526	wsv525	wsv524	wsv523	wsv522	wsv521	wsv520	wsv519	wsv518	wsv517
MG702567.1	42	0	0	0	15	0	7	0	10	0
KT995471.1	0	0	0	0	0	0	0	0	0	0
KY827813.1	0	0	0	0	0	0	0	0	0	0
MF768985.1	0	0	0	0	0	0	0	0	0	0
KU216744.1	0	0	0	0	0	0	0	0	0	0
AF369029.2	0	0	0	0	0	0	0	0	0	0
KT995470.1	0	0	0	0	0	0	0	0	0	0
JX515788.1	0	0	0	0	0	0	0	0	0	0
KX686117.1	0	0	0	0	0	0	0	0	0	0
KT995472.1	0	0	0	0	0	0	0	0	0	0

Output Image: HeatmapClusterOn.png

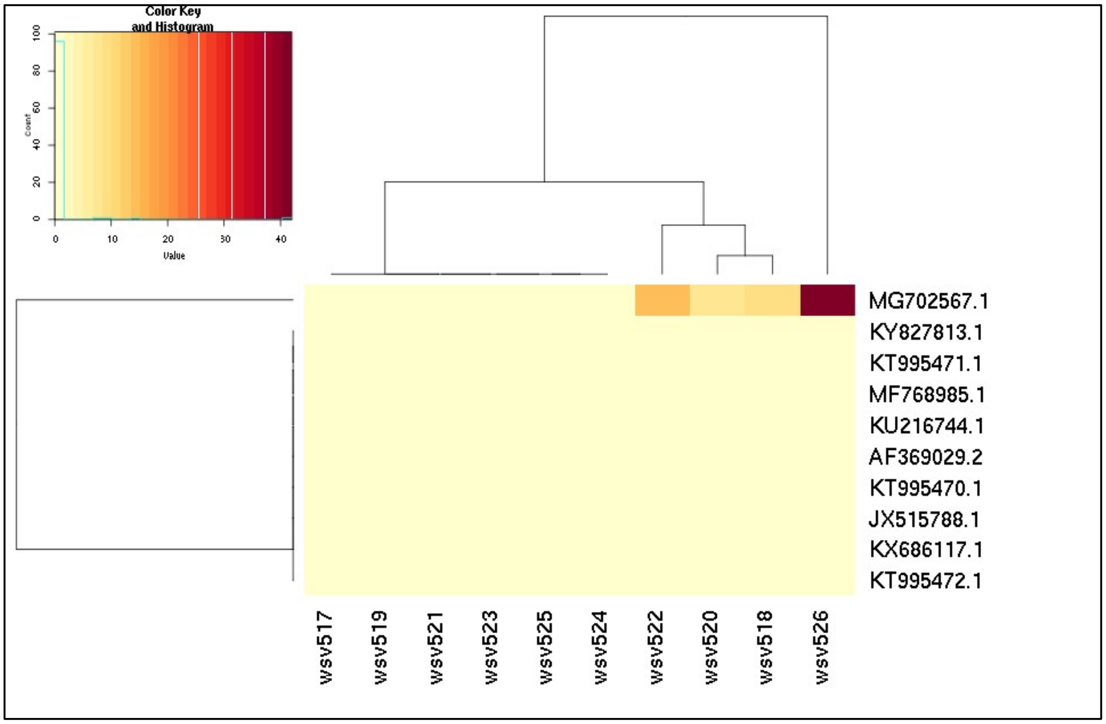

8.3.7 List proteins in range (-R)

To list proteins from CDS 'm' to CDS 'n', call the program with **–R** option. In this example, the coding sequences from 475 to 485 are fetched.

```
bash batch-run-mrf-wrapper.sh -d query -r AF332093.fasta -f AF332093.gff3 \
-o WSSV -R 475,485
```

Output File: cdsHeatmap.txt

**Protein_name**	**wsv475**	**wsv476**	**wsv477**	**wsv478**	**wsv479**	**wsv480**	**wsv481**	**wsv482**	**wsv483**	**wsv484**	**wsv485**
**MG702567.1**	0	19	12	12	701	0	195	540	375	344	50
**KT995471.1**	0	0	0	0	688	0	195	540	375	344	50
**KY827813.1**	138	0	0	0	688	0	195	540	375	344	50
**MF768985.1**	123	0	0	0	688	0	195	540	375	344	249
**KU216744.1**	0	0	0	0	1491	333	195	540	375	344	50
**AF369029.2**	0	0	0	0	1501	333	195	540	375	344	50
**KT995470.1**	0	0	0	0	0	0	0	0	0	0	0
**JX515788.1**	138	0	0	0	0	0	0	0	0	0	0
**KX686117.1**	35	0	0	0	0	0	0	0	0	0	0
**KT995472.1**	0	0	0	0	0	0	0	0	0	0	0

Output Image: HeatmapClusterOn.png

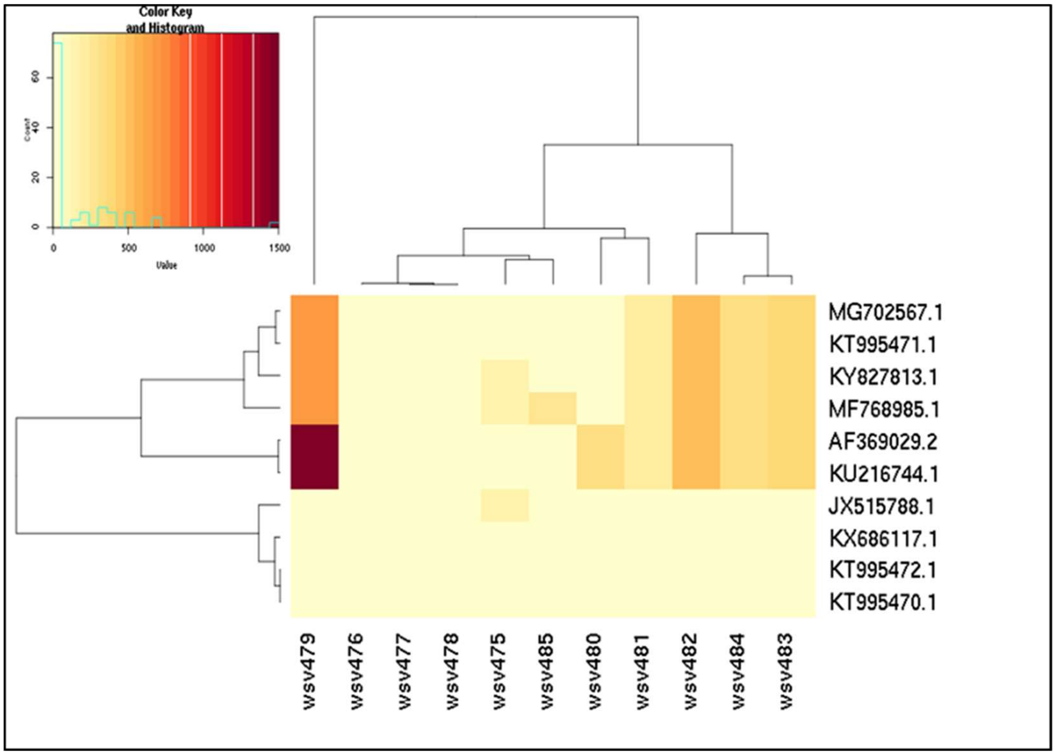

8.3.8 List only top n affected genomes (-U)

By default only top ten affected genomes are listed, however this can be modified by setting the **–U** option. This option can be combined with options **–I/-N/-F/-Z/-R**, as shown in below example it is combined with **–R.**

```
bash batch-run-mrf-wrapper.sh -d query -r AF332093.fasta -f AF332093.gff3 \
-o WSSV –U 11
```

Output File: cdsHeatmap.txt

Protein_name	wsv475	wsv476	wsv477	wsv478	wsv479	wsv480	wsv481	wsv482	wsv483	wsv484	wsv485
MG702567.1	0	19	12	12	701	0	195	540	375	344	50
KT995471.1	0	0	0	0	688	0	195	540	375	344	50
KY827813.1	138	0	0	0	688	0	195	540	375	344	50
MF768985.1	123	0	0	0	688	0	195	540	375	344	249
KU216744.1	0	0	0	0	1491	333	195	540	375	344	50
AF369029.2	0	0	0	0	1501	333	195	540	375	344	50
KT995470.1	0	0	0	0	0	0	0	0	0	0	0
JX515788.1	138	0	0	0	0	0	0	0	0	0	0
KX686117.1	35	0	0	0	0	0	0	0	0	0	0
KT995472.1	0	0	0	0	0	0	0	0	0	0	0
AF440570.1	0	0	0	0	0	0	0	0	0	0	0

Output Image: HeatmapClusterOn.png

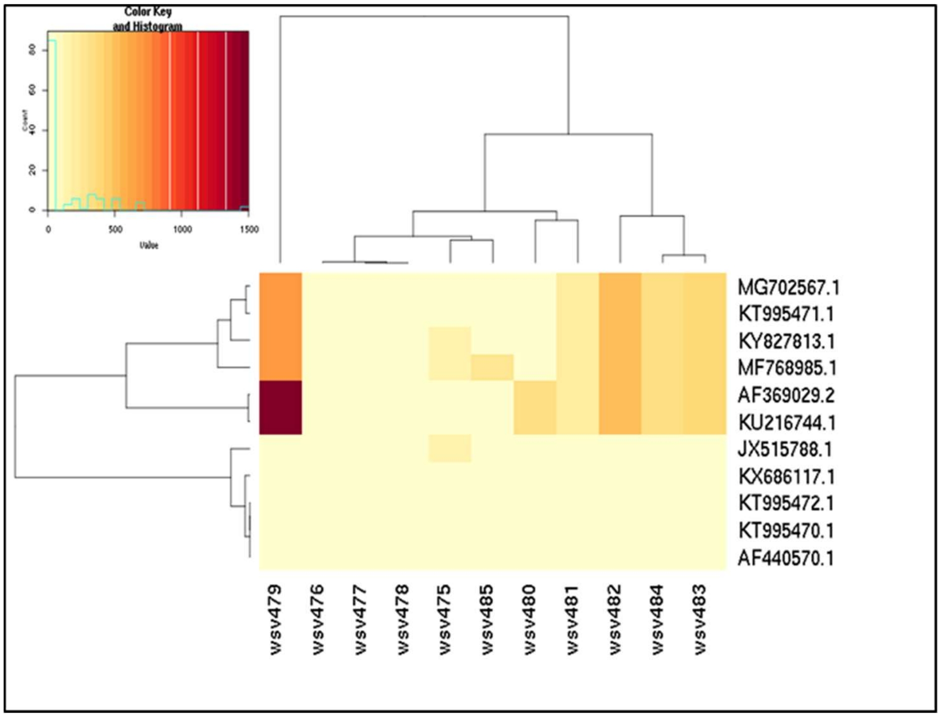

8.4 Plot options:

Options **–L** and**-B** are used to modify the default output of heatmap and Stacked barplot respectively. The **–L** option can be used to increase of decreased the number of coding sequences that are shown in heatmap. The **–B** option is used to increase or decrease the number of genomes shown.

8.4.1 Use case: SARS-COV2

As of 24/12/2020, there are 30927 complete genomes available for the SARS-COV2 virus. It is a time consuming process to compare all these genomic variants. In situations like these, it is better to have a bird's eyeview of everything in order to shortlist them. Since, it is a RNA virus, MRF is run with mum length as 15, with other things kept as default.

```
bash batch-run-mrf-wrapper.sh -d coronaGenomes -r NC_045512.2.fna -f NC_045512.gff3 -m 15 -o SARS2
```

Output File: cdsHeatmap.txt

Protein name	ORF1ab-polyprotein	ORF7b	ORF8-protein	nucleocapsid-phosphoprotein	ORF10-protein	ORF1ab-polyprotein	ORF1a-polyprotein	surface-glycoprotein	ORF3a-protein	envelope-protein	membrane-glycoprotein	ORF6-protein	ORF7a-protein
**LR877184.1**	10432	128	366	1260	117	8088	15	3822	828	228	669	186	366
**LR878228.1**	8559	128	366	801	117	6470	8574	3408	828	228	669	186	366
**LR878240.1**	5304	45	214	53	0	2078	5304	820	45	196	78	186	11
**LR878231.1**	3666	0	0	33	117	2084	3666	1390	0	0	241	0	0
**LR878257.1**	2294	45	161	37	117	1657	2294	1642	0	191	0	0	0
**LR878232.1**	3057	0	65	520	0	1887	3057	604	0	0	0	0	0
**LR877980.1**	1688	33	0	0	0	1645	1688	890	0	0	0	0	242
**LR878233.1**	2073	42	169	3	0	845	2073	873	0	0	0	0	0
**LR878069.1**	1796	0	0	0	0	1380	1796	580	0	0	0	0	0
**LR878242.1**	1710	19	162	0	0	730	1710	795	0	0	236	0	0

Output Image: cdsLostvsFreq.png

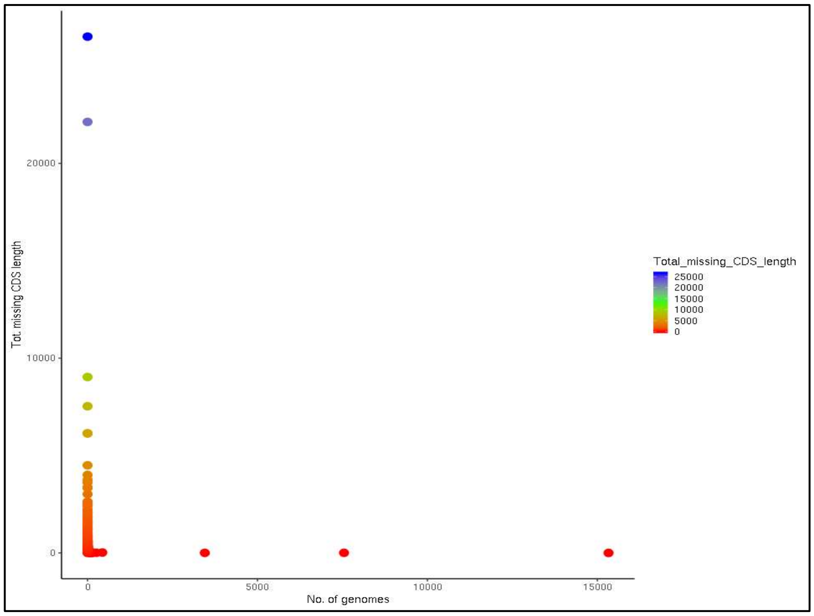

Output Image: HeatmapClusterOn.png

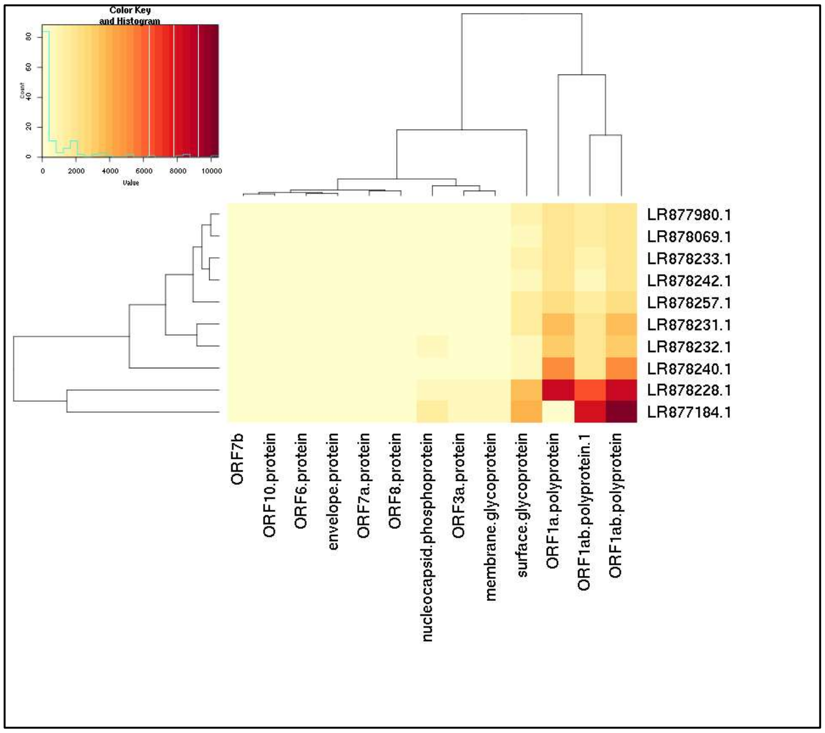

One can see from the above image, out of more than 30k genomes, 10 genomes were identified with the largest deletions in comparison with the reference. Subsequently one can go through the Stacked Bar chart to see the top affected genomes.

Output Image: StackedBar.png

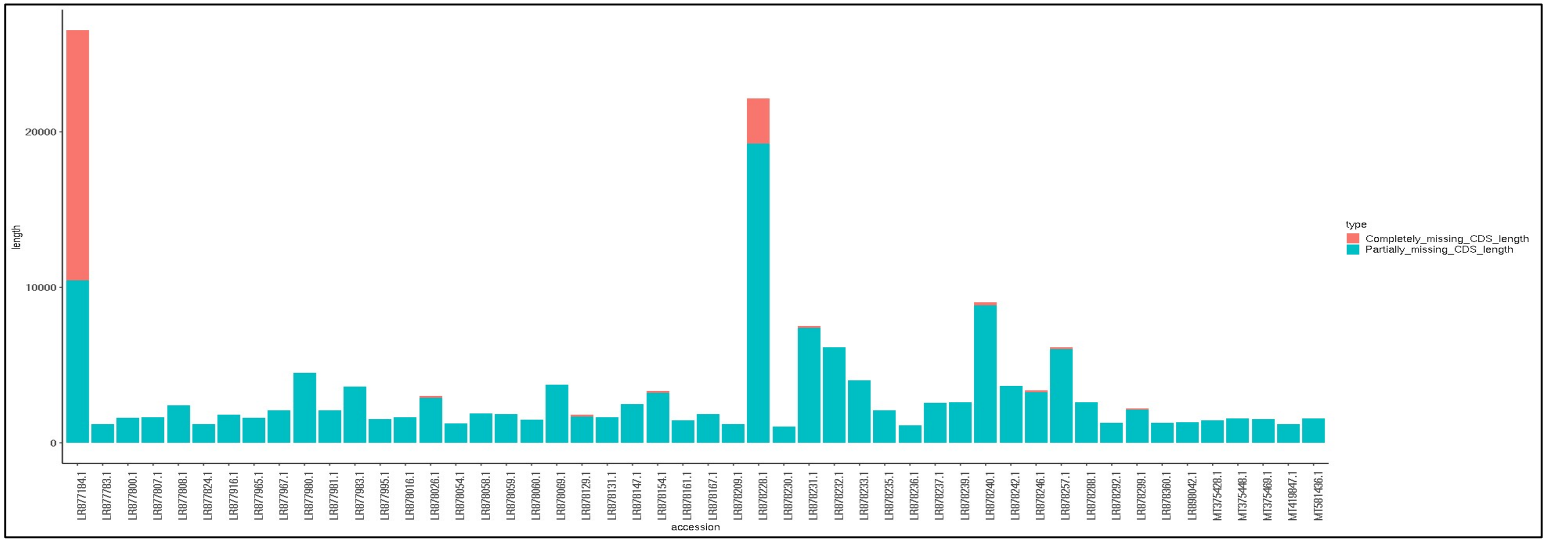

To see the top 20 genomes in the heatmap instead of the default 10, it can be run with following command

```
bash batch-run-mrf-wrapper.sh -d coronaGenomes -r NC_045512.2.fna -f NC_045512.gff3 -m 15 -o SARS2
```

But since the above command will run MRF for all the genomes again, one can directly run the perl parser command as below

```
perl parse_mrf_output.pl --top 20
```

This will recreate the cdsHeatmap.txt file again by fetching the top 20 affected genomes.To see this as a heatmap, run the following command.

```
Rscript genPlots.R
```

The above command will generate the plots again by using the new cdsHeatmap.txt file.

Output Image: HeatmapClusterOn.png

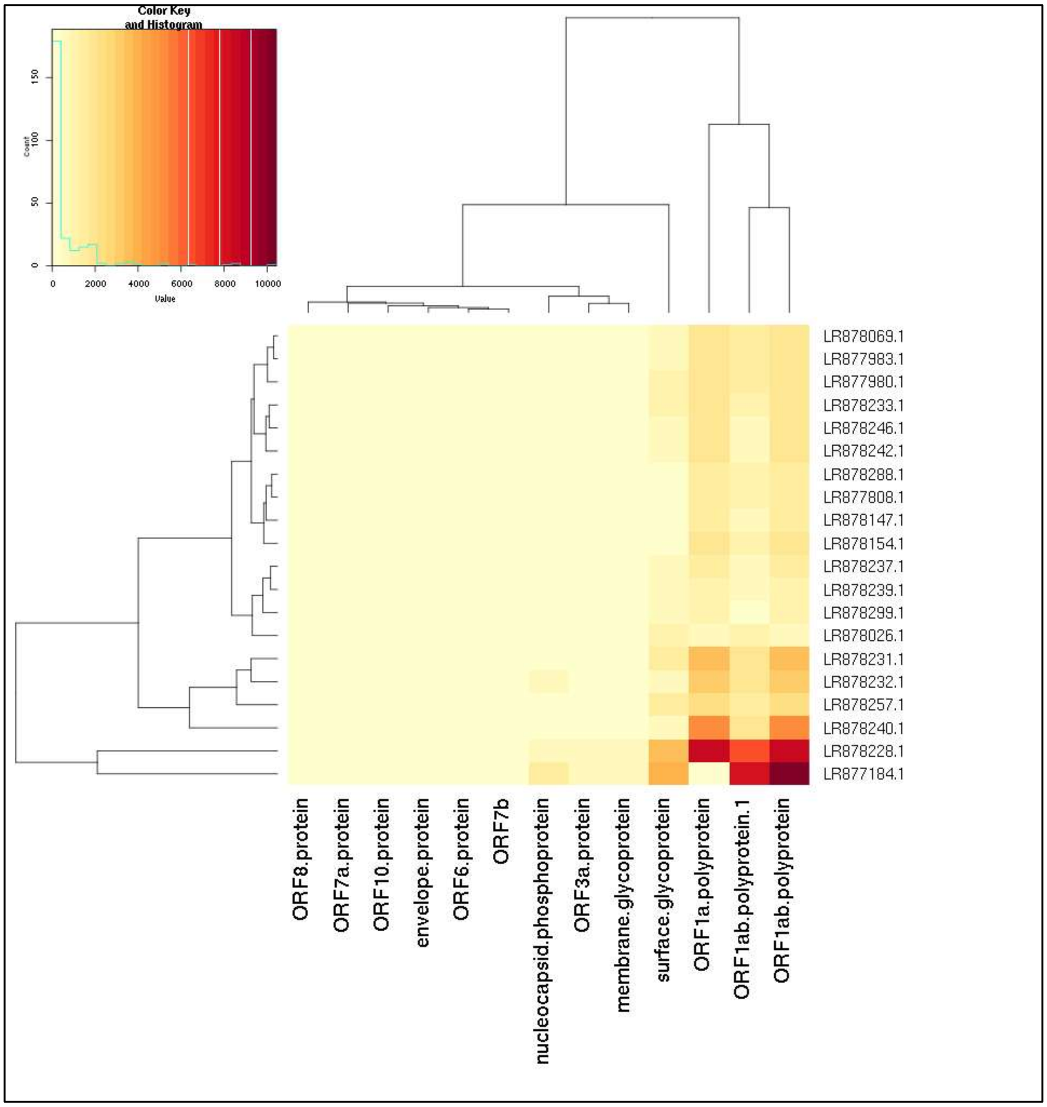

Similarily, to show more than the default 50 genomes in the StackedBar chart, run the Rscript as below

```
Rscript genPlots.R 100
```

Output Image: StackedBar.png

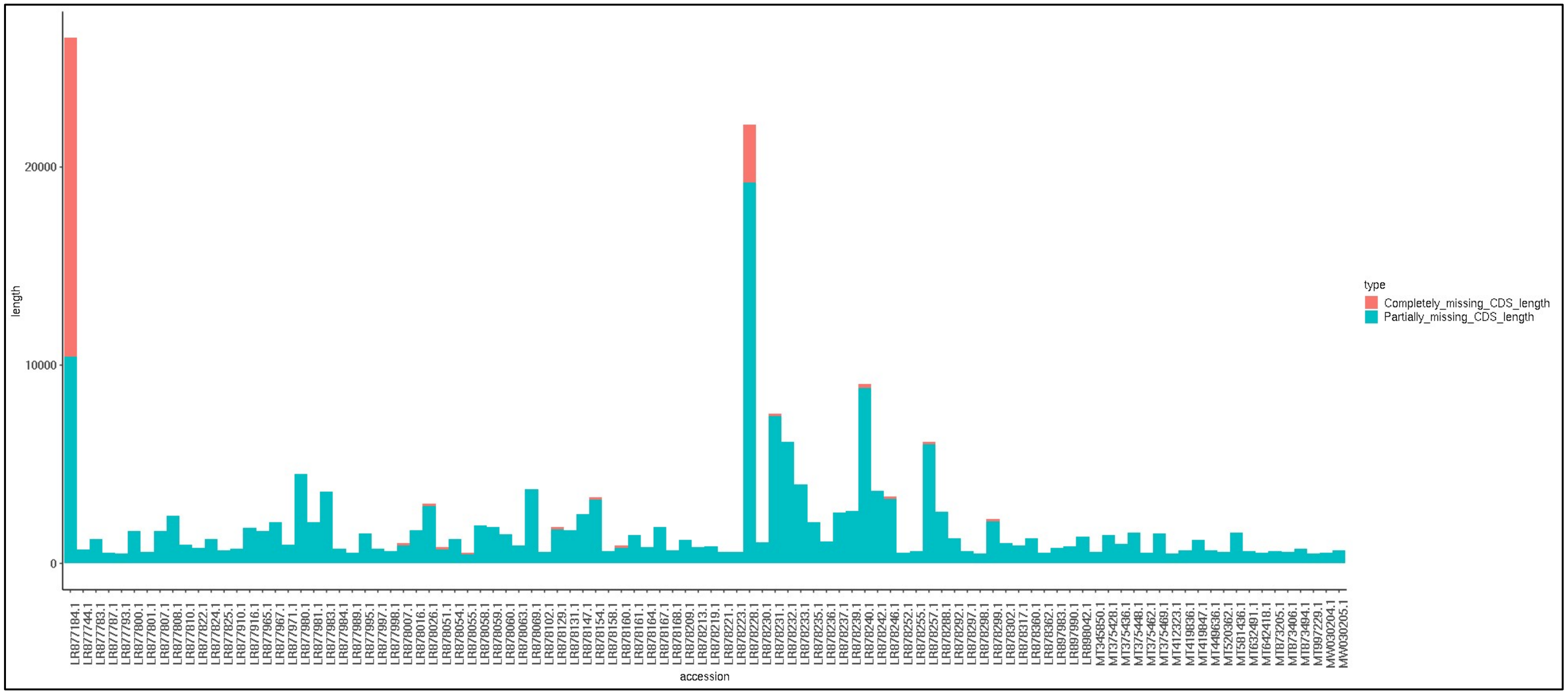

8.4.2 Use case: ASFV

To demonstrate the utility of MRF-batch mode in quickly listing the key proteins that differ in virulent and vaccine strain, 17 isolates of African Swine Fever Virus are compared against the virulent isolate of AFSV, Benin 97/1 (Accession no. AM712239). The list of accessions is given in table 4.

Table 4. List of ASFV genome accession used for batch mode analysis of MRF

S.No	Accession number	genome size	Strain	Country
1	FN557520	181187	E75	Spain
2	KM102979	182906	isolate 26544/OG10	Italy
3	KM111295	184368	Ken06.Bus	Kenya
4	KM111294	191058	Ken05/Tk1	Kenya
5	KM262845	172051	NHV	Portugal
6	KM262844	182362	L60	Portugal
7	U18466	170101	BA71V	Spain
8	FR682468	189344	Georgia 2007/1	Georgia
9	KP843857	189333	Odintsovo_02/14	Russia
10	AM712240	171719	OURT 88/3	Portugal
11	AM712239	182284	Benin 97/1	Benin
12	AY261366	186528	Warthog	Namibia
13	AY261365	190773	Warmbaths	South Africa
14	AY261364	185689	Tengani 62	Malawi
15	AY261363	190324	Pretorisuskop/96/4	South Africa
16	AY261362	192714	Mkuzi 1979	South Africa
17	AY261361	187612	Malawi Lil-20/1	Malawi
18	AY261360	193886	Kenya 1950	Kenya

The query directory contains the below fasta files

```
26544OG10.fasta          E75.fasta                      Ken05.fasta
Kenya_1950.fasta         Malawi_Lil-201(1983).fasta     NHV.fasta
OURT_883.fasta           Tengani_62.fasta               Warthog.fasta
BA71qqV.fasta            georgia_2007.fasta             Ken06.fasta
L60.fasta                Mkuzi_1979.fasta               Odintsovo.fasta
Pretorisuskop964.fasta   Warmbaths.fasta
```

The below command is run to analyze and visualize all the genomes in the query directory

```
bash batch-run-mrf-wrapper.sh -d query -r Benin-971.fasta -f \
Benin-971.gff3 -o ASFV -U 17
```

Output Image: StackedBar.png

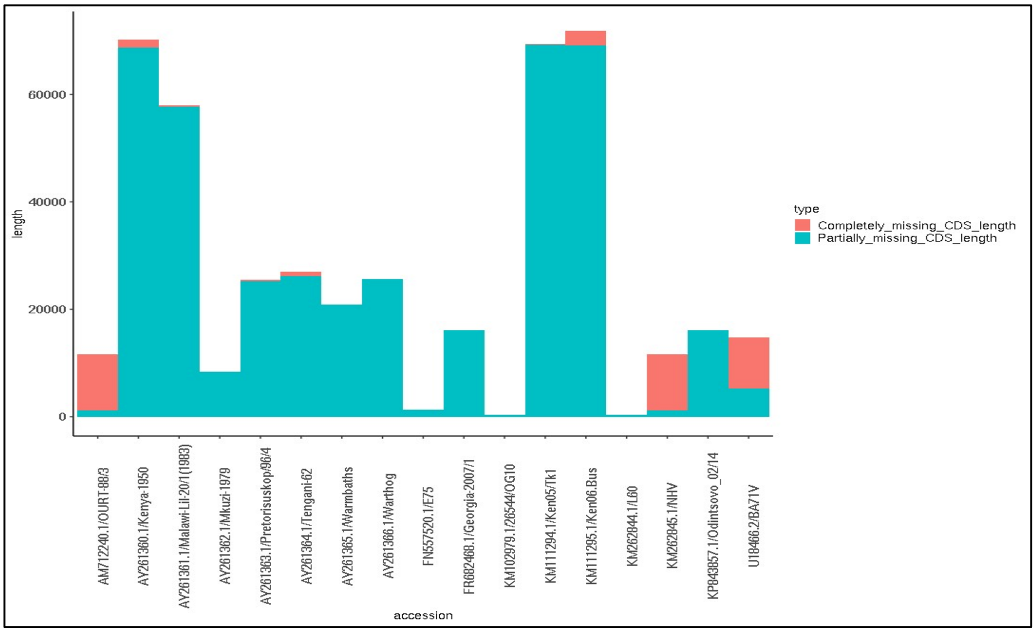

The above stacked bar depicts the overview of deletions in the genomes of ASFV

Output File: cdsHeatmap.txt

protein_name	MGF-360L-10L	MGF-505-1R	MGF-360-12L	MGF-360-13L	MGF-505-2R	MGF-505-4R	MGF-505-6R	MGF-505-7R	Ribonucleotide-reductase-large-subunit	Helicase	RNA-polymerase-subunit-2	CD2-homolog	pB602L	DNA-polymerase	220kDa-Polyprotein
KM111295.1/ Ken06.Bus	960	1021	575	596	936	984	1026	1357	1293	1303	1317	968	722	1226	3913
AY261360.1/ Kenya-1950	964	992	646	657	924	928	1104	1394	1351	1429	1234	955	715	1246	3483
KM111294.1/ Ken05/Tk1	986	963	627	631	790	918	1160	1373	1333	1314	1362	961	751	1162	3486
AY261361.1/ Malawi-Lil-20/1(1983)	588	722	511	628	769	964	990	1267	1259	1221	1198	961	668	954	2869
AY261364.1/ Tengani-62	506	472	212	224	112	880	769	202	561	476	514	768	460	445	1269
AY261366.1/ Warthog	324	519	275	253	243	784	725	219	637	367	420	869	508	451	1541
AY261363.1/ Pretorisuskop/96/4	298	578	288	344	226	847	478	291	587	382	380	793	474	488	1796
AY261365.1/ Warmbaths	384	466	268	292	203	646	767	192	467	411	452	768	487	500	445
KP843857.1/ Odintsovo_02/14	440	445	245	122	64	477	649	175	549	318	359	875	438	280	2352
FR682468.1/ Georgia-2007/1	440	445	245	122	64	477	649	175	549	318	359	875	430	280	2352
U18466.2/ BA71V	1071	1596	1053	1062	0	0	612	910	0	0	0	0	289	0	0
AM712240.1/ OURT-88/3	1071	1596	1053	1062	1581	0	0	0	0	0	0	0	289	0	0
KM262845.1/ NHV	1071	1596	1053	1062	1581	0	0	0	0	0	0	0	289	0	0
AY261362.1/ Mkuzi-1979	17	405	210	0	0	4	362	170	270	134	209	698	438	93	524
FN557520.1/ E75	0	0	0	0	0	0	0	0	0	3	0	0	289	0	0
KM262844.1/ L60	0	0	0	0	0	0	0	0	0	0	0	82	234	0	0
KM102979.1/ 26544/OG10	0	0	0	0	0	0	0	0	0	0	0	0	290	0	0

Output Image: HeatmapClusterOn.png

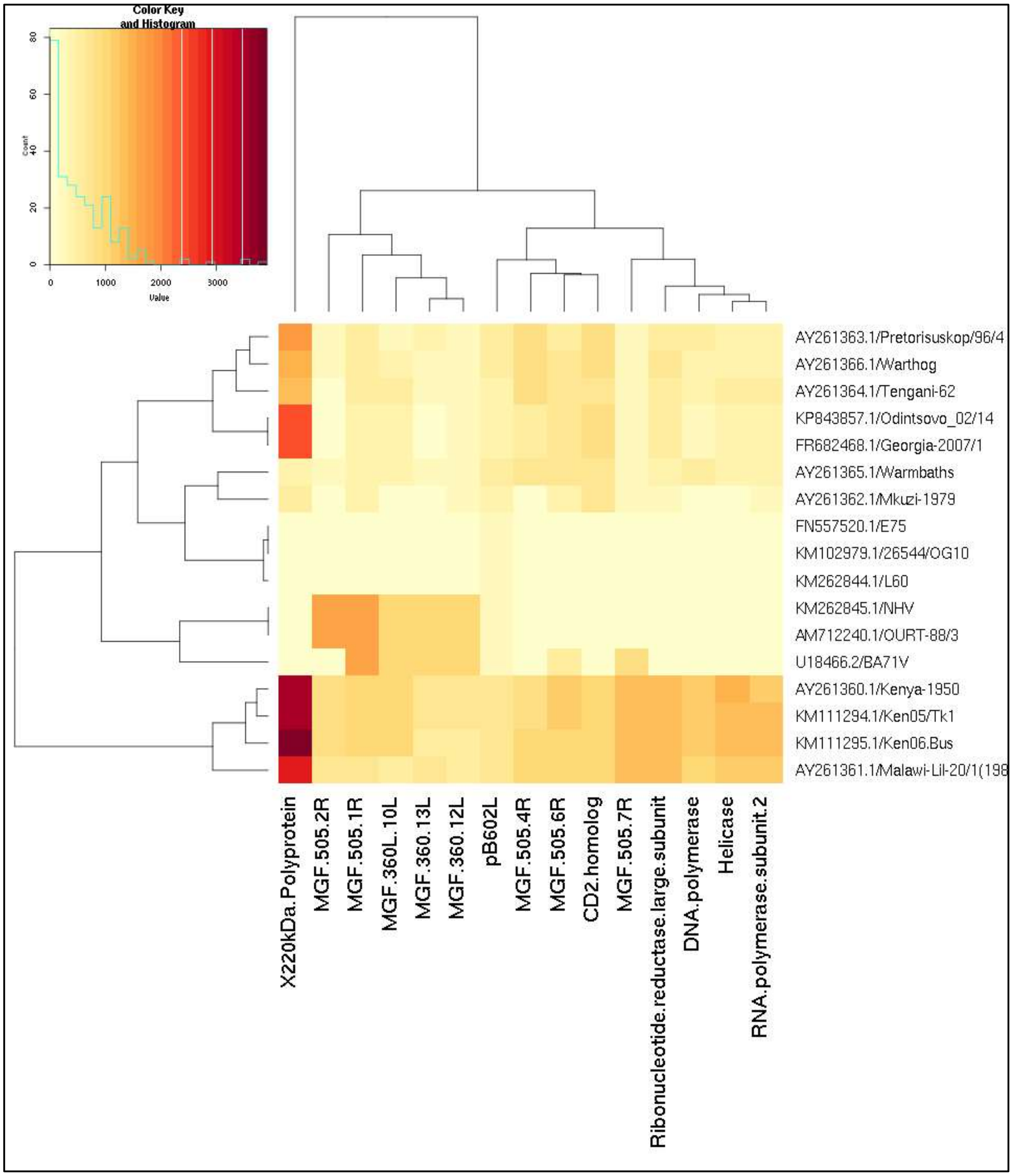

Other than the 220kDa protein, it is observed that the MGF family of proteins have suffered a lot of deletions. To see the variation in those proteins, we select the ones of MGF family listed in the above image and run the following command.

```
batch-run-mrf-wrapper.sh -d query -r Benin-971.fasta -f Benin-971.gff3 \
-o ASFV -N MGF-360L-10L,MGF-360-11L,MGF-360-12L,MGF-360-13L,MGF-360-14L,\
MGF-505-1R,MGF-505 -2R,MGF-505-3R -U 17
```

Output File: cdsHeatmap.txt

protein_name	MGF-360L-10L	MGF-360-11L	MGF-360-12L	MGF-360-13L	MGF-360-14L	MGF-505-1R	MGF-505-2R	MGF-505-3R
**KM111295.1/Ken06.Bus**	960	757	575	596	559	1021	936	561
**AY261360.1/Kenya-1950**	964	772	646	657	460	992	924	607
**KM111294.1/Ken05/Tk1**	986	789	627	631	541	963	790	591
**AY261361.1/Malawi-Lil-20/1(1983)**	588	702	511	628	540	722	769	432
**AY261364.1/Tengani-62**	506	121	212	224	234	472	112	257
**AY261366.1/Warthog**	324	150	275	253	151	519	243	267
**AY261363.1/Pretorisuskop/96/4**	298	162	288	344	183	578	226	220
**AY261365.1/Warmbaths**	384	161	268	292	273	466	203	207
**KP843857.1/Odintsovo_02/14**	440	97	245	122	10	445	64	157
**FR682468.1/Georgia-2007/1**	440	97	245	122	10	445	64	157
**U18466.2/BA71V**	1071	1062	1053	1062	1050	1596	0	0
**KM262845.1/NHV**	1071	1062	1053	1062	1074	1596	1581	509
**AM712240.1/OURT-88/3**	1071	1062	1053	1062	1074	1596	1581	509
**AY261362.1/Mkuzi-1979**	17	21	210	0	0	405	0	39
**FN557520.1/E75**	0	0	0	0	0	0	0	0
**KM262844.1/L60**	0	0	0	0	0	0	0	0
**KM102979.1/26544/OG10**	0	0	0	0	0	0	0	0

Output Image: HeatmapClusterOn.png

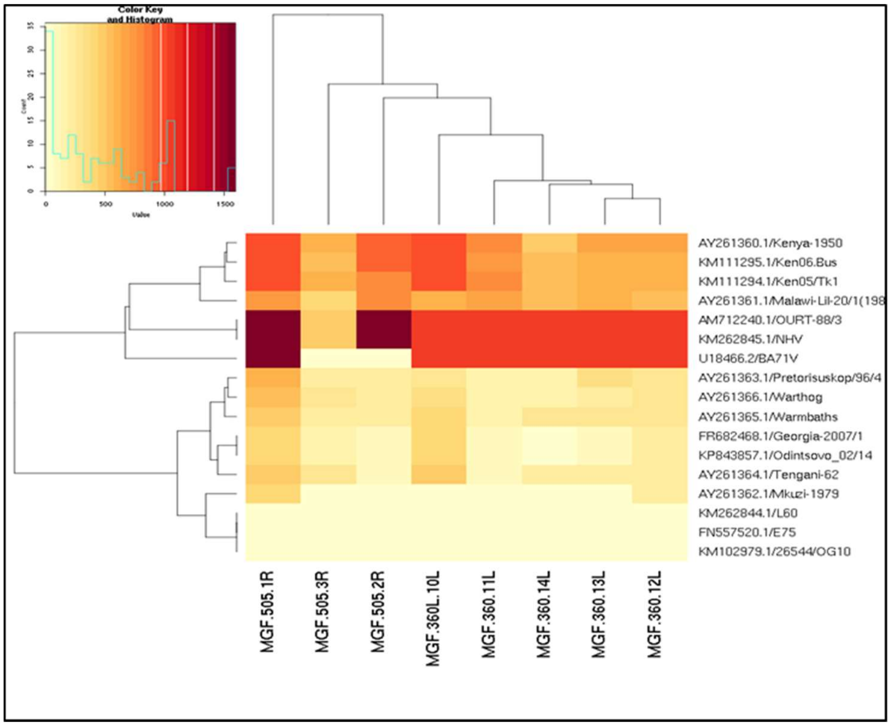

9. Conclusion

The MRF tool is a fast and reliable tool to identify and visualize the genomic differences between a pair of virus genomes. The tool produces two important output files, missing regions and missing coding sequences in a tabular format. In addition, a publication-friendly and downloadable graphical output also would be produced by MRF. The tool is robust as it is built on top of the exact match algorithm and also flexible as it allows users to screen the exact matches and optimize the parameters to obtain the best results. At present it has the capability of handling both DNA viruses and RNA viruses and small genomes of organelles. Complex genomes with recombination are currently not handled well by the tool but the future versions will be designed to handle them.

APPENDIX – I (*Motivation for building MRF*)

In virus genomics research, it is very common to compare genome sequences of two or more virus isolates/strains in order to understand the similarities and differences between them. Currently, one of the most common practices in comparative genomics is to perform a BLAST search for one query genome against a reference genome to gain insights. Mostly, researchers involved in virus genomics studies would be interested to know the coding sequences that are gained or lost in certain isolates in comparison to others. Towards this goal, how MRF has potential to supplement the output of blast search is explained in this Appendix. The genome of White Spot Syndrome Virus (WSSV) was taken to explain the contrast between blast and MRF.

Different genome accessions of WSSV vary in genome length ranging from 280 Kb to 305 Kb. Let us compare two genomes of WSSV, KX686117 (300,223 bp) and AF332093 (305,119 bp). A nucleotide blast (blastn) search was performed for KX686117 against a well annotated AF332093 with the following parameters.

Search Parameters	
Program	blastn
Word size	28
Expect value	10
Hitlist size	100
Match/Mismatch scores	1,-2
Gapcosts	0,2.5
Low Complexity Filter	Yes
Filter string	L;m;
Genetic Code	1

Following is the output of *blastn* search. It is apparent that despite the obvious genome size difference of around 5kb between the two genomes, the result is, a near exact match with 100% query coverage and with near 100% identity.

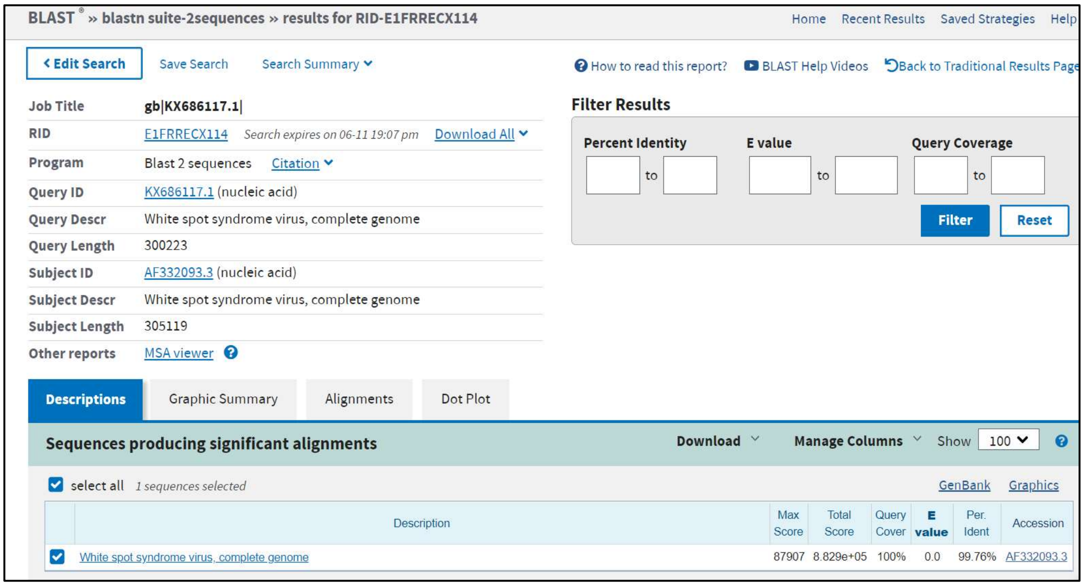

The results infer that the genomes are very identical and there is not much difference between them. But it does not account for the difference in genome length at least not in a straight forward way. It is further understood from the alignments tab that the results are supported by 1242 alignments and each alignment has its individual percentage identity and query coverage as shown in the snapshots given below.

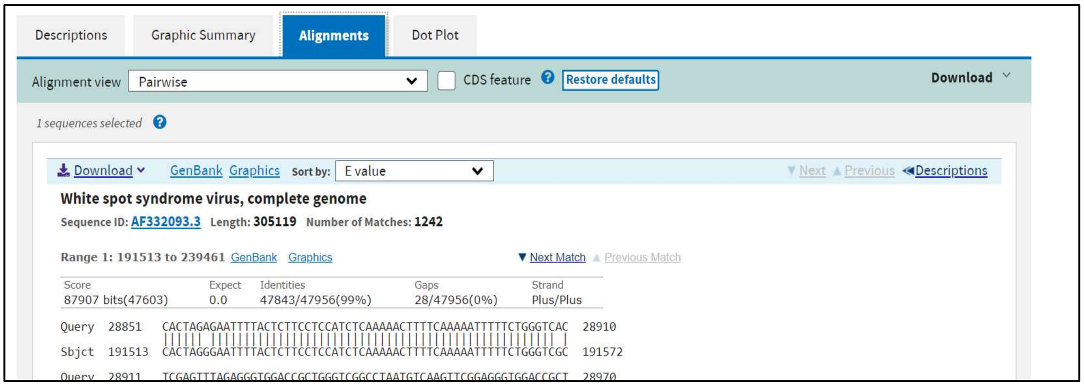


```
# blastn
# Iteration: 0
# Query: KX686117.1 White spot syndrome virus, complete genome
# RID: E1FRRECX114
# Database: n/a
# Fields: query acc.ver, subject acc.ver, % identity, alignment length, mismatches, gap opens, q. start, q. end, s. start, s. end, evalue, bit score
# 1242 hits found
KX686117.1	AF332093.3	99.764	47956	85	13	28851	76785	191513	239461	0.0	87907
KX686117.1	AF332093.3	99.933	31481	16	3	77893	109369	240253	271732	0.0	58013
KX686117.1	AF332093.3	99.869	29732	10	8	137442	167161	1	29715	0.0	54661
KX686117.1	AF332093.3	99.833	29361	37	12	249882	279239	112231	141582	0.0	53938
KX686117.1	AF332093.3	99.798	29202	37	3	327	29507	163476	192676	0.0	53579
KX686117.1	AF332093.3	99.789	20877	13	11	170277	191146	31992	52844	0.0	38280
KX686117.1	AF332093.3	99.912	19251	8	5	280978	300223	143927	163173	0.0	35447
KX686117.1	AF332093.3	99.952	14674	4	2	122768	137441	290449	305119	0.0	27056
KX686117.1	AF332093.3	99.356	14914	62	25	220501	235404	82277	97166	0.0	26978
KX686117.1	AF332093.3	99.717	14113	30	5	236575	250687	98678	112780	0.0	25832
KX686117.1	AF332093.3	99.687	13417	33	7	109358	122770	272310	285721	0.0	24535
KX686117.1	AF332093.3	99.886	11412	6	3	191094	202498	52931	64342	0.0	20995
KX686117.1	AF332093.3	99.307	11108	53	18	201777	212872	63813	74908	0.0	20064
KX686117.1	AF332093.3	99.114	8355	47	24	212828	221170	75110	83449	0.0	14994
KX686117.1	AF332093.3	98.943	2366	21	2	278807	281168	141404	143769	0.0	4228
KX686117.1	AF332093.3	97.372	2169	45	12	168615	170777	30643	32805	0.0	3679
KX686117.1	AF332093.3	98.638	1982	13	9	166816	168790	29074	31048	0.0	3498
```

So, the quest to map the base coordinates for the 5kb sequence (reason for length difference between genomes) in the larger genome has becomes a cumbersome exercise as one has to manually go through all the alignments considering the percentage identity and query coverage for each alignment. Added to this, the circular genomes pose further complication as these genomes may not have uniform start positions in linear sequence accession.

This situation might get better by re-visiting the objective. Instead of trying to identify the 5kb portion in the larger genome and understand its significance, the blast search can be modified to find if any CDS in the larger genome is lost in the smaller genome. The modified objective can be met by performing a *blastn* search for all the coding sequences (CDS) of the larger genome (AF332093) against the smaller genome (KX686117) and identify if any CDS does not have a match. Even though this seems simple, there may be certain problems, which are listed below.

1. The blast output table contains the similarity hits that are labelled by sequence identifiers (accession number in most cases) only. You may also browse hits with CDS nomenclature, but one hit only at a time. In genomes with several CDS, it is not convenient to track all of them individually. This situation warrants pre-processing of CDS fasta in such way to identify all the matches using CDS names.
2. It is imperative that each match has query coverage listed along with it, as it gives information about how much of the query is actually having a match. But the hit table available from the Blast website does not have this information and one has to go through each match separately to fetch query coverage. This problem may be mitigated by performing *blastn* search through command line using *-OUTFMT* 6 option.

   Therefore, we can affirmatively conclude that with blast or any other similarity search tools, one cannot find the missing CDS and their base coordinates in one go. In case of partially missing CDS, knowledge of missing base coordinates is highly valuable. This gap is filled with MRF. The MRF compares two genomes, one as query and the other as reference genome. The users need to give gff3 file of the reference genome also as input. Then MRF can tabulate and depict the complete and partially missing CDS in the query genome as per the CDS nomenclature of the reference genome. Therefore, users have the opportunity to compare several query genomes to a single reference genome and build a deletion profile.

Table A1. Features that contrast blast and MRF.

Blast	MRF
Blast uses heuristics to search for the subsequences of defined word length and extends them with gaps and mismatches.	MRF utilizes the exact match algorithm of mummer to find perfect matches of defined mum length or more and extracts the missing regions and deleted coding sequences from the mummer output.
Blast output is a list of matches along with its statistics. The CDS that do not have a hit and partial hit have to be manually extracted.	The output of MRF is a table of complete and partially deleted coding sequences. The CDS that have perfect hits can be obtained from the 'completely present

	coding sequences' file and can be further confirmed through the 'generate missing regions' option
Since blast is optimized to find matches, it is able to span across the regions with poor identity	Works well when the sequences have high similarity by reporting unambiguous matches.
Reducing the word length increases the possibility of finding a match, but this leads to too many matches and sometimes with very weak alignments.	Reducing the mum length increases the possibility of finding a match, however this may result in many false matches but does not lead to an overall false alignment as each match is perfect and is not extended

The following table compares the some of the complimentary features of Blast and MRF

Table A2. Contrast between the commonly used blast-based searches and MRF.

Feature	Simple blast-based search	MRF
**Initial seed to initiate alignment**	Word length	Mum length or exact match length
**Can compare two genomes**	Yes. One genome as reference and the other as query.	Yes. One genome as reference and the other as query.
**Output includes list of missing genomic regions**	No	Yes
**Output includes the list of completely deleted CDS in query genome**	No. However, the coding sequences without blast hits can be taken as missing in query.	Yes
**Can infer partially missing coding sequences in query**	No	Yes. The partially missing coding sequences in query are tabulated.
**Prints total number of bases deleted in query genome**	No	Yes
**Can take both genome sequence and annotation as input**	No. Can take either total genome or coding sequences in fasta file as input. Only 2 files as input, reference sequence and query	Yes. Takes both sequence and annotation as input. Takes 3 files as input, reference sequence, query sequence and .gff3 file of reference genome.

Few notes on MRF output

1. The primary output of MRF is *missing genomic regions*, which are the base coordinates of genomic regions (of reference) that do not have a match with query genome. Here, we conclude these regions as missing in the query genome. These *missing genomic regions* are generally true in most cases except for genomes which have not lost the region but have undergone too many mutations. Sometimes, a genomic region might vary between query and reference genomes due to several point mutations but without deletions. In this scenario, a perfect match could not be established and the genomic region might end up in '*missing genomic regions*' list.
2. The output of 'completely missing coding sequences' always indicate the CDS that are lost in the query genome. There might be exceptions while handling highly mutable genomes. Sometimes, there might be a case of partial deletion only. But if the existing partial sequence harbors too many mutations leading to non-detection of exact matches, then a 'partially missing CDS' might be tabulated under 'completely missing CDS'.
3. Again in case of output indicating 'partially missing CDS', the missing lengths might be inflated in case of highly mutable genomes.
4. MRF has three levels of safety to avoid problems related to highly mutable genomes as explained in points 2 and 3.
    a. Point mutations: Single base deletions have been excluded from 'missing length' calculations as these are actually mutated sites in most cases. These point mutations are still displayed in *missing genomic regions* output table (Non_hit_region = X). Indirectly, this could be a summary of mutation sites between analyzed genomes.
    b. Exact match length: Users have the provision to choose their own 'exact match length' before submitting a job to MRF. While handling highly mutable genomes, users can reduce 'exact match length' from the default value, 20. This avoids reporting of highly mutated regions as missing because exact matches of shorter lengths could be made between mutated sites.
    c. Negative and positive offset: Running jobs with shorter 'exact match length' might lead to false matches at random positions. The negative and positive offset parameters safeguard against false matches. Assume that we define, negative offset = 2 and positive offset =2. Then MRF checks the 2 exact match upstream (negative offset) and downstream (positive offset) to the current exact match for the base coordinates representing sequence contiguity of the query genome. If base coordinates are not contiguous then the current exact match would be treated as false match.
5. MRF cannot detect mutated genomic regions shorter than 'exact match length' because the mutated sites prevent detection of exact matches between query and reference genomes.

APPENDIX – II (*Benchmarking studies*)

This sections describes as how MRF complements and differs with Blast in terms of ease of use, precise identification of deletions and summarization. The blast search is the most commonly used similarity-search tool in comparative genomics. Therefore it would be ideal to compare the results provided by MRF with that of blast search. This Appendix section provides a detailed account of the relative merits and demerits between MRF and blast search utilizing a few virus genomes as use cases.

In addition to comparison with Blast, a few case studies have been included by analyzing multiple isolates of WSSV, multiple strains of AFSV and several strains of HIV-1. These studies help to identify 'deletion hotspots', regions which are prone to frequent deletions and mutations. In addition to that, it is also possible to identify genes which are crucial to virulence or fitness of a particular strain when compared to vaccine or attenuated strain.

A2.1 USE CASE 1: White Spot Syndrome Virus (highly-similar but length-varying genomes)

Genome accessions: KX686117 (300,223 bp) and AF332093 (305,119 bp).

A2.1.1 Benchmarking MRF with Blast

A2.1.1.1 NCBI Blast

Query: coding sequences of AF332093 genome
Subject: KX686117 genome

Search Parameters	
Program	blastn
Word size	28
Expect value	10
Hitlist size	100
Match/Mismatch scores	1,-2
Gapcosts	0,2.5
Low Complexity Filter	Yes
Filter string	L;m;
Genetic Code	1

The WSSV genome is the largest among animal viruses. The genome of AF332093 isolate has 524 coding sequences. To obtain the missing CDS in KX686117, the hit table of Blast output containing all 524 sequences has to be evaluated for their Hit Status, Percentage identity and query coverage. These statistics for the coding sequences with no blast hit and sequences with less than 50% query coverage are consolidated from the hit table and presented below in Table A3.

Table A3: The statistics for the coding sequences with no blast hit and sequences with less than 50% query coverage

CDS name	Percentage identity (%)	Query Coverage (%)
wsv095	No hit	
wsv164	No hit	
wsv462	No hit	
wsv463	No hit	
wsv489	No hit	
wsv490	No hit	
wsv492	No hit	
wsv493	No hit	
wsv494	No hit	
wsv495	No hit	
wsv496	No hit	
wsv497	No hit	
wsv498	No hit	
wsv499	No hit	
wsv461	99.46	39

A2.1.1.2 MRF

Query: KX686117 genome [300223 bp]
Reference: AF332093 genome [305119 bp]
The input options for MRF are shown below in screenshot.

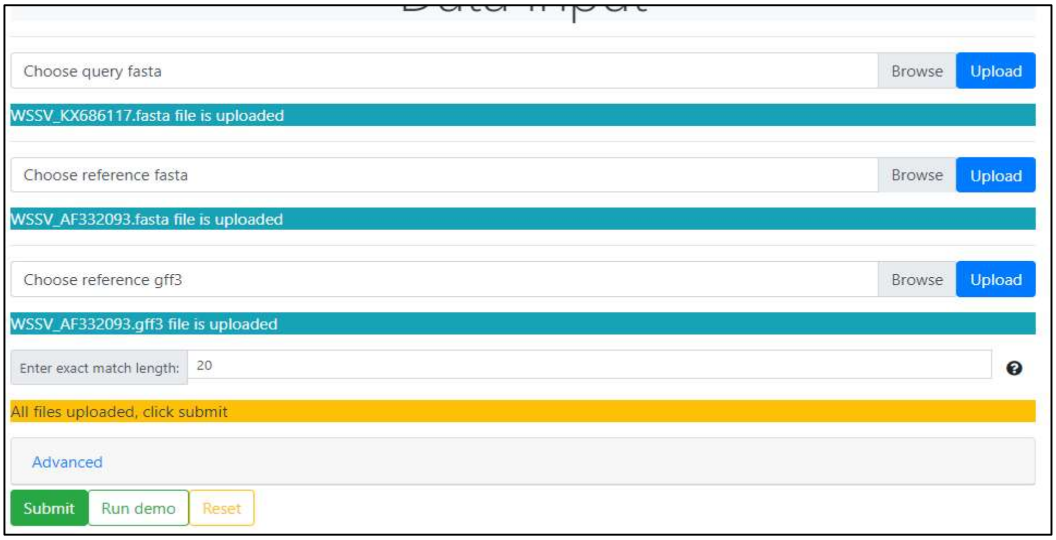

The tabulated output from MRF indicating the complete and partial (>5% lost) missing coding sequences has been given in Table A4 and A5 respectively.

Table A4: Completely missing coding sequences

Missing Region Start	Missing Region End	CDS Start	CDS End	Missing Region Length	CDS Product	Protein id
271729	272317	272021	272272	252	wsv462	AAL33463.1
271729	272317	272046	272276	231	wsv463	AAL33464.1
285722	290448	285750	286487	738	wsv489	AAL33490.1
285722	290448	286530	286868	339	wsv490	AIX03692.1
285722	290448	286797	287207	411	wsv492	AIX03693.1
285722	290448	287295	287981	687	wsv493	AAL33494.1
285722	290448	287584	287775	192	wsv494	AAL33495.1
285722	290448	288375	288635	261	wsv495	AAL33496.1
285722	290448	288693	288890	198	wsv496	AAL33497.1
285722	290448	288697	290394	1698	wsv497	AAL33498.1
285722	290448	288710	289015	306	wsv498	AAL33499.1
285722	290448	289034	289300	267	wsv499	AAL33500.1

Table A5: Partially missing coding sequences

Missing Region Start	Missing Region End	Missing Region Length	CDS Length	CDS Start	CDS End	Missing CDS Proportion(%)	CDS Product	Protein id
64343	64508	166	1113	63556	64668	14.91	wsv128	AAL33132.1
64343	64508	166	1074	63564	64637	15.46	wsv129	AAL33133.1
82386	82797	84	414	82056	82469	20.29	wsv149a	AIX03667.1
82386	82797	412	600	82375	82974	68.67	wsv150	AIX03668.1
98150	98677	528	909	97838	98746	58.09	wsv178	AAL33182.1
98150	98677	528	666	98115	98780	79.28	wsv179	AAL33183.1
112028	112094	67	540	111761	112300	12.41	wsv203	AAL33207.1
112148	112230	83	540	111761	112300	15.37	wsv203	AAL33207.1
112148	112230	27	390	112204	112593	6.92	wsv204	AAL33208.1
112506	112532	27	390	112204	112593	6.92	wsv204	AAL33208.1
112506	112532	27	210	112412	112621	12.86	wsv205	AAL33209.1
143784	144012	229	2352	142216	144567	9.74	wsv249	AAL33252.1
143622	143658	37	561	143496	144056	6.6	wsv250	AAL33253.1
143784	144012	229	561	143496	144056	40.82	wsv250	AAL33253.1
271729	272317	294	474	271549	272022	62.03	wsv461	AAL33462.1
278157	278577	35	186	278006	278191	18.82	wsv475	AAL33476.1

A2.1.1.3 Comparison between Blast and MRF results

Table A6: Comparison table between Blast and MRF results

**CDS Name**	**Blast**		**MRF**
	**Query coverage (%)**	**Percentage identity (%)**	**% Lost (> 50)**
wsv095	No hit		0
wsv164	No hit		0
wsv150	100	98.18	68.67
wsv178	100	100	58.09
wsv179	100	100	79.28
wsv461	39	99.46	62.03
wsv462	No hit		100
wsv463	No hit		100
wsv489	No hit		100
wsv490	No hit		100
wsv492	No hit		100
wsv493	No hit		100
wsv494	No hit		100
wsv495	No hit		100
wsv496	No hit		100
wsv497	No hit		100
wsv498	No hit		100
wsv499	No hit		100

It is interesting to note and further probe the results obtained for wsv095 and wsv164. The blast search yielded no hits for these two CDS which means they were absent in subject genome (KX686117) whereas MRF reported no deletions in these two CDS in query genome (KX686117).

Let us examine the exact match coordinates for these two CDS in the 'genomic missing regions' table obtained with MRF.

wsv095 (48619 - 48939)

47372	48722	185667	187017
48718	48817	187010	187109
48804	49161	187105	187462

wsv164 (89897 - 90091)

88660	90080	226897	228317
90082	94882	228319	233119

The results from MRF clearly indicated exact matches for these 2 CDS between query and reference genomes. Therefore, we shall repeat blast search with less stringent conditions. For each of these two CDS, blast search was performed with the 'somewhat similar sequences' option, which runs with word size of 11.

```
CDS             48619..48939
                /codon_start=1
                /product="wsv095"
                /protein_id="AAL33099.1"
                /translation="MTETHQHRKKWRRRRKKNNNNSQKKKAMVMRTKKKNNNNNNNQK
                EKRRIRMQIVTATVIAAAAVVAAVAAVVVVAAVVAAAVVAVKMKLKRRKKRKYLPRFR
                RERG"
```

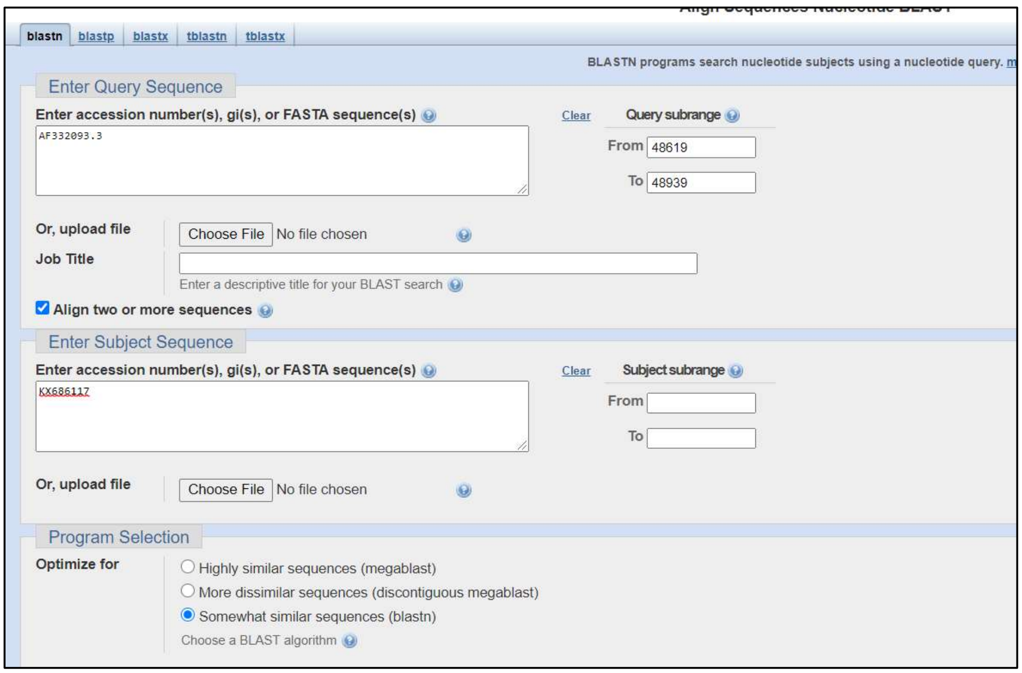

The changed parameters gave a hit for wsv095 in the subject genome with 100% query coverage as shown below.

Job Title	gb|AF332093.3|
RID	DMP6B3SN114 *Search expires on 06-06 22:38 pm* Download All
Program	Blast 2 sequences Citation
Query ID	AF332093.3 (nucleic acid)
Query Descr	White spot syndrome virus, complete genome
Query Length	321
Subject ID	KX686117.1 (nucleic acid)
Subject Descr	White spot syndrome virus, complete genome
Subject Length	300223
Other reports	MSA viewer

Filter Results

Percent Identity ___ to ___ E value ___ to ___ Query Coverage ___ to ___

Filter Reset

Descriptions | Graphic Summary | Alignments | Dot Plot

Sequences producing significant alignments Download Manage Columns Show 100

select all *1 sequences selected* GenBank Graphics

	Description	Max Score	Total Score	Query Cover	E value	Per. Ident	Accession
☑	White spot syndrome virus, complete genome	544	693	100%	1e-156	96.36%	KX686117.1

Following is the alignment for the hit region.

Download GenBank Graphics Sort by: E value

White spot syndrome virus, complete genome

Sequence ID: KX686117.1 Length: 300223 Number of Matches: 6

Range 1: 186914 to 187240 GenBank Graphics ▼ Next Match ▲ Previous Match

Score	Expect	Identities	Gaps	Strand
544 bits(602)	1e-156	318/330(96%)	12/330(3%)	Plus/Plus

```
Query  48619   ATGACGGAGACGCACCAGCacaggaagaaatggaggaggagaaggaagaagaacaacaac  48678
               ||||||||||||||||||||||||||||||||||||||||||||||||||||||||||||
Sbjct  186914  ATGACGGAGACGCACCAGCACAGGAAGAAATGGAGGAGGAGAAGGAAGAAGAACAACAAC  186973

Query  48679   aacagccagaagaagaaagcaatggtaatgagaaccaagaagaagaacaacaacaacaac  48738
               |||||||||||||||||||||||||||||||||||||||||||||   ||||||||||||
Sbjct  186974  AACAGCCAGAAGAAGAAAGCAATGGTAATGAGAACCAAGAAGAA---CAACAACAACAAC  187030

Query  48739   aacaacCAGAAAGAGAAGAGGAGAATAAGGATgcagatagtgacagcgacagtgatagca  48798
               ||||||||||||||||||||||||||||||||||||||||||||||||||||||||||||
Sbjct  187031  AACAACCAGAAAGAGAAGAGGAGAATAAGGATGCAGATAGTGACAGCGACAGTGATAGCA  187090

Query  48799   gcagc---------agcagtagtagcagcagtagcagcagtagtagtagtagcagcagta  48849
               |||||         ||||||||||||||||||||||||||||||||||||||||||||||
Sbjct  187091  GCAGCAGCAGTAGTAGCAGTAGTAGCAGCAGTAGCAGCAGTAGTAGTAGTAGCAGCAGTA  187150

Query  48850   gtagcagcagcagtagtagcagtgaaaatgaagctgaaaagaagaaagaagaggaagtac  48909
               ||||||||||||||||||||||||||||||||||||||||||||||||||||||||||||
Sbjct  187151  GTAGCAGCAGCAGTAGTAGCAGTGAAAATGAAGCTGAAAAGAAGAAAGAAGAGGAAGTAC  187210

Query  48910   ctgccaagattcagaagagaaagaGGCTAA  48939
               ||||||||||||||||||||||||||||||
Sbjct  187211  CTGCCAAGATTCAGAAGAGAAAGAGGCTAA  187240
```

Similarly, for wsv164 also, a hit was found in blast search.

```
                     [illegible]
CDS                  89897..90091
                     /codon_start=1
                     /product="wsv164"
                     /protein_id="AAL33168.1"
                     /translation="MFVFNILLFFLLSIFFFFLFTLFFLFFPLLFLPFPFLFPFLFFF
                     ITSSSSTTFLISVSFSLIIF"
CDS                  90213..90482
```

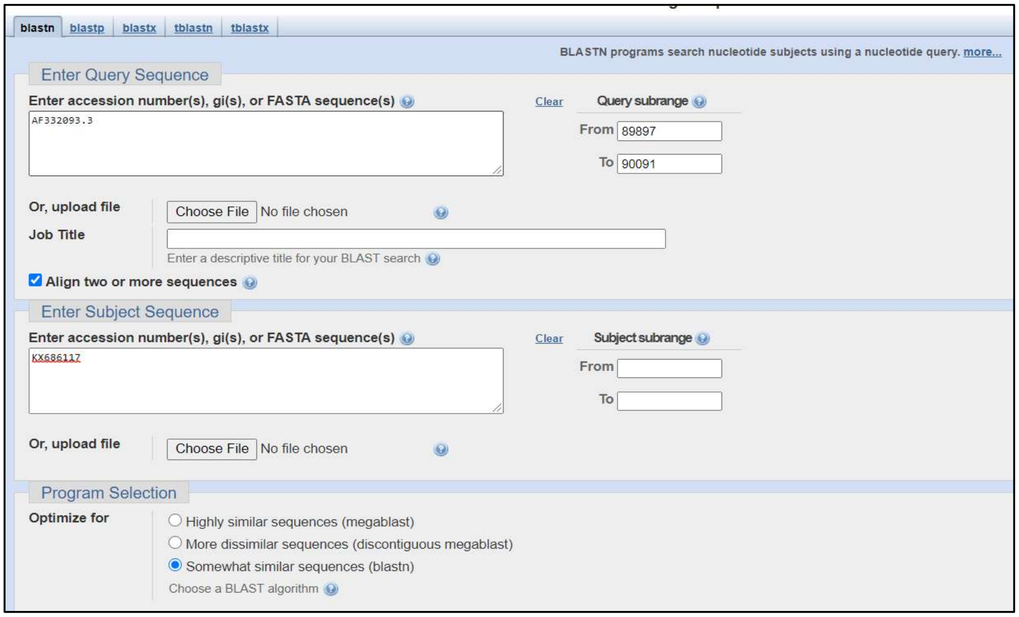

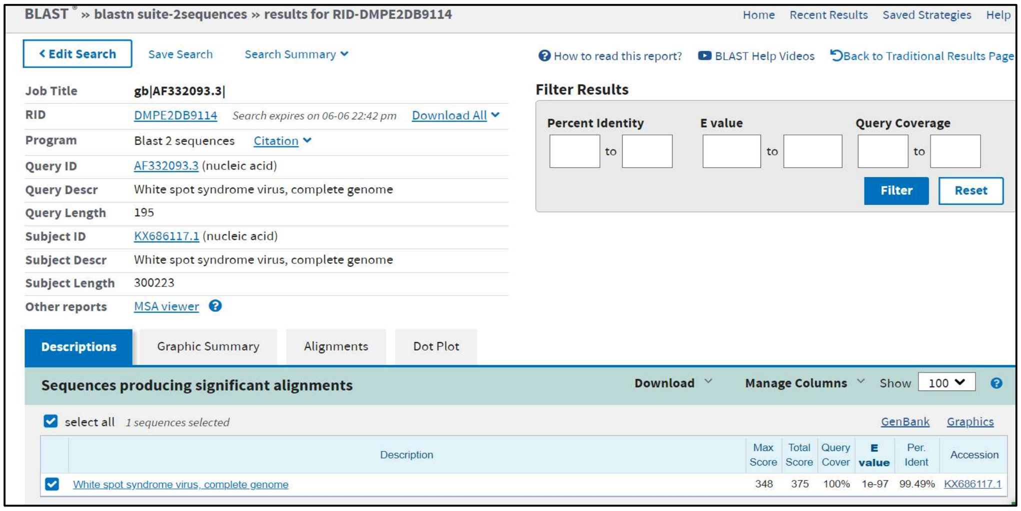

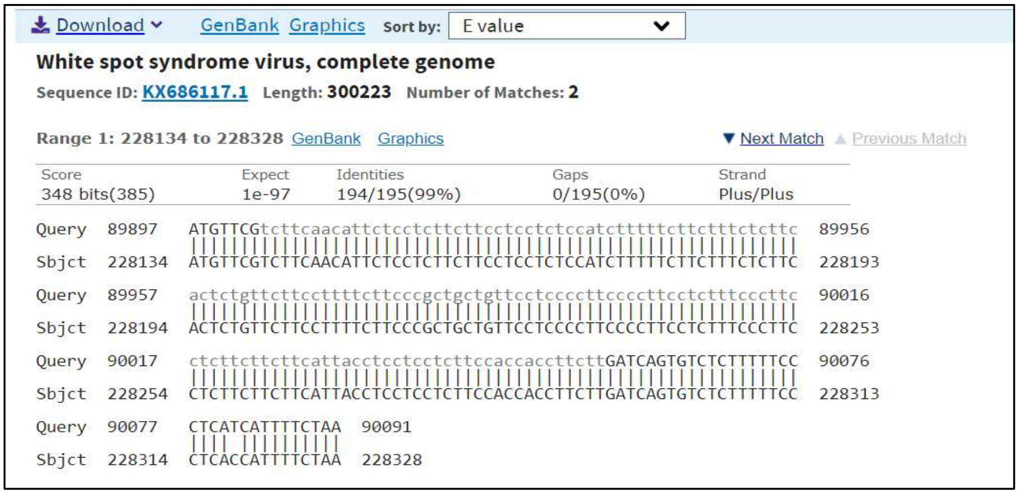

Reducing the word length from 28 to 11 has a drastic change in hit status, from 'no hit' to a hit with 100% query coverage and near 100% identity. When the

alignments were looked closely, there were even perfect matches greater than the length 28; however, they do not get reported as they were not considered as High Scoring Portions (HSPs). The alignments have low-complexity regions which blast generally indicates with lower-case nucleotides. The CDS with low complexity regions is not an issue while working MRF as it does not do any post processing for its matches and they are reported as it is.

wsv178

Now, let us consider the case of wsv178 for which blast reported a hit in subject genome with 100% similarity and 100% query coverage however MRF reported deletion of 58.09% of CDS length. Let us check the alignments printed during blast search for wsv178.

```
Range 2: 236575 to 236643 GenBank Graphics        ▼ Next Match ▲ Previous Match ▲ First Match

Score          Expect   Identities     Gaps       Strand
128 bits(69)   5e-31    69/69(100%)    0/69(0%)   Plus/Plus

Query  98678   CGCAAAAAGCGTGCCGCACCTCCACCTGAGGATGAAGAAGAGGATGATTTCTACCGCAAA  98737
               ||||||||||||||||||||||||||||||||||||||||||||||||||||||||||||
Sbjct  236575  CGCAAAAAGCGTGCCGCACCTCCACCTGAGGATGAAGAAGAGGATGATTTCTACCGCAAA  236634

Query  98738   AAGCGTTAA  98746
               |||||||||
Sbjct  236635  AAGCGTTAA  236643


Range 3: 236575 to 236640 GenBank Graphics        ▼ Next Match ▲ Previous Match ▲ First Match

Score          Expect   Identities     Gaps       Strand
122 bits(66)   3e-29    66/66(100%)    0/66(0%)   Plus/Plus

Query  98138   CGCAAAAAGCGTGCCGCACCTCCACCTGAGGATGAAGAAGAGGATGATTTCTACCGCAAA  98197
               ||||||||||||||||||||||||||||||||||||||||||||||||||||||||||||
Sbjct  236575  CGCAAAAAGCGTGCCGCACCTCCACCTGAGGATGAAGAAGAGGATGATTTCTACCGCAAA  236634

Query  98198   AAGCGT  98203
               ||||||
Sbjct  236635  AAGCGT  236640


Range 4: 236575 to 236640 GenBank Graphics        ▼ Next Match ▲ Previous Match ▲ First Match

Score          Expect   Identities     Gaps       Strand
122 bits(66)   3e-29    66/66(100%)    0/66(0%)   Plus/Plus

Query  98516   CGCAAAAAGCGTGCCGCACCTCCACCTGAGGATGAAGAAGAGGATGATTTCTACCGCAAA  98575
               ||||||||||||||||||||||||||||||||||||||||||||||||||||||||||||
Sbjct  236575  CGCAAAAAGCGTGCCGCACCTCCACCTGAGGATGAAGAAGAGGATGATTTCTACCGCAAA  236634

Query  98576   AAGCGT  98581
               ||||||
Sbjct  236635  AAGCGT  236640
```

```
Range 5: 236575 to 236640  GenBank  Graphics          ▼ Next Match  ▲ Previous Match  First Match

Score            Expect     Identities       Gaps         Strand
122 bits(66)     3e-29      66/66(100%)      0/66(0%)     Plus/Plus

Query  98570   CGCAAAAAGCGTGCCGCACCTCCACCTGAGGATGAAGAAGAGGATGATTTCTACCGCAAA  98629
               ||||||||||||||||||||||||||||||||||||||||||||||||||||||||||||
Sbjct  236575  CGCAAAAAGCGTGCCGCACCTCCACCTGAGGATGAAGAAGAGGATGATTTCTACCGCAAA  236634

Query  98630   AAGCGT  98635
               ||||||
Sbjct  236635  AAGCGT  236640


Range 6: 236575 to 236640  GenBank  Graphics          ▼ Next Match  ▲ Previous Match  First Match

Score            Expect     Identities       Gaps         Strand
122 bits(66)     3e-29      66/66(100%)      0/66(0%)     Plus/Plus

Query  98624   CGCAAAAAGCGTGCCGCACCTCCACCTGAGGATGAAGAAGAGGATGATTTCTACCGCAAA  98683
               ||||||||||||||||||||||||||||||||||||||||||||||||||||||||||||
Sbjct  236575  CGCAAAAAGCGTGCCGCACCTCCACCTGAGGATGAAGAAGAGGATGATTTCTACCGCAAA  236634

Query  98684   AAGCGT  98689
               ||||||
Sbjct  236635  AAGCGT  236640


Range 7: 236575 to 236640  GenBank  Graphics          ▼ Next Match  ▲ Previous Match  First Match

Score            Expect     Identities       Gaps         Strand
117 bits(63)     1e-27      65/66(98%)       0/66(0%)     Plus/Plus

Query  98192   CGCAAAAAGCGTGCCGCACCTCCACCTGAGGATGAAGAAGAGGATGAGTTCTACCGCAAA  98251
               ||||||||||||||||||||||||||||||||||||||||||||||| ||||||||||||
Sbjct  236575  CGCAAAAAGCGTGCCGCACCTCCACCTGAGGATGAAGAAGAGGATGATTTCTACCGCAAA  236634

Query  98252   AAGCGT  98257
               ||||||
Sbjct  236635  AAGCGT  236640
```

```
Range 8: 236575 to 236640  GenBank  Graphics          ▼ Next Match  ▲ Previous Match  First Match

Score            Expect     Identities       Gaps         Strand
117 bits(63)     1e-27      65/66(98%)       0/66(0%)     Plus/Plus

Query  98246   CGCAAAAAGCGTGCCGCACCTCCACCTGAGGATGAAGAAGAGGATGAGTTCTACCGCAAA  98305
               ||||||||||||||||||||||||||||||||||||||||||||||| ||||||||||||
Sbjct  236575  CGCAAAAAGCGTGCCGCACCTCCACCTGAGGATGAAGAAGAGGATGATTTCTACCGCAAA  236634

Query  98306   AAGCGT  98311
               ||||||
Sbjct  236635  AAGCGT  236640


Range 9: 236575 to 236640  GenBank  Graphics          ▼ Next Match  ▲ Previous Match  First Match

Score            Expect     Identities       Gaps         Strand
117 bits(63)     1e-27      65/66(98%)       0/66(0%)     Plus/Plus

Query  98300   CGCAAAAAGCGTGCCGCACCTCCACCTGAGGATGAAGAAGAGGATGAGTTCTACCGCAAA  98359
               ||||||||||||||||||||||||||||||||||||||||||||||| ||||||||||||
Sbjct  236575  CGCAAAAAGCGTGCCGCACCTCCACCTGAGGATGAAGAAGAGGATGATTTCTACCGCAAA  236634

Query  98360   AAGCGT  98365
               ||||||
Sbjct  236635  AAGCGT  236640


Range 10: 236575 to 236640  GenBank  Graphics         ▼ Next Match  ▲ Previous Match  First Match

Score            Expect     Identities       Gaps         Strand
117 bits(63)     1e-27      65/66(98%)       0/66(0%)     Plus/Plus

Query  98354   CGCAAAAAGCGTGCCGCACCTCCACCTGAGGATGAAGAAGAGGATGAGTTCTACCGCAAA  98413
               ||||||||||||||||||||||||||||||||||||||||||||||| ||||||||||||
Sbjct  236575  CGCAAAAAGCGTGCCGCACCTCCACCTGAGGATGAAGAAGAGGATGATTTCTACCGCAAA  236634

Query  98414   AAGCGT  98419
               ||||||
Sbjct  236635  AAGCGT  236640
```

```
Range 11: 236575 to 236640 GenBank Graphics        ▼ Next Match ▲ Previous Match ▲ First Match

Score          Expect   Identities     Gaps        Strand
117 bits(63)   1e-27    65/66(98%)     0/66(0%)    Plus/Plus

Query  98408   CGCAAAAAGCGTGCCGCACCTCCACCTGAGGATGAAGAAGAGGATGAGTTCTACCGCAAA  98467
               |||||||||||||||||||||||||||||||||||||||||||||||| |||||||||||
Sbjct  236575  CGCAAAAAGCGTGCCGCACCTCCACCTGAGGATGAAGAAGAGGATGATTTCTACCGCAAA  236634

Query  98468   AAGCGT  98473
               ||||||
Sbjct  236635  AAGCGT  236640


Range 12: 236575 to 236640 GenBank Graphics        ▼ Next Match ▲ Previous Match ▲ First Match

Score          Expect   Identities     Gaps        Strand
117 bits(63)   1e-27    65/66(98%)     0/66(0%)    Plus/Plus

Query  98462   CGCAAAAAGCGTGCCGCACCTCCACCTGAGGATGAAGAAGAGGATGAGTTCTACCGCAAA  98521
               |||||||||||||||||||||||||||||||||||||||||||||||| |||||||||||
Sbjct  236575  CGCAAAAAGCGTGCCGCACCTCCACCTGAGGATGAAGAAGAGGATGATTTCTACCGCAAA  236634

Query  98522   AAGCGT  98527
               ||||||
Sbjct  236635  AAGCGT  236640
```

Notice the different regions of query making alignment to same base positions of the subject genome (236575 - 236640). It is evident from the alignments that there is no continuous match, but there is a 66 bp repeat region for which blast is able to cover the entire query sequence.

In the CDS of wsv178, there are repeat regions of 66 bp in AF332093 accession. These repeat regions are overlapping. Every repeat region shares a 12 bp sequence with previous repeat region. You can notice the blast alignments are all of 66 bp length (98138 – 98203, 98192 – 98257, ….. 98624 – 98689 and 98678 - 98746) except the last one which is of 69 bp length (as it includes stop codon). Below is the pair-wise sequence alignment for wsv178 from AF332093 and KX686117 accessions.

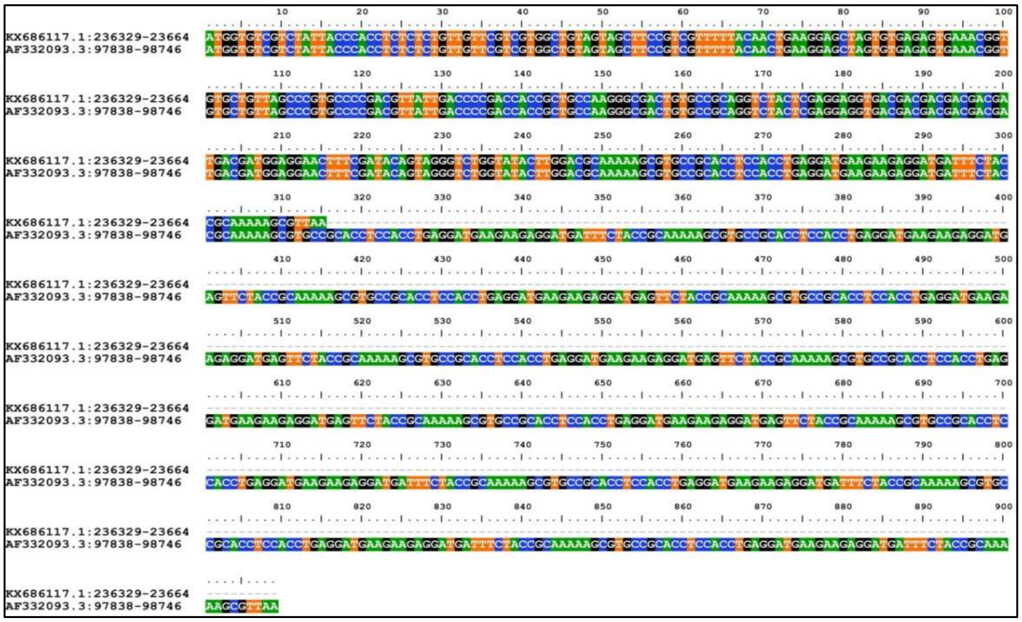

In fact, the KX686117 has lost some repeat regions. The MRF on account of mummer's exact match algorithm is able to report those lost regions. However, blast failed to report the missing regions because several repeat regions of query make multiple alignments with one repeat region of the subject. That is the reason for the blast reporting 100% query coverage with 100% identity for wsv178. In certain virus cases, number of repeats might be linked to virulence. Therefore obtaining accurate information becomes mandatory.

These are few cases of complete and partially missing coding sequences of WSSV where MRF proved to be performing better than blast search thus offering the potential to supplement blast search in virus genomics.

A2.1.2 Case study:Identifying deletion hotspots in isolates of WSSV with MRF

White Spot Syndrome Virus exhibits a highly variable genome size with respect to its geography and host. There is a length difference of about 28.6 Kb between shortest and longest genomes. To study this huge variation, we have collected 14 complete WSSV genome sequences (Table 5) and analyzed them using MRF tool to extract the missing proteins. The China isolate, AF332093 was kept as the reference and 13 other genomes were considered as queries. Nucleotide *fasta* sequences of the reference and a query along with the gff3 table of the reference were given as input for MRF tool.

Table A7. List of complete WSSV genomes used for comparative genomics analyses using MRF tool.

Accession	Country of Origin	Isolate	Host	Genome length, bp	Sample collection year	References
**MF768985**	Australia	AU	*Penaeus monodon*	285973	2016	(1)
**KX686117**	China	CN-Pc	*Procambarus clarkii*	300223	2015	(2)
**AF332093**	China	CN	*Marsupenaeus japonicus*	305119	1996	(3)
**KT995470**	China	CN02	*Procambarus clarkii*	294261	2010	(4)
**KT995471**	China	CN03	*Litopenaeus vannamei*	284148	2010	(4)
**KT995472**	China	CN01	*Marsupenaeus japonicus*	309286	1994	(4)
**KY827813**	China	CN04	*Marsupenaeus japonicus*	281054	2012	(5)
**KR083866**	Egypt	EG3	-	305119	2014	-
**KU216744**	Mexico	MEX2008	*Litopenaeus vannamei*	293183	2008	(6)

**AF440570**	Taiwan	Taiwan	*Penaeus monodon*	307287	1994	(7)
**AF369029**	Thailand	TH	*Procambarus clarkii*	292967	1996	(8)
**MG702567**	India	IN_AP4RU	*Litopenaeus vannamei*	280591	2013	(9)
**JX515788**	Korea	K-LV1	*Litopenaeus vannamei*	295884	2011	(10)
**MH090824**	Ecuador	EC-15098	*Litopenaeus vannamei*	288997	2015	(11)

A2.1.2.1 Deletions in WSSV genomes

The length of fourteen complete WSSV genomes analysed in this study varied from 280,591 to 309,286 base pairs. The GC content is mostly uniform ranging from 40.85 % to 41.08 %. The 14 isolates belong to 9 different countries. The genome length of the reference isolate, CN is higher than all other isolates except the CN01 and Taiwan.

Compared to CN isolate, the bases deleted in most other isolates are quite close to the absolute genome length difference between them (Fig A1). However, there are certain isolates which differ, for example, the MEX2008 isolate is only 11,936 bases shorter but lost about 13,183 bases compared to CN isolate. Similarly, the IN_AP4RU and CN-Pc isolates which are shorter by 24,528 and 4,896 bases but show deletions of 26,713 and 6,429 bases respectively when compared to CN isolate. Therefore the longer genome does not necessarily have all the bases of the shorter genomes. This is very much evident in the case of CN01 and Taiwan isolates which are 4,167 and 2,168 bases longer than the reference but still lack 970 and 772 bases respectively that are present in CN isolate.

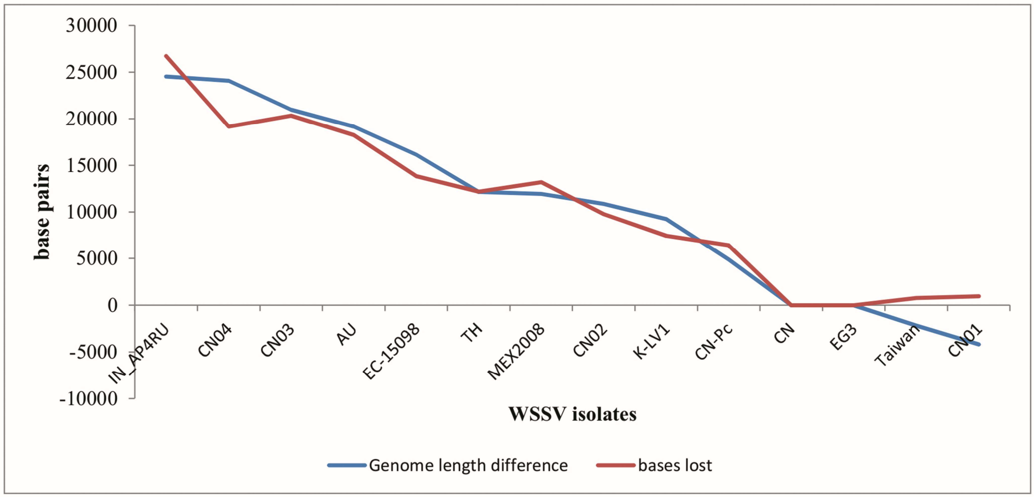

Figure A1. Comparison of genome length difference (blue line) and base length deleted (red line) in WSSV isolates with respect to CN isolate as identified by MRF tool.

The MRF tool helped us to document the completely and partially missing CDS in all the 13 WSSV isolates in comparison to CN isolate. Importantly, the missing CDS reported and commented in the study follow the nomenclature of the reference genome (in this case CN isolate). The CN isolate has 524 CDS, out of which 82 CDS are either completely or partially lost in at least one of the other WSSV isolates. There are 46 CDS that show complete deletion in at least one isolate compared to CN isolate. Some of them are deleted in only one isolate while others in as many as 10 isolates (Table A8).

Table A8. Completely deleted coding sequences and the number of isolates showing deletion in comparison to CN isolate.

**Completely deleted CDS of WSSV**	**No. of isolates showing deletion**
wsv060, wsv234, wsv235, wsv236, wsv242, wsv244, wsv319, wsv320, wsv337	1
wsv053, wsv074, wsv196, wsv245, wsv480, wsv500, wsv501, wsv503	2
wsv178, wsv179, wsv180, wsv338	3
wsv237	4
wsv238, wsv241	5
wsv239, wsv240, wsv462, wsv463, wsv485, wsv486, wsv487, wsv488	6
wsv481, wsv482, wsv483, wsv484	7
wsv489, wsv497	9
wsv490, wsv492, wsv493, wsv494, wsv495, wsv496, wsv498, wsv499	10

A2.1.2.2 Deletion hotspots

In this study, the sequence regions where a stretch of 3 CDS are completely lost in three or more isolates are defined as deletion hotspots. We report three deletion hotspots in WSSV genome which are wsv481/wsv499 (read as wsv481 through wsv499), wsv237/wsv241 and wsv178/wsv180 (Fig A2). As MRF also prints the missing genomic regions coordinates, it is possible to understand the position of deleted bases (5’ end or 3’end or within) in the CDS present in deletion hotspots.

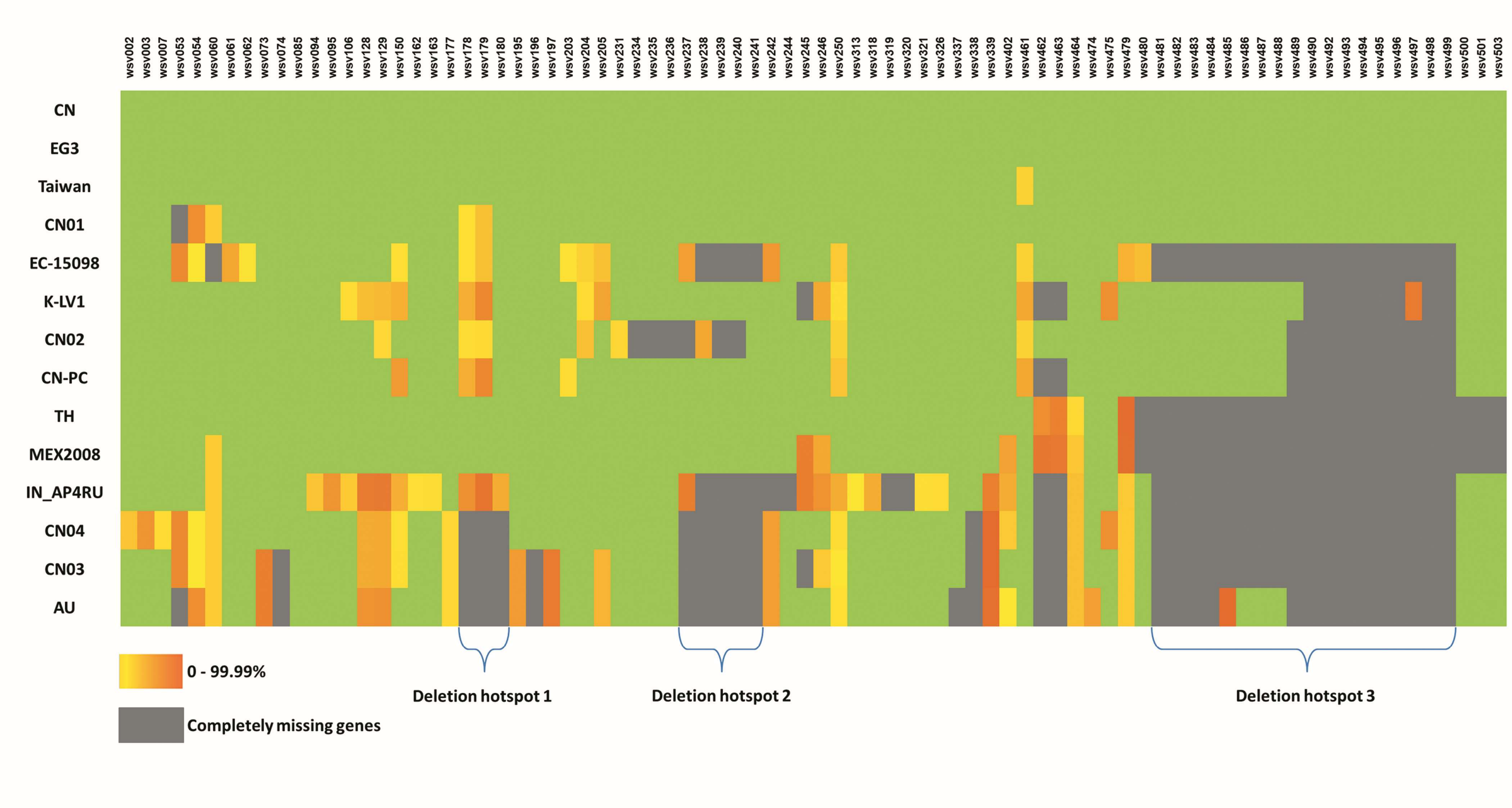

Figure A2. Depiction of the deleted regions within the coding sequences of WSSV genomes at the identified deletion hotspots. Grey represents completely missing genes, green represents completely present genes and yellow to orange represents partially missing genes based on percentage of the missing regions.

i. wsv178/wsv180.

All three proteins in this deletion hotspot are completely lost in CN03, CN04 and AU isolates but all 3 proteins showed only partial deletions in IN_AP4RU isolate. The CN01, EC-15098, K-LV1, CN02 and CN-Pc isolates showed partial deletions in wsv178 and wsv179 only while retaining full coding sequence for wsv180. The EG3, Taiwan, TH and MEX2008 are not affected by this deletion hotspot. The wsv178 displaying partial to complete deletion in various isolates is an immediate early protein.

ii. wsv237/wsv241.

The EG3, Taiwan, CN01, K-LV1, CN-Pc, TH, and MEX2008 isolates did not show any deletions in this deletion hotspot which consists of two important envelope proteins VP41A (wsv237) and VP52A (wsv238). The genomes of AU, IN_AP4RU, EC-15098, CN02, CN03 and CN04 isolates have lost this segment in variable proportions. The wsv237 is only partially lost in IN_AP4RU and EC-15098 isolates. Another envelope protein, VP41B (wsv242) which is adjacent to this deletion hotspot is partially lost in EC-15098, CN03, CN04 and AU isolates whereas the same CDS is completely lost in IN_AP4RU isolate. Other CDS nearer to this deletion hotspot, the wsv234/wsv236 are completely lost in only CN02 isolate and wsv244 is lost only in IN_AP4RU isolate. The CN02 retained wsv241 and lost partial sequence of wsv238.

iii. wsv481/wsv499.

The sequence region that encodes wsv481/wsv499 is lost in EC-15098, IN_AP4RU, MEX2008, TH and CN03 & CN04 isolates of China. These 6 isolates along with AU showed partial deletion (> 40%) of wsv479. The TH and MEX2008 isolates in particular share a common deletion of wsv480/wsv503, few more CDS extending on either side of deletion hotspot. The genomes of CN02, CN-Pc and AU isolates share a common deletion pattern ranging from wsv489 to wsv499. The K-LV1 isolate also shares similar deletion pattern except that wsv489 is present and wsv497 is partially (89%) lost. The CN01, Taiwan and EG3 isolates retained entire genomic region that included this deletion hotspot.

iv. Other deletions.

The annotated coding sequences and the genome-wide deleted regions in WSSV isolates have been depicted in S1 Fig. The CDS for wsv53 is deleted in CN01 and AU isolates whereas the CDS for wsv60 is deleted in EC-15098 isolate. Only CN03 and AU isolates showed deletion of CDS for wsv74 and wsv196 proteins. The coding sequence of two proteins, wsv319 and wsv320 are deleted in only IN_AP4RU isolate. The CDS of an envelope protein, VP62 (wsv338) is completely deleted in CN03, CN04 and AU isolates whereas the same isolates lost more than 95% of the CDS of another envelope protein, VP39 (wsv339). The VP39 was

identified as integument protein (12) and studies show targeting this protein increases the survival rate in shrimps (13). The coding sequence of protein, wsv337 was observed to be lost in only AU isolate. The genomes of K-LV1, CN-Pc, IN_AP4RU, CN03, CN04 and AU isolates completely lost the CDS for wsv462 and wsv463 proteins.

A2.2 USE CASE 2: African swine fever virus (ASFV)

The ASFV has several multigene families where copy number variation in a multigene family contributes to differences in genome length between isolates. It is interesting to compare MRF and blast in assessing deleted CDS between genomes of different isolates varying in genome length.

A2.2.1 Benchmarking MRF with Blast

A2.2.1.1 NCBI BLAST

Query: coding sequences of AM712239 genome [182,284 bp, Strain: Benin 97/1]
Subject: AM712240 genome [171,719 bp, Strain: OURT 88/3]

Search Parameters	
Program	blastn
Word size	28
Expect value	10
Hitlist size	100
Match/Mismatch scores	1,-2
Gapcosts	0,2.5
Low Complexity Filter	Yes
Filter string	L;m;
Genetic Code	1

Table A9: Consolidated results from Blast Hit table. The CDS with 100% identity and 100% query coverage are not listed below

CDS Name	Query coverage (%)	Percentage identity (%)
MGF 110-12L	No Hit	
MGF 360-6L	No Hit	
MGF 360L-10L	No Hit	
MGF 360-11L	No Hit	
MGF 505-1R	No Hit	
MGF 360-12L	No Hit	
MGF 360-13L	No Hit	
MGF 360-14L	No Hit	
MGF-505-2R	28	76.21
MGF-110-13L	69	86.06
MGF-505-3R	40	100
MGF-360-19R	79	100

A2.2.1.2 MRF

Query: AM712240 genome [171719 bp, Strain: OURT 88/3]
Reference: AM712239 genome [182284 bp, Strain: Benin 97/1]

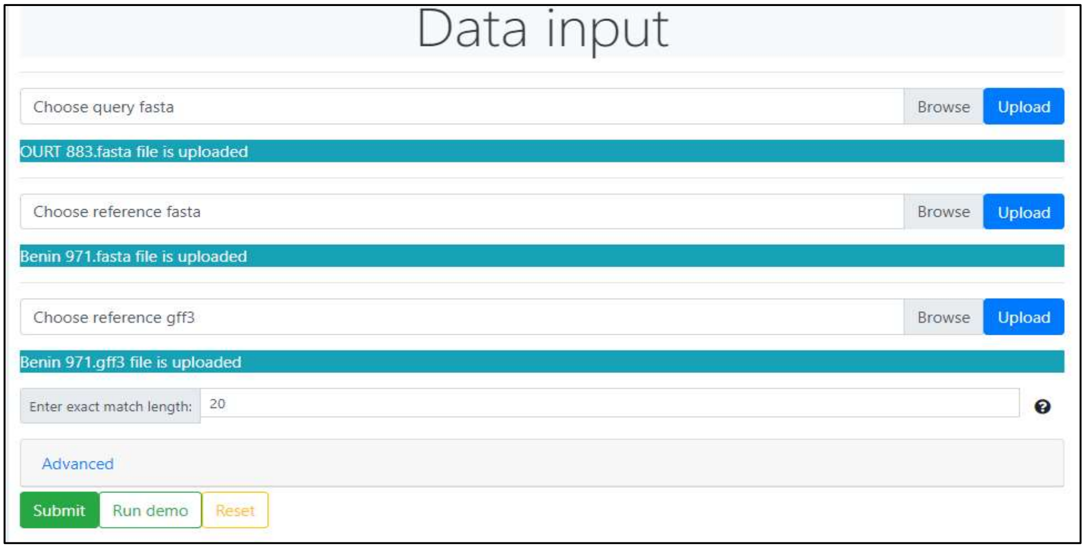

Table A10: Completely missing coding sequences

Missing Region Start	Missing Region End	CDS Start	CDS End	Missing Region Length	CDS Product	Protein id
6897	8278	7332	7643	312	MGF 110-12L	CAN10106.1
6897	8278	7721	8197	477	MGF 110-13L	CAN10107.1
10344	12289	10692	11819	1128	MGF 360-6L	CAN10110.1
19450	29527	19607	20677	1071	MGF 360L-10L	CAN10118.1
19450	29527	20703	21764	1062	MGF 360-11L	CAN10119.1
19450	29527	21971	23566	1596	MGF 505-1R	CAN10120.1
19450	29527	23616	24668	1053	MGF 360-12L	CAN10121.1
19450	29527	24840	25901	1062	MGF 360-13L	CAN10122.1
19450	29527	26073	27146	1074	MGF 360-14L	CAN10123.1
19450	29527	27352	28932	1581	MGF 505-2R	CAN10124.1

Table A11: Partially missing coding sequences

Missing Region Start	Missing Region End	Missing Region Length	CDS Length	CDS Start	CDS End	Missing CDS Proportion(%)	CDS Product	Protein id
6897	8278	115	381	8164	8544	30.18	MGF 110-14	CAN10108.1
19450	29527	38	1053	18435	19487	3.61	MGF 360-9L	CAN10117.1
19450	29527	509	843	29019	29861	60.38	MGF 505-3R	CAN10125.1
96065	96353	289	1905	95012	96916	15.17	pB602L	CAN10179.1
166580	166594	10	732	165858	166589	1.37	transcription factor SII homolog	CAN10237.1
180743	182284	228	1092	179879	180970	20.88	MGF 360-19R	CAN10256.1

A2.2.1.3 Consolidated results from Blast and MRF

Table A12: Consolidated results from Blast and MRF

CDS Name	Blast		MRF
	Query Coverage (%)	Percentage Identity (%)	% Lost
MGF 110-12L	No Hit		100
MGF 110-13L	69	86.06	100
MGF 360-6L	No Hit		100
MGF 360L-10L	No Hit		100
MGF 360-11L	No Hit		100
MGF 505-1R	No Hit		100
MGF 360-12L	No Hit		100
MGF 360-13L	No Hit		100
MGF 360-14L	No Hit		100
MGF 505-2R	28	76.21	100
MGF 110-14	94	96.95	30.18
MGF 505-3R	40	100	60.38
pB602L	100	95.1	15.17
MGF 360-19R	79	100	20.88

From the above table, it can be inferred that blast and MRF gave near similar results in case of completely missing coding sequences except for MGF110-13L. Both the tools agree on absence of 8 CDS in the OURT88/3 genome. But when it comes to partially missing CDS, further scrutiny is required before concluding.

The CDS ‘MGF 110-13L’ was reported as completely lost by MRF however blast reported an alignment with several mismatches and 69% query coverage.

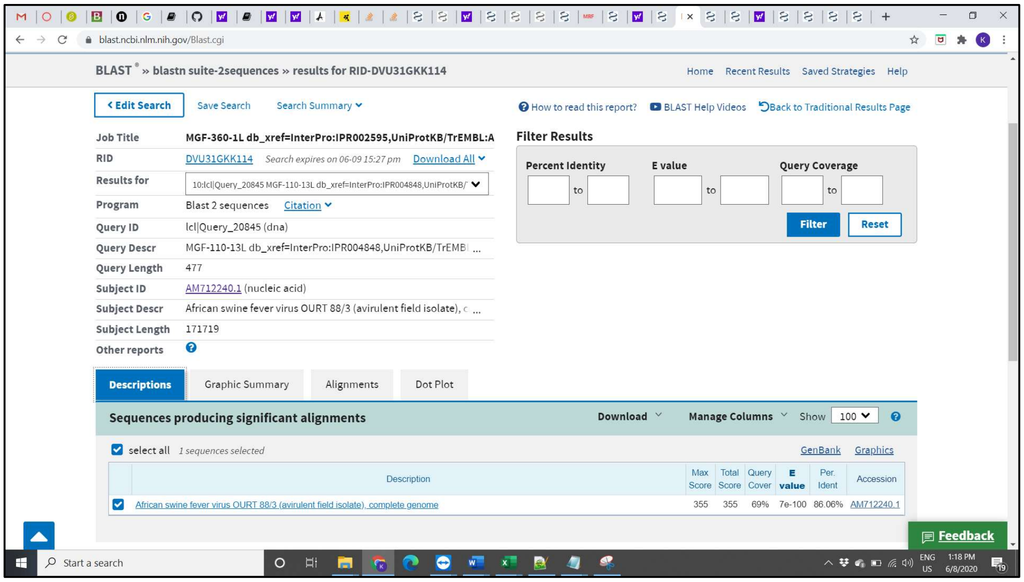

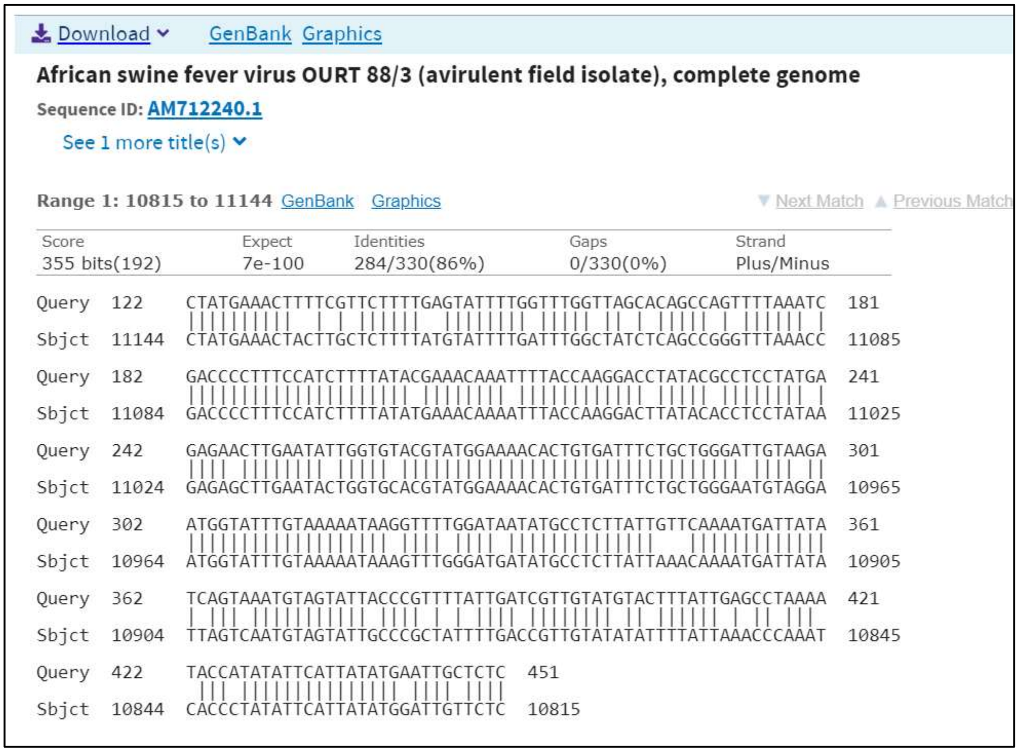

Here, the MRF was re-run with different parameters. As this genome display several mismatches, reducing exact match length might give better results. During re-run, the mum length was reduced to 15 and false match threshold was reduced to 10 as shown in the below snapshot.

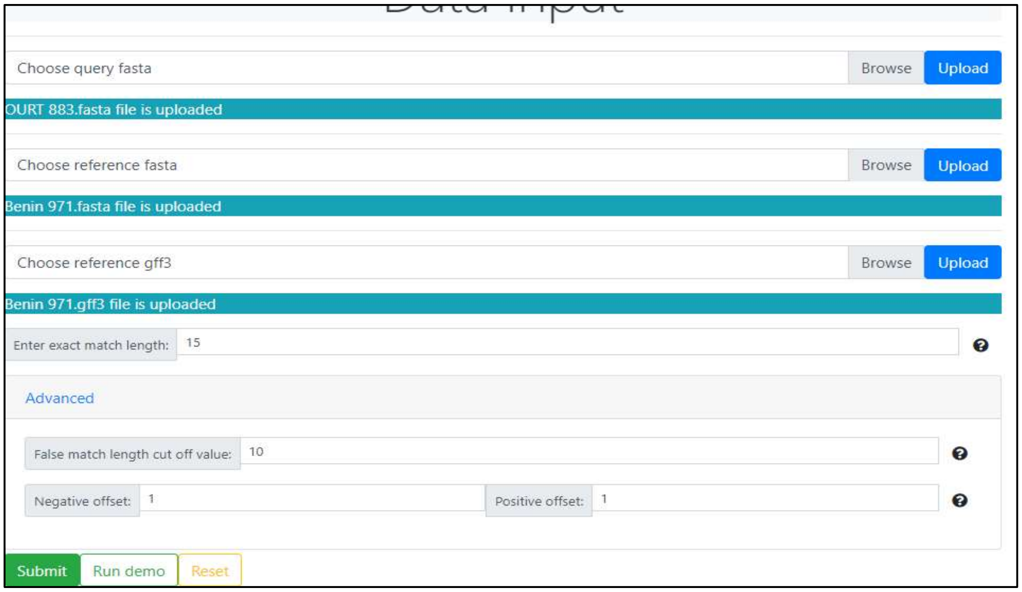

As per the revised results, the 'MGF 110-13L' was in 'partially missing CDS' list. But still, the percentage of missing CDS length is very high. This is due to the MRF's inability to find enough exact matches of length '15', as the CDS in the query genome has significant variations when compared to reference as evident

from the blast alignment. This is a situation where the ‘exact match’ algorithm suffers over blast or any other comparable program.

Missing Region Start	Missing Region End	Missing Region Length	CDS Length	CDS Start	CDS End	Missing CDS Proportion(%)	CDS Product	Protein id
7014	7756	36	477	7721	8197	7.55	MGF 110-13L	CAN10107.1
7772	7835	64	477	7721	8197	13.42	MGF 110-13L	CAN10107.1
7851	8278	347	477	7721	8197	72.75	MGF 110-13L	CAN10107.1

It is also worth noting that despite the reduced mum length, the coding sequences ‘MGF 110-12L, MGF 360-6L, MGF 360L-10L, MGF 360-11L, MGF 505-1R, MGF 360-12L, MGF 360-13L, MGF 360-14L’ are still reported as completely lost confirming their status.

Completely Missing Coding Sequences

Missing Region Start	Missing Region End	CDS Start	CDS End	Missing Region Length	CDS Product	Protein id	Notes
7014	7756	7332	7643	312	MGF 110-12L	CAN10106.1	
10352	12289	10692	11819	1128	MGF 360-6L	CAN10110.1	
19450	29527	19607	20677	1071	MGF 360L-10L	CAN10118.1	
19450	29527	20703	21764	1062	MGF 360-11L	CAN10119.1	
19450	29527	21971	23566	1596	MGF 505-1R	CAN10120.1	
19450	29527	23616	24668	1053	MGF 360-12L	CAN10121.1	
19450	29527	24840	25901	1062	MGF 360-13L	CAN10122.1	
19450	29527	26073	27146	1074	MGF 360-14L	CAN10123.1	
19450	29527	27352	28932	1581	MGF 505-2R	CAN10124.1	Member of multigene family 505 (A489R)%3B gene expression: late

In the case of ‘MGF 505-2R’, it was reported as lost by MRF whereas blast reported a poor alignment. It was noted from the list of CDS that the OURT88/3 genome does not have ‘MGF 505-2R’. However, blast with its heuristic nature of the algorithm is able to find a weak match.

African swine fever virus OURT 88/3 (avirulent field isolate), complete genome

Sequence ID: AM712240.1

See 1 more title(s)

Range 1: 29532 to 29981 GenBank Graphics ▼ Next Match ▲ Previous Match

Score	Expect	Identities	Gaps	Strand
233 bits(126)	1e-62	346/454(76%)	8/454(1%)	Plus/Plus

```
Query  1      ATGTTTTCCCTTCAAGACCTTTGCCGAAAGCATCTTTTTATTCTTCCCGATGTTTTTGGC  60
              ||||| |||||||| || || ||||| ||| |  |||  ||||||||  |    || ||
Sbjct  29532  ATGTTCTCCCTTCAGGAGCTCTGCCGGAAGAACATTTACATTCTTCCTTACCCCTTGGGT  29591

Query  61     GAGCATGTACTACAACGATTAGGACTGTATTGGAGATGTCACGGCTCCCTTCAACGCATA  120
               |||||||||| |||| | |||| ||||| ||||   | || || || |||||||| ||
Sbjct  29592  AAGCATGTACTTCAACAACTAGGGCTGTACTGGAAGGGACATGGATCTCTTCAACGAATC  29651

Query  121    GGAGACGACCACATACTCATACGACGGGATCTCATCCTTTCCACCAACGAGGCCTTAAGA  180
              ||||| |||||  ||||| ||| || ||| || ||| |||||| ||| ||||||||||||
Sbjct  29652  GGAGATGACCATGTACTCTTACAACAGGACCTGATCTTTTCCATCAATGAGGCCTTAAGA  29711

Query  181    ATGGCGGGAGAGGAAGGAAACAATGAAGTAGTAAAGCTCTTGTTACTGTGGAAGGGAAAT  240
              ||||||| ||| ||||||||||||||||||||||||||||||||||||||| |||||||
Sbjct  29712  ATGGCGGCAGAAGAAGGAAACAATGAAGTAGTAAAGCTCTTGTTACTGTGGGAGGGAAAC  29771

Query  241    CTTCATTACGCCGTCATAGGAGCCCTGCAGGGTGATCAATATGACCTGATCCATAAGTAT  300
              |||||||| ||| ||||||||||  |  |||| || | ||| ||||| |||||||| |||
Sbjct  29772  CTTCATTATGCCATCATAGGAGCTTTAGAGGGCGACCGATACGACCTTATCCATAAATAT  29831

Query  301    GAAAACCAAATCGGCGACTTTCATTTTAT-CTTACCATTGATTCAAGACGCG-AATACGT  358
               |  | ||||| || ||||  ||    || ||| || || || |||||| || ||  | |
Sbjct  29832  TATGAACAAATTGGGGACTGCCACAAGATTCTT-CCTTTAATCCAAGACCCGCAAATC-T  29889

Query  359    TTGAAAAATGCCACGCTTT-AG-AACGTTTTTGTGGTGTTTCATGTCTGCTAAAACATGC  416
              ||||||||||||| |  || || ||| |  | ||    ||  ||| ||  || |||||||
Sbjct  29890  TTGAAAAATGCCATGAATTGAGTAAC-TCCT-GTAACATTCGATGCCTTTTAGAACATGC  29947

Query  417    TACAAAATACAACATGCTCCCTATTCTCCAAAAA  450
                 |||| ||||||||||  ||||||| ||||||
Sbjct  29948  AGTAAAACACAACATGCTTTCTATTCTTCAAAAA  29981
```

When the coordinates of the hit region were further examined, it was found to be the sequence of ‘MGF 505-10R’. Here blast search erred by falsely reporting an alignment with MGF-505-10R as a hit to MGF 505-2R. The sequence of MGF 505-2R shares similarity with the sequence of MGF 505-10R. This is depicted in pair-wise sequence alignment given below.

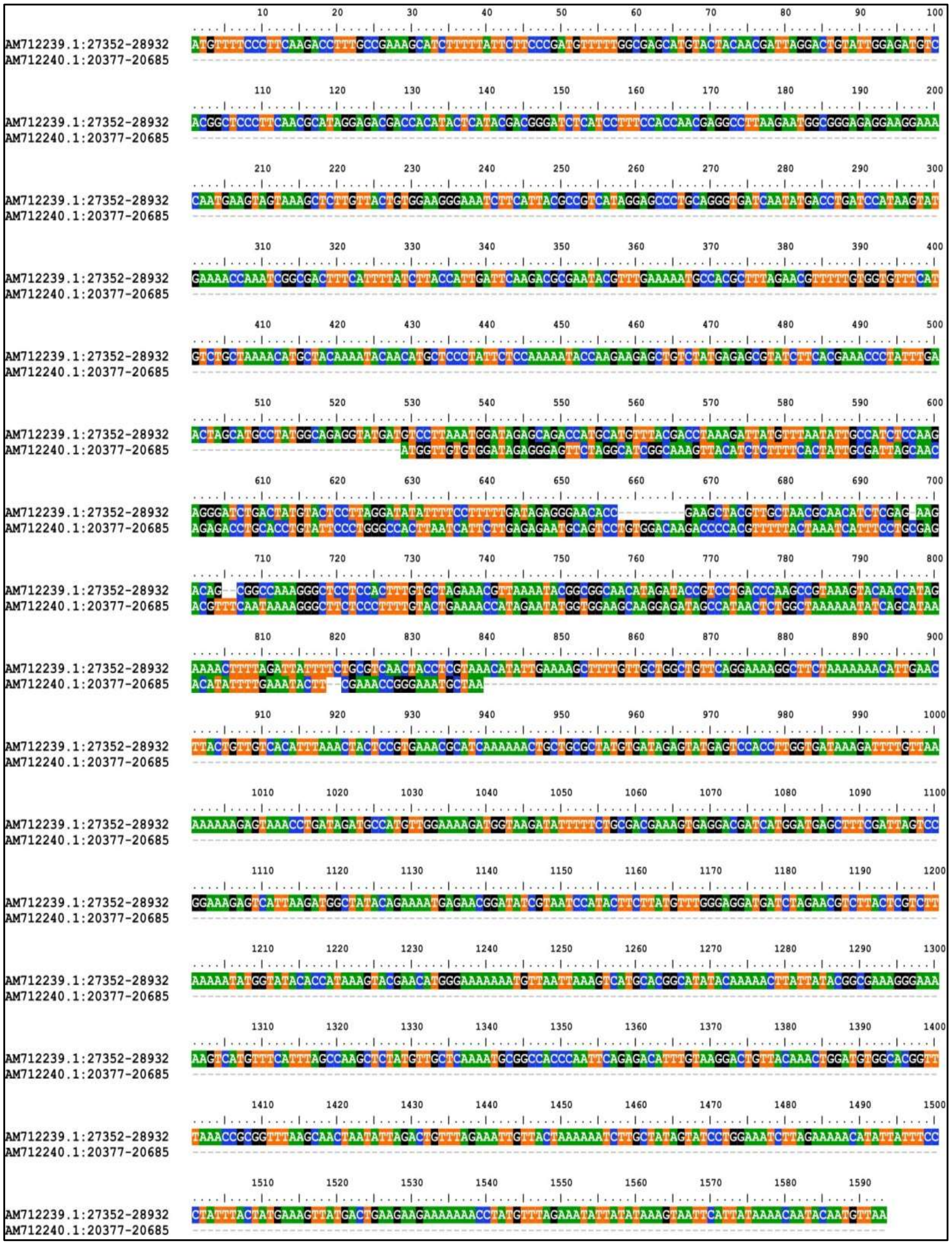

In fact, MGF 505-3R was reported as 60% lost by MRF and blast result too supported with a perfect alignment, even though for only 39% of the query sequence.

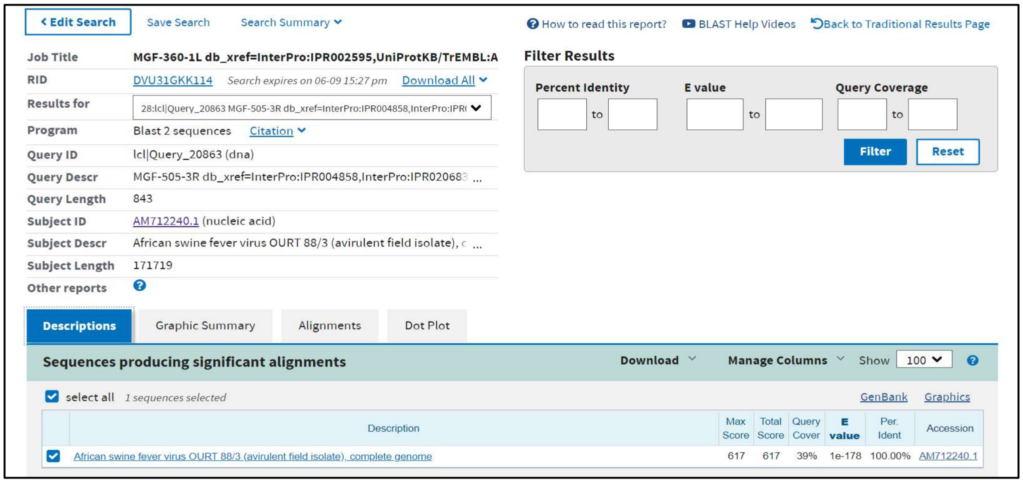

As for the other CDS with less than 50% deletion reported by the MRF, we can infer that those CDS have undergone significant mutations which can be further examined through blast alignments.

A2.2.2 Case study: Analyze multiple strains of ASFV with MRF and identify deletion hotspots

African Swine Fever Virus (ASFV) is a double-stranded DNA virus that causes hemorrhagic fever with high mortality in domestic pigs and related animals. Like WSSV, the ASFV also shows variation in genome length ranging between 170 to 193 Kb among different strains (14–16). Like ASFV, the major envelope proteins of WSSV are also not glycosylated (17), a feature that is uncommon in animal viruses. A vaccine for protection against ASFV is not available but immunization of pigs with attenuated isolates like OURT88/3 can confer protection against lethal challenges from virulent isolates (18). When compared with the highly virulent isolate, Benin 97/1, the OURT88/3 isolate contains major deletions in the MGF360 and MGF530/505 gene families which have the potential to modulate type 1 interferon response (19). The presence or absence of these gene families was attributed to the variation in genome length (16) as well as virus attenuation (19). Therefore, in the present study, we tested the utility of MRF tool for quick tabulation of these missing gene families in various isolates of ASFV in comparison to highly virulent, Benin 97/1.

While analyzing ASFV genomes with MRF tool, major emphasis was to highlight the deletion profile of 8 genes in MGF360 and MGF530/505 gene families (MGF360-10L to MGF360-14L and MGF530/505-1R to MGF530/505-3R), which are crucial for virulence of the pathogen. The deletion of these 8 genes has been documented in 17 complete genomes of AFSV in comparison to highly virulent, Benin 97/1 isolate and depicted in figure A3. The missing genes are presented as two groups, complete (100 %) and partially (>50 %) deleted genes.

The low virulent isolates, OURT88/3 and NHV shared an identical deletion profile for both complete and partially deleted genes. Both of them did not have genes, MGF360-10L to MGF360-14L and MGF530/505-1R to MGF530/505-2R. The MGF530/505-3R gene is only partially lost in these isolates. Another avirulent isolate, Ba71V also shared similar deletion profile as that of OURT88/3 and NHV isolates, except that it still retained MGF530/505-2R and MGF530/505-3R genes and the MGF360-14L gene is only partially lost.

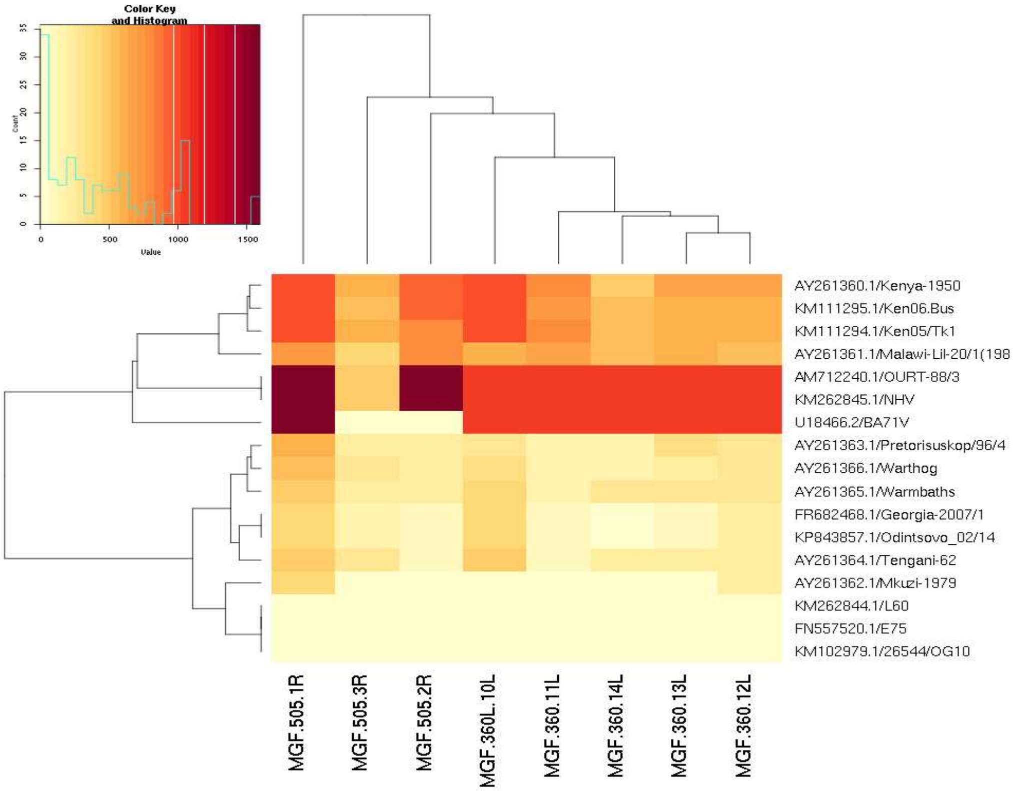

Figure A3. MRF batch mode was run by limiting the output to the 8 proteins (MGF360-10L - MGF360-14L, MGF530/505-1R - MGF530/505-2R) by calling the filter by protein name arguent (-N)

A single run of MRF tabulated all the completely and partially deleted genes in query genomes compared to reference genome. Therefore, we could notice that apart from the MGF360 and MGF530/505 genes, many isolates showed complete deletion of certain MGF110 family of genes. The MGF110-12L and MGF110-13L genes have been found to be completely deleted in OURT88/3, NHV, Ba71V and tengani isolates. However it has been reported that the presence or absence of MGF110 family of genes does not alter the state of virulence of the virus (20). The pDP71L gene is also lost in ken05, ken06, kenya1950 and malawi isolates. Several other genes showing partial deletions have been depicted in Figure A4.

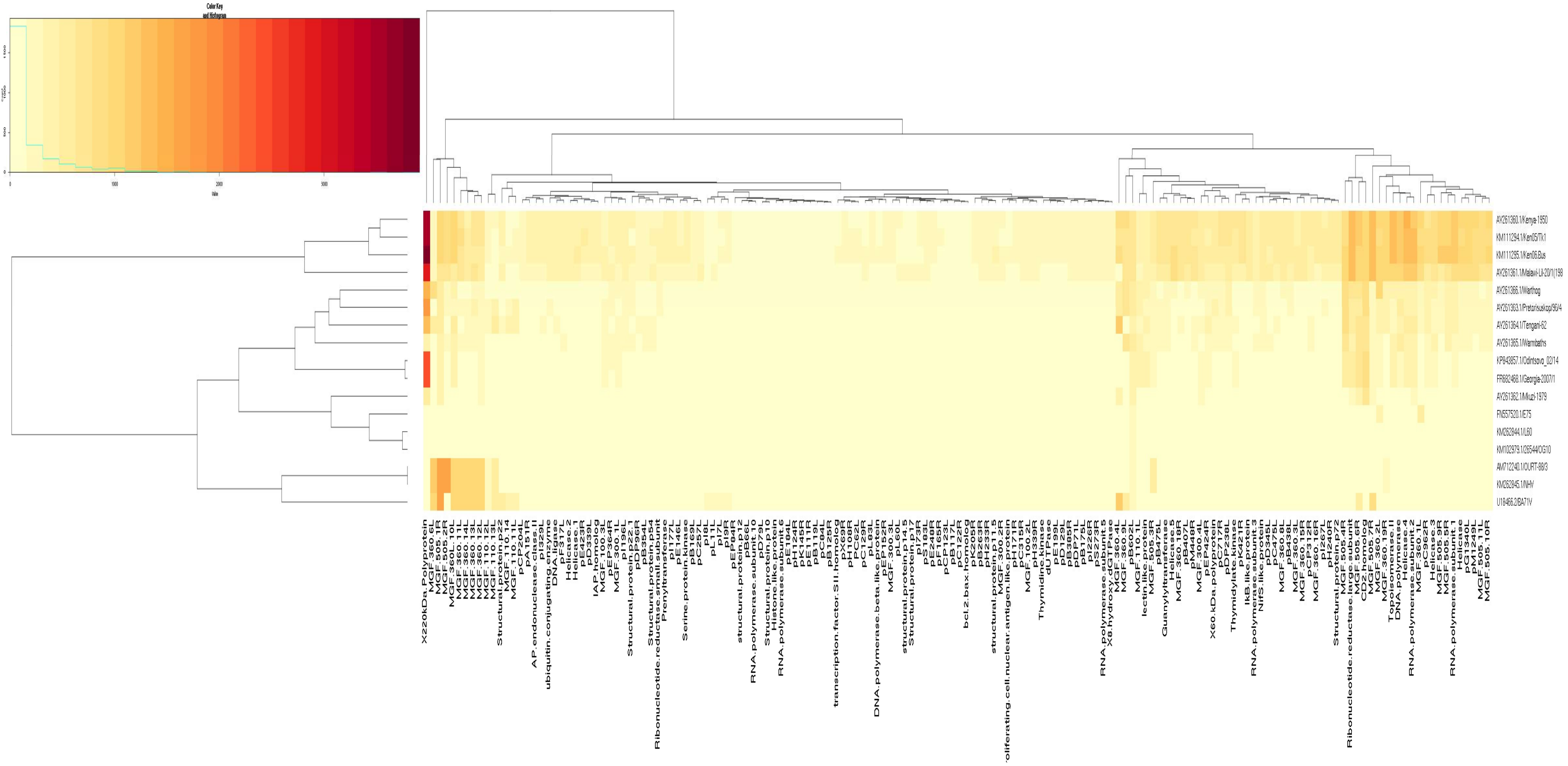

Figure A4. Completely and partially deleted CDS in ASFV isolates depicted using batch mode utility of MRF (-Y 100).

A2.3 USE CASE 3: HIV-1 genomes

From the earlier case studies, it is quite clear that running a blast search and then extracting CDS with no or partial hit is quite tedious. Instead, a quick approach would be to run MRF first to list the deleted CDS and then verify the ones in doubt through blast. This is demonstrated in this case study.

A2.3.1 Benchmarking Blast with MRF

A2.3.1.1 MRF

Query: KF766537 genome [genome size: 9,652 bp]
Reference: AF033819 genome [genome size: 9,181 bp]

Table A13: Reference CDS information

Accession	CDS start	CDS end	CDS length	Protein name	Protein id
AF033819.3	336	1838	1503	gag	AAC82593.1
AF033819.3	1631	4642	3012	pol	AAC82598.2
AF033819.3	4587	5165	579	vif	AAC82594.1
AF033819.3	5105	5341	237	vpr	AAC82595.1
AF033819.3	5377	5591	215	tat	AAC82591.1
AF033819.3	7925	7970	46	tat	AAC82591.1
AF033819.3	5516	5591	76	rev	AAC82592.1
AF033819.3	7925	8199	275	rev	AAC82592.1
AF033819.3	5608	5856	249	vpu	AAD20388.1
AF033819.3	5771	8341	2571	env	AAC82596.1
AF033819.3	8343	8714	372	nef	AAC82597.1

Analysis parameters

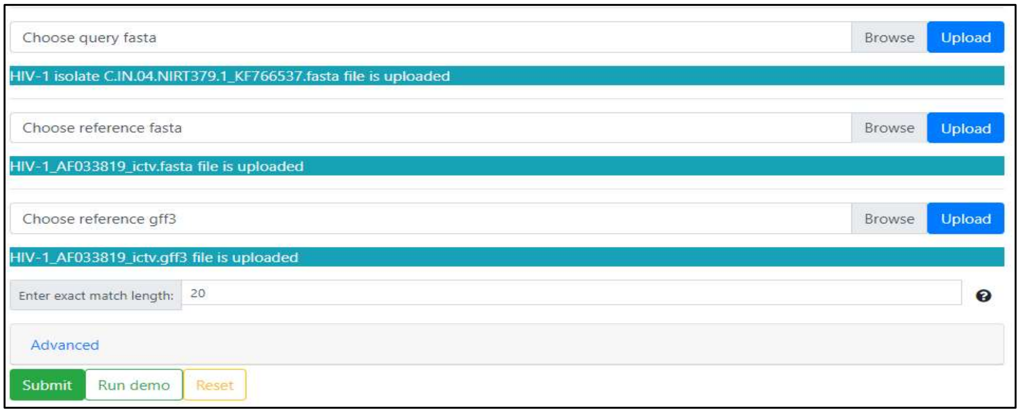

Table A14: Completely missing coding sequences

Missing Region Start	Missing Region End	CDS Start	CDS End	Missing Region Length	CDS Product	Protein id
5073	5366	5105	5341	237	Vpr	AAC82595.1
7906	8055	7925	7970	46	Tat	AAC82591.1

Table A15: Partially missing coding sequences (<5% deletions not shown)

Missing Region Start	Missing Region End	Missing Region Length	CDS Length	CDS Start	CDS End	Missing CDS Proportion(%)	CDS Product	Protein id
458	854	397	1503	336	1838	26.41	Gag	AAC82593.1
1060	1208	149	1503	336	1838	9.91	Gag	AAC82593.1
1292	1370	79	1503	336	1838	5.26	Gag	AAC82593.1
1394	1556	163	1503	336	1838	10.84	Gag	AAC82593.1
1714	1920	125	1503	336	1838	8.32	Gag	AAC82593.1
1714	1920	207	3012	1631	4642	6.87	Pol	AAC82598.2
3985	4183	199	3012	1631	4642	6.61	Pol	AAC82598.2
4608	4737	130	579	4587	5165	22.45	Vif	AAC82594.1
4761	4835	75	579	4587	5165	12.95	Vif	AAC82594.1
4856	5006	151	579	4587	5165	26.08	Vif	AAC82594.1
5073	5366	93	579	4587	5165	16.06	Vif	AAC82594.1
5396	5415	20	215	5377	5591	9.3	Tat	AAC82591.1
5442	5511	70	215	5377	5591	32.56	Tat	AAC82591.1
5547	5748	45	215	5377	5591	20.93	Tat	AAC82591.1
5547	5748	45	76	5516	5591	59.21	Rev	AAC82592.1

7906	8055	131	275	7925	8199	47.64	Rev	AAC82592.1
8080	8296	120	275	7925	8199	43.64	Rev	AAC82592.1
5547	5748	141	249	5608	5856	56.63	Vpu	AAD20388.1
5781	5871	76	249	5608	5856	30.52	Vpu	AAD20388.1
6251	6496	246	2571	5771	8341	9.57	Env	AAC82596.1
6525	6748	224	2571	5771	8341	8.71	Env	AAC82596.1
6772	7171	400	2571	5771	8341	15.56	Env	AAC82596.1
7549	7820	272	2571	5771	8341	10.58	Env	AAC82596.1
7906	8055	150	2571	5771	8341	5.83	Env	AAC82596.1
8080	8296	217	2571	5771	8341	8.44	Env	AAC82596.1
8323	8554	212	372	8343	8714	56.99	Nef	AAC82597.1
8584	8608	25	372	8343	8714	6.72	Nef	AAC82597.1
8640	8687	48	372	8343	8714	12.9	Nef	AAC82597.1

A2.3.1.2 NCBI Blast

Query: Coding sequences of AF033819 genome [genome size: 9,181 bp]
Subject: KF766537 genome [genome size: 9652 bp]

Search Parameters	
Program	blastn
Word size	28
Expect value	10
Hitlist size	100
Match/Mismatch scores	1,-2
Gapcosts	0,2.5
Low Complexity Filter	Yes
Filter string	L;m;
Genetic Code	1

Table A16: Blast output consolidated from Hit table

query acc.ver	subject acc.ver	Percentage identity (%)	Query coverage (%)
gag	KF766537.1	85.572	100
pol	KF766537.1	88.922	100
tat	KF766537.1	83.796	82
rev	KF766537.1	85.526	21
vpu	KF766537.1	87.578	64
env	KF766537.1	80.466	95
nef	KF766537.1	81.963	99

A2.3.1.3 Comparison between blast and MRF output

Table A17: Comparison between blast and MRF output

CDS	Blast		MRF
	Query coverage (%)	percentage identity (%)	% Lost
gag	100	85.572	60.74
pol	100	88.922	13.48
vif	No Hit	No Hit	77.54
vpr	No Hit	No Hit	100
tat	82	83.796	69.34
rev	21	85.526	84.33
vpu	64	87.578	87.15
env	95	80.466	58.69
nef	99	81.963	76.61

Both blast and MRF report the CDS *vpr* as absent in the KF766537 genome. For *vif* gene, blast could not find a hit and MRF reported 77.54% deletion. For other CDS too, MRF reported large deletions and blast reported less than 90% similarity at varying query coverage.

The default word size of 28 for blastn search and the default exact match length of 20 for MRF run might be too high for RNA viruses having too many mutations. Therefore both blastn search and MRF run were performed again with reduced word length and exact match length respectively.

A2.3.1.4 MRF run with reduced exact match length (10):

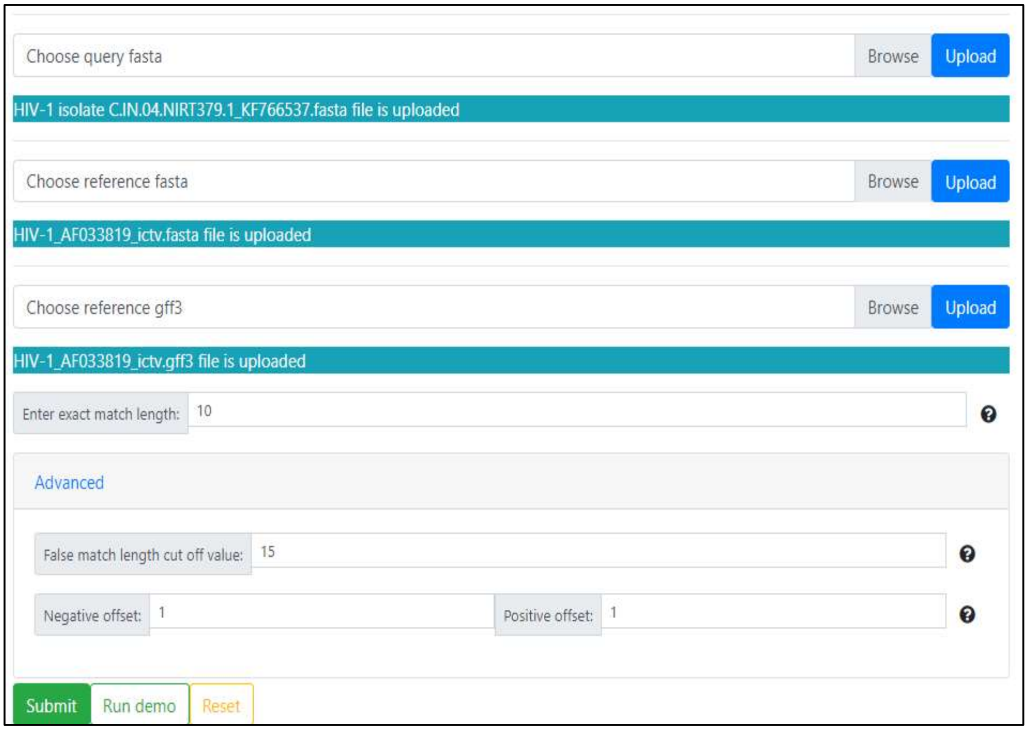

Table A18: Partially missing coding sequences (< 2% not shown)

Missing Region Start	Missing Region End	Missing Region Length	CDS Length	CDS Start	CDS End	Missing CDS Proportion(%)	CDS Product	Protein id
669	755	87	1503	336	1838	5.79	Gag	AAC82593.1
1396	1517	122	1503	336	1838	8.12	Gag	AAC82593.1
4706	4737	32	579	4587	5165	5.53	Vif	AAC82594.1
4856	4956	101	579	4587	5165	17.44	Vif	AAC82594.1
5125	5240	41	579	4587	5165	7.08	Vif	AAC82594.1
5125	5240	116	237	5105	5341	48.95	Vpr	AAC82595.1
5267	5281	15	237	5105	5341	6.33	Vpr	AAC82595.1
5293	5317	25	237	5105	5341	10.55	Vpr	AAC82595.1
5442	5511	70	215	5377	5591	32.56	Tat	AAC82591.1
5547	5613	45	215	5377	5591	20.93	Tat	AAC82591.1
7906	7943	19	46	7925	7970	41.3	Tat	AAC82591.1
7959	7969	11	46	7925	7970	23.91	Tat	AAC82591.1
5547	5613	45	76	5516	5591	59.21	Rev	AAC82592.1
7906	7943	19	275	7925	8199	6.91	Rev	AAC82592.1
8092	8113	22	275	7925	8199	8	Rev	AAC82592.1
8130	8227	70	275	7925	8199	25.45	Rev	AAC82592.1
5624	5642	19	249	5608	5856	7.63	Vpu	AAD20388.1
5673	5688	16	249	5608	5856	6.43	Vpu	AAD20388.1
5728	5748	21	249	5608	5856	8.43	Vpu	AAD20388.1
5781	5871	76	249	5608	5856	30.52	Vpu	AAD20388.1
8385	8409	25	372	8343	8714	6.72	Nef	AAC82597.1
8451	8543	93	372	8343	8714	25	Nef	AAC82597.1
8584	8608	25	372	8343	8714	6.72	Nef	AAC82597.1
8640	8687	48	372	8343	8714	12.9	Nef	AAC82597.1

A2.3.1.5 NCBI blast search with reduced word length (11)

Search Parameters	
Program	blastn
Word size	11
Expect value	10
Hitlist size	100
Match/Mismatch scores	2,-3
Gapcosts	5,2
Low Complexity Filter	Yes
Filter string	L;m;
Genetic Code	1

Table A19: Blast output consolidated from Hit table

query acc.ver	subject acc.ver	% identity	% query coverage
gag	KF766537.1	85.03	100%
pol	KF766537.1	88.782	100%
vif	KF766537.1	86.183	100%
vpr	KF766537.1	81.857	100%
tat	KF766537.1	83.562	96%
rev	KF766537.1	76.344	97%
vpu	KF766537.1	79.913	91%
env	KF766537.1	79.318	97%
nef	KF766537.1	80.965	99%

A2.3.1.6 Comparison between Blast and MRF output

Table A20: Comparison between Blast and MRF output

CDS Name	Blast		MRF
	percentage identity (%)	Query coverage (%)	% Lost
gag	85.03	100%	36.65
pol	88.782	100%	2.72
vif	86.183	100%	48.87
vpr	81.857	100%	65.83
tat	83.562	96%	55.55
rev	76.344	97%	54.41
vpu	79.913	91%	61.85
env	79.318	97%	32.2
nef	80.965	99%	53.49

A2.3.1.7 Inference

From the above table, it can be inferred that all the CDS of AF033819 have mutated considerably from the KF766537 genome where both blast and MRF report significant differences among these two genomes. Since RNA viruses tend to acquire lots of mutations, MRF struggles to report the deletions accurately neither do the blast as its results are supported by several alignments which are rather spurious but true. So, a quick run in MRF shows none of the CDS in the KF766537 genome are intact and all have suffered mutations.

A2.3.2 Case Study: Tracking the *nef* gene in HIV-1 strains

Generally, the mutation rates in RNA genomes are higher than DNA genomes of viruses (21). Therefore, we have tested the suitability of MRF tool for handling RNA genomes of the Human Immunodeficiency Virus (HIV). The Negative factor (Nef) gene of HIV genome plays a crucial role in progression of HIV infected individuals towards Acquired Immune Deficiency Syndrome (AIDS) (22, 23). Attenuation of HIV was observed in mutated strains carrying deletions in the Nef gene, where the onset of AIDS was also delayed (24, 25). Here, we tracked the Nef gene in 9 HIV-1 type C strains from India (table A21) keeping Refseq strain (NC_001802.1) as reference, using MRF tool. Since HIV has an RNA genome with high mutation rates, the MRF was run with exact match length as 10 and false match length cutoff as 10 instead of the defaults.

When the deletions are analysed, it was observed that apart from *Nef* gene, other genes such as Envelope surface glycoprotein gp160 2C precursor, Gag-Pol genes have suffered the significant deletions. In the case of Envelope surface glycoprotein gp160 2C precursor, it was observed that this gene inherently contains the genes *Vpu, Rev and Tat* either completely or partially. So to get a clear picture about deletions in *Nef* gene, we restricted the output to *Nef, Vpu, Rev, Vpr and Tat* genes by using the *filter by protein name (N)* argument. This has revealed that all the compared genomes suffered about around 400 bp deletions with the least deletions in KP109484.1 at 358 bp

Table A21: List of complete HIV genomes used for comparative genomics analyses using MRF tool.

Accession	Name	Sampling year	Sequence length
KF766538	C.IN.04.NIRT379.2	2004	9651
KF766539	C.IN.04.NIRT379.3	2004	9652
KF766541	C.IN.05.NIRT723.1	2005	9690
KF766542	C.IN.05.NIRT723.2	2005	9673
KP109480	DEMC00IN005	2000	8920
KP109481	DEMC00IN006	2000	8931
KP109482	DEMC00IN007	2000	8904
KP109483	DEMC00IN008	2000	8940
KP109484	DEMC00IN009	2000	8891

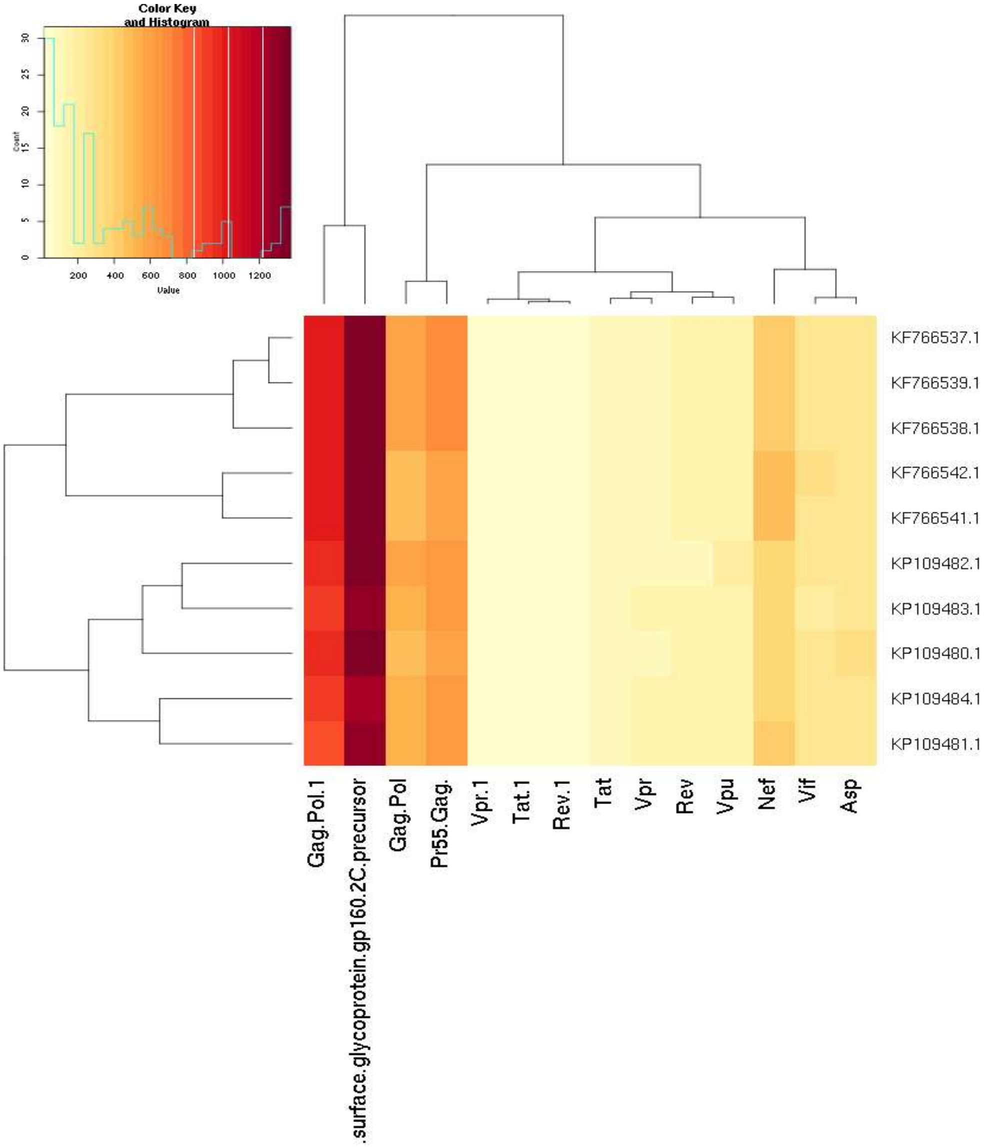

Figure A5. Deletions in CDS of HIV-1 subtype C strains observed as a heatmap.

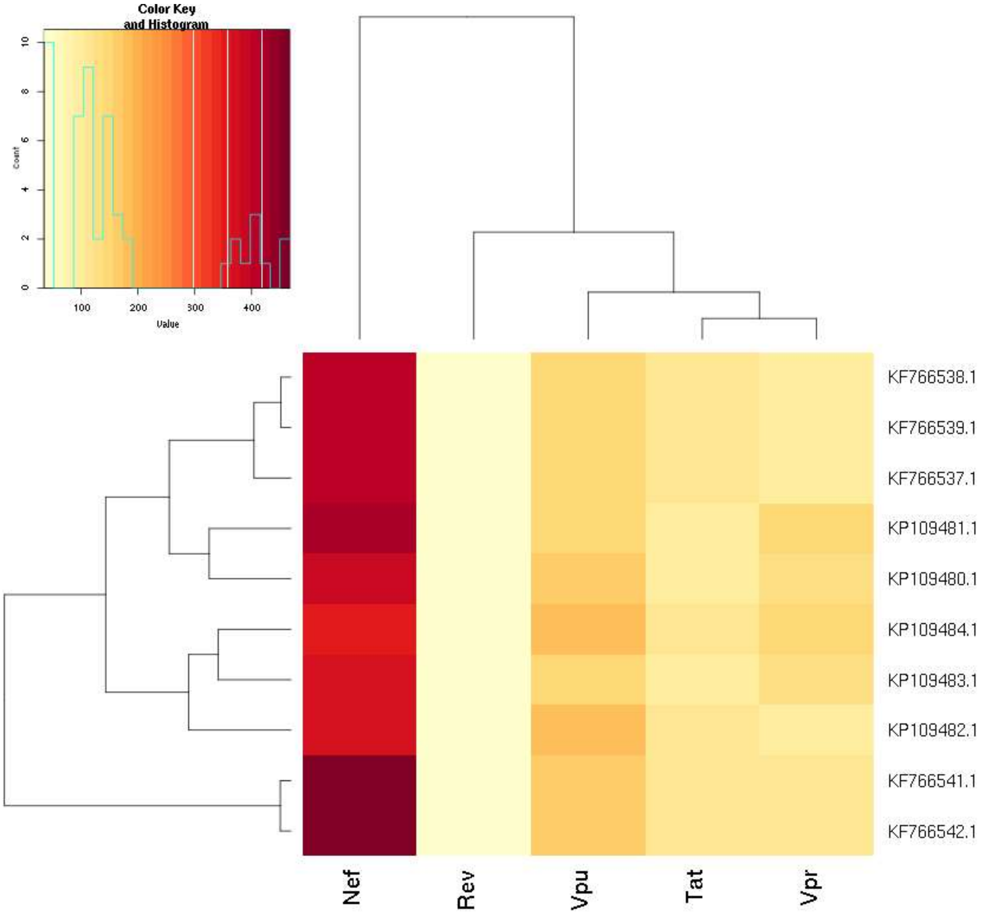

Figure A6. Deletions in selected CDS of HIV-1 subtype C strains observed as a heatmap. The CDS are restricted to those genes by applying 'filter by protein name' argument.

A2.4 USE CASE 4: SARS-COV2 genomes

The utility of MRF is better exemplified in the case of SARS-COV2 where lots of samples from different regions have been sequenced at rapid pace. Majority of research focuses on construction of phylogenies and evaluating the distances between the genomes but it is also important to know the CDS contributing differences between genomes.

Here, the utility of MRF is demonstrated by comparing a recently sequenced isolate from USA [MT506675] with the refseq isolate [NC_045512] from China.

A2.4.1 MRF

Query: MT506675 genome [genome size: 29,813 bp]
Reference: NC_045512 genome [genome size: 29,903 bp]

MRF parameters:

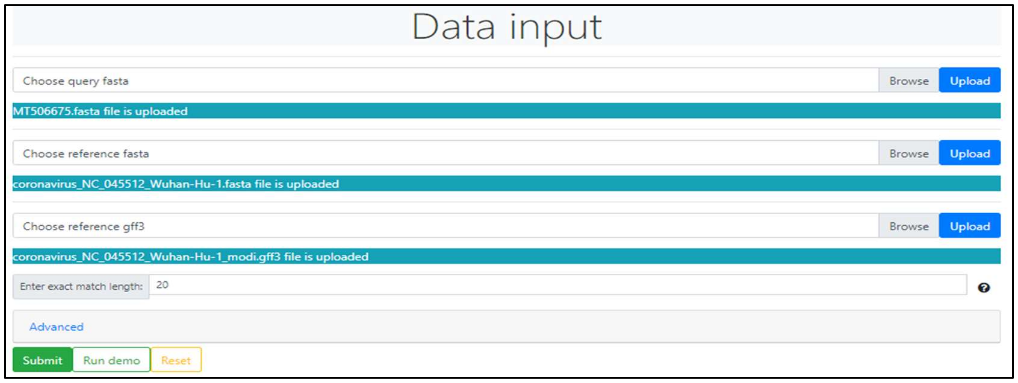

Table A22: Partially missing coding sequences

Missing Region Start	Missing Region End	Missing Region Length	CDS Length	CDS Start	CDS End	Missing CDS Proportion (%)	CDS Product	Protein id
19291	19339	49	8088	13468	21555	0.61	orf1ab polyprotein	YP_009724389.1
19367	19558	192	8088	13468	21555	2.37	orf1ab polyprotein	YP_009724389.1

A2.4.2 NCBI blast: batch

Query: Coding sequences of NC_045512 genome [genome size: 29,903 bp]
Subject: MT506675 genome [genome size: 29,813 bp]

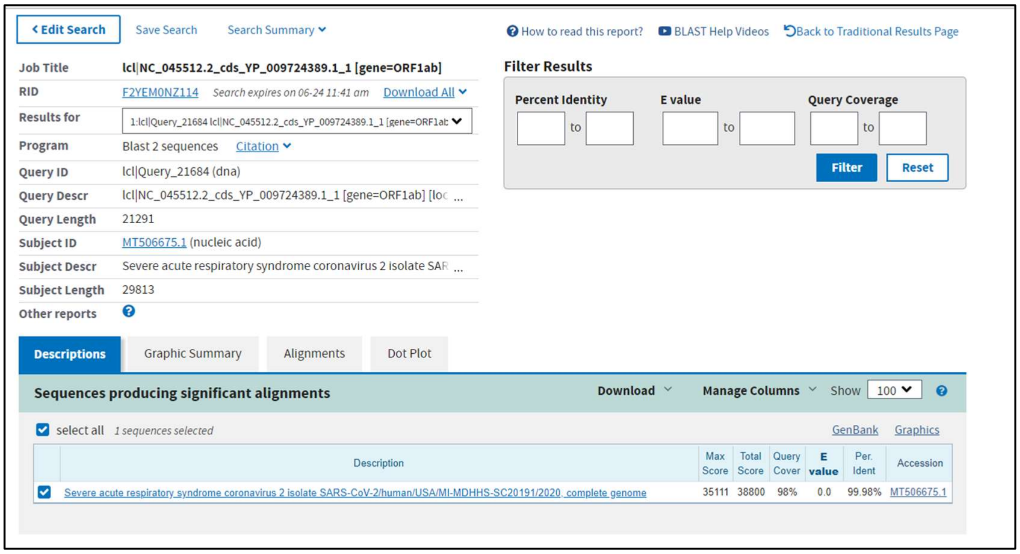

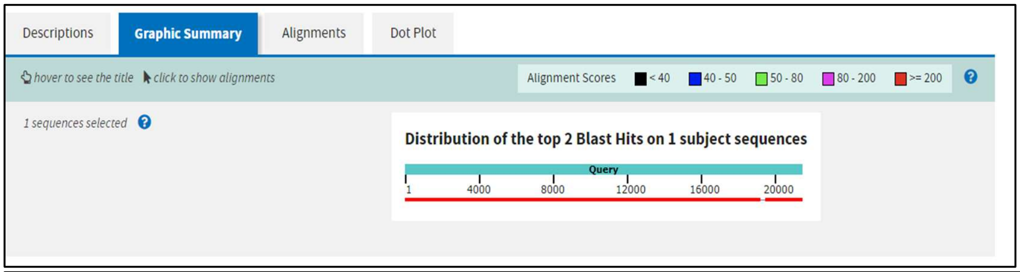

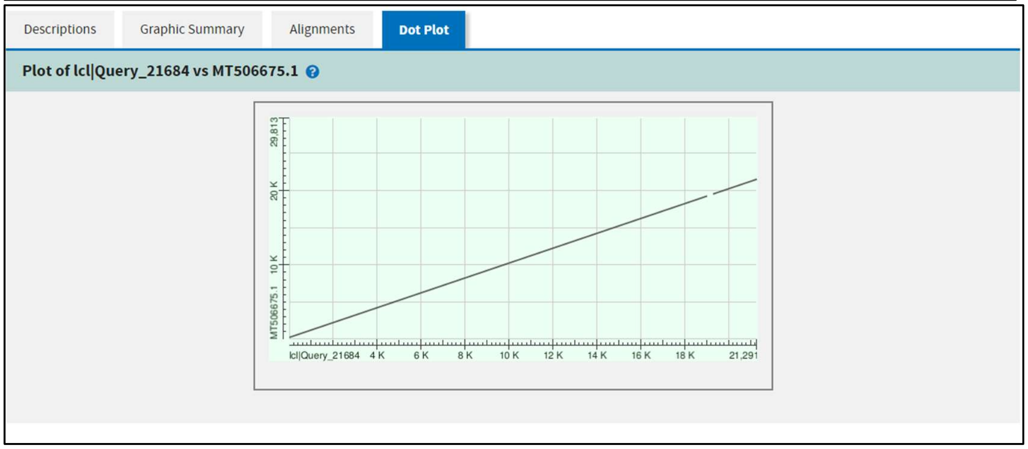

The ORF1ab sequence is reported as partially deleted by MRF. Similarly, blast search also showed the same in graphic summary and dotplot.

To conclude, MRF and blast can be used in conjunction to compare and list the deleted coding sequences in one genome with respect to another genome. MRF simplifies the procedure on account of its exact match algorithm and any ambiguous results could be verified with blast at least while handling pathogen genomes. In one go, MRF also identifies the missing genome sequence coordinates.

A2.4.3 Case study: Identification of Single Nucleotide Polymorphisms (SNP's)

MRF tool also identifies the substitutions (SNP's) as a part of identifying the deleted genomic regions. The substitutions are marked as X in the output file. The feature is demonstrated using one of the B.1.617 strain of SARS-COV2 as the query genome (Accession no. MZ140618) against the reference genome (Accession no. NC_045512). The B.1.617 lineage carries multiple mutations but two mutations namely L452R and E484Q in the spike protein is of significant concern and hence referred to as "double mutant", this variant has been suggested to more transmissible than the previous ones.

The results showed that MRF was able to detect the corresponding nucleotide changes of the above mutations as highlighted in the following images.

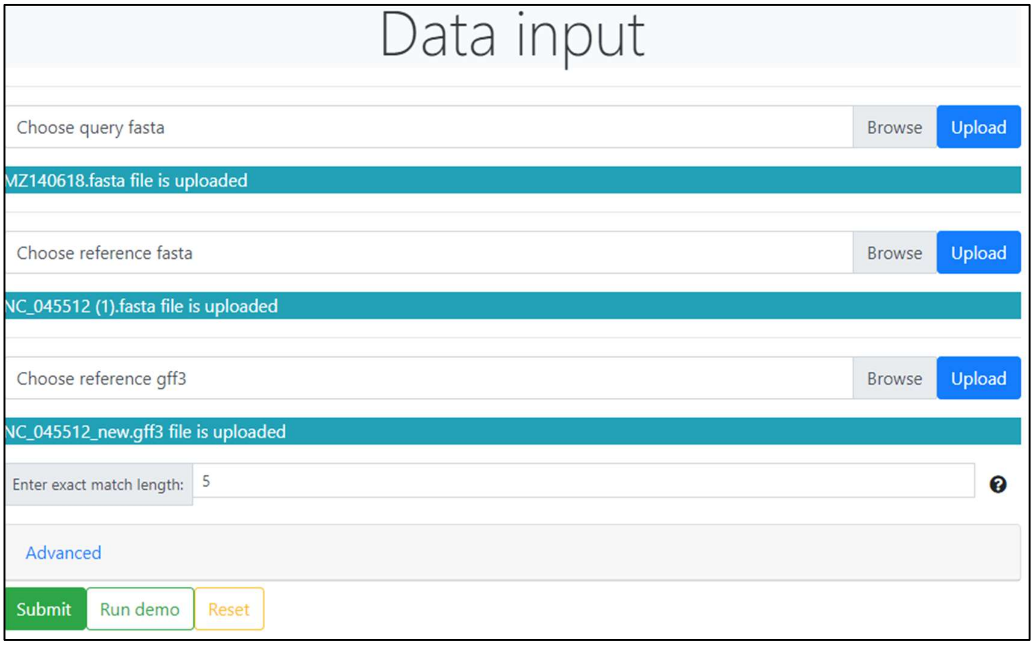

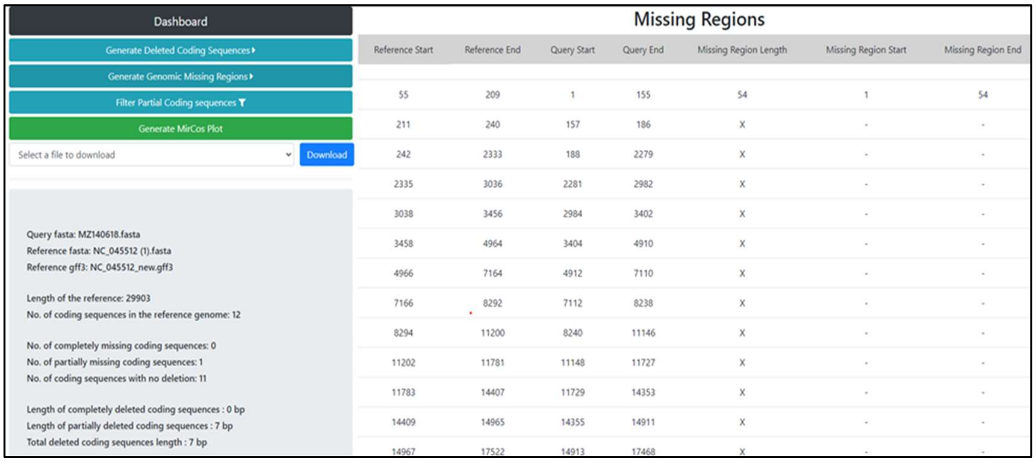

Reference Start	Reference End	Query Start	Query End	Missing Region Length	Missing Region Start	Missing Region End
55	209	1	155	54	1	54
211	240	157	186	X	-	-
242	2333	188	2279	X	-	-
2335	3036	2281	2982	X	-	-
3038	3456	2984	3402	X	-	-
3458	4964	3404	4910	X	-	-
4966	7164	4912	7110	X	-	-
7166	8292	7112	8238	X	-	-
8294	11200	8240	11146	X	-	-
11202	11781	11148	11727	X	-	-
11783	14407	11729	14353	X	-	-
14409	14965	14355	14911	X	-	-
14967	17522	14913	17468	X	-	-

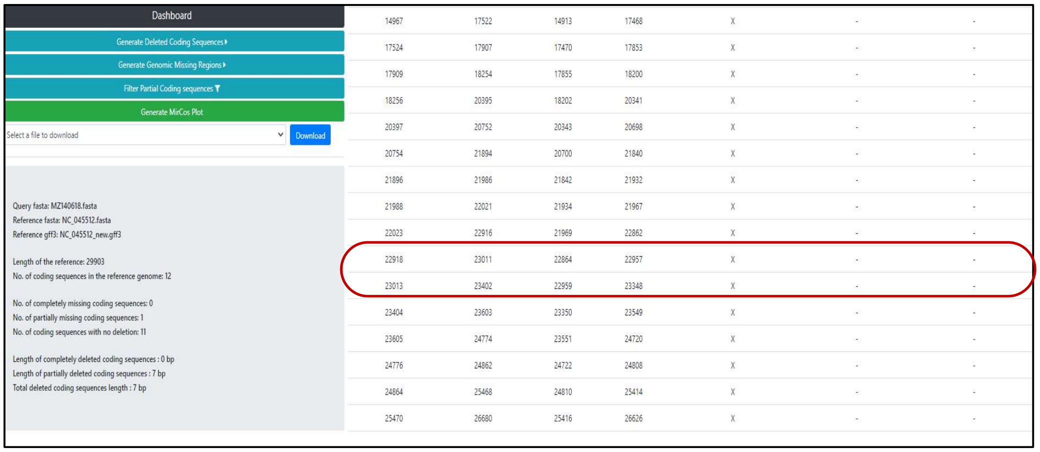

14967	17522	14913	17468	X	-	-
17524	17907	17470	17853	X	-	-
17909	18254	17855	18200	X	-	-
18256	20395	18202	20341	X	-	-
20397	20752	20343	20698	X	-	-
20754	21894	20700	21840	X	-	-
21896	21986	21842	21932	X	-	-
21988	22021	21934	21967	X	-	-
22023	22916	21969	22862	X	-	-
22918	23011	22864	22957	X	-	-
23013	23402	22959	23348	X	-	-
23404	23603	23350	23549	X	-	-
23605	24774	23551	24720	X	-	-
24776	24862	24722	24808	X	-	-
24864	25468	24810	25414	X	-	-
25470	26680	25416	26626	X	-	-

Eventhough MRF is able to detect single base pair changes (substitutions), it is currently not able to map these to amino acid changes. Another limitation being, MRF is able to identify only single base pair substitutions but not able to characterize indels.

A2.5 USE CASE 5: Marek's disease virus (Gallid herpesvirus 2)

To protect poultry from Marek's disease, many mutant strains of Marek's disease virus (MDV) have been developed as live vaccines. Out of many, the most successful has been the CVI988/Rispens strain. The key difference between the virulent and vaccine strain is an 18 bp deletion in *UL49* gene in the latter. The *UL49* gene codes for the protein vp22 which is involved in cell-to-cell transmission of the virus.

A virulent strain and a vaccine strain are compared with MRF and blast as well.

A2.5.1 MRF

Query: GHV-2 CVI988/Rispens vaccine strain [Accession no: DQ530348, 178311 bp]
Reference: GHV-2 RB-1B virulent strain [Accession no: EF523390, 178246 bp]

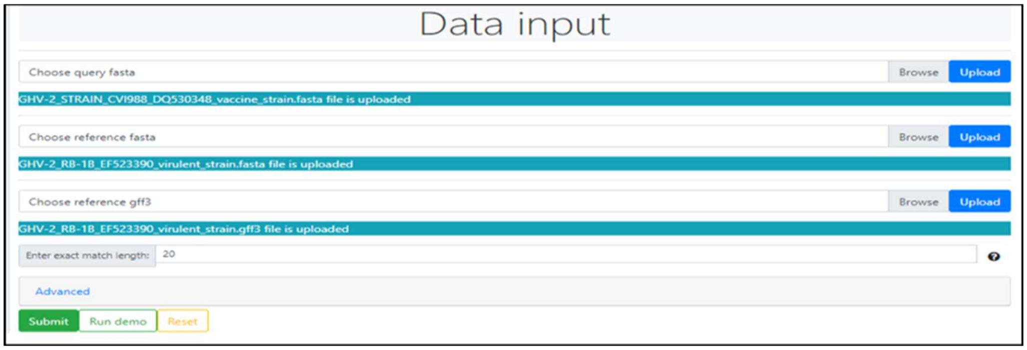

Completely Missing Coding Sequences

Missing Region Start	Missing Region End	CDS Start	CDS End	Missing Region Length	CDS Product	Protein id	Notes

Partially Missing Coding Sequences

Missing Region Start	Missing Region End	Missing Region Length	CDS Length	CDS Start	CDS End	Missing CDS Proportion(%)	CDS Product	Protein id	Notes
5597	5615	19	1020	4806	5825	1.86	R-LORF7	ABR13046.1	
5597	5615	19	240	5561	5800	7.92	hypothetical protein	ABR13049.1	
80556	80574	19	10074	79111	89184	0.19	UL36	ABR13118.1	
80693	80763	71	10074	79111	89184	0.70	UL36	ABR13118.1	
80961	81046	86	10074	79111	89184	0.85	UL36	ABR13118.1	
88098	88107	10	10074	79111	89184	0.10	UL36	ABR13118.1	
112197	112214	18	750	111918	112667	2.40	UL49	ABR13137.1	
113071	113083	13	288	112813	113100	4.51	UL49.5	ABR13138.1	
113071	113083	4	1311	113080	114390	0.31	UL50	ABR13139.1	
128176	128182	7	873	127629	128501	0.80	R-LORF14a	ABR13155.1	
136728	136746	19	1020	136518	137537	1.86	R-LORF7	ABR13170.1	

A2.5.2 Blast

Query: Coding sequences of GHV-2 RB-1B virulent strain [Accession no: EF523390, 178246 bp]

Subject: GHV-2 CVI988/Rispens vaccine strain [Accession no: DQ530348, 178311 bp]

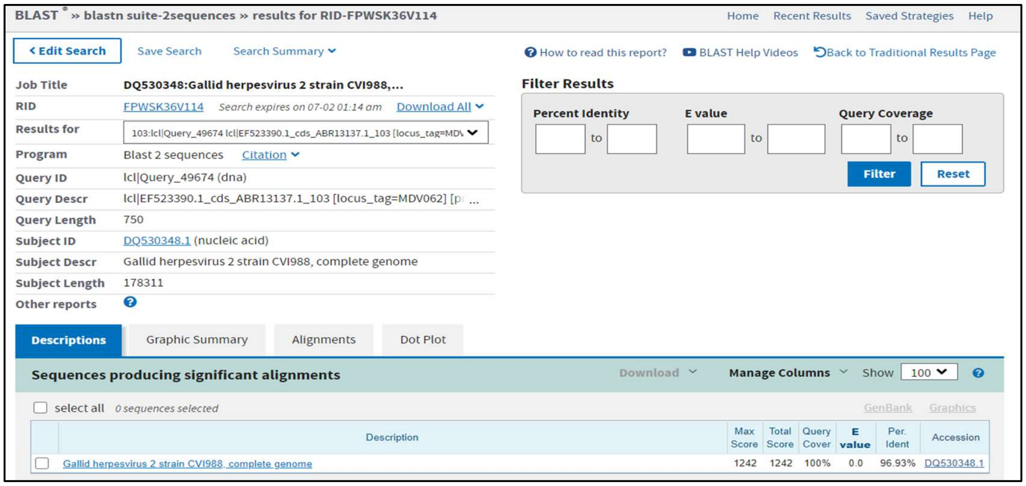

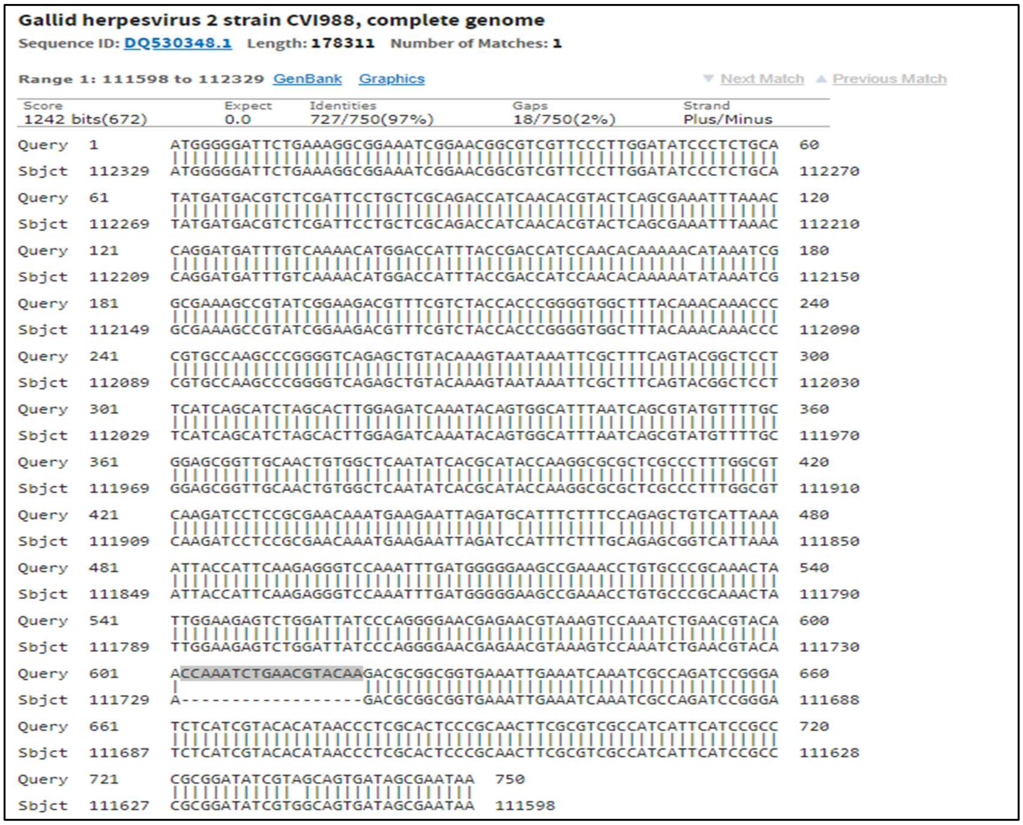

Gallid herpesvirus 2 strain CVI988, complete genome

Sequence ID: DQ530348.1 Length: 178311 Number of Matches: 1

Range 1: 111598 to 112329 GenBank Graphics ▼ Next Match ▲ Previous Match

Score	Expect	Identities	Gaps	Strand
1242 bits(672)	0.0	727/750(97%)	18/750(2%)	Plus/Minus

```
Query  1       ATGGGGGATTCTGAAAGGCGGAAATCGGAACGGCGTCGTTCCCTTGGATATCCCTCTGCA  60
               ||||||||||||||||||||||||||||||||||||||||||||||||||||||||||||
Sbjct  112329  ATGGGGGATTCTGAAAGGCGGAAATCGGAACGGCGTCGTTCCCTTGGATATCCCTCTGCA  112270

Query  61      TATGATGACGTCTCGATTCCTGCTCGCAGACCATCAACACGTACTCAGCGAAATTTAAAC  120
               ||||||||||||||||||||||||||||||||||||||||||||||||||||||||||||
Sbjct  112269  TATGATGACGTCTCGATTCCTGCTCGCAGACCATCAACACGTACTCAGCGAAATTTAAAC  112210

Query  121     CAGGATGATTTGTCAAAACATGGACCATTTACCGACCATCCAACACAAAAACATAAATCG  180
               ||||||||||||||||||||||||||||||||||||||||||||||||||| ||||||||
Sbjct  112209  CAGGATGATTTGTCAAAACATGGACCATTTACCGACCATCCAACACAAAAATATAAATCG  112150

Query  181     GCGAAAGCCGTATCGGAAGACGTTTCGTCTACCACCCGGGGTGGCTTTACAAACAAACCC  240
               ||||||||||||||||||||||||||||||||||||||||||||||||||||||||||||
Sbjct  112149  GCGAAAGCCGTATCGGAAGACGTTTCGTCTACCACCCGGGGTGGCTTTACAAACAAACCC  112090

Query  241     CGTGCCAAGCCCGGGGTCAGAGCTGTACAAAGTAATAAATTCGCTTTCAGTACGGCTCCT  300
               ||||||||||||||||||||||||||||||||||||||||||||||||||||||||||||
Sbjct  112089  CGTGCCAAGCCCGGGGTCAGAGCTGTACAAAGTAATAAATTCGCTTTCAGTACGGCTCCT  112030

Query  301     TCATCAGCATCTAGCACTTGGAGATCAAATACAGTGGCATTTAATCAGCGTATGTTTTGC  360
               ||||||||||||||||||||||||||||||||||||||||||||||||||||||||||||
Sbjct  112029  TCATCAGCATCTAGCACTTGGAGATCAAATACAGTGGCATTTAATCAGCGTATGTTTTGC  111970

Query  361     GGAGCGGTTGCAACTGTGGCTCAATATCACGCATACCAAGGCGCGCTCGCCCTTTGGCGT  420
               ||||||||||||||||||||||||||||||||||||||||||||||||||||||||||||
Sbjct  111969  GGAGCGGTTGCAACTGTGGCTCAATATCACGCATACCAAGGCGCGCTCGCCCTTTGGCGT  111910

Query  421     CAAGATCCTCCGCGAACAAATGAAGAATTAGATGCATTTCTTTCCAGAGCTGTCATTAAA  480
               ||||||||||||||||||||||||||||||||| ||||||||| ||||||| ||||||||
Sbjct  111909  CAAGATCCTCCGCGAACAAATGAAGAATTAGATCCATTTCTTTGCAGAGCGGTCATTAAA  111850

Query  481     ATTACCATTCAAGAGGGTCCAAATTTGATGGGGGAAGCCGAAACCTGTGCCCGCAAACTA  540
               ||||||||||||||||||||||||||||||||||||||||||||||||||||||||||||
Sbjct  111849  ATTACCATTCAAGAGGGTCCAAATTTGATGGGGGAAGCCGAAACCTGTGCCCGCAAACTA  111790

Query  541     TTGGAAGAGTCTGGATTATCCCAGGGGAACGAGAACGTAAAGTCCAAATCTGAACGTACA  600
               ||||||||||||||||||||||||||||||||||||||||||||||||||||||||||||
Sbjct  111789  TTGGAAGAGTCTGGATTATCCCAGGGGAACGAGAACGTAAAGTCCAAATCTGAACGTACA  111730

Query  601     ACCAAATCTGAACGTACAAGACGCGGCGGTGAAATTGAAATCAAATCGCCAGATCCGGGA  660
               |                  |||||||||||||||||||||||||||||||||||||||||
Sbjct  111729  A------------------GACGCGGCGGTGAAATTGAAATCAAATCGCCAGATCCGGGA  111688

Query  661     TCTCATCGTACACATAACCCTCGCACTCCCGCAACTTCGCGTCGCCATCATTCATCCGCC  720
               ||||||||||||||||||||||||||||||||||||||||||||||||||||||||||||
Sbjct  111687  TCTCATCGTACACATAACCCTCGCACTCCCGCAACTTCGCGTCGCCATCATTCATCCGCC  111628

Query  721     CGCGGATATCGTAGCAGTGATAGCGAATAA  750
               |||||||||||| |||||||||||||||||
Sbjct  111627  CGCGGATATCGTGGCAGTGATAGCGAATAA  111598
```

Both MRF and blast were able to detect this deletion, though it takes a strenuous search through alignments to detect this in blast output.

A2.6 SUMMARY

While similarity-search tools rely on the word length to build seed alignments and find similarities between two genomes, MRF uses the exact match length to find matching and missing genome regions. The blast or any other similarity search tool cannot directly output the missing CDS and their base coordinates in one go. This kind of output is highly valuable while studying pathogenic viruses. The MRF fills void for such tools in comparative genomics of viruses.

The MRF has three types of output, summary, tabular and graphic. The summary contains the number of coding sequences and the length of genome missing in query genome. The tables include completely missing coding sequences, partially missing coding sequences and genomic missing regions along with base coordinates. The graphic output is a MirCos plot of completely and partially missing coding sequences in query genome.

A highly-similar but length-varying genome (White Spot Syndrome Virus, WSSV), two clinical viruses (African Swine Fever Virus, ASFV and Human Immunodeficiency Virus-1, HIV-1), an emerging pathogen (2019 novel corona virus, 2019-nCoV) and a bird virus (Marek's disease virus, MDV) were taken to benchmark the efficiency of MRF over the commonly used similarity-search tool, the blast.

In case of highly-similar but length-varying genomes like WSSV, blast search ended up reporting high similarity between genomes and building several alignments between query and reference. Whereas the output of MRF could be inferred in a straightforward way to note the missing CDS and their base coordinates in genome. In addition, the circular nature and inconsistent annotations among WSSV genomes made MRF a better tool than blast to identify the genome-wide differences. In other use cases MRF gave comparable results to blast search while offering certain merits while handling genomes with high mutations.

Sometimes, blast algorithm might report false hits due to sequence alignments generated at random positions in the genome as was observed in the case of MGF 505-2R gene of ASFV. In such cases, the advanced features of MRF consisting of false match length and offset parameters gave an opportunity for users to control false matches and to perform several re-runs in less time. In genomes with high mutations like HIV-1, both MRF and blast algorithms struggled to avoid false matches/alignments. More number of mismatches between query and reference genomes led to several spurious alignments with blast and false matches with MRF. In case of HIV-1 genomes, utility of stringent offset parameters to control most of the false matches was further demonstrated.

Comparable results were obtained between MRF and blast search while analyzing 2019 nCoV genomes. Further, in the case of MDV, where an 18 bp deletion in UL49 gene differentiates a vaccine strain from a virulent strain, MRF was good enough to find this deletion in partially missing coding sequences table. Finally, MRF could handle the genome of the largest (2 Mbp) known virus, *Pandoravirus salinus*. Here a

genome with custom-deletions was given as query as against a full genome and MRF could successfully complete the analysis and print results.

The following table gives a brief comparison of blast and MRF based on the use cases mentioned in the previous sections

Table A23. Brief outline of the merits offered by MRF tool based on the results on tested use cases.

S. No.	Unique Capabilities of MRF	Proof of capability
1	MRF could analyse genomes having multiple coding sequences	Demonstrated with the case of WSSV which has 524 coding sequences
2	MRF could analyse multiple genomes	Demonstrated with the case of Corona virus where we have analysed 30,927 genomes and successfully printed the output
3	MRF handles the low-complexity regions and repeat-rich regions better than BLAST	Demonstrated with the case of WSSV
4	MRF handles the highly-similar genes better than BLAST	Demonstrated with the case of multi-gene families of African Swine Fever Virus
5	MRF is on par with BLAST in reporting short deletions but the results are easy to comprehend than BLAST	Demonstrated with the case of Marek's disease virus (18 bp deletion)
6	MRF has safety features to avoid false matches in cases of genomes displaying high mutations	Demonstrated with the case of Human Immunodeficiency Virus-1. This use case was explained in great detail in the user manual.
7	MRF can handle any virus genome	We have demonstrated a successful run with the case of the largest known virus, *Pandoravirus salinus* (2 Mb size).

References

1. Oakey HJ, Smith CS. 2018. Complete genome sequence of a white spot syndrome virus associated with a disease incursion in Australia. Aquaculture 484:152–159.

2. Jiang L, Xiao J, Liu L, Pan Y, Yan S, Wang Y. 2017. Characterization and prevalence of a novel white spot syndrome viral genotype in naturally infected wild crayfish, Procambarus clarkii, in Shanghai, China. Virusdisease 28:250–261.

3. Yang F, He J, Lin X, Li Q, Pan D, Zhang X, Xu X. 2001. Complete genome sequence of the shrimp white spot bacilliform virus. J Virol 75:11811–11820.

4. Li F, Gao M, Xu L, Yang F. 2017. Comparative genomic analysis of three white spot syndrome virus isolates of different virulence. Virus Genes 53:249–258.

5. Li Z, Li F, Han Y, Xu L, Yang F. 2016. VP24 is a chitin-binding protein involved in white spot syndrome virus infection. J Virol 90:842–850.

6. Rodriguez-Anaya LZ, Gonzalez-Galaviz JR, Casillas-Hernandez R, Lares-Villa F, Estrada K, Ibarra-Gamez JC, Sanchez-Flores A. 2016. Draft genome sequence of white spot syndrome virus isolated from cultured Litopenaeus vannamei in Mexico. Genome Announc 4:e01674--15.

7. Tsai M-F, Lo C-F, Van Hulten MCW, Tzeng H-F, Chou C-M, Huang C-J, Wang C-H, Lin J-Y, Vlak JM, Kou G-H. 2000. Transcriptional analysis of the ribonucleotide reductase genes of shrimp white spot syndrome virus. Virology 277:92–99.

8. Leu J-H, Tsai J-M, Wang H-C, Wang AH-J, Wang C-H, Kou G-H, Lo C-F. 2005. The unique stacked rings in the nucleocapsid of the white spot syndrome virus virion are formed by the major structural protein VP664, the largest viral structural protein ever found. J Virol 79:140–149.

9. Kumar KV, Shekhar MS, Otta SK, Karthic K, Kumar JA, Gopikrishna G, Vijayan KK. 2018. First Report of a Complete Genome Sequence of White spot syndrome virus from India. Genome Announc 6:e00055--18.

10. Chai CY, Yoon J, Lee YS, Kim YB, Choi T-J. 2013. Analysis of the complete nucleotide sequence of a white spot syndrome virus isolated from Pacific white shrimp. J Microbiol 51:695–699.

11. Restrepo L, Reyes A, Bajaña L, Betancourt I, Bayot B. 2018. Draft genome sequence of a white spot syndrome virus isolate obtained in Ecuador. Genome Announc 6:e00605--18.

12. Tsai J-M, Wang H-C, Leu J-H, Wang AH-J, Zhuang Y, Walker PJ, Kou G-H, Lo C-F. 2006. Identification of the nucleocapsid, tegument, and envelope proteins of the shrimp white spot syndrome virus virion. J Virol 80:3021–3029.

13. Akhila DS, Mani MK, Rai P, Condon K, Owens L, Karunasagar I. 2015. Antisense RNA mediated protection from white spot syndrome virus (WSSV) infection in Pacific white shrimp Litopenaeus vannamei. Aquaculture 435:306–309.

14. Blasco R, de la Vega I, Almazan F, Aguero M, Viñuela E. 1989. Genetic variation of African swine fever virus: variable regions near the ends of the viral DNA. Virology 173:251–257.

15. De Villiers EP, Gallardo C, Arias M, Da Silva M, Upton C, Martin R, Bishop RP. 2010. Phylogenomic analysis of 11 complete African swine fever virus genome sequences. Virology 400:128–136.

16. Dixon LK, Chapman DAG, Netherton CL, Upton C. 2013. African swine fever virus replication and genomics. Virus Res 173:3–14.

17. Van Hulten MCW, Reijns M, Vermeesch AMG, Zandbergen F, Vlak JM. 2002. Identification of VP19 and VP15 of white spot syndrome virus (WSSV) and glycosylation status of the WSSV major structural proteins. J Gen Virol 83:257–265.

18. Abrams CC, Goatley L, Fishbourne E, Chapman D, Cooke L, Oura CA, Netherton CL, Takamatsu H-H, Dixon LK. 2013. Deletion of virulence associated genes from attenuated African swine fever virus isolate OUR T88/3 decreases its ability to protect against challenge with virulent virus. Virology 443:99–105.

19. Reis AL, Abrams CC, Goatley LC, Netherton C, Chapman DG, Sanchez-Cordon P, Dixon LK. 2016. Deletion of African swine fever virus interferon inhibitors from the genome of a virulent isolate reduces virulence in domestic pigs and induces a protective response. Vaccine 34:4698–4705.

20. Agüero M, Blasco R, Wilkinson P, Vinuela E. 1990. Analysis of naturally occurring deletion variants of African swine fever virus: multigene family 110 is not essential for infectivity or virulence in pigs. Virology 176:195–204.

21. Duffy S. 2018. Why are RNA virus mutation rates so damn high? PLoS Biol 16:e3000003.

22. Foster JL, Garcia JV. 2008. HIV-1 Nef: at the crossroads. Retrovirology 5:84.

23. Gorry PR, McPhee DA, Verity E, Dyer WB, Wesselingh SL, Learmont J, Sullivan JS, Roche M, Zaunders JJ, Gabuzda D, others. 2007. Pathogenicity and immunogenicity of attenuated, nef-deleted HIV-1 strains in vivo. Retrovirology 4:66.

24. Schindler M, Münch J, Kutsch O, Li H, Santiago ML, Bibollet-Ruche F, Müller-Trutwin MC, Novembre FJ, Peeters M, Courgnaud V, others. 2006. Nef-mediated suppression of T cell activation was lost in a lentiviral lineage that gave rise to HIV-1. Cell 125:1055–1067.

25. Basmaciogullari S, Pizzato M. 2014. The activity of Nef on HIV-1 infectivity. Front Microbiol 5:232.
